# Exploring the Molecular Machinery of Denitrification in *Haloferax mediterranei* Through Proteomics

**DOI:** 10.3389/fmicb.2020.605859

**Published:** 2020-12-08

**Authors:** Javier Torregrosa-Crespo, Carmen Pire, David J. Richardson, Rosa María Martínez-Espinosa

**Affiliations:** ^1^División de Bioquímica y Biología Molecular, Departamento de Agroquímica y Bioquímica, Facultad de Ciencias, Universidad de Alicante, Alicante, Spain; ^2^Multidisciplinary Institute for Environmental Studies “Ramón Margalef”, University of Alicante, Alicante, Spain; ^3^School of Biological Sciences, University of East Anglia, Norwich, United Kingdom

**Keywords:** haloarchaea, detergents, liquid chromatography-mass spectrometry, denitrification, *Haloferax*, anaerobiosis, proteomics, electron transfer

## Abstract

Many proteins and enzymes involved in denitrification in haloarchaea can be inferred to be located between the cytoplasmic membrane and the S-layer, based on the presence of a Tat signal sequence and the orientation of the active site that some of these enzymes have. The membrane fraction of the haloarchaeon *Haloferax mediterranei* (R-4), grown under anaerobic conditions in the presence of nitrate, was solubilized to identify the respiratory proteins associated or anchored to it. Using Triton X-100, CHAPS, and n-Octyl-β-d-glucopyranoside at different concentrations we found the best conditions for isolating membrane proteins in micelles, in which enzymatic activity and stability were maintained. Then, they were subjected to purification using two chromatographic steps followed by the analysis of the eluents by NANO-ESI Chip-HPLC-MS/MS. The results showed that the four main enzymes of denitrification (nitrate, nitrite, nitric oxide, and nitrous oxide reductases) in *H. mediterranei* were identified and they were co-purified thanks to the micelles made with Triton X-100 (20% w/v for membrane solubilisation and 0.2% w/v in the buffers used during purification). In addition, several accessory proteins involved in electron transfer processes during anaerobic respiration as well as proteins supporting ATP synthesis, redox balancing and oxygen sensing were detected. This is the first characterization of anaerobic membrane proteome of haloarchaea under denitrifying conditions using liquid chromatography-mass spectrometry. It provides new information for a better understanding of the anaerobic respiration in haloarchaea.

## Introduction

In the absence of oxygen, the most energetically favorable respiratory pathway is denitrification: the reduction of nitrate (${\text{NO}}_{3}^{-}$) via nitrite (${\text{NO}}_{2}^{-}$), nitric oxide (NO), and nitrous oxide (N_2_O) to dinitrogen (N_2_) (Richardson, 2000; Zumft and Kroneck, 2006; Philippot et al., 2007; Bakken et al., 2012). Although there is extensive and detailed knowledge about this route, traditional studies have been restricted to a few model organisms, mostly bacteria that inhabit agricultural and forestry soils (Bergaust et al., 2008; Samad et al., 2016; Roco et al., 2017). However, the role of denitrification is less known in saline and hypersaline ecosystems. where the reduction of N-oxyanions is favored by two main factors: on the one hand, due to the high salt concentrations resulting in low oxygen solubility (Rodríguez-Valera et al., 1985; Oren, 2013); on the other hand, because of the increasing nitrate/nitrite concentrations due to anthropogenic activities (Martínez-Espinosa et al., 2007, 2011; Ochoa-Hueso et al., 2014; Torregrosa-Crespo et al., 2018). Moreover, saline, and hypersaline areas are currently increasing in size and prevalence as a result of desertification (Torregrosa-Crespo et al., 2018).

Hypersaline environments are dominated by haloarchaea when the salt concentration exceeds 16% (Andrei et al., 2012; Edbeib et al., 2016). In the last years, one of their members has been used as model organism for the study of denitrification: *Haloferax mediterranei*. It is an haloarchaeon able to carry out the complete reduction of nitrate to dinitrogen through the four key enzymes of denitrification: nitrate, nitrite, nitric oxide, and nitrous oxide reductases (Torregrosa-Crespo et al., 2018, 2019, 2020).

The enzyme catalyzing the first reaction, the respiratory nitrate reductase, catalyzes the reduction of nitrate to nitrite (${\text{NO}}_{3}^{-}$+2e^−^ +2H^+^ → ${\text{NO}}_{2}^{-}$+H_2_O). It is composed of two core subunits: a catalytic α subunit containing a molybdopterin cofactor (NarG) and a β subunit containing four [4Fe-4S] clusters (NarH) (Lledó et al., 2004). During the second step of denitrification, the periplasmic nitrite reductase catalyzes the reduction of nitrite to nitric oxide as follows: ${\text{NO}}_{2}^{-}$+2H^+^+e^−^ → NO+H_2_O. In *H. mediterranei*, this enzyme is the copper containing Nir-type (CuNiR), encoded by the gene *nirK*. Although the native form has never been purified, it was expressed homologously using *H. volcanii* as host (Esclapez et al., 2013). Its characterization indicated that it is a green copper-dependent nitrite reductase like NirK isolated from *H. denitrificans*. In terms of structure, it is a homotrimer, in which a monomer contains one type I Cu and one type II Cu sites (Esclapez et al., 2013). In CuNiR, the electron for nitrite reduction is supplied from a physiological redox partner, generally small electron-transfer proteins such as a cupredoxin (blue copper protein) or a cytochrome c (Nojiri, 2017). However, in case of *H. mediterranei*, it remains unknown which protein fulfills this function.

The third enzyme of denitrification is the nitric oxide reductase, producing nitrous oxide from nitric oxide: 2NO+2H^+^+2e^−^ → N_2_O+H_2_O. Bacterial respiratory Nors can be classified mainly as short-chain respiratory Nors (scNors) or long-chain respiratory Nors (lcNors) (Torregrosa-Crespo et al., 2017): on one hand, scNors are characterized by a transmembrane catalytic subunit forming a complex with a *c-*type cytochrome, that is the electron receiving domain. These NorBC complexes are also known as cNors; on the other hand, lcNors contain a single subunit, NorZ, also named as qNors because they accept electrons directly from the reduced quinol pool (Hendriks et al., 2000). Since the *H. mediterranei* genome had a chromosomal gene encoding for a putative nitric oxide reductase annotated as *norB* (Han et al., 2012; Becker et al., 2014), it was supposed that it had a nitric oxide reductase type scNor. However, subsequent bioinformatics studies revealed that *H. mediterranei* respiratory NO-reductase is a non-electrogenic single subunit qNor closely related to the bacterial NorZs that derive their electrons directly from the quinone pool (Torregrosa-Crespo et al., 2017).

For the final step, the periplasmic enzyme nitrous oxide reductase NosZ catalyzes the reduction of nitrous oxide to dinitrogen: N_2_O+2e^−^+2H^+^ → N_2_+H_2_O. Probably, it is the most unknown denitrification enzyme in haloarchaea. So far, only *in vivo* experiments have been performed to test its functionality in the presence of oxygen or low pH (Torregrosa-Crespo et al., 2020), but its structure remains unknown. *A priori*, based on the gene sequence, it must be a typical nitrous oxide reductase containing two multicopper sites: a binuclear CuA electron- transferring center and a tetranuclear copper sulfide catalytic center, named the “CuZ center” (Pauleta et al., 2017). As well as for NirK, its electron donor remains unknown in haloarchaea.

Some evidence suggest that these enzymes are located between the cell membrane and the outer S-layer, in the so-called pseudo-periplasm of the Archaea: (i) the characterization of the membrane bound-nitrate reductase in *H. mediterranei* showing its active site located on the positive side of the cell membrane, facing the pseudo-periplasm (Martínez-Espinosa et al., 2007); (ii) the genes coding for nitrite reductase and nitrous oxide reductase contain Tat sequences to direct export the codified proteins outside the membrane (Torregrosa-Crespo et al., 2016); (iii) the nitric oxide reductase has been identified as integral protein of the membrane (Torregrosa-Crespo et al., 2017). By contrast to the well-characterized Bacterial denitrification systems, it is not clear how the pseudo-periplasm N-reductases are coupled to energy conserving electron transfer in Haloarchaea. For example, in Bacteria the NarG and NarH core subunits are anchored to the cytoplasmic (membrane potential positive) side of the cytoplasmic membrane by a di-*b*-haem quinol oxidizing subunit NarI. Electron transport from quinol through NarI to NarGH is coupled to membrane potential generation. However, there is no homolog of NarI in *H. mediterranei* and NarGH are located on the membrane potential positive side of the membrane in the pseudo-periplasm. A novel mechanism of moving electrons from quinol to NarGH is therefore required. A model by which this may occur through a number of putative proteins encoded by genes that cluster close to *narGH* has been proposed (Martínez-Espinosa et al., 2007). These include: a putative di-b-haem protein NarC, that is not a homolog of the Bacterial NarI, but could serve a role as an energy conserving quinol dehydrogenase; a putative pseudo-periplasmic Rieske-type [2Fe-2S] cluster protein NarB and a pseudo-periplasmic facing mono b-haem protein Orf7 (Martínez-Espinosa et al., 2007). No experimental evidence yet exists to any association of the proteins with NarGH.

To date, much of the experimental work on denitrification in Haloarchaea has been focused on physiological studies or, more recently, transcriptional analysis (Torregrosa-Crespo et al., 2019, 2020). These approaches cannot get a global perspective on protein associated with denitrification. Moreover, apart from nitrate reductase (Lledó et al., 2004), none of denitrification enzymes or any of the accessory elements of the electron transport chain have been purified or characterized in native form. Therefore, to have a complete view, a fractionation approach coupled with proteomic analyses was carried out to identify putative components of the denitrification machinery in *H. mediterranei* at the protein level. It was based on an integrated approach consisting on the production of lipid micelles encapsulating the proteins of interest for their subsequent enrichment and identification. This avoids any artificial interactions between them that are common in approaches such as those based on cross-linking and allows the monitoring of enzymatic activities in environments that mimetic the lipid bilayer. The results reveal new insights in wider accessory electron transfer proteins possibly involved in denitrification, notably NarC as a possible component of a NarCGH quinol oxidizing nitrate reductase complex.

## Materials and Methods

### Haloarchaeal Strain, Media, and Growth Conditions

*Haloferax mediterranei* (R-4) was grown in optimal media: 20% (w/v) mixture of salts (20% SW) (Rodríguez-Valera et al., 1980; Lledó et al., 2004) and 0.5% (w/v) yeast extract. The pH was adjusted with NaOH to 7.3.

Pre-inocula were raised aerobically at 42°C and 200 rpm in 250 mL Erlenmeyers containing 50 mL medium. Upon reaching OD_600_ ~ 0.3, these cells served as inoculum [1% (w/v)] to the aerobic cultures: a total of 12 3L-Erlenmeyers containing 600 mL medium each, grown under the same conditions as the pre-inocula. When they reached the mid-stationary phase of growth (OD_600_ ~ 1), anaerobic conditions were applied by transferring the cultures to 10 L sterilized bottle, adding 1 L KNO_3_ (0.5 M) to a final concentration of 50 mM and 2 L fresh medium, thus avoiding overhead space. Cultures were then incubated at 42°C without shaking for 6 days.

### Insoluble Fraction Isolation, Solubilization, and Obtention of Crude Extract

Cells were harvested by centrifugation at 16,300 × g for 45 min at 4°C in a Beckman J-20 XP centrifuge, washed with 20% SW for three times and then centrifuged again at 16,300 × g for 60 min at 4°C. The cell pellet obtained was resuspended in 10 mM Tris-HCl, 1 mM DTT buffer pH 8.0 (40 % w/v for cellular resuspension in the mentioned buffer). After that, cells were disrupted by sonication and the suspension was centrifuged at 2,100 × g for 30 min at 4°C in a Beckman J-20 XP to remove non-lysed cells. The supernatant (containing lysed cells) was centrifuged at 105,000 × g for 60 min at 4°C in a Beckman Coulter Optima™ XL-100K Ultracentrifuge. The new supernatant was discarded and the pellet (containing the insoluble fraction) was then subjected to solubilisation using three different detergents in a range of concentrations [0.5–20% (w/v)]: Triton X-100, n-octyl-β-D-glucopyranoside and CHAPS (provided by Sigma or Anatrace companies). For each of the assays, they were added to the buffer used for the isolation of the crude extract (10 mM Tris-HCl, 1 mM DTT, pH 8), gently stirred at 4°C overnight. Then, the solubilized samples were centrifuged at 105,000 × g for 60 min at 4°C in a Beckman Coulter Optima™ XL-100K Ultracentrifuge. Supernatants were collected to quantify protein by Bradford method and to measure nitrate reductase activity (see below for details). From these experiments the detergent showing the best results (Triton X-100) was selected to get the micelles at large scale for protein purification and proteomic analysis.

### Enrichment of Micelles Involving Proteins of Denitrification

The micelles obtained by solubilizing the membrane were then enriched in denitrification proteins following the next purification steps. All of them were carried out at 25°C:

Step 1: DEAE-Sepharose CL-6B chromatography. The extract obtained (132 mL ± 2) was applied to a DEAE-Sepharose CL-6B column (2.5 × 10 cm), which had previously been equilibrated with 10 mM Tris-HCl, 1 mM DTT, Triton X-100 (0.2%) (pH 8.0). The column was washed with 250 mL of 10 mM Tris-HCl, 1 mM DTT, Triton X-100 (0.2%) (pH 8.0) containing 200 mM NaCl. Elution was carried out with an increasing linear gradient (500 mL) of 200 mM to 2 M NaCl in 10 mM Tris-HCl, 1 mM DTT, Triton X-100 (0.2%) buffer (pH 8.0), at a flow rate of 30 mL/h. Fractions containing Nar and Nir activity were pooled and dialyzed against 10 mM Tris-HCl, 1 mM DTT, Triton X-100 (0.2%) (pH 8.0). The resulting solution was applied to a HiPrep™ Q-Sepharose 16/10 FF column.

Step 2: HiPrep™ Q-Sepharose 16/10 FF. A HiPrep™ Q-Sepharose 16/10 FF column (1.6 x 10 cm) was equilibrated with 10 mM Tris-HCl, 1 mM DTT, Triton X-100 (0.2%) (pH 8.0). The column was washed using the same buffer, at a flow rate of 0.8 ml/h (100 mL). The final pool of micelles was eluted using an increasing linear gradient (100 mL) of 0 to 2 M NaCl in 10 mM Tris-HCl, 1 mM DTT, Triton X-100 (0.2%) (pH 8.0).

### Enzymatic Activity Assays and Protein Quantification

The protein content was determined by the Bradford method, with bovine serum albumin as a standard (Bradford, 1976). Nar and Nir activities were assayed in solubilized extracts as well as along the purification process as previously described (Martínez-Espinosa et al., 2001a,b; Lledó et al., 2004). The assay mixture for Nar activity contained, in a final volume of 250 μL, 100 mM Tris-HCl pH 8, 3.6 M NaCl, 4 mM methyl viologen (MV), 35 mM KNO_3_, 17 mM Na_2_S_2_O_4_ (freshly prepared in 0.1 M NaHCO_3_) and 50 μL of sample preparations; for Nir activity, the mixture contained, in a final volume of 250 μL, 50 mM phosphate buffer pH 7.5, 3.2 M NaCl, 3 mM methyl viologen (MV), 5 mM KNO_2_, 17 mM Na_2_S_2_O_4_ (freshly prepared in 0.1 M NaHCO_3_) and 50 μL of sample preparations The assay was developed at 40°C for 15 min (Nar activity) or 20 min (Nir activity). Nar specific activity is expressed as micromoles of ${\text{NO}}_{2}^{-}$ appearing per minute per milligram of protein, while Nir specific activity is expressed as micromoles of ${\text{NO}}_{2}^{-}$ disappearing per minute per milligram of protein (U = μmol ${\text{NO}}_{2}^{-}$/min). All the assays were carried out in triplicate and against a control assay without enzyme.

### Proteomic Analysis

#### Sample Preparation

Fifty microgram protein from two biological replicates were precipitated with Trichloroacetic acid 10% (w/v) in a final volume of 500 μL overnight at −20°C. Then, the proteins were pelleted by centrifugation at 16,000 × g in a centrifuge Eppendorf 5418 R for 5 min and washed three times using 1 mL of cold acetone for each sample. The clean precipitate was then resuspended in a solution containing urea 6 M (50 μL).

#### Tryptic In-solution Digestion

For tryptic in-solution digestion, 50 μg of protein sample were reduced with 5 μL of 0.2 M DTT followed by incubation for 1 h at 37°C and S-alkylation with 20 μL of 0.2 M iodoacetamide followed by incubation for 1 h in the dark at room temperature. Then, 25 mM ammonium bicarbonate buffer was added to reduce the concentration of urea to 0.6 M. For in-solution digestion, trypsin was added to the protein mixture at an enzyme-to-substrate ratio of 1:30 (w/w). After incubation at 37°C for 16 h, additional trypsin (1:60, w/w) was added to the sample and incubation was continued for 5 h to ensure complete digestion. Tryptic peptides were dried down in a Speed-Vacbenchtop centrifuge and resuspended in 5% acetonitrile and 0.5% trifluoroacetic acid. The resulting peptides were desalted with PepClean C-18 Spin Columns (Agilent Technologies) in batches of 30 μg of protein according to manufacturer recommendations. Eventually, eluted peptides were dried down in a Speed-Vacbenchtop centrifuge and resuspended in 10 μL of first LC mobile phase (5% acetonitrile and 0.1% formic acid).

#### LC-MS/MS Conditions

Peptide separation was performed using an Agilent 1290 Infinity LC system coupled to the 6550 Accurate-Mass QTOF (Agilent Technologies, Santa Clara, CA, USA) with electrospray interface (Jet Stream Technology) operating in positive-ion mode (3,500 V) and in high sensitivity mode. The best conditions for the electrospray interface were: gas temperature 250°C, drying gas 14 L/min, nebulizer 35 psi, sheath gas temperature 250°C, sheath gas flow 11 L/min. Samples were injected (10 μL) on an Agilent Advance Bio Peptide mapping column (2.1 × 250 mm, 2.7 μm) (Agilent Technologies) with a 3–40% gradient of solvent B (0.1% formic acid in 90% acetonitrile) for 140 min operating at 50°C and a flow rate of 0.4 mL/min. The data were acquired with Agilent Mass Hunter Workstation Software, LC/MS Data Acquisition B.08.00 (Build 8.00.8058.0) operating in Auto MS/MS mode whereby the 20 most intense ions (charge states, 2–5) within 300–1,700 m/z mass range above a threshold of 1,000 counts were selected for MS/MS analysis. MS/MS spectra (50–1,700 m/z) were collected with the quadrupole set to “narrow” resolution and were acquired until 25,000 total counts were collected or for a maximum accumulation time of 333 ms. To ensure the desired mass accuracy of recorded ions, continuous internal calibration was performed during analyses with the use of signals m/z 322.0481 (detected m/z [C6H18N3O6P3-H]^+^) and m/z 1221.9906 (detected m/z [C24H18O6N3P3F36-H]^+^).

#### Data Analysis

Each MS/MS spectra data set was processed to determine monoisotopic masses and charge states, to merge MS/MS spectra with the same precursor (Δm/z < 1.4 Da and chromatographic Δt < 60 s) and to select high quality spectra with the Extraction tool of SpectrumMill Proteomics Workbench (Agilent). The reduced data set was searched against the *Haloferax* NCBInr database in the identity mode with the MS/MS search tool of SpectrumMillProteomics Workbench and with the following settings: trypsin, up to 2 missed cleavages, carbamidomethylation of cysteines as fixed modifications, oxidation of methionine as variable, mass tolerance of ±20 ppm for precursor and ±50 ppm for product ions. The precursor mass shift was set between −18 to 177 Da to take into consideration variable modifications such as the presence of sodium and potassium adducts. Peptide hits were validated in the peptide mode to achieve a false discovery rate (FDR) of <1.2% and then in the protein mode according to the score settings recommended by the manufacturer. Positive identifications were considered only when two or more peptides were matched, and their summed score was >30.

## Results and Discussion

### Membrane Solubilisation

Membrane solubilisation is a critical step in any *in vitro* analysis of proteins related to membrane as the aim is to maximally disrupt the lipid components while loading the proteins in an un-natural environment without perturbing them (Duquesne and Sturgis, 2010). Detergents interact with proteins and membranes as micelles, consequently, the solubilisation of this kind of proteins is dependent upon their formation, usually spherical shaped in solution. Several parameters must be considered when choosing detergents: critical micelle concentration (CMC), effects of hydrophilic or hydrophobic groups on CMC, effects of electrolytes on CMC (NaCl for instance) and cloud point and aggregation numbers (the number of detergent monomers present within a micelle) (Rosen, 2004).

Thus, three detergents were chosen to solubilize the membrane extracts based on their physicochemical properties (non-ionic detergents) and their relatively low cost: Triton X-100, n-octyl-β-D-glucopyranoside and CHAPS. They were assayed at different concentrations between 0.5 and 20% (w/v) to determine the optimal one to get the highest protein concentration, while showing the highest nitrate reductase activity. This was selected as target enzymatic activity to monitor the stability of the biological function of the enzymes within the micelles: it is a highly sensitive method, low cost and low time consuming compared to those usually used to measure nitrite, nitric oxide or nitrous oxide reductases activities. Table 1 displays the best results from each detergent.

**Table 1:** Parameters of the different detergents tested for the formation of micelles from crude extract: CMC (critical micelle concentration); % detergent (w/v); protein concentration (mg/mL); nitrate reductase specific activity (U/mg).

	**Detergent**		
	**Triton X-100**	**Chaps**	**n-octyl-β-D-**
			**glucopyranoside**
CMC	0.2–0.9 mM	6–10 mM	18–20 mM
% Detergent (w/v)	1 (16 mM)	1.2 (20 mM)	1 (34 mM)
Protein concentration (mg/mL)	16.5 ± 0.8	9.3 ± 0.5	10.5 ± 0.2
Nar activity (U/mg protein)	0.18 ± 0.03	0.12 ±0.02	0.20 ± 0.02

Detergents traditionally used for protein solubilisation are Triton X-100 (non-ionic) and CHAPS (zwitterionic), used alone or in combination with urea or urea-thiourea (Luche et al., 2003). Among them, CHAPS is highly chaotropic thus disrupting typical protein interactions within protein complexes. Apart from them, in the last years, the most efficient non-ionic detergents reported belongs to the glycoside family (e.g., octyl glucoside, dodecylmaltoside) (Witzmann et al., 1991; Taylor and Pfeiffer, 2003). In this study, the membrane solubilized sample showed the highest Nar activity when using n-octyl-β-D-glucopyranoside, followed by Triton X-100 and CHAPS; protein concentration was higher in Triton X-100 extracts followed by n-octyl-β-D-glucopyranoside and CHAPS. From these results, it can be concluded that both Triton X-100 and n-octyl-β-D-glucopyranoside were good enough to reach high ratio of solubilisation avoiding the loss of enzymatic activity. Considering that Nar activities were similar in both samples, but protein concentration was slightly higher in Triton X-100 samples, it was chosen as detergent to carry out protein enrichment and proteomic analysis. Triton X-100 concentrations around 2% (w/v) were optimal for the solubilisation of the membranes before proceeding with protein enrichment.

### The Enrichment of the Molecular Machinery of Denitrification in *H. mediterranei*: From Crude Extract to Micelles

#### Denitrification Enzymes

**Figure 1:**
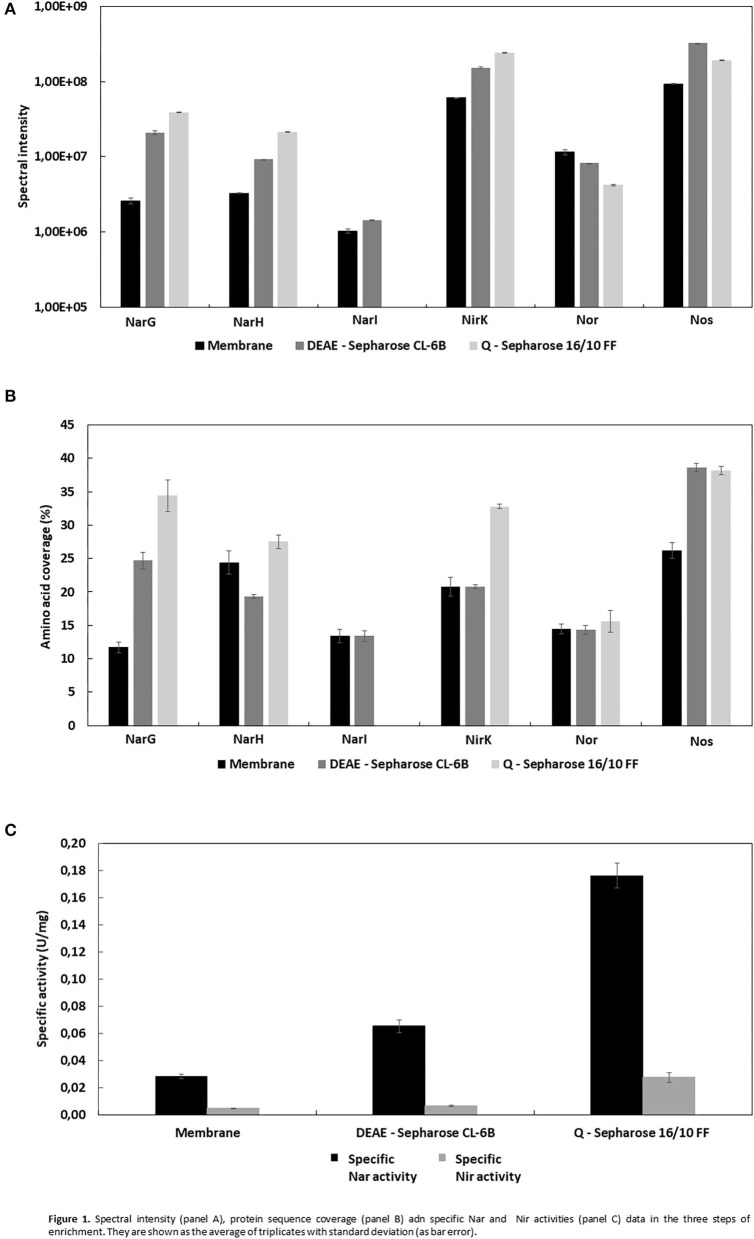
Spectral intensity **(A)**, protein sequence coverage **(B)** adn specific Nar and Nir activities **(C)** data in the three steps of enrichment. They are shown as the average of triplicates with standard deviation (as bar error).

**Figure 2:**
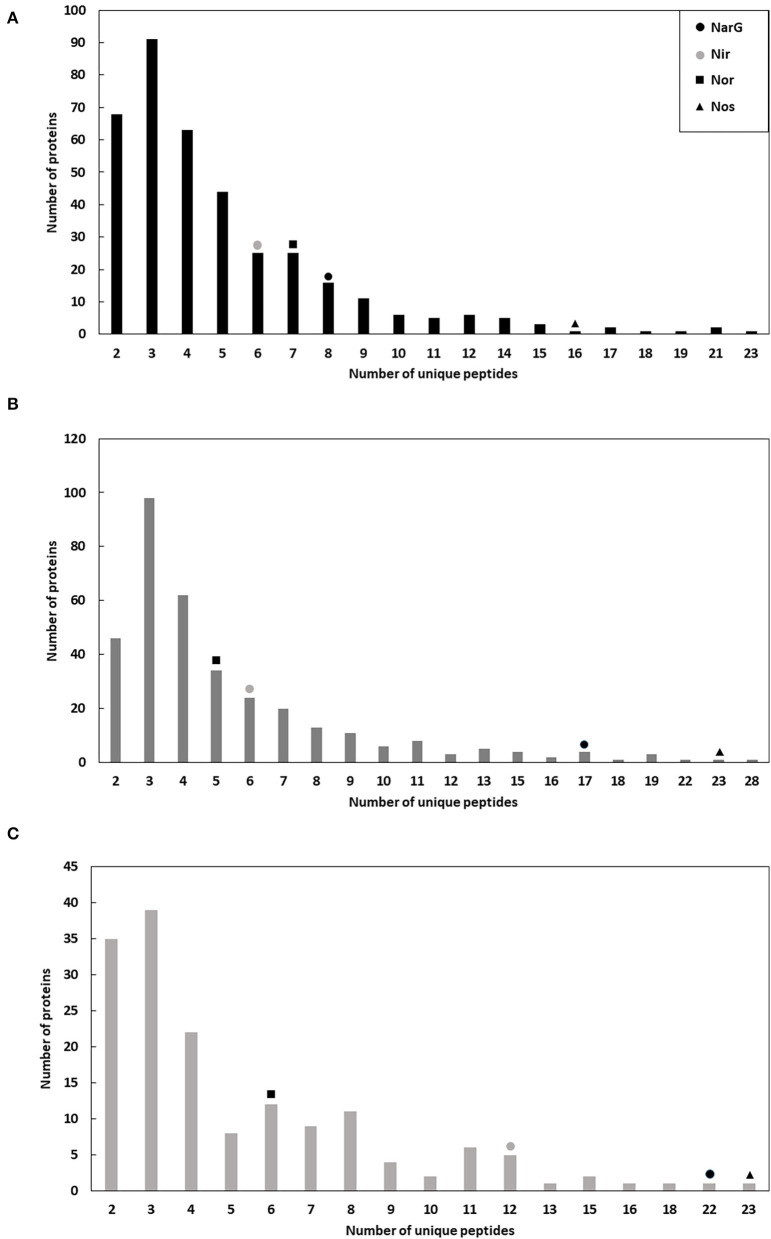
Number of proteins with different and unique peptides in the micelles during the enrichment process: crude extract **(A)**, DEAE-Sepharose **(B)**, and Q-Sepharose **(C)**. Positive identifications are considered only when two or more peptides are matched, and their summed score is >30.

The different sets of micelles obtained from crude extracts were used for a protein purification process involving two chromatographic steps in order to enrich the samples for denitrification proteins. Thus, from each chromatographic step elution samples were analyzed by liquid chromatography coupled to mass spectrometry (LC/MS-MS) (Supplementary Tables 1–4). In respect of the enzymes of denitrification, the protocol undertaken resulted in enrichment of the micelles in terms of Mascot score, protein sequence coverage, spectral intensity and the Nar and Nir specific activities (Figure 1, Tables 2, 3). The enrichment process resulted in the reduction of the total number of proteins in the micelles by ~43%: from 375 proteins in the crude extract to 162 obtained after HiPrep™ Q-Sepharose 16/10 FF. Likewise, the number of different peptides counted for the identification of denitrification enzymes increased considerably: NarG and NosZ were the proteins with the highest number of different peptides identified from the whole protein set (22 and 23 peptides, respectively) (Figure 2). NirK peptides also increased, from 6 to 12, this enzyme also being one of the 10 with the highest number of different peptides identified. In case of Nor, the values remained practically constant throughout the enrichment process (from 7 to 6), which was in line with the protein sequence coverage, score and spectral intensity data (Figure 2).

**Table 2:** Score, protein sequence coverage, and spectral intensity values of the N-reductases during the enrichment process.

	**NarG**			**NarH**			**Orf7**		
**Fraction**	**Score**	**%aa coverage**	**Spectral intensity**	**Score**	**%aa coverage**	**Spectral intensity**	**Score**	**%aa coverage**	**Spectral intensity**
**A**									
Crude extract	119.17 ± 19.8	11.70 ± 0.8	2.58E6 ± 2.5E5	112.14 ± 4.7	24.40 ± 1.7	3.27E6 ± 2.7E4	49.88 ± 1.5	13.40 ± 1.1	1.03E6 ± 7.5E4
DEAE-Sepharose	269.39 ± 9.1	24.70 ± 1.2	2.09E7 ± 1.2E6	95.18 ± 1.7	19.30 ± 0.3	9.26E6 ± 2.3E4	47.03 ± 2.4	13.40 ± 0.8	1.43E6 ± 2.7E4
Q-Sepharose	380.05 ± 11.3	34.40 ± 2.3	3.88E7 ± 2.9E5	136.08 ± 2.7	27.50 ± 1.0	2.12E7 ± 3.1E5	n.a.	n.a.	n.a.
	**Nir**			**Nor**			**Nos**		
**Fraction**	**Score**	**%aa coverage**	**Spectral intensity**	**Score**	**%aa coverage**	**Spectral intensity**	**Score**	**%aa coverage**	**Spectral intensity**
**B**									
Crude extract	108.21 ± 1.7	20.80 ± 1.4	6.04E7 ± 7.4E5	126.21 ± 9.3	14.53 ± 0.8	1.15E7 ± 8.5E5	263.67 ± 14.8	26.2 ± 1.2	9.36E7 ± 2.1E5
DEAE-Sepharose	112.23 ± 8.0	20.80 ± 0.3	1.53E8 ± 3.2E6	99.58 ± 2.5	14.3 ± 0.7	8.25E6 ± 2.7E4	381.40 ± 3.1	38.6 ± 0.6	3.24E8 ± 1.5E6
Q-Sepharose	161.07 ± 4.9	32.80 ± 0.3	2.40E8 ± 4.0E6	106.03 ± 7.0	15.6 ± 1.7	4.20E6 ± 7.0E4	349.62 ± 5.3	38.2 ± 0.6	1.90E8 ± 2.1E6

*Data are shown as the average of triplicates with standard deviation. (A) Results related to subunits involved on respiratory nitrate reductase complex. (B) Results related to the other three structural enzymes of denitrification in Haloferax mediterranei*.

**Table 3:** Purification parameters of Nar (A) and Nir (B) during the enrichment of micelles.

	**Nar**						
**Fraction**	**Volume** ** (mL)**	**Protein concentration** ** (mg/mL)**	**Total protein** ** (mg)**	**Total activity** ** (U)**	**Specific activity** ** (U/mg)**	**Purification** ** (Fold)**	**Yield** ** (%)**
**A**							
Crude extract	132 ± 2.0	5.64 ± 0.3	747.06 ± 49	20.86 ± 0.3	0.028 ± 0.002	1.00	100
DEAE-Sepharose	75 ± 1.7	1.70 ± 0.1	127.38 ± 4.9	8.29 ± 0.3	0.065 ± 0.005	2.33 ± 0.2	39.77 ± 1.8
Q-Sepharose	11.70 ± 0.7	1.41 ± 0.05	16.47 ± 0.6	2.90 ± 0.21	0.176 ± 0.009	6.32 ± 0.67	13.92 ± 0.8
	**Nir**						
**Fraction**	**Volume** ** (mL)**	**Protein concentration** ** (mg/mL)**	**Total protein** ** (mg)**	**Total activity** ** (U)**	**Specific activity** ** (U/mg)**	**Purification** ** (Fold)**	**Yield** ** (%)**
**B**							
Crude extract	132 ± 2.0	5.64 ± 0.3	747.06 ± 49	3.46 ± 0.15	0.005 ± 0.000	1.00	100
DEAE-Sepharose	75 ± 1.7	1.70 ± 0.1	127.38 ± 5.0	0.83 ± 0.07	0.007 ± 0.001	1.41 ± 0.07	24.09 ± 2.5
Q-Sepharose	11.70 ± 0.8	1.41 ± 0.05	16.47 ± 0.6	0.45 ± 0.05	0.028 ± 0.004	5.97 ± 1.0	13.10 ± 1.4

*Values are shown as the average of triplicates with standard deviation*.

Dealing first with the catalytic subunit of nitrate reductase (NarG), this the most enriched protein in the micelles with respect to the crude extract: the Mascot score and protein sequence coverage improved by ~219 and ~194%, respectively, and the spectral intensity increased from 2.58E6 ± 2.5E5 to 3.88E7 ± 2.9E5 (Table 2A; Figures 1A,B). This was in line with Nar specific activity increment, from 0.028 ± 0.002 to 0.176 ± 0.009 U/mg (Table 3A; Figure 1C). The values obtained for NarG in terms of specific activity, purification (fold) and yield were remarkably similar to those obtained in the penultimate step of the purification of Nar in *H. mediterranei* (Lledó et al., 2004). The subunit NarH, involved in the electron transfer to NarG, also displayed an improved Mascot score and protein sequence coverage (~21 and ~13%, respectively) (Table 2A). The spectral intensity also increased by an order of magnitude (from 3.27E6 ± 2.7E4 to 2.12E7 ± 3.1E5) (Table 2A; Figures 1A,B).

For the nitrite reductase, NirK, both the Mascot score and the protein sequence coverage improved by ~49 and 58%, respectively (Table 2B; Figure 1B). The spectral intensity was the highest of all the enzymes for the last enrichment step, reaching 2.40E8 ± 4.0E6 (Table 2B; Figure 1A). Despite this, the specific activity registered in the micelles was low compared to Nar activity (0.028 ± 0.004 U/mg), although 5.5 times higher than the obtained in the crude extract (0.005 U/mg) (Table 3B). Testing different strategies of enrichment, nitrite reductase activity was always difficult to measure. In fact, when trying to apply a third chromatography, based on size exclusion (SuperoseTM 6 Increase 10/300 GL—GE Healthcare), nitrite reductase activity could not be measured, although the enzyme was detected. This NirK is an oxygen sensitive copper containing enzyme and so the loss of activity could be due to a combination of two factors, loss of copper or exposure during the purification process as previously described in the literature (Sánchez et al., 2010; Felgate et al., 2012), but this was not investigated further in this study.

The nitric oxide reductase was the only one of the four enzymes of denitrification that did not appear to enrich in the micelles using this protocol: the Mascot score and protein sequence coverage remained fairly constant, from 126.21 ± 9.3 to 106.03 ± 7.0 and from 14.53 ± 0.8 to 15.6 ± 1.7, respectively, while the spectral intensity decreased until 4.20E6 ± 7.0E4 (Table 2B; Figure 1B). In *H. mediterranei*, qNor is a protein with 14 transmembrane segments, which could lead to low detection by LC-MS/MS (Supplementary Figure 1). For example, the lipids of the membrane or the micelle could prevent the access of trypsin to the transmembrane regions, which would adversely affect their digestion and subsequent detection. However, the protocol applied in this study did enable the detection of 6/7 peptides, so it does not appear that this was a problem in this case.

Finally, nitrous oxide reductase also showed improved Mascot score values (~33%) and protein sequence coverage (~45%) (Table 2B; Figure 1B). After the last step of enrichment, the spectral intensity reached 1.90E8 ± 2.1E6, the second highest after nitrite reductase (Table 2B; Figure 1A). In fact, these two enzymes had the best spectral intensity values from the beginning. The data are in line with those obtained when trying to isolate the crude extract using a lower percentage of Triton X-100 (10% w/v). In that case, none of the denitrification enzymes were identified, except for nitrite and nitrous oxide reductase (Supplementary Table 1). The predominance of these two proteins in the micelles could indicate that they are more highly synthesized than the other catalytic subunits. This is consistent with a recent study showing that the highest peaks of expression during denitrification were reached by the genes that encode them (nirK and nosZ) (Torregrosa-Crespo et al., 2020). Despite these observations, this hypothesis should be addressed using quantitative proteomics.

### Accessory Proteins for N-Reductases

Denitrification enzymes combined with a variety of electrons donors to enable the electron flow from intracellular electron donors to the different N-substrates. With the proteomic approach here described, it was possible to identify the potential electron donors, some of them unknown or only annotated at gene level to date in *Haloferax* genomes. For example, several subunits closely related to proton-translocating enzymes, like NADH dehydrogenase, as well as several enzymes involved in ubiquinone/menaquinone biosynthesis could be identified (Supplementary Table 4). Electrogenic Q-reducing enzymes like formate dehydrogenases or hydrogenases could also generate proton motive force (Jormakka et al., 2002). However, these enzymes have not been identified through proteomics in this approach. ATP synthases are also required in this system: most of the subunits of the rotary A-type ATP synthase have been identified in this study (Supplementary Table 4). This was in line with the general postulate that the predominant ATP synthases in Archaea are those of type A (Grüber et al., 2014).

As previously described, during respiratory electron transfer, Nar enzymes receive electrons from quinols located within the lipid phase of the cytoplasmic membrane (Martínez-Espinosa et al., 2007). Nar from *H. mediterranei* was described as pNar, i.e., the active site is facing the positive site of the membrane. This orientation brings the question of whether being located at the positive face of the membrane, pNarG could be a poorly coupled enzyme, like Bacterial periplasmic NapA, or whether there is a mechanism by which pNarG can maintain bioenergetic equivalence with its nNarG bacterial counterpart (Martínez-Espinosa et al., 2007). At present, it is not known how electrons move from the Q-pool to pNarG in haloarchaea. Through bioinformatics analysis, we previously raised the possibility of a role for three putative proteins in electron transfer from quinol to NarGH that were encoded by genes that clustered with narGH: Orf7, a monotopic di-b-heme protein; NarC, di-b-heme protein comprising nine transmembrane helices; and NarB, a Rieske iron-sulfur protein with the redox-active domain facing the pseudo-periplasm (Martínez-Espinosa et al., 2007). In support of a role in electron transfer to NarGH, NarC enriched through all 3 steps of the purification procedure. By contrast NarB and Orf7 did not, with neither being dectable after the 3rd step (Table 4A). Tacking as a reference the Q-cycle coupling mechanism for the pNar enzyme of *H. mediterranei* proposed in 2007 (Martínez-Espinosa et al., 2007), it is possible to infer that the main core for respiratory nitrate reductase in haloarchaea could be NarGHC.

Table 4Score, percentage coverage, and spectral intensity values of different accessory proteins of denitrification enzymes during the enrichment process.

**Table:** 

	**NarB**			**NarC**		
**Fraction**	**Score**	**%aa coverage**	**Spectral intensity**	**Score**	**%aa coverage**	**Spectral intensity**
**A**						
Crude extract	46.02 ± 1.0	28.60 ± 0.8	9.83E5 ± 3.06E3	30.00 ± 1.3	3.90 ± 0.4	1.36E6 ± 1.73E3
DEAE-Sepharose	n.a.	n.a.	n.a.	40.50 ± 2.1	10.90 ± 0.6	9.75E6 ± 3.46E4
Q-Sepharose	n.a.	n.a.	n.a.	30.23 ± 1.2	8.60 ± 0.5	7.00E5 ± 1.53E3

**Table:** 

	**Nir-Copper containing**			**Halocyanin**			**HFX_2180**		
**Fraction**	**Score**	**%aa coverage**	**Spectral intensity**	**Score**	**%aa coverage**	**Spectral intensity**	**Score**	**%aa coverage**	**Spectral intensity**
**B**									
Crude extract	80.16 ± 3.8	17.30 ± 1.1	4.77E6 ± 2.08E4	88.97 ± 1.9	26.50 ± 1.3	1.52E7 ± 1.53E5	40.21 ± 1.9	8.70 ± 0.7	3.36E6 ± 2.08E4
DEAE-Sepharose	123.51 ± 6.2	25.10 ± 2.5	3.91E7 ± 6.66E5	107.3 ± 1.9	35.70 ± 1.3	3.41E7 ± 2.08E5	51.09 ± 1.8	8.70 ± 0.4	2.99E6 ± 4.90E5
Q-Sepharose	182.42 ± 8.7	46.30 ± 2.0	5.32E7 ± 2.08E5	136.09 ± 1.3	42.30 ± 1.1	1.16E7 ± 1.00E5	30.95 ± 3.4	3.50 ± 0.6	8.84E5 ± 4.16E3

**Table:** 

	**Copper-binding plastocyanin**		
**Fraction**	**Score**	**%aa coverage**	**Spectral intensity**
**C**			
Crude extract	34.00 ± 0.9	20.80 ± 1.0	1.90E6 ± 1.00E4
DEAE-Sepharose	61.54 ± 2.2	36.90 ± 1.0	1.08E7 ± 2.08E5
Q-Sepharose	59.55 ± 4.7	36.90 ± 2.8	1.25E7 ± 3.21E5

*Data are shown as the average of triplicates with standard deviation. (A) proteins related to Nar; (B): proteins related to Nir and Nor; (C) proteins related to Nos. n.a., not applicable*.

Different proteins that could act as electron donors located in the *nir-nor* cluster were also sought. One candidate is a putative halocyanin protein encoded by a gene annotated as *hcy*. Halocyanins are small blue copper membrane associated proteins from halophilic archaea that are thought to act as mobile electron carriers similarly to plant plastocyanins with a molecular mass of about 15 kDa (Scharf and Engelhard, 1993; Torregrosa-Crespo et al., 2016). During the enrichment process, the score and protein sequence coverage showed an increment near 50 and 60%, similar to Nir data (Table 4B). The knowledge about halocyanins is scarce, one belonging to *Natronomonas pharaonis* being the only one purified to date (Mattar et al., 1994), but the current work suggests that it might be acting in the electron transfer during nitrite and/or nitric oxide reduction (Brischwein et al., 1993; Mattar et al., 1994).

Another putative protein encoded within the *nir-nor* cluster is annotated in the database as “nitrite reductase-copper containing.” Although its name in the database suggests that it could be a second copy of Nir, the alignment of its nucleotide sequences did not show any significant result, while their amino acid sequences had a very low similarity (23.30%) (Supplementary Figure 2). It seems more likely that it is a copper oxidoreductase, which could be involved in the electron transfer during nitrite/nitric oxide reduction (like halocyanin). For this protein, the Mascot score and protein sequence coverage improved by ~128 and ~168%, respectively, during enrichment (Table 4B). These values are higher than those for the nitrite reductase. In addition, the spectral intensity reached 5.32E7 ± 2.1E5, higher than for some terminal denitrification enzymes such as Nar and Nor (Table 4B).

During the enrichment process, a hypothetical protein (named HFX_2180) of 440 amino acids whose gene is located close to *nirK* was also identified (Table 4B). Although previously its gene had not been annotated as part of the *nir-nor* cluster, it could be a candidate as an electron donor, but as it has a conserved COX-type domain, belonging to heme-Cu-oxidase I Superfamily it is possible that it is involved in oxygen scavenging.

Finally, in case of nitrous oxide reductase, the plastocyanin encoded by the gene located immediately upstream of *nosZ* (*pcy*) was identified. The results from the Mascot score and protein sequence coverage for this protein improved around 75% and the spectral intensity reached 1.25E7 (Table 4C). Being a small blue copper protein, it could act as potential electron donor to Nos. However, there are some indications that suggest that *pcy* is incorrectly annotated, and this protein was actually an azurin: (i) plastocyanins are involved in electron transfer processes in the plant chloroplasts or in cyanobacteria, never found in non-photosynthetic organisms (Pérez-Henarejos et al., 2015); (ii) the number of amino acids contained in this protein (170) is very high for a plastocyanin, which usually does not reach a hundred, while azurins from different species contain between 120 and 130 amino acids (Pérez-Henarejos et al., 2015). Likewise, azurins have been identified in different denitrifying bacteria such as *Pseudomonas* or *Alcaligenes* (Machczynski et al., 2002; Dell'acqua et al., 2011), with their function being closely related to denitrification.

## Conclusions

To our knowledge, proteomics has never been used as a tool for the study of denitrification in haloarchaea. In this group of microorganisms, this approach has been used to make comparative studies between mid-log and late-log phases in *Haloarcula marismortui* (Chu et al., 2011), *Haloferax volcanii*, or *Natrialba magadii* (Cerletti et al., 2018), to compare differences in protein expression under different salt concentrations in *Halobacterium salinarum* (Leuko et al., 2009) or to study the effect of proteases in *Haloferax volcanii* (Costa et al., 2018).

Using proteomics and the generation of different populations of micelles, key proteins involved in denitrification can be identified. This is especially useful in organisms in which knowledge about this respiratory pathway is scarce, such as extremophiles, in which some proteins involved in denitrification remain unknown (such as some electron donors). Thus, the results obtained point the scientific community toward new information at a macro level of denitrification proteins in the haloarchaea, which remain poorly studied compared to their bacterial counterparts. These clues as this the nature of this macro-organization will form the basis of future biochemical studies of our group and others around the work that are interested in haloarchaea. Although there is a bias toward rich populations in nitrate reductases, it was demonstrated that the enrichment of the micelles based on Nar activity was a useful, fast and cheap tool that allows the co-purification of all the N-reductases together with their accessory proteins that may be involved in sustaining the proton motive force and electron flow in native conditions.

**Figure 3:**
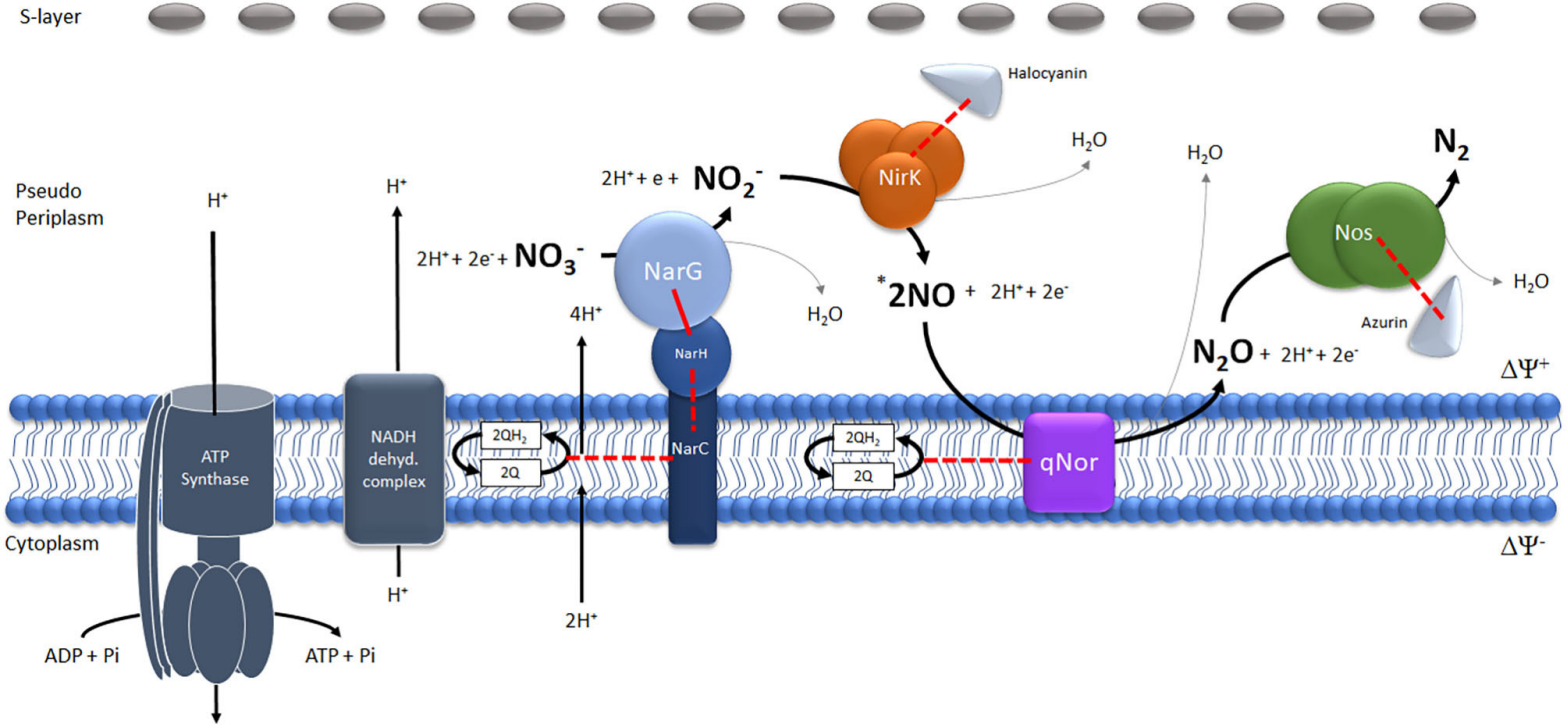
Schematic model of the potential denitrification super-complex in *Haloferax mediterranei*. The different reactions catalyzed by the four enzymes (Nitrate-Nar, Nitrite-Nir, Nitric oxide-Nor and Nitrous oxide- Nos reductases) have been represented stoichiometrically. *The number of NO molecules generated by the reduction of one molecule of ${\text{NO}}_{2}^{-}$ has been multiplied by two to maintain the stoichiometry of the generation of one molecule of N_2_O.The red lines represent physiologically tested (continuous lines) or potential(discontinuous lines) electron flows.

The results obtained in this work make possible to propose the existence of a denitrification super-complex of proteins located between the cellular membrane and S-layer in haloarchaea (Figure 3). The proposed model forms the basis for testing in future biochemical work considering that qNor and NarC (tightly attached to NarGH) are integral proteins defining the main core of this super-complex.

With this work new questions have been raised related to the molecular machinery for denitrification in haloarchaea, including aspects directly connected to bioenergetics and redox status. In the future, studies based on comparative and quantitative proteomics between different denitrifying conditions should be undertaken in order to better understand this pathway in hypersaline environments.

## Data Availability Statement

The original contributions presented in the study are publicly available. This data can be found in the PRIDE repository, under accession number PXD022473.

## Author Contributions

The design of the experiments were conducted by CP and RM-E. Membranes solubilization, protein purification, and enzymatic activity measurements were performed by JT-C and RM-E. Proteomic analysis was performed by JT-C. Conceptualization, administration, and funding of the project were supplied by DR, CP, and RM-E. All authors contributed to the manuscript writing.

## Conflict of Interest

The authors declare that the research was conducted in the absence of any commercial or financial relationships that could be construed as a potential conflict of interest.
