# Supplementary material for: Exploring the Molecular Machinery of Denitrification in Haloferax mediterranei Through Proteomics
Source: Front Microbiol. 2020 Dec 8;11:605859. doi: 10.3389/fmicb.2020.605859 (PMC7754194; doi:10.3389/fmicb.2020.605859)
Supplement: Supplementary file 1 [file Data_Sheet_1.docx]

Exploring the molecular machinery of denitrification in *Haloferax mediterranei* through proteomics

Javier Torregrosa-Crespo^1^, Carmen Pire^1^, David J. Richardson^2^, Rosa María Martínez-Espinosa^1*^

^1^Departamento de Agroquímica y Bioquímica, División de Bioquímica y Biología Molecular, Facultad de Ciencias, Universidad de Alicante, Carretera San Vicente del Raspeig s/n - 03690 San Vicente del Raspeig, Alicante, Spain

^2^School of Biological Sciences, University of East Anglia, Norwich Research Park, Norwich NR4 7TJ, UK

*** Correspondence:**Corresponding Author: Rosa María Martínez-Espinosa
e-mail: [rosa.martinez@ua.es](mailto:rosa.martinez@ua.es); Telephone: +34 96 590 3400 ext. 1258; 8841; Fax: +34 96 590 3464

Keywords: haloarchaea, denitrification, anaerobiosis, proteomics, electron transfer, *Haloferax*, liquid chromatography-mass spectrometry, detergents, micelles.

Table S1. Representative proteins identified in the crude extract using Triton X-100 (10% w/v) from 3 LC-MS/MS runs.

| **Protein Name** | **Database**  **Accession** | **Spectra** | **Distinct**  **Peptides** | **Distinct**  **Summed**  **MS/MS Search**  **Score** | **% aa**  **Coverage** | **Total Protein Spectral Intensity** |
| --- | --- | --- | --- | --- | --- | --- |
| SPFH domain, Band 7 family protein | [AFK18490.1](http://smserver.sti.ua.es/millhtml/SM_instruct/servadmn.htm#update_acclinks?AFK18490.1) | 32 | 23 | 400.03 | [59.1](http://smserver.sti.ua.es/millbin/msdigest.cgi?missed_cleavages=2&msparams_dir=msparams_mill/&hide_protein_sequence=2&database=PA_haloferax_mediterranei_genbank&seqdb_dir=D:\SeqDB\&enzyme=Trypsin&access_method=Accession+Number&accession_num=AFK18490.1&coverage_map=0+42+9+1+30+6+14+5+37+9+39+3+10+22+17+2+8+5+28+26+60+53) | 2.70e+008 |
| SPFH domain, Band 7 family protein | [AFK17777.1](http://smserver.sti.ua.es/millhtml/SM_instruct/servadmn.htm#update_acclinks?AFK17777.1) | 11 | 9 | 147.90 | [29.1](http://smserver.sti.ua.es/millbin/msdigest.cgi?missed_cleavages=2&msparams_dir=msparams_mill/&hide_protein_sequence=2&database=PA_haloferax_mediterranei_genbank&seqdb_dir=D:\SeqDB\&enzyme=Trypsin&access_method=Accession+Number&accession_num=AFK17777.1&coverage_map=0+89+25+7+28+16+25+10+19+23+10+28+11+114) | 5.11e+007 |
| AAA-type ATPase (transitional ATPase-like protein) | [AFK20074.1](http://smserver.sti.ua.es/millhtml/SM_instruct/servadmn.htm#update_acclinks?AFK20074.1) | 28 | 25 | 391.22 | [46](http://smserver.sti.ua.es/millbin/msdigest.cgi?missed_cleavages=2&msparams_dir=msparams_mill/&hide_protein_sequence=2&database=PA_haloferax_mediterranei_genbank&seqdb_dir=D:\SeqDB\&enzyme=Trypsin&access_method=Accession+Number&accession_num=AFK20074.1&coverage_map=0+22+23+26+10+46+15+39+19+17+19+5+56+11+35+7+21+62+25+4+9+4+12+10+12+5+21+47+36+57+19+19+10+19) | 4.41e+007 |
| A-type ATP synthase subunit A | [AFK18041.1](http://smserver.sti.ua.es/millhtml/SM_instruct/servadmn.htm#update_acclinks?AFK18041.1) | 27 | 17 | 306.52 | [35.6](http://smserver.sti.ua.es/millbin/msdigest.cgi?missed_cleavages=2&msparams_dir=msparams_mill/&hide_protein_sequence=2&database=PA_haloferax_mediterranei_genbank&seqdb_dir=D:\SeqDB\&enzyme=Trypsin&access_method=Accession+Number&accession_num=AFK18041.1&coverage_map=0+10+16+79+8+99+32+24+41+15+15+11+17+47+13+56+15+23+10+7+19+3+23+3) | 1.19e+008 |
| succinate dehydrogenase, subunit A (flavoprotein) | [AFK20495.1](http://smserver.sti.ua.es/millhtml/SM_instruct/servadmn.htm#update_acclinks?AFK20495.1) | 20 | 16 | 293.07 | [35.9](http://smserver.sti.ua.es/millbin/msdigest.cgi?missed_cleavages=2&msparams_dir=msparams_mill/&hide_protein_sequence=2&database=PA_haloferax_mediterranei_genbank&seqdb_dir=D:\SeqDB\&enzyme=Trypsin&access_method=Accession+Number&accession_num=AFK20495.1&coverage_map=1+17+51+16+23+13+26+21+54+60+5+22+73+14+93+19+10+11+2+29+56) | 6.45e+007 |
| dipeptide ABC transporter dipeptide-binding protein | [AFK17806.1](http://smserver.sti.ua.es/millhtml/SM_instruct/servadmn.htm#update_acclinks?AFK17806.1) | 20 | 17 | 281.44 | [27.9](http://smserver.sti.ua.es/millbin/msdigest.cgi?missed_cleavages=2&msparams_dir=msparams_mill/&hide_protein_sequence=2&database=PA_haloferax_mediterranei_genbank&seqdb_dir=D:\SeqDB\&enzyme=Trypsin&access_method=Accession+Number&accession_num=AFK17806.1&coverage_map=0+253+21+22+36+31+17+29+9+5+22+23+12+56+22+24+33) | 1.21e+008 |
| A-type ATP synthase subunit C | [AFK18039.1](http://smserver.sti.ua.es/millhtml/SM_instruct/servadmn.htm#update_acclinks?AFK18039.1) | 22 | 16 | 271.71 | [64.6](http://smserver.sti.ua.es/millbin/msdigest.cgi?missed_cleavages=2&msparams_dir=msparams_mill/&hide_protein_sequence=2&database=PA_haloferax_mediterranei_genbank&seqdb_dir=D:\SeqDB\&enzyme=Trypsin&access_method=Accession+Number&accession_num=AFK18039.1&coverage_map=0+20+11+3+61+15+24+49+17+3+10+10+36+9+50+14+16) | 6.55e+007 |
| dipeptide/oligopeptide/nickel ABC transporter periplasmic substrate-binding protein | [AFK18790.1](http://smserver.sti.ua.es/millhtml/SM_instruct/servadmn.htm#update_acclinks?AFK18790.1) | 19 | 16 | 262.89 | [34.6](http://smserver.sti.ua.es/millbin/msdigest.cgi?missed_cleavages=2&msparams_dir=msparams_mill/&hide_protein_sequence=2&database=PA_haloferax_mediterranei_genbank&seqdb_dir=D:\SeqDB\&enzyme=Trypsin&access_method=Accession+Number&accession_num=AFK18790.1&coverage_map=0+80+44+45+25+38+20+22+15+92+28+41+7+8+14+1+28+41+9+33+11+3+13) | 5.37e+007 |
| A-type ATP synthase subunit B | [AFK18042.1](http://smserver.sti.ua.es/millhtml/SM_instruct/servadmn.htm#update_acclinks?AFK18042.1) | 23 | 15 | 256.40 | [38.4](http://smserver.sti.ua.es/millbin/msdigest.cgi?missed_cleavages=2&msparams_dir=msparams_mill/&hide_protein_sequence=2&database=PA_haloferax_mediterranei_genbank&seqdb_dir=D:\SeqDB\&enzyme=Trypsin&access_method=Accession+Number&accession_num=AFK18042.1&coverage_map=0+69+72+3+20+45+15+5+13+32+16+44+31+29+6+3+7+58) | 1.11e+008 |
| poly(3-hydroxyalkanoate) synthase subunit PhaC (plasmid) | [AFK21054.1](http://smserver.sti.ua.es/millhtml/SM_instruct/servadmn.htm#update_acclinks?AFK21054.1) | 19 | 14 | 253.12 | [41.4](http://smserver.sti.ua.es/millbin/msdigest.cgi?missed_cleavages=2&msparams_dir=msparams_mill/&hide_protein_sequence=2&database=PA_haloferax_mediterranei_genbank&seqdb_dir=D:\SeqDB\&enzyme=Trypsin&access_method=Accession+Number&accession_num=AFK21054.1&coverage_map=1+12+1+11+7+11+71+23+32+20+10+30+3+55+163+43) | 2.41e+007 |
| sugar ABC transporter substrate binding protein | [AFK20386.1](http://smserver.sti.ua.es/millhtml/SM_instruct/servadmn.htm#update_acclinks?AFK20386.1) | 17 | 14 | 247.01 | [40.9](http://smserver.sti.ua.es/millbin/msdigest.cgi?missed_cleavages=2&msparams_dir=msparams_mill/&hide_protein_sequence=2&database=PA_haloferax_mediterranei_genbank&seqdb_dir=D:\SeqDB\&enzyme=Trypsin&access_method=Accession+Number&accession_num=AFK20386.1&coverage_map=0+78+8+22+31+8+44+32+9+33+24+21+15+53+25+2+8+31+45+22) | 1.04e+008 |
| NADH dehydrogenase, subunit CD (ubiquinone) | [AFK18696.1](http://smserver.sti.ua.es/millhtml/SM_instruct/servadmn.htm#update_acclinks?AFK18696.1) | 20 | 15 | 245.61 | [30.5](http://smserver.sti.ua.es/millbin/msdigest.cgi?missed_cleavages=2&msparams_dir=msparams_mill/&hide_protein_sequence=2&database=PA_haloferax_mediterranei_genbank&seqdb_dir=D:\SeqDB\&enzyme=Trypsin&access_method=Accession+Number&accession_num=AFK18696.1&coverage_map=0+20+15+52+29+145+26+30+14+7+12+34+32+30+34+18+8+51) | 7.21e+007 |
| NADH dehydrogenase, subunit D (ubiquinone) | [AFK18684.1](http://smserver.sti.ua.es/millhtml/SM_instruct/servadmn.htm#update_acclinks?AFK18684.1) | 6 | 4 | 71.91 | [7.9](http://smserver.sti.ua.es/millbin/msdigest.cgi?missed_cleavages=2&msparams_dir=msparams_mill/&hide_protein_sequence=2&database=PA_haloferax_mediterranei_genbank&seqdb_dir=D:\SeqDB\&enzyme=Trypsin&access_method=Accession+Number&accession_num=AFK18684.1&coverage_map=0+314+14+7+12+57+9+30+9+102) | 2.88e+007 |
| halocyanin hcpG | [AFK18949.1](http://smserver.sti.ua.es/millhtml/SM_instruct/servadmn.htm#update_acclinks?AFK18949.1) | 17 | 13 | 242.96 | [26.5](http://smserver.sti.ua.es/millbin/msdigest.cgi?missed_cleavages=2&msparams_dir=msparams_mill/&hide_protein_sequence=2&database=PA_haloferax_mediterranei_genbank&seqdb_dir=D:\SeqDB\&enzyme=Trypsin&access_method=Accession+Number&accession_num=AFK18949.1&coverage_map=0+70+12+6+24+22+22+2+17+139+19+33+35+17+11+78+15+42+14+5+37+46+11+141) | 3.84e+007 |
| A-type ATP synthase subunit I | [AFK18036.1](http://smserver.sti.ua.es/millhtml/SM_instruct/servadmn.htm#update_acclinks?AFK18036.1) | 15 | 13 | 238.25 | [22.4](http://smserver.sti.ua.es/millbin/msdigest.cgi?missed_cleavages=2&msparams_dir=msparams_mill/&hide_protein_sequence=2&database=PA_haloferax_mediterranei_genbank&seqdb_dir=D:\SeqDB\&enzyme=Trypsin&access_method=Accession+Number&accession_num=AFK18036.1&coverage_map=0+16+14+40+12+2+24+51+18+55+79+291+8+88+9+22) | 5.86e+007 |
| periplasmic solute binding protein | [AFK20085.1](http://smserver.sti.ua.es/millhtml/SM_instruct/servadmn.htm#update_acclinks?AFK20085.1) | 16 | 13 | 235.59 | [46](http://smserver.sti.ua.es/millbin/msdigest.cgi?missed_cleavages=2&msparams_dir=msparams_mill/&hide_protein_sequence=2&database=PA_haloferax_mediterranei_genbank&seqdb_dir=D:\SeqDB\&enzyme=Trypsin&access_method=Accession+Number&accession_num=AFK20085.1&coverage_map=0+119+41+14+22+7+17+3+17+27+19+4+21+16+28+3) | 1.35e+008 |
| dipeptide ABC transporter ATP-binding protein | [AFK18330.2](http://smserver.sti.ua.es/millhtml/SM_instruct/servadmn.htm#update_acclinks?AFK18330.2) | 18 | 13 | 231.75 | [32.8](http://smserver.sti.ua.es/millbin/msdigest.cgi?missed_cleavages=2&msparams_dir=msparams_mill/&hide_protein_sequence=2&database=PA_haloferax_mediterranei_genbank&seqdb_dir=D:\SeqDB\&enzyme=Trypsin&access_method=Accession+Number&accession_num=AFK18330.2&coverage_map=0+87+39+63+83+224+51+10+15) | 9.04e+007 |
| nucleoside-binding protein | [AFK19185.1](http://smserver.sti.ua.es/millhtml/SM_instruct/servadmn.htm#update_acclinks?AFK19185.1) | 15 | 12 | 209.36 | [33.4](http://smserver.sti.ua.es/millbin/msdigest.cgi?missed_cleavages=2&msparams_dir=msparams_mill/&hide_protein_sequence=2&database=PA_haloferax_mediterranei_genbank&seqdb_dir=D:\SeqDB\&enzyme=Trypsin&access_method=Accession+Number&accession_num=AFK19185.1&coverage_map=0+132+30+33+24+23+9+25+45+36+17) | 7.45e+007 |
| thermosome alpha subunit | [AFK18461.1](http://smserver.sti.ua.es/millhtml/SM_instruct/servadmn.htm#update_acclinks?AFK18461.1) | 14 | 12 | 196.35 | [31.4](http://smserver.sti.ua.es/millbin/msdigest.cgi?missed_cleavages=2&msparams_dir=msparams_mill/&hide_protein_sequence=2&database=PA_haloferax_mediterranei_genbank&seqdb_dir=D:\SeqDB\&enzyme=Trypsin&access_method=Accession+Number&accession_num=AFK18461.1&coverage_map=0+33+7+124+12+27+40+55+14+39+24+12+15+19+9+17+18+5+11+20+15+8) | 3.04e+007 |
| thermosome, beta subunit | [AFK18158.2](http://smserver.sti.ua.es/millhtml/SM_instruct/servadmn.htm#update_acclinks?AFK18158.2) | 14 | 11 | 190.56 | [29.4](http://smserver.sti.ua.es/millbin/msdigest.cgi?missed_cleavages=2&msparams_dir=msparams_mill/&hide_protein_sequence=2&database=PA_haloferax_mediterranei_genbank&seqdb_dir=D:\SeqDB\&enzyme=Trypsin&access_method=Accession+Number&accession_num=AFK18158.2&coverage_map=0+24+8+3+7+116+9+115+28+18+21+41+32+11+17+27+41+36) | 2.80e+007 |
| thermosome, alpha subunit | [AFK17883.2](http://smserver.sti.ua.es/millhtml/SM_instruct/servadmn.htm#update_acclinks?AFK17883.2) | 12 | 11 | 187.62 | [33](http://smserver.sti.ua.es/millbin/msdigest.cgi?missed_cleavages=2&msparams_dir=msparams_mill/&hide_protein_sequence=2&database=PA_haloferax_mediterranei_genbank&seqdb_dir=D:\SeqDB\&enzyme=Trypsin&access_method=Accession+Number&accession_num=AFK17883.2&coverage_map=0+4+38+96+20+15+29+21+24+58+7+17+22+36+10+33+35+95) | 2.01e+007 |
| PBS lyase HEAT-like repeat protein | [AFK18736.1](http://smserver.sti.ua.es/millhtml/SM_instruct/servadmn.htm#update_acclinks?AFK18736.1) | 14 | 10 | 176.14 | [34](http://smserver.sti.ua.es/millbin/msdigest.cgi?missed_cleavages=2&msparams_dir=msparams_mill/&hide_protein_sequence=2&database=PA_haloferax_mediterranei_genbank&seqdb_dir=D:\SeqDB\&enzyme=Trypsin&access_method=Accession+Number&accession_num=AFK18736.1&coverage_map=0+85+15+98+14+70+24+4+26+5+27+8+37+7) | 4.15e+007 |
| transmembrane oligosaccharyl transferase / dolichyl-diphosphooligosaccharide--protein glycosyltransferase | [AFK19298.1](http://smserver.sti.ua.es/millhtml/SM_instruct/servadmn.htm#update_acclinks?AFK19298.1) | 12 | 11 | 168.62 | [11.6](http://smserver.sti.ua.es/millbin/msdigest.cgi?missed_cleavages=2&msparams_dir=msparams_mill/&hide_protein_sequence=2&database=PA_haloferax_mediterranei_genbank&seqdb_dir=D:\SeqDB\&enzyme=Trypsin&access_method=Accession+Number&accession_num=AFK19298.1&coverage_map=0+14+7+28+16+57+22+68+6+147+11+16+8+255+12+43+10+42+9+62+8+11+13+182) | 1.21e+007 |
| A-type ATP synthase subunit D | [AFK18044.1](http://smserver.sti.ua.es/millhtml/SM_instruct/servadmn.htm#update_acclinks?AFK18044.1) | 14 | 11 | 159.41 | [48.6](http://smserver.sti.ua.es/millbin/msdigest.cgi?missed_cleavages=2&msparams_dir=msparams_mill/&hide_protein_sequence=2&database=PA_haloferax_mediterranei_genbank&seqdb_dir=D:\SeqDB\&enzyme=Trypsin&access_method=Accession+Number&accession_num=AFK18044.1&coverage_map=0+18+6+27+9+9+40+8+10+27+10+1+22+27+14) | 2.28e+007 |
| stress response protein | [AFK20336.2](http://smserver.sti.ua.es/millhtml/SM_instruct/servadmn.htm#update_acclinks?AFK20336.2) | 12 | 10 | 158.80 | [46.6](http://smserver.sti.ua.es/millbin/msdigest.cgi?missed_cleavages=2&msparams_dir=msparams_mill/&hide_protein_sequence=2&database=PA_haloferax_mediterranei_genbank&seqdb_dir=D:\SeqDB\&enzyme=Trypsin&access_method=Accession+Number&accession_num=AFK20336.2&coverage_map=0+6+6+2+21+21+5+36+24+21+26+4+36+6+13+54) | 2.67e+007 |
| ABC-type dipeptide/oligopeptide/nickel transport system, substrate binding protein | [AFK20131.1](http://smserver.sti.ua.es/millhtml/SM_instruct/servadmn.htm#update_acclinks?AFK20131.1) | 11 | 10 | 157.17 | [29.7](http://smserver.sti.ua.es/millbin/msdigest.cgi?missed_cleavages=2&msparams_dir=msparams_mill/&hide_protein_sequence=2&database=PA_haloferax_mediterranei_genbank&seqdb_dir=D:\SeqDB\&enzyme=Trypsin&access_method=Accession+Number&accession_num=AFK20131.1&coverage_map=0+99+16+34+32+31+14+22+9+30+23+86+23+2+16+34+19+42+9) | 1.79e+007 |
| hypothetical protein HFX_2220 | [AFK19909.2](http://smserver.sti.ua.es/millhtml/SM_instruct/servadmn.htm#update_acclinks?AFK19909.2) | 13 | 9 | 152.71 | [5.7](http://smserver.sti.ua.es/millbin/msdigest.cgi?missed_cleavages=2&msparams_dir=msparams_mill/&hide_protein_sequence=2&database=PA_haloferax_mediterranei_genbank&seqdb_dir=D:\SeqDB\&enzyme=Trypsin&access_method=Accession+Number&accession_num=AFK19909.2&coverage_map=0+138+8+623+27+282+11+932+36+25+19+22+29+93) | 5.08e+007 |
| glutamine synthetase | [AFK17986.2](http://smserver.sti.ua.es/millhtml/SM_instruct/servadmn.htm#update_acclinks?AFK17986.2) | 11 | 9 | 150.61 | [31.1](http://smserver.sti.ua.es/millbin/msdigest.cgi?missed_cleavages=2&msparams_dir=msparams_mill/&hide_protein_sequence=2&database=PA_haloferax_mediterranei_genbank&seqdb_dir=D:\SeqDB\&enzyme=Trypsin&access_method=Accession+Number&accession_num=AFK17986.2&coverage_map=0+26+17+11+39+9+19+188+16+22+25+30+26+28) | 3.64e+007 |
| NADH dehydrogenase | [AFK19343.1](http://smserver.sti.ua.es/millhtml/SM_instruct/servadmn.htm#update_acclinks?AFK19343.1) | 11 | 8 | 148.16 | [38](http://smserver.sti.ua.es/millbin/msdigest.cgi?missed_cleavages=2&msparams_dir=msparams_mill/&hide_protein_sequence=2&database=PA_haloferax_mediterranei_genbank&seqdb_dir=D:\SeqDB\&enzyme=Trypsin&access_method=Accession+Number&accession_num=AFK19343.1&coverage_map=0+51+24+2+9+65+21+4+27+80+38+31+18+4+14+9) | 1.97e+007 |
| flavin-dependent dehydrogenase | [AFK20419.1](http://smserver.sti.ua.es/millhtml/SM_instruct/servadmn.htm#update_acclinks?AFK20419.1) | 11 | 9 | 145.44 | [26.1](http://smserver.sti.ua.es/millbin/msdigest.cgi?missed_cleavages=2&msparams_dir=msparams_mill/&hide_protein_sequence=2&database=PA_haloferax_mediterranei_genbank&seqdb_dir=D:\SeqDB\&enzyme=Trypsin&access_method=Accession+Number&accession_num=AFK20419.1&coverage_map=0+113+8+6+17+4+29+18+44+67+19+48+28+153) | 1.43e+007 |
| nitrous-oxide reductase (plasmid) | [AFK20926.1](http://smserver.sti.ua.es/millhtml/SM_instruct/servadmn.htm#update_acclinks?AFK20926.1) | 9 | 9 | 140.39 | [11.7](http://smserver.sti.ua.es/millbin/msdigest.cgi?missed_cleavages=2&msparams_dir=msparams_mill/&hide_protein_sequence=2&database=PA_haloferax_mediterranei_genbank&seqdb_dir=D:\SeqDB\&enzyme=Trypsin&access_method=Accession+Number&accession_num=AFK20926.1&coverage_map=0+122+8+52+7+47+8+69+9+58+12+44+11+122+13+7+5+69+6+1) | 2.96e+007 |
| A-type ATP synthase subunit E | [AFK18038.1](http://smserver.sti.ua.es/millhtml/SM_instruct/servadmn.htm#update_acclinks?AFK18038.1) | 9 | 7 | 135.59 | [58.7](http://smserver.sti.ua.es/millbin/msdigest.cgi?missed_cleavages=2&msparams_dir=msparams_mill/&hide_protein_sequence=2&database=PA_haloferax_mediterranei_genbank&seqdb_dir=D:\SeqDB\&enzyme=Trypsin&access_method=Accession+Number&accession_num=AFK18038.1&coverage_map=0+17+30+12+8+39+58+2+18+10) | 2.24e+007 |
| membrane protease subunit, stomatin/prohibitin | [AFK17891.1](http://smserver.sti.ua.es/millhtml/SM_instruct/servadmn.htm#update_acclinks?AFK17891.1) | 11 | 8 | 134.69 | [34.3](http://smserver.sti.ua.es/millbin/msdigest.cgi?missed_cleavages=2&msparams_dir=msparams_mill/&hide_protein_sequence=2&database=PA_haloferax_mediterranei_genbank&seqdb_dir=D:\SeqDB\&enzyme=Trypsin&access_method=Accession+Number&accession_num=AFK17891.1&coverage_map=0+91+17+4+8+40+14+26+15+10+13+2+25+3+22+42) | 2.95e+007 |
| ABC-type dipeptide/oligopeptide/nickel transport system, substrate-binding protein | [AFK19559.1](http://smserver.sti.ua.es/millhtml/SM_instruct/servadmn.htm#update_acclinks?AFK19559.1) | 10 | 9 | 132.98 | [17.1](http://smserver.sti.ua.es/millbin/msdigest.cgi?missed_cleavages=2&msparams_dir=msparams_mill/&hide_protein_sequence=2&database=PA_haloferax_mediterranei_genbank&seqdb_dir=D:\SeqDB\&enzyme=Trypsin&access_method=Accession+Number&accession_num=AFK19559.1&coverage_map=0+192+7+127+12+17+7+26+14+75+35+9+21+10+15+81) | 8.67e+006 |
| poly(3-hydroxyalkanoate) synthase subunit PhaE (plasmid) | [AFK21053.1](http://smserver.sti.ua.es/millhtml/SM_instruct/servadmn.htm#update_acclinks?AFK21053.1) | 12 | 7 | 131.64 | [53.2](http://smserver.sti.ua.es/millbin/msdigest.cgi?missed_cleavages=2&msparams_dir=msparams_mill/&hide_protein_sequence=2&database=PA_haloferax_mediterranei_genbank&seqdb_dir=D:\SeqDB\&enzyme=Trypsin&access_method=Accession+Number&accession_num=AFK21053.1&coverage_map=0+62+31+12+57+11+9) | 1.14e+008 |
| potassium transport protein kefC | [AFK19701.1](http://smserver.sti.ua.es/millhtml/SM_instruct/servadmn.htm#update_acclinks?AFK19701.1) | 12 | 9 | 130.26 | [17.9](http://smserver.sti.ua.es/millbin/msdigest.cgi?missed_cleavages=2&msparams_dir=msparams_mill/&hide_protein_sequence=2&database=PA_haloferax_mediterranei_genbank&seqdb_dir=D:\SeqDB\&enzyme=Trypsin&access_method=Accession+Number&accession_num=AFK19701.1&coverage_map=0+337+9+73+8+56+47+30+17+3+17+18+7+4+9) | 1.90e+007 |
| glutamate dehydrogenase (NAD(P)+) | [AFK19225.1](http://smserver.sti.ua.es/millhtml/SM_instruct/servadmn.htm#update_acclinks?AFK19225.1) | 10 | 9 | 129.78 | [24.9](http://smserver.sti.ua.es/millbin/msdigest.cgi?missed_cleavages=2&msparams_dir=msparams_mill/&hide_protein_sequence=2&database=PA_haloferax_mediterranei_genbank&seqdb_dir=D:\SeqDB\&enzyme=Trypsin&access_method=Accession+Number&accession_num=AFK19225.1&coverage_map=0+37+19+29+9+12+17+17+12+47+8+159+39+12) | 1.02e+007 |
| glycosyl transferase | [AFK19287.1](http://smserver.sti.ua.es/millhtml/SM_instruct/servadmn.htm#update_acclinks?AFK19287.1) | 8 | 8 | 129.57 | [29.1](http://smserver.sti.ua.es/millbin/msdigest.cgi?missed_cleavages=2&msparams_dir=msparams_mill/&hide_protein_sequence=2&database=PA_haloferax_mediterranei_genbank&seqdb_dir=D:\SeqDB\&enzyme=Trypsin&access_method=Accession+Number&accession_num=AFK19287.1&coverage_map=0+44+16+7+11+24+14+33+32+28+20+7+19+129) | 4.73e+006 |
| putative phosphonate ABC transporter, periplasmic phosphonate-binding protein | [AFK19847.1](http://smserver.sti.ua.es/millhtml/SM_instruct/servadmn.htm#update_acclinks?AFK19847.1) | 8 | 7 | 129.42 | [23.4](http://smserver.sti.ua.es/millbin/msdigest.cgi?missed_cleavages=2&msparams_dir=msparams_mill/&hide_protein_sequence=2&database=PA_haloferax_mediterranei_genbank&seqdb_dir=D:\SeqDB\&enzyme=Trypsin&access_method=Accession+Number&accession_num=AFK19847.1&coverage_map=0+108+24+73+19+65+11+2+16+12+20+33) | 2.49e+007 |
| ABC-type iron(III) transport system,substrate-binding protein | [AFK19499.1](http://smserver.sti.ua.es/millhtml/SM_instruct/servadmn.htm#update_acclinks?AFK19499.1) | 9 | 7 | 128.61 | [33.5](http://smserver.sti.ua.es/millbin/msdigest.cgi?missed_cleavages=2&msparams_dir=msparams_mill/&hide_protein_sequence=2&database=PA_haloferax_mediterranei_genbank&seqdb_dir=D:\SeqDB\&enzyme=Trypsin&access_method=Accession+Number&accession_num=AFK19499.1&coverage_map=0+57+30+24+35+7+10+38+8+81+47+50) | 5.05e+007 |
| serine protease (plasmid) | [AFK21203.1](http://smserver.sti.ua.es/millhtml/SM_instruct/servadmn.htm#update_acclinks?AFK21203.1) | 10 | 9 | 128.28 | [20.8](http://smserver.sti.ua.es/millbin/msdigest.cgi?missed_cleavages=2&msparams_dir=msparams_mill/&hide_protein_sequence=2&database=PA_haloferax_mediterranei_genbank&seqdb_dir=D:\SeqDB\&enzyme=Trypsin&access_method=Accession+Number&accession_num=AFK21203.1&coverage_map=0+6+6+3+11+2+38+88+23+198+8+9+16+33+8+110+10+7) | 1.74e+007 |
| immunogenic protein | [AFK20638.1](http://smserver.sti.ua.es/millhtml/SM_instruct/servadmn.htm#update_acclinks?AFK20638.1) | 8 | 7 | 124.73 | [40.4](http://smserver.sti.ua.es/millbin/msdigest.cgi?missed_cleavages=2&msparams_dir=msparams_mill/&hide_protein_sequence=2&database=PA_haloferax_mediterranei_genbank&seqdb_dir=D:\SeqDB\&enzyme=Trypsin&access_method=Accession+Number&accession_num=AFK20638.1&coverage_map=0+57+20+28+63+42+37+69+16+4) | 5.53e+007 |
| isocitrate dehydrogenase (NADP+) | [AFK20291.1](http://smserver.sti.ua.es/millhtml/SM_instruct/servadmn.htm#update_acclinks?AFK20291.1) | 9 | 9 | 124.35 | [23.3](http://smserver.sti.ua.es/millbin/msdigest.cgi?missed_cleavages=2&msparams_dir=msparams_mill/&hide_protein_sequence=2&database=PA_haloferax_mediterranei_genbank&seqdb_dir=D:\SeqDB\&enzyme=Trypsin&access_method=Accession+Number&accession_num=AFK20291.1&coverage_map=0+50+11+14+16+7+19+23+11+128+12+58+20+41+9) | 9.85e+006 |
| CBS domain-containing protein | [AFK20079.1](http://smserver.sti.ua.es/millhtml/SM_instruct/servadmn.htm#update_acclinks?AFK20079.1) | 10 | 7 | 122.70 | [28.1](http://smserver.sti.ua.es/millbin/msdigest.cgi?missed_cleavages=2&msparams_dir=msparams_mill/&hide_protein_sequence=2&database=PA_haloferax_mediterranei_genbank&seqdb_dir=D:\SeqDB\&enzyme=Trypsin&access_method=Accession+Number&accession_num=AFK20079.1&coverage_map=1+19+14+17+42+9+95+24+5+26+30+13+86) | 2.11e+007 |
| putative signal transduction protein with CBS domains (plasmid) | [AFK21146.1](http://smserver.sti.ua.es/millhtml/SM_instruct/servadmn.htm#update_acclinks?AFK21146.1) | 8 | 8 | 122.31 | [20.2](http://smserver.sti.ua.es/millbin/msdigest.cgi?missed_cleavages=2&msparams_dir=msparams_mill/&hide_protein_sequence=2&database=PA_haloferax_mediterranei_genbank&seqdb_dir=D:\SeqDB\&enzyme=Trypsin&access_method=Accession+Number&accession_num=AFK21146.1&coverage_map=0+81+11+47+19+120+7+8+14+23+15+49+8+7+20+36) | 7.92e+006 |
| hypothetical protein HFX_5081 (plasmid) | [AFK20916.2](http://smserver.sti.ua.es/millhtml/SM_instruct/servadmn.htm#update_acclinks?AFK20916.2) | 9 | 8 | 121.68 | [32.1](http://smserver.sti.ua.es/millbin/msdigest.cgi?missed_cleavages=2&msparams_dir=msparams_mill/&hide_protein_sequence=2&database=PA_haloferax_mediterranei_genbank&seqdb_dir=D:\SeqDB\&enzyme=Trypsin&access_method=Accession+Number&accession_num=AFK20916.2&coverage_map=0+2+17+54+8+4+14+122+35+12+8+28+23) | 2.95e+007 |
| NADH dehydrogenase, subunit B (ubiquinone) | [AFK18695.1](http://smserver.sti.ua.es/millhtml/SM_instruct/servadmn.htm#update_acclinks?AFK18695.1) | 13 | 8 | 120.79 | [48.4](http://smserver.sti.ua.es/millbin/msdigest.cgi?missed_cleavages=2&msparams_dir=msparams_mill/&hide_protein_sequence=2&database=PA_haloferax_mediterranei_genbank&seqdb_dir=D:\SeqDB\&enzyme=Trypsin&access_method=Accession+Number&accession_num=AFK18695.1&coverage_map=0+23+7+6+12+39+7+4+14+7+60+34+13+7) | 2.81e+007 |
| hypothetical protein HFX_2459 | [AFK20144.2](http://smserver.sti.ua.es/millhtml/SM_instruct/servadmn.htm#update_acclinks?AFK20144.2) | 13 | 6 | 116.86 | [8.4](http://smserver.sti.ua.es/millbin/msdigest.cgi?missed_cleavages=2&msparams_dir=msparams_mill/&hide_protein_sequence=2&database=PA_haloferax_mediterranei_genbank&seqdb_dir=D:\SeqDB\&enzyme=Trypsin&access_method=Accession+Number&accession_num=AFK20144.2&coverage_map=0+288+12+5+12+274+29+92+13+48+10+121) | 2.04e+008 |
| amino acid-binding protein (plasmid) | [AFK21485.1](http://smserver.sti.ua.es/millhtml/SM_instruct/servadmn.htm#update_acclinks?AFK21485.1) | 7 | 7 | 116.54 | [28.4](http://smserver.sti.ua.es/millbin/msdigest.cgi?missed_cleavages=2&msparams_dir=msparams_mill/&hide_protein_sequence=2&database=PA_haloferax_mediterranei_genbank&seqdb_dir=D:\SeqDB\&enzyme=Trypsin&access_method=Accession+Number&accession_num=AFK21485.1&coverage_map=0+107+30+26+23+80+31+30+7+4+28+53) | 5.41e+006 |
| hypothetical protein HFX_6053 (plasmid) | [AFK21180.1](http://smserver.sti.ua.es/millhtml/SM_instruct/servadmn.htm#update_acclinks?AFK21180.1) | 7 | 7 | 113.93 | [26.6](http://smserver.sti.ua.es/millbin/msdigest.cgi?missed_cleavages=2&msparams_dir=msparams_mill/&hide_protein_sequence=2&database=PA_haloferax_mediterranei_genbank&seqdb_dir=D:\SeqDB\&enzyme=Trypsin&access_method=Accession+Number&accession_num=AFK21180.1&coverage_map=0+21+13+116+17+75+33+6+12+4+19+37) | 5.42e+006 |
| phosphate ABC transporter periplasmic substrate-binding protein | [AFK20069.1](http://smserver.sti.ua.es/millhtml/SM_instruct/servadmn.htm#update_acclinks?AFK20069.1) | 8 | 7 | 111.81 | [20.6](http://smserver.sti.ua.es/millbin/msdigest.cgi?missed_cleavages=2&msparams_dir=msparams_mill/&hide_protein_sequence=2&database=PA_haloferax_mediterranei_genbank&seqdb_dir=D:\SeqDB\&enzyme=Trypsin&access_method=Accession+Number&accession_num=AFK20069.1&coverage_map=0+163+10+52+7+58+54) | 1.19e+007 |
| ABC-type dipeptide/oligopeptide/nickel transport system, substrate-binding protein (plasmid) | [AFK21027.1](http://smserver.sti.ua.es/millhtml/SM_instruct/servadmn.htm#update_acclinks?AFK21027.1) | 8 | 8 | 108.79 | [14.9](http://smserver.sti.ua.es/millbin/msdigest.cgi?missed_cleavages=2&msparams_dir=msparams_mill/&hide_protein_sequence=2&database=PA_haloferax_mediterranei_genbank&seqdb_dir=D:\SeqDB\&enzyme=Trypsin&access_method=Accession+Number&accession_num=AFK21027.1&coverage_map=0+116+7+184+14+86+21+5+8+44+7+10+26+28) | 1.52e+007 |
| phosphonates ABC transporter ATP-binding protein | [AFK19846.1](http://smserver.sti.ua.es/millhtml/SM_instruct/servadmn.htm#update_acclinks?AFK19846.1) | 9 | 6 | 108.71 | [36.7](http://smserver.sti.ua.es/millbin/msdigest.cgi?missed_cleavages=2&msparams_dir=msparams_mill/&hide_protein_sequence=2&database=PA_haloferax_mediterranei_genbank&seqdb_dir=D:\SeqDB\&enzyme=Trypsin&access_method=Accession+Number&accession_num=AFK19846.1&coverage_map=0+9+7+77+22+17+19+14+28+22+21+28) | 7.96e+006 |
| aldehyde dehydrogenase | [AFK20313.1](http://smserver.sti.ua.es/millhtml/SM_instruct/servadmn.htm#update_acclinks?AFK20313.1) | 7 | 7 | 107.77 | [18.6](http://smserver.sti.ua.es/millbin/msdigest.cgi?missed_cleavages=2&msparams_dir=msparams_mill/&hide_protein_sequence=2&database=PA_haloferax_mediterranei_genbank&seqdb_dir=D:\SeqDB\&enzyme=Trypsin&access_method=Accession+Number&accession_num=AFK20313.1&coverage_map=0+89+13+17+8+134+16+19+12+5+28+11+9+139+11+10) | 6.00e+006 |
| FAD dependent oxidoreductase | [AFK19621.1](http://smserver.sti.ua.es/millhtml/SM_instruct/servadmn.htm#update_acclinks?AFK19621.1) | 7 | 7 | 103.68 | [19.6](http://smserver.sti.ua.es/millbin/msdigest.cgi?missed_cleavages=2&msparams_dir=msparams_mill/&hide_protein_sequence=2&database=PA_haloferax_mediterranei_genbank&seqdb_dir=D:\SeqDB\&enzyme=Trypsin&access_method=Accession+Number&accession_num=AFK19621.1&coverage_map=0+47+10+7+25+86+21+59+9+109+12+49+13+10) | 3.88e+006 |
| dihydrolipoamide S-acyltransferase (pyruvate dehydrogenase E2 component) | [AFK20618.1](http://smserver.sti.ua.es/millhtml/SM_instruct/servadmn.htm#update_acclinks?AFK20618.1) | 7 | 7 | 103.54 | [15.6](http://smserver.sti.ua.es/millbin/msdigest.cgi?missed_cleavages=2&msparams_dir=msparams_mill/&hide_protein_sequence=2&database=PA_haloferax_mediterranei_genbank&seqdb_dir=D:\SeqDB\&enzyme=Trypsin&access_method=Accession+Number&accession_num=AFK20618.1&coverage_map=0+112+8+4+17+7+18+168+7+45+12+54+9+27+7+5) | 6.61e+006 |
| putative hydrolase or acyltransferase of alpha/beta superfamily | [AFK18525.1](http://smserver.sti.ua.es/millhtml/SM_instruct/servadmn.htm#update_acclinks?AFK18525.1) | 7 | 6 | 101.36 | [30.8](http://smserver.sti.ua.es/millbin/msdigest.cgi?missed_cleavages=2&msparams_dir=msparams_mill/&hide_protein_sequence=2&database=PA_haloferax_mediterranei_genbank&seqdb_dir=D:\SeqDB\&enzyme=Trypsin&access_method=Accession+Number&accession_num=AFK18525.1&coverage_map=0+5+21+1+16+2+33+67+16+99+9+39) | 1.28e+007 |
| hypothetical protein HFX_1950 | [AFK19646.1](http://smserver.sti.ua.es/millhtml/SM_instruct/servadmn.htm#update_acclinks?AFK19646.1) | 6 | 6 | 99.96 | [45.2](http://smserver.sti.ua.es/millbin/msdigest.cgi?missed_cleavages=2&msparams_dir=msparams_mill/&hide_protein_sequence=2&database=PA_haloferax_mediterranei_genbank&seqdb_dir=D:\SeqDB\&enzyme=Trypsin&access_method=Accession+Number&accession_num=AFK19646.1&coverage_map=0+18+14+11+10+38+27+1+30+30) | 6.92e+006 |
| aldehyde dehydrogenase (NAD+) | [AFK18912.1](http://smserver.sti.ua.es/millhtml/SM_instruct/servadmn.htm#update_acclinks?AFK18912.1) | 7 | 7 | 99.83 | [15.3](http://smserver.sti.ua.es/millbin/msdigest.cgi?missed_cleavages=2&msparams_dir=msparams_mill/&hide_protein_sequence=2&database=PA_haloferax_mediterranei_genbank&seqdb_dir=D:\SeqDB\&enzyme=Trypsin&access_method=Accession+Number&accession_num=AFK18912.1&coverage_map=0+26+12+43+10+128+19+71+9+154+18+3+10+4) | 3.36e+006 |
| prepilin signal peptidase | [AFK20651.1](http://smserver.sti.ua.es/millhtml/SM_instruct/servadmn.htm#update_acclinks?AFK20651.1) | 7 | 7 | 98.16 | [22.8](http://smserver.sti.ua.es/millbin/msdigest.cgi?missed_cleavages=2&msparams_dir=msparams_mill/&hide_protein_sequence=2&database=PA_haloferax_mediterranei_genbank&seqdb_dir=D:\SeqDB\&enzyme=Trypsin&access_method=Accession+Number&accession_num=AFK20651.1&coverage_map=1+12+74+10+55+16+1+31+90+9+39) | 5.51e+006 |
| S-adenosylmethionine-dependent methyltransferase-like protein | [AFK18456.1](http://smserver.sti.ua.es/millhtml/SM_instruct/servadmn.htm#update_acclinks?AFK18456.1) | 7 | 7 | 97.99 | [29.7](http://smserver.sti.ua.es/millbin/msdigest.cgi?missed_cleavages=2&msparams_dir=msparams_mill/&hide_protein_sequence=2&database=PA_haloferax_mediterranei_genbank&seqdb_dir=D:\SeqDB\&enzyme=Trypsin&access_method=Accession+Number&accession_num=AFK18456.1&coverage_map=0+33+13+73+17+3+11+3+34+65) | 4.86e+006 |
| biotin carboxylase | [AFK20173.1](http://smserver.sti.ua.es/millhtml/SM_instruct/servadmn.htm#update_acclinks?AFK20173.1) | 6 | 6 | 97.76 | [10.9](http://smserver.sti.ua.es/millbin/msdigest.cgi?missed_cleavages=2&msparams_dir=msparams_mill/&hide_protein_sequence=2&database=PA_haloferax_mediterranei_genbank&seqdb_dir=D:\SeqDB\&enzyme=Trypsin&access_method=Accession+Number&accession_num=AFK20173.1&coverage_map=0+55+16+74+13+246+11+7+9+7+17+146) | 3.72e+006 |
| phosphoenolpyruvate synthase / pyruvate, water dikinase | [AFK18505.1](http://smserver.sti.ua.es/millhtml/SM_instruct/servadmn.htm#update_acclinks?AFK18505.1) | 7 | 7 | 94.93 | [9.1](http://smserver.sti.ua.es/millbin/msdigest.cgi?missed_cleavages=2&msparams_dir=msparams_mill/&hide_protein_sequence=2&database=PA_haloferax_mediterranei_genbank&seqdb_dir=D:\SeqDB\&enzyme=Trypsin&access_method=Accession+Number&accession_num=AFK18505.1&coverage_map=0+10+10+81+12+401+23+44+7+100+10+50+7) | 3.59e+006 |
| succinate dehydrogenase, subunit B (iron-sulfur protein) | [AFK20496.1](http://smserver.sti.ua.es/millhtml/SM_instruct/servadmn.htm#update_acclinks?AFK20496.1) | 7 | 7 | 94.87 | [23.8](http://smserver.sti.ua.es/millbin/msdigest.cgi?missed_cleavages=2&msparams_dir=msparams_mill/&hide_protein_sequence=2&database=PA_haloferax_mediterranei_genbank&seqdb_dir=D:\SeqDB\&enzyme=Trypsin&access_method=Accession+Number&accession_num=AFK20496.1&coverage_map=0+49+9+3+21+47+22+79+6+34+12+11) | 9.42e+006 |
| ABC-type dipeptide/oligopeptide/nickel transport system, substrate binding protein (plasmid) | [AFK20740.1](http://smserver.sti.ua.es/millhtml/SM_instruct/servadmn.htm#update_acclinks?AFK20740.1) | 6 | 6 | 94.51 | [14.5](http://smserver.sti.ua.es/millbin/msdigest.cgi?missed_cleavages=2&msparams_dir=msparams_mill/&hide_protein_sequence=2&database=PA_haloferax_mediterranei_genbank&seqdb_dir=D:\SeqDB\&enzyme=Trypsin&access_method=Accession+Number&accession_num=AFK20740.1&coverage_map=0+91+8+48+40+9+8+19+13+299+15+29) | 5.81e+006 |
| A-type ATP synthase subunit H | [AFK18035.1](http://smserver.sti.ua.es/millhtml/SM_instruct/servadmn.htm#update_acclinks?AFK18035.1) | 6 | 6 | 93.99 | [62.7](http://smserver.sti.ua.es/millbin/msdigest.cgi?missed_cleavages=2&msparams_dir=msparams_mill/&hide_protein_sequence=2&database=PA_haloferax_mediterranei_genbank&seqdb_dir=D:\SeqDB\&enzyme=Trypsin&access_method=Accession+Number&accession_num=AFK18035.1&coverage_map=0+11+25+7+14+14+30+9) | 4.64e+006 |
| NADH dehydrogenase/oxidoreductase-like protein | [AFK19950.1](http://smserver.sti.ua.es/millhtml/SM_instruct/servadmn.htm#update_acclinks?AFK19950.1) | 8 | 6 | 93.42 | [28.9](http://smserver.sti.ua.es/millbin/msdigest.cgi?missed_cleavages=2&msparams_dir=msparams_mill/&hide_protein_sequence=2&database=PA_haloferax_mediterranei_genbank&seqdb_dir=D:\SeqDB\&enzyme=Trypsin&access_method=Accession+Number&accession_num=AFK19950.1&coverage_map=0+107+19+39+17+40+41+28+10) | 8.53e+006 |
| gluconate dehydratase | [AFK19260.1](http://smserver.sti.ua.es/millhtml/SM_instruct/servadmn.htm#update_acclinks?AFK19260.1) | 6 | 6 | 93.39 | [17.9](http://smserver.sti.ua.es/millbin/msdigest.cgi?missed_cleavages=2&msparams_dir=msparams_mill/&hide_protein_sequence=2&database=PA_haloferax_mediterranei_genbank&seqdb_dir=D:\SeqDB\&enzyme=Trypsin&access_method=Accession+Number&accession_num=AFK19260.1&coverage_map=0+18+12+52+15+36+13+28+22+77+12+127) | 1.37e+007 |
| succinate--CoA ligase beta subunit (ADP-forming) | [AFK20155.1](http://smserver.sti.ua.es/millhtml/SM_instruct/servadmn.htm#update_acclinks?AFK20155.1) | 6 | 6 | 93.00 | [22.5](http://smserver.sti.ua.es/millbin/msdigest.cgi?missed_cleavages=2&msparams_dir=msparams_mill/&hide_protein_sequence=2&database=PA_haloferax_mediterranei_genbank&seqdb_dir=D:\SeqDB\&enzyme=Trypsin&access_method=Accession+Number&accession_num=AFK20155.1&coverage_map=0+9+36+24+12+73+9+73+10+76+19+40) | 2.59e+006 |
| oxidoreductase | [AFK18898.2](http://smserver.sti.ua.es/millhtml/SM_instruct/servadmn.htm#update_acclinks?AFK18898.2) | 6 | 6 | 92.12 | [30.4](http://smserver.sti.ua.es/millbin/msdigest.cgi?missed_cleavages=2&msparams_dir=msparams_mill/&hide_protein_sequence=2&database=PA_haloferax_mediterranei_genbank&seqdb_dir=D:\SeqDB\&enzyme=Trypsin&access_method=Accession+Number&accession_num=AFK18898.2&coverage_map=0+19+13+24+27+95+12+29+8+4+31+37) | 6.93e+006 |
| glutamate-1-semialdehyde aminotransferase | [AFK17832.1](http://smserver.sti.ua.es/millhtml/SM_instruct/servadmn.htm#update_acclinks?AFK17832.1) | 6 | 6 | 91.87 | [19.7](http://smserver.sti.ua.es/millbin/msdigest.cgi?missed_cleavages=2&msparams_dir=msparams_mill/&hide_protein_sequence=2&database=PA_haloferax_mediterranei_genbank&seqdb_dir=D:\SeqDB\&enzyme=Trypsin&access_method=Accession+Number&accession_num=AFK17832.1&coverage_map=0+12+14+24+27+33+14+127+26+125+7+36) | 4.32e+006 |
| proline dehydrogenase | [AFK18055.1](http://smserver.sti.ua.es/millhtml/SM_instruct/servadmn.htm#update_acclinks?AFK18055.1) | 7 | 6 | 90.53 | [24.7](http://smserver.sti.ua.es/millbin/msdigest.cgi?missed_cleavages=2&msparams_dir=msparams_mill/&hide_protein_sequence=2&database=PA_haloferax_mediterranei_genbank&seqdb_dir=D:\SeqDB\&enzyme=Trypsin&access_method=Accession+Number&accession_num=AFK18055.1&coverage_map=0+8+15+138+12+4+34+55+8+5) | 5.87e+006 |
| catalase (including: peroxidase) | [AFK19564.1](http://smserver.sti.ua.es/millhtml/SM_instruct/servadmn.htm#update_acclinks?AFK19564.1) | 6 | 6 | 90.52 | [10.3](http://smserver.sti.ua.es/millbin/msdigest.cgi?missed_cleavages=2&msparams_dir=msparams_mill/&hide_protein_sequence=2&database=PA_haloferax_mediterranei_genbank&seqdb_dir=D:\SeqDB\&enzyme=Trypsin&access_method=Accession+Number&accession_num=AFK19564.1&coverage_map=0+116+6+326+10+52+34+123+10+2+14+21) | 6.53e+006 |
| ABC-type branched-chain amino acid transport systems, substrate-binding protein | [AFK20482.1](http://smserver.sti.ua.es/millhtml/SM_instruct/servadmn.htm#update_acclinks?AFK20482.1) | 10 | 5 | 90.41 | [20.5](http://smserver.sti.ua.es/millbin/msdigest.cgi?missed_cleavages=2&msparams_dir=msparams_mill/&hide_protein_sequence=2&database=PA_haloferax_mediterranei_genbank&seqdb_dir=D:\SeqDB\&enzyme=Trypsin&access_method=Accession+Number&accession_num=AFK20482.1&coverage_map=0+57+19+167+30+86+41+39) | 2.92e+007 |
| hypothetical protein HFX_2976 | [AFK20640.1](http://smserver.sti.ua.es/millhtml/SM_instruct/servadmn.htm#update_acclinks?AFK20640.1) | 6 | 6 | 87.60 | [6.4](http://smserver.sti.ua.es/millbin/msdigest.cgi?missed_cleavages=2&msparams_dir=msparams_mill/&hide_protein_sequence=2&database=PA_haloferax_mediterranei_genbank&seqdb_dir=D:\SeqDB\&enzyme=Trypsin&access_method=Accession+Number&accession_num=AFK20640.1&coverage_map=0+153+8+59+8+421+7+131+25+99+13+21) | 1.07e+007 |
| ATP-dependent protease Lon | [AFK18465.1](http://smserver.sti.ua.es/millhtml/SM_instruct/servadmn.htm#update_acclinks?AFK18465.1) | 5 | 5 | 85.62 | [9.4](http://smserver.sti.ua.es/millbin/msdigest.cgi?missed_cleavages=2&msparams_dir=msparams_mill/&hide_protein_sequence=2&database=PA_haloferax_mediterranei_genbank&seqdb_dir=D:\SeqDB\&enzyme=Trypsin&access_method=Accession+Number&accession_num=AFK18465.1&coverage_map=0+125+8+106+20+27+14+84+11+65+13+222) | 3.61e+006 |
| branched-chain-amino-acid aminotransferase | [AFK18053.1](http://smserver.sti.ua.es/millhtml/SM_instruct/servadmn.htm#update_acclinks?AFK18053.1) | 6 | 6 | 85.22 | [26.9](http://smserver.sti.ua.es/millbin/msdigest.cgi?missed_cleavages=2&msparams_dir=msparams_mill/&hide_protein_sequence=2&database=PA_haloferax_mediterranei_genbank&seqdb_dir=D:\SeqDB\&enzyme=Trypsin&access_method=Accession+Number&accession_num=AFK18053.1&coverage_map=0+161+16+12+16+3+24+37+28+15) | 5.02e+006 |
| dipeptide/oligopeptide/nickel ABC transporter ATP-binding protein | [AFK17802.1](http://smserver.sti.ua.es/millhtml/SM_instruct/servadmn.htm#update_acclinks?AFK17802.1) | 6 | 5 | 85.13 | [14.7](http://smserver.sti.ua.es/millbin/msdigest.cgi?missed_cleavages=2&msparams_dir=msparams_mill/&hide_protein_sequence=2&database=PA_haloferax_mediterranei_genbank&seqdb_dir=D:\SeqDB\&enzyme=Trypsin&access_method=Accession+Number&accession_num=AFK17802.1&coverage_map=0+31+15+79+8+51+25+108+11+13+8+106) | 4.73e+006 |
| hypothetical protein HFX_1766 | [AFK19471.1](http://smserver.sti.ua.es/millhtml/SM_instruct/servadmn.htm#update_acclinks?AFK19471.1) | 7 | 6 | 84.98 | [30.5](http://smserver.sti.ua.es/millbin/msdigest.cgi?missed_cleavages=2&msparams_dir=msparams_mill/&hide_protein_sequence=2&database=PA_haloferax_mediterranei_genbank&seqdb_dir=D:\SeqDB\&enzyme=Trypsin&access_method=Accession+Number&accession_num=AFK19471.1&coverage_map=0+62+9+13+28+25+8+19+14+23+7+8) | 2.09e+007 |
| gas-vesicle operon protein gvpC | [AFK19401.1](http://smserver.sti.ua.es/millhtml/SM_instruct/servadmn.htm#update_acclinks?AFK19401.1) | 7 | 5 | 84.95 | [17.8](http://smserver.sti.ua.es/millbin/msdigest.cgi?missed_cleavages=2&msparams_dir=msparams_mill/&hide_protein_sequence=2&database=PA_haloferax_mediterranei_genbank&seqdb_dir=D:\SeqDB\&enzyme=Trypsin&access_method=Accession+Number&accession_num=AFK19401.1&coverage_map=0+39+24+126+30+7+14+141) | 8.55e+006 |
| ABC-type glutamine/glutamate/polar amino acids transport system, ATP-binding protein | [AFK20124.1](http://smserver.sti.ua.es/millhtml/SM_instruct/servadmn.htm#update_acclinks?AFK20124.1) | 5 | 5 | 84.49 | [22.3](http://smserver.sti.ua.es/millbin/msdigest.cgi?missed_cleavages=2&msparams_dir=msparams_mill/&hide_protein_sequence=2&database=PA_haloferax_mediterranei_genbank&seqdb_dir=D:\SeqDB\&enzyme=Trypsin&access_method=Accession+Number&accession_num=AFK20124.1&coverage_map=0+11+9+31+25+18+12+101+8+27) | 5.17e+006 |
| hypothetical protein HFX_2780 | [AFK20456.1](http://smserver.sti.ua.es/millhtml/SM_instruct/servadmn.htm#update_acclinks?AFK20456.1) | 7 | 6 | 82.95 | [21.2](http://smserver.sti.ua.es/millbin/msdigest.cgi?missed_cleavages=2&msparams_dir=msparams_mill/&hide_protein_sequence=2&database=PA_haloferax_mediterranei_genbank&seqdb_dir=D:\SeqDB\&enzyme=Trypsin&access_method=Accession+Number&accession_num=AFK20456.1&coverage_map=0+31+23+7+18+76+11+32+16+106) | 3.46e+006 |
| pyruvate--ferredoxin oxidoreductase, alpha subunit | [AFK19081.1](http://smserver.sti.ua.es/millhtml/SM_instruct/servadmn.htm#update_acclinks?AFK19081.1) | 7 | 5 | 82.63 | [12.6](http://smserver.sti.ua.es/millbin/msdigest.cgi?missed_cleavages=2&msparams_dir=msparams_mill/&hide_protein_sequence=2&database=PA_haloferax_mediterranei_genbank&seqdb_dir=D:\SeqDB\&enzyme=Trypsin&access_method=Accession+Number&accession_num=AFK19081.1&coverage_map=0+65+16+73+20+180+16+4+16+147+12+82) | 4.88e+006 |
| oligopeptide ABC transporter ATPase component | [AFK17803.1](http://smserver.sti.ua.es/millhtml/SM_instruct/servadmn.htm#update_acclinks?AFK17803.1) | 5 | 5 | 79.94 | [18.8](http://smserver.sti.ua.es/millbin/msdigest.cgi?missed_cleavages=2&msparams_dir=msparams_mill/&hide_protein_sequence=2&database=PA_haloferax_mediterranei_genbank&seqdb_dir=D:\SeqDB\&enzyme=Trypsin&access_method=Accession+Number&accession_num=AFK17803.1&coverage_map=0+6+12+11+25+21+11+48+18+198) | 2.50e+007 |
| short chain dehydrogenase/ reductase | [AFK18407.1](http://smserver.sti.ua.es/millhtml/SM_instruct/servadmn.htm#update_acclinks?AFK18407.1) | 5 | 5 | 78.60 | [26.3](http://smserver.sti.ua.es/millbin/msdigest.cgi?missed_cleavages=2&msparams_dir=msparams_mill/&hide_protein_sequence=2&database=PA_haloferax_mediterranei_genbank&seqdb_dir=D:\SeqDB\&enzyme=Trypsin&access_method=Accession+Number&accession_num=AFK18407.1&coverage_map=0+4+13+29+17+58+16+13+11+71+17+32) | 4.63e+006 |
| dihydrolipoamide dehydrogenase | [AFK20619.1](http://smserver.sti.ua.es/millhtml/SM_instruct/servadmn.htm#update_acclinks?AFK20619.1) | 5 | 5 | 78.57 | [12.6](http://smserver.sti.ua.es/millbin/msdigest.cgi?missed_cleavages=2&msparams_dir=msparams_mill/&hide_protein_sequence=2&database=PA_haloferax_mediterranei_genbank&seqdb_dir=D:\SeqDB\&enzyme=Trypsin&access_method=Accession+Number&accession_num=AFK20619.1&coverage_map=0+27+13+51+13+48+18+101+7+19+9+169) | 4.45e+006 |
| gas-vesicle operon protein gvpA | [AFK19402.1](http://smserver.sti.ua.es/millhtml/SM_instruct/servadmn.htm#update_acclinks?AFK19402.1) | 5 | 4 | 78.17 | [70.5](http://smserver.sti.ua.es/millbin/msdigest.cgi?missed_cleavages=2&msparams_dir=msparams_mill/&hide_protein_sequence=2&database=PA_haloferax_mediterranei_genbank&seqdb_dir=D:\SeqDB\&enzyme=Trypsin&access_method=Accession+Number&accession_num=AFK19402.1&coverage_map=1+15+4+23+18+18) | 3.01e+007 |
| ABC transporter ATP-binding protein | [AFK20685.1](http://smserver.sti.ua.es/millhtml/SM_instruct/servadmn.htm#update_acclinks?AFK20685.1) | 5 | 5 | 78.12 | [19.3](http://smserver.sti.ua.es/millbin/msdigest.cgi?missed_cleavages=2&msparams_dir=msparams_mill/&hide_protein_sequence=2&database=PA_haloferax_mediterranei_genbank&seqdb_dir=D:\SeqDB\&enzyme=Trypsin&access_method=Accession+Number&accession_num=AFK20685.1&coverage_map=0+92+13+14+22+102+27+51) | 2.14e+006 |
| poly(3-hydroxyalkanoate) granule-associated 12 kDa protein (plasmid) | [AFK21051.1](http://smserver.sti.ua.es/millhtml/SM_instruct/servadmn.htm#update_acclinks?AFK21051.1) | 7 | 4 | 77.21 | [34.5](http://smserver.sti.ua.es/millbin/msdigest.cgi?missed_cleavages=2&msparams_dir=msparams_mill/&hide_protein_sequence=2&database=PA_haloferax_mediterranei_genbank&seqdb_dir=D:\SeqDB\&enzyme=Trypsin&access_method=Accession+Number&accession_num=AFK21051.1&coverage_map=0+21+11+43+27+8) | 2.33e+007 |
| poly(3-hydroxyalkanoate) synthase subunit PhaC | [AFK20356.1](http://smserver.sti.ua.es/millhtml/SM_instruct/servadmn.htm#update_acclinks?AFK20356.1) | 6 | 5 | 76.87 | [17.4](http://smserver.sti.ua.es/millbin/msdigest.cgi?missed_cleavages=2&msparams_dir=msparams_mill/&hide_protein_sequence=2&database=PA_haloferax_mediterranei_genbank&seqdb_dir=D:\SeqDB\&enzyme=Trypsin&access_method=Accession+Number&accession_num=AFK20356.1&coverage_map=0+128+10+63+29+25+11+86+6+43+21+20) | 2.19e+006 |
| iron-sulfur protein (4Fe-4S) | [AFK17960.2](http://smserver.sti.ua.es/millhtml/SM_instruct/servadmn.htm#update_acclinks?AFK17960.2) | 5 | 5 | 76.20 | [7.8](http://smserver.sti.ua.es/millbin/msdigest.cgi?missed_cleavages=2&msparams_dir=msparams_mill/&hide_protein_sequence=2&database=PA_haloferax_mediterranei_genbank&seqdb_dir=D:\SeqDB\&enzyme=Trypsin&access_method=Accession+Number&accession_num=AFK17960.2&coverage_map=0+57+7+230+10+20+7+304+31+38) | 6.36e+006 |
| putative mechanosensitive ion channel | [AFK19964.1](http://smserver.sti.ua.es/millhtml/SM_instruct/servadmn.htm#update_acclinks?AFK19964.1) | 5 | 5 | 75.61 | [24.6](http://smserver.sti.ua.es/millbin/msdigest.cgi?missed_cleavages=2&msparams_dir=msparams_mill/&hide_protein_sequence=2&database=PA_haloferax_mediterranei_genbank&seqdb_dir=D:\SeqDB\&enzyme=Trypsin&access_method=Accession+Number&accession_num=AFK19964.1&coverage_map=0+53+7+130+67+43) | 3.80e+006 |
| ArsR family regulatory protein | [AFK19320.1](http://smserver.sti.ua.es/millhtml/SM_instruct/servadmn.htm#update_acclinks?AFK19320.1) | 4 | 4 | 74.93 | [34.4](http://smserver.sti.ua.es/millbin/msdigest.cgi?missed_cleavages=2&msparams_dir=msparams_mill/&hide_protein_sequence=2&database=PA_haloferax_mediterranei_genbank&seqdb_dir=D:\SeqDB\&enzyme=Trypsin&access_method=Accession+Number&accession_num=AFK19320.1&coverage_map=0+20+22+1+49+114) | 3.55e+006 |
| menaquinol--cytochrome-c reductase (cytochrome bc complex) cytochrome b/c subunit | [AFK18533.1](http://smserver.sti.ua.es/millhtml/SM_instruct/servadmn.htm#update_acclinks?AFK18533.1) | 5 | 4 | 73.76 | [18.1](http://smserver.sti.ua.es/millbin/msdigest.cgi?missed_cleavages=2&msparams_dir=msparams_mill/&hide_protein_sequence=2&database=PA_haloferax_mediterranei_genbank&seqdb_dir=D:\SeqDB\&enzyme=Trypsin&access_method=Accession+Number&accession_num=AFK18533.1&coverage_map=0+40+8+17+14+50+15+92+9+9) | 1.22e+007 |
| cytochrome bc1 complex cytochrome b/c subunit | [AFK18497.1](http://smserver.sti.ua.es/millhtml/SM_instruct/servadmn.htm#update_acclinks?AFK18497.1) | 4 | 4 | 67.42 | [17.4](http://smserver.sti.ua.es/millbin/msdigest.cgi?missed_cleavages=2&msparams_dir=msparams_mill/&hide_protein_sequence=2&database=PA_haloferax_mediterranei_genbank&seqdb_dir=D:\SeqDB\&enzyme=Trypsin&access_method=Accession+Number&accession_num=AFK18497.1&coverage_map=0+49+8+17+14+50+15+92+9+9) | 9.19e+006 |
| hypothetical protein HFX_0293 | [AFK18032.2](http://smserver.sti.ua.es/millhtml/SM_instruct/servadmn.htm#update_acclinks?AFK18032.2) | 5 | 5 | 73.04 | [14.9](http://smserver.sti.ua.es/millbin/msdigest.cgi?missed_cleavages=2&msparams_dir=msparams_mill/&hide_protein_sequence=2&database=PA_haloferax_mediterranei_genbank&seqdb_dir=D:\SeqDB\&enzyme=Trypsin&access_method=Accession+Number&accession_num=AFK18032.2&coverage_map=0+106+10+18+9+24+15+35+15+97+17+96) | 1.11e+006 |
| menaquinol--cytochrome-c reductase | [AFK20314.1](http://smserver.sti.ua.es/millhtml/SM_instruct/servadmn.htm#update_acclinks?AFK20314.1) | 5 | 4 | 72.52 | [19.5](http://smserver.sti.ua.es/millbin/msdigest.cgi?missed_cleavages=2&msparams_dir=msparams_mill/&hide_protein_sequence=2&database=PA_haloferax_mediterranei_genbank&seqdb_dir=D:\SeqDB\&enzyme=Trypsin&access_method=Accession+Number&accession_num=AFK20314.1&coverage_map=0+65+16+106+14+3+25+52) | 2.14e+007 |
| zinc-transporting ATPase (plasmid) | [AFK21399.1](http://smserver.sti.ua.es/millhtml/SM_instruct/servadmn.htm#update_acclinks?AFK21399.1) | 5 | 5 | 71.52 | [7.7](http://smserver.sti.ua.es/millbin/msdigest.cgi?missed_cleavages=2&msparams_dir=msparams_mill/&hide_protein_sequence=2&database=PA_haloferax_mediterranei_genbank&seqdb_dir=D:\SeqDB\&enzyme=Trypsin&access_method=Accession+Number&accession_num=AFK21399.1&coverage_map=0+94+9+41+22+175+13+74+12+294+12+136) | 1.23e+006 |
| oxidoreductase (geranylgeranyl hydrogenase-like protein) | [AFK19602.1](http://smserver.sti.ua.es/millhtml/SM_instruct/servadmn.htm#update_acclinks?AFK19602.1) | 5 | 4 | 71.13 | [13.1](http://smserver.sti.ua.es/millbin/msdigest.cgi?missed_cleavages=2&msparams_dir=msparams_mill/&hide_protein_sequence=2&database=PA_haloferax_mediterranei_genbank&seqdb_dir=D:\SeqDB\&enzyme=Trypsin&access_method=Accession+Number&accession_num=AFK19602.1&coverage_map=0+117+15+86+12+131+10+5+17+17) | 1.99e+006 |
| poly(3-hydroxyalkanoate) granule-associated protein(phasin) (plasmid) | [AFK21052.1](http://smserver.sti.ua.es/millhtml/SM_instruct/servadmn.htm#update_acclinks?AFK21052.1) | 12 | 5 | 70.72 | [27.2](http://smserver.sti.ua.es/millbin/msdigest.cgi?missed_cleavages=2&msparams_dir=msparams_mill/&hide_protein_sequence=2&database=PA_haloferax_mediterranei_genbank&seqdb_dir=D:\SeqDB\&enzyme=Trypsin&access_method=Accession+Number&accession_num=AFK21052.1&coverage_map=0+17+7+14+14+11+21+70) | 1.44e+008 |
| methanol dehydrogenase regulatory protein | [AFK18978.1](http://smserver.sti.ua.es/millhtml/SM_instruct/servadmn.htm#update_acclinks?AFK18978.1) | 5 | 5 | 70.71 | [15.1](http://smserver.sti.ua.es/millbin/msdigest.cgi?missed_cleavages=2&msparams_dir=msparams_mill/&hide_protein_sequence=2&database=PA_haloferax_mediterranei_genbank&seqdb_dir=D:\SeqDB\&enzyme=Trypsin&access_method=Accession+Number&accession_num=AFK18978.1&coverage_map=0+96+12+31+15+4+12+88+8+2+6+75) | 3.06e+006 |
| methanol dehydrogenase regulatory protein (plasmid) | [AFK21086.1](http://smserver.sti.ua.es/millhtml/SM_instruct/servadmn.htm#update_acclinks?AFK21086.1) | 1 | 1 | 21.38 | [4.6](http://smserver.sti.ua.es/millbin/msdigest.cgi?missed_cleavages=2&msparams_dir=msparams_mill/&hide_protein_sequence=2&database=PA_haloferax_mediterranei_genbank&seqdb_dir=D:\SeqDB\&enzyme=Trypsin&access_method=Accession+Number&accession_num=AFK21086.1&coverage_map=0+97+15+208) | 6.91e+005 |
| hypothetical protein HFX_0491 | [AFK18222.1](http://smserver.sti.ua.es/millhtml/SM_instruct/servadmn.htm#update_acclinks?AFK18222.1) | 6 | 4 | 69.82 | [34.1](http://smserver.sti.ua.es/millbin/msdigest.cgi?missed_cleavages=2&msparams_dir=msparams_mill/&hide_protein_sequence=2&database=PA_haloferax_mediterranei_genbank&seqdb_dir=D:\SeqDB\&enzyme=Trypsin&access_method=Accession+Number&accession_num=AFK18222.1&coverage_map=0+73+12+10+33+15+11+10) | 3.62e+006 |
| 3-oxoacyl-[acyl-carrier protein] reductase | [AFK19230.1](http://smserver.sti.ua.es/millhtml/SM_instruct/servadmn.htm#update_acclinks?AFK19230.1) | 5 | 4 | 69.76 | [19.4](http://smserver.sti.ua.es/millbin/msdigest.cgi?missed_cleavages=2&msparams_dir=msparams_mill/&hide_protein_sequence=2&database=PA_haloferax_mediterranei_genbank&seqdb_dir=D:\SeqDB\&enzyme=Trypsin&access_method=Accession+Number&accession_num=AFK19230.1&coverage_map=0+7+13+46+13+89+12+25+11+36) | 8.13e+006 |
| hypothetical protein HFX_0588 | [AFK18313.1](http://smserver.sti.ua.es/millhtml/SM_instruct/servadmn.htm#update_acclinks?AFK18313.1) | 5 | 4 | 68.80 | [16.2](http://smserver.sti.ua.es/millbin/msdigest.cgi?missed_cleavages=2&msparams_dir=msparams_mill/&hide_protein_sequence=2&database=PA_haloferax_mediterranei_genbank&seqdb_dir=D:\SeqDB\&enzyme=Trypsin&access_method=Accession+Number&accession_num=AFK18313.1&coverage_map=0+162+15+2+13+57+28+67) | 8.92e+006 |
| pyridoxal biosynthesis lyase PdxS | [AFK20032.1](http://smserver.sti.ua.es/millhtml/SM_instruct/servadmn.htm#update_acclinks?AFK20032.1) | 5 | 5 | 68.42 | [17.5](http://smserver.sti.ua.es/millbin/msdigest.cgi?missed_cleavages=2&msparams_dir=msparams_mill/&hide_protein_sequence=2&database=PA_haloferax_mediterranei_genbank&seqdb_dir=D:\SeqDB\&enzyme=Trypsin&access_method=Accession+Number&accession_num=AFK20032.1&coverage_map=0+19+21+36+13+57+9+116+10+21) | 2.53e+006 |
| sugar ABC transporter ATP-binding protein | [AFK20383.1](http://smserver.sti.ua.es/millhtml/SM_instruct/servadmn.htm#update_acclinks?AFK20383.1) | 6 | 4 | 68.19 | [17.2](http://smserver.sti.ua.es/millbin/msdigest.cgi?missed_cleavages=2&msparams_dir=msparams_mill/&hide_protein_sequence=2&database=PA_haloferax_mediterranei_genbank&seqdb_dir=D:\SeqDB\&enzyme=Trypsin&access_method=Accession+Number&accession_num=AFK20383.1&coverage_map=0+42+17+57+13+21+17+97+21+110) | 1.03e+007 |
| hypothetical protein HFX_1575 | [AFK19282.2](http://smserver.sti.ua.es/millhtml/SM_instruct/servadmn.htm#update_acclinks?AFK19282.2) | 5 | 4 | 68.10 | [22.3](http://smserver.sti.ua.es/millbin/msdigest.cgi?missed_cleavages=2&msparams_dir=msparams_mill/&hide_protein_sequence=2&database=PA_haloferax_mediterranei_genbank&seqdb_dir=D:\SeqDB\&enzyme=Trypsin&access_method=Accession+Number&accession_num=AFK19282.2&coverage_map=0+77+8+24+14+63+18+8+11+5) | 1.46e+007 |
| lysophospholipase | [AFK20242.1](http://smserver.sti.ua.es/millhtml/SM_instruct/servadmn.htm#update_acclinks?AFK20242.1) | 4 | 4 | 67.95 | [16.6](http://smserver.sti.ua.es/millbin/msdigest.cgi?missed_cleavages=2&msparams_dir=msparams_mill/&hide_protein_sequence=2&database=PA_haloferax_mediterranei_genbank&seqdb_dir=D:\SeqDB\&enzyme=Trypsin&access_method=Accession+Number&accession_num=AFK20242.1&coverage_map=0+98+20+31+14+9+11+51+7+72) | 6.68e+006 |
| hypothetical protein HFX_2555 | [AFK20236.1](http://smserver.sti.ua.es/millhtml/SM_instruct/servadmn.htm#update_acclinks?AFK20236.1) | 4 | 4 | 67.69 | [17.2](http://smserver.sti.ua.es/millbin/msdigest.cgi?missed_cleavages=2&msparams_dir=msparams_mill/&hide_protein_sequence=2&database=PA_haloferax_mediterranei_genbank&seqdb_dir=D:\SeqDB\&enzyme=Trypsin&access_method=Accession+Number&accession_num=AFK20236.1&coverage_map=0+83+13+23+12+98+33+74) | 5.96e+006 |
| orotate phosphoribosyltransferase-like protein/conserved Entner-Douderoff pathway protein | [AFK18805.1](http://smserver.sti.ua.es/millhtml/SM_instruct/servadmn.htm#update_acclinks?AFK18805.1) | 6 | 5 | 67.52 | [25.2](http://smserver.sti.ua.es/millbin/msdigest.cgi?missed_cleavages=2&msparams_dir=msparams_mill/&hide_protein_sequence=2&database=PA_haloferax_mediterranei_genbank&seqdb_dir=D:\SeqDB\&enzyme=Trypsin&access_method=Accession+Number&accession_num=AFK18805.1&coverage_map=0+2+15+4+20+59+11+54+7+38) | 4.56e+006 |
| putative RNA-associated protein | [AFK19132.1](http://smserver.sti.ua.es/millhtml/SM_instruct/servadmn.htm#update_acclinks?AFK19132.1) | 4 | 4 | 67.21 | [23.9](http://smserver.sti.ua.es/millbin/msdigest.cgi?missed_cleavages=2&msparams_dir=msparams_mill/&hide_protein_sequence=2&database=PA_haloferax_mediterranei_genbank&seqdb_dir=D:\SeqDB\&enzyme=Trypsin&access_method=Accession+Number&accession_num=AFK19132.1&coverage_map=1+11+7+14+97+33+76) | 3.98e+006 |
| AAA-type ATPase (transitional ATPase-like protein) | [AFK20391.1](http://smserver.sti.ua.es/millhtml/SM_instruct/servadmn.htm#update_acclinks?AFK20391.1) | 5 | 5 | 67.09 | [6.4](http://smserver.sti.ua.es/millbin/msdigest.cgi?missed_cleavages=2&msparams_dir=msparams_mill/&hide_protein_sequence=2&database=PA_haloferax_mediterranei_genbank&seqdb_dir=D:\SeqDB\&enzyme=Trypsin&access_method=Accession+Number&accession_num=AFK20391.1&coverage_map=0+93+10+100+8+98+15+119+8+188+8+107) | 1.75e+006 |
| pyruvate--ferredoxin oxidoreductase, beta subunit | [AFK19080.1](http://smserver.sti.ua.es/millhtml/SM_instruct/servadmn.htm#update_acclinks?AFK19080.1) | 4 | 4 | 67.04 | [16.9](http://smserver.sti.ua.es/millbin/msdigest.cgi?missed_cleavages=2&msparams_dir=msparams_mill/&hide_protein_sequence=2&database=PA_haloferax_mediterranei_genbank&seqdb_dir=D:\SeqDB\&enzyme=Trypsin&access_method=Accession+Number&accession_num=AFK19080.1&coverage_map=0+241+23+16+30+2) | 2.66e+006 |
| aminopeptidase (plasmid) | [AFK21163.2](http://smserver.sti.ua.es/millhtml/SM_instruct/servadmn.htm#update_acclinks?AFK21163.2) | 5 | 5 | 66.23 | [11.1](http://smserver.sti.ua.es/millbin/msdigest.cgi?missed_cleavages=2&msparams_dir=msparams_mill/&hide_protein_sequence=2&database=PA_haloferax_mediterranei_genbank&seqdb_dir=D:\SeqDB\&enzyme=Trypsin&access_method=Accession+Number&accession_num=AFK21163.2&coverage_map=0+4+17+73+10+89+12+189+8+20) | 1.40e+007 |
| hypothetical protein HFX_1249 | [AFK18962.1](http://smserver.sti.ua.es/millhtml/SM_instruct/servadmn.htm#update_acclinks?AFK18962.1) | 5 | 5 | 66.15 | [8.7](http://smserver.sti.ua.es/millbin/msdigest.cgi?missed_cleavages=2&msparams_dir=msparams_mill/&hide_protein_sequence=2&database=PA_haloferax_mediterranei_genbank&seqdb_dir=D:\SeqDB\&enzyme=Trypsin&access_method=Accession+Number&accession_num=AFK18962.1&coverage_map=0+123+15+60+11+184+12+27) | 1.73e+006 |
| hypothetical protein HFX_6278 (plasmid) | [AFK21400.1](http://smserver.sti.ua.es/millhtml/SM_instruct/servadmn.htm#update_acclinks?AFK21400.1) | 4 | 4 | 66.00 | [29.9](http://smserver.sti.ua.es/millbin/msdigest.cgi?missed_cleavages=2&msparams_dir=msparams_mill/&hide_protein_sequence=2&database=PA_haloferax_mediterranei_genbank&seqdb_dir=D:\SeqDB\&enzyme=Trypsin&access_method=Accession+Number&accession_num=AFK21400.1&coverage_map=0+65+18+9+20+15) | 5.21e+006 |
| hypothetical protein HFX_6273 (plasmid) | [AFK21396.2](http://smserver.sti.ua.es/millhtml/SM_instruct/servadmn.htm#update_acclinks?AFK21396.2) | 4 | 4 | 65.92 | [15.1](http://smserver.sti.ua.es/millbin/msdigest.cgi?missed_cleavages=2&msparams_dir=msparams_mill/&hide_protein_sequence=2&database=PA_haloferax_mediterranei_genbank&seqdb_dir=D:\SeqDB\&enzyme=Trypsin&access_method=Accession+Number&accession_num=AFK21396.2&coverage_map=0+32+22+21+11+109+8+55+13+85) | 1.61e+006 |
| ferredoxin (2Fe-2S) | [AFK19572.1](http://smserver.sti.ua.es/millhtml/SM_instruct/servadmn.htm#update_acclinks?AFK19572.1) | 4 | 4 | 65.80 | [29.3](http://smserver.sti.ua.es/millbin/msdigest.cgi?missed_cleavages=2&msparams_dir=msparams_mill/&hide_protein_sequence=2&database=PA_haloferax_mediterranei_genbank&seqdb_dir=D:\SeqDB\&enzyme=Trypsin&access_method=Accession+Number&accession_num=AFK19572.1&coverage_map=0+26+17+1+15+88+14+22+11) | 4.85e+006 |
| ABC-type dipeptide/oligopeptide/nickel transport systems, ATP-binding protein I & II | [AFK18329.1](http://smserver.sti.ua.es/millhtml/SM_instruct/servadmn.htm#update_acclinks?AFK18329.1) | 5 | 5 | 65.62 | [6.9](http://smserver.sti.ua.es/millbin/msdigest.cgi?missed_cleavages=2&msparams_dir=msparams_mill/&hide_protein_sequence=2&database=PA_haloferax_mediterranei_genbank&seqdb_dir=D:\SeqDB\&enzyme=Trypsin&access_method=Accession+Number&accession_num=AFK18329.1&coverage_map=0+163+25+15+12+242+17+20+8+396) | 3.26e+006 |
| preprotein translocase subunit SecY | [AFK20246.1](http://smserver.sti.ua.es/millhtml/SM_instruct/servadmn.htm#update_acclinks?AFK20246.1) | 6 | 4 | 65.47 | [8.5](http://smserver.sti.ua.es/millbin/msdigest.cgi?missed_cleavages=2&msparams_dir=msparams_mill/&hide_protein_sequence=2&database=PA_haloferax_mediterranei_genbank&seqdb_dir=D:\SeqDB\&enzyme=Trypsin&access_method=Accession+Number&accession_num=AFK20246.1&coverage_map=0+4+9+253+9+11+24+179) | 9.77e+006 |
| transport ATPase (substrate arsenite) | [AFK18406.1](http://smserver.sti.ua.es/millhtml/SM_instruct/servadmn.htm#update_acclinks?AFK18406.1) | 4 | 4 | 65.24 | [12.3](http://smserver.sti.ua.es/millbin/msdigest.cgi?missed_cleavages=2&msparams_dir=msparams_mill/&hide_protein_sequence=2&database=PA_haloferax_mediterranei_genbank&seqdb_dir=D:\SeqDB\&enzyme=Trypsin&access_method=Accession+Number&accession_num=AFK18406.1&coverage_map=0+226+14+75+29+25+7+28) | 2.82e+006 |
| hypothetical protein HFX_2088 | [AFK19779.1](http://smserver.sti.ua.es/millhtml/SM_instruct/servadmn.htm#update_acclinks?AFK19779.1) | 4 | 4 | 65.09 | [13.7](http://smserver.sti.ua.es/millbin/msdigest.cgi?missed_cleavages=2&msparams_dir=msparams_mill/&hide_protein_sequence=2&database=PA_haloferax_mediterranei_genbank&seqdb_dir=D:\SeqDB\&enzyme=Trypsin&access_method=Accession+Number&accession_num=AFK19779.1&coverage_map=0+200+10+100+11+21+32+11) | 2.81e+006 |
| 2-oxoglutarate ferredoxin oxidoreductase, subunit alpha | [AFK18583.1](http://smserver.sti.ua.es/millhtml/SM_instruct/servadmn.htm#update_acclinks?AFK18583.1) | 4 | 4 | 64.41 | [7.8](http://smserver.sti.ua.es/millbin/msdigest.cgi?missed_cleavages=2&msparams_dir=msparams_mill/&hide_protein_sequence=2&database=PA_haloferax_mediterranei_genbank&seqdb_dir=D:\SeqDB\&enzyme=Trypsin&access_method=Accession+Number&accession_num=AFK18583.1&coverage_map=0+22+8+37+10+253+14+46+14+182) | 3.49e+006 |
| aspartyl-tRNA synthetase | [AFK18364.2](http://smserver.sti.ua.es/millhtml/SM_instruct/servadmn.htm#update_acclinks?AFK18364.2) | 5 | 5 | 64.21 | [15.4](http://smserver.sti.ua.es/millbin/msdigest.cgi?missed_cleavages=2&msparams_dir=msparams_mill/&hide_protein_sequence=2&database=PA_haloferax_mediterranei_genbank&seqdb_dir=D:\SeqDB\&enzyme=Trypsin&access_method=Accession+Number&accession_num=AFK18364.2&coverage_map=0+26+11+94+6+23+32+10+9+69+9+145) | 5.25e+006 |
| electron transfer flavoprotein alpha subunit | [AFK20418.1](http://smserver.sti.ua.es/millhtml/SM_instruct/servadmn.htm#update_acclinks?AFK20418.1) | 5 | 5 | 63.30 | [9.5](http://smserver.sti.ua.es/millbin/msdigest.cgi?missed_cleavages=2&msparams_dir=msparams_mill/&hide_protein_sequence=2&database=PA_haloferax_mediterranei_genbank&seqdb_dir=D:\SeqDB\&enzyme=Trypsin&access_method=Accession+Number&accession_num=AFK20418.1&coverage_map=0+19+17+4+10+150+12+99+8+223+10+42) | 4.79e+006 |
| putative hydrolase or acyltransferase of alpha/beta superfamily | [AFK19333.1](http://smserver.sti.ua.es/millhtml/SM_instruct/servadmn.htm#update_acclinks?AFK19333.1) | 4 | 4 | 62.24 | [18.7](http://smserver.sti.ua.es/millbin/msdigest.cgi?missed_cleavages=2&msparams_dir=msparams_mill/&hide_protein_sequence=2&database=PA_haloferax_mediterranei_genbank&seqdb_dir=D:\SeqDB\&enzyme=Trypsin&access_method=Accession+Number&accession_num=AFK19333.1&coverage_map=1+11+31+11+150+19+11+12+32) | 5.44e+006 |
| hypothetical protein HFX_0292 | [AFK18031.1](http://smserver.sti.ua.es/millhtml/SM_instruct/servadmn.htm#update_acclinks?AFK18031.1) | 4 | 4 | 61.00 | [12.1](http://smserver.sti.ua.es/millbin/msdigest.cgi?missed_cleavages=2&msparams_dir=msparams_mill/&hide_protein_sequence=2&database=PA_haloferax_mediterranei_genbank&seqdb_dir=D:\SeqDB\&enzyme=Trypsin&access_method=Accession+Number&accession_num=AFK18031.1&coverage_map=0+57+8+107+7+20+15+131+16+19) | 2.74e+006 |
| aldehyde dehydrogenase (NAD+) (plasmid) | [AFK21494.1](http://smserver.sti.ua.es/millhtml/SM_instruct/servadmn.htm#update_acclinks?AFK21494.1) | 4 | 4 | 60.56 | [13.4](http://smserver.sti.ua.es/millbin/msdigest.cgi?missed_cleavages=2&msparams_dir=msparams_mill/&hide_protein_sequence=2&database=PA_haloferax_mediterranei_genbank&seqdb_dir=D:\SeqDB\&enzyme=Trypsin&access_method=Accession+Number&accession_num=AFK21494.1&coverage_map=0+40+15+183+35+37+15+158) | 1.27e+006 |
| molybdate transport protein | [AFK19675.1](http://smserver.sti.ua.es/millhtml/SM_instruct/servadmn.htm#update_acclinks?AFK19675.1) | 4 | 4 | 60.19 | [14.5](http://smserver.sti.ua.es/millbin/msdigest.cgi?missed_cleavages=2&msparams_dir=msparams_mill/&hide_protein_sequence=2&database=PA_haloferax_mediterranei_genbank&seqdb_dir=D:\SeqDB\&enzyme=Trypsin&access_method=Accession+Number&accession_num=AFK19675.1&coverage_map=0+87+14+79+15+38+10+7+12+89) | 2.77e+006 |
| ABC-type glutamine/glutamate/polar amino acids transport system, substrate-binding protein | [AFK20126.1](http://smserver.sti.ua.es/millhtml/SM_instruct/servadmn.htm#update_acclinks?AFK20126.1) | 6 | 4 | 60.00 | [23](http://smserver.sti.ua.es/millbin/msdigest.cgi?missed_cleavages=2&msparams_dir=msparams_mill/&hide_protein_sequence=2&database=PA_haloferax_mediterranei_genbank&seqdb_dir=D:\SeqDB\&enzyme=Trypsin&access_method=Accession+Number&accession_num=AFK20126.1&coverage_map=0+71+12+40+35+64+10+15) | 7.55e+006 |
| hypothetical protein HFX_0195 | [AFK17936.1](http://smserver.sti.ua.es/millhtml/SM_instruct/servadmn.htm#update_acclinks?AFK17936.1) | 4 | 3 | 59.03 | [52.7](http://smserver.sti.ua.es/millbin/msdigest.cgi?missed_cleavages=2&msparams_dir=msparams_mill/&hide_protein_sequence=2&database=PA_haloferax_mediterranei_genbank&seqdb_dir=D:\SeqDB\&enzyme=Trypsin&access_method=Accession+Number&accession_num=AFK17936.1&coverage_map=0+11+29+15) | 1.86e+007 |
| phosphopyruvate hydratase | [AFK20460.1](http://smserver.sti.ua.es/millhtml/SM_instruct/servadmn.htm#update_acclinks?AFK20460.1) | 4 | 4 | 59.02 | [13.5](http://smserver.sti.ua.es/millbin/msdigest.cgi?missed_cleavages=2&msparams_dir=msparams_mill/&hide_protein_sequence=2&database=PA_haloferax_mediterranei_genbank&seqdb_dir=D:\SeqDB\&enzyme=Trypsin&access_method=Accession+Number&accession_num=AFK20460.1&coverage_map=0+16+18+33+11+32+19+258+6+6) | 2.58e+006 |
| branched-chain/neutral amino acids amide ABC transporter periplasmic substrate-binding protein | [AFK19529.1](http://smserver.sti.ua.es/millhtml/SM_instruct/servadmn.htm#update_acclinks?AFK19529.1) | 4 | 4 | 58.85 | [12.6](http://smserver.sti.ua.es/millbin/msdigest.cgi?missed_cleavages=2&msparams_dir=msparams_mill/&hide_protein_sequence=2&database=PA_haloferax_mediterranei_genbank&seqdb_dir=D:\SeqDB\&enzyme=Trypsin&access_method=Accession+Number&accession_num=AFK19529.1&coverage_map=0+69+10+185+28+50+16+68) | 1.06e+007 |
| Pyruvate dehydrogenase E1 component subunit beta | [AFK20617.1](http://smserver.sti.ua.es/millhtml/SM_instruct/servadmn.htm#update_acclinks?AFK20617.1) | 4 | 4 | 58.52 | [14.6](http://smserver.sti.ua.es/millbin/msdigest.cgi?missed_cleavages=2&msparams_dir=msparams_mill/&hide_protein_sequence=2&database=PA_haloferax_mediterranei_genbank&seqdb_dir=D:\SeqDB\&enzyme=Trypsin&access_method=Accession+Number&accession_num=AFK20617.1&coverage_map=0+135+30+18+18+126) | 3.84e+006 |
| regulatory protein PrrC | [AFK18961.1](http://smserver.sti.ua.es/millhtml/SM_instruct/servadmn.htm#update_acclinks?AFK18961.1) | 4 | 3 | 57.51 | [21.5](http://smserver.sti.ua.es/millbin/msdigest.cgi?missed_cleavages=2&msparams_dir=msparams_mill/&hide_protein_sequence=2&database=PA_haloferax_mediterranei_genbank&seqdb_dir=D:\SeqDB\&enzyme=Trypsin&access_method=Accession+Number&accession_num=AFK18961.1&coverage_map=0+134+18+8+13+31+17+2) | 1.07e+007 |
| aconitate hydratase | [AFK18238.1](http://smserver.sti.ua.es/millhtml/SM_instruct/servadmn.htm#update_acclinks?AFK18238.1) | 4 | 4 | 57.48 | [6.2](http://smserver.sti.ua.es/millbin/msdigest.cgi?missed_cleavages=2&msparams_dir=msparams_mill/&hide_protein_sequence=2&database=PA_haloferax_mediterranei_genbank&seqdb_dir=D:\SeqDB\&enzyme=Trypsin&access_method=Accession+Number&accession_num=AFK18238.1&coverage_map=0+44+8+33+19+149+23+539+8+103) | 4.26e+006 |
| hypothetical protein HFX_1448 | [AFK19156.1](http://smserver.sti.ua.es/millhtml/SM_instruct/servadmn.htm#update_acclinks?AFK19156.1) | 4 | 3 | 57.44 | [23.6](http://smserver.sti.ua.es/millbin/msdigest.cgi?missed_cleavages=2&msparams_dir=msparams_mill/&hide_protein_sequence=2&database=PA_haloferax_mediterranei_genbank&seqdb_dir=D:\SeqDB\&enzyme=Trypsin&access_method=Accession+Number&accession_num=AFK19156.1&coverage_map=1+53+167) | 7.50e+006 |
| preprotein translocase subunit SecD | [AFK19761.1](http://smserver.sti.ua.es/millhtml/SM_instruct/servadmn.htm#update_acclinks?AFK19761.1) | 4 | 4 | 57.28 | [8.2](http://smserver.sti.ua.es/millbin/msdigest.cgi?missed_cleavages=2&msparams_dir=msparams_mill/&hide_protein_sequence=2&database=PA_haloferax_mediterranei_genbank&seqdb_dir=D:\SeqDB\&enzyme=Trypsin&access_method=Accession+Number&accession_num=AFK19761.1&coverage_map=0+45+10+21+21+83+12+331) | 2.26e+006 |
| hypothetical protein HFX_1776 | [AFK19481.1](http://smserver.sti.ua.es/millhtml/SM_instruct/servadmn.htm#update_acclinks?AFK19481.1) | 4 | 4 | 57.20 | [8.4](http://smserver.sti.ua.es/millbin/msdigest.cgi?missed_cleavages=2&msparams_dir=msparams_mill/&hide_protein_sequence=2&database=PA_haloferax_mediterranei_genbank&seqdb_dir=D:\SeqDB\&enzyme=Trypsin&access_method=Accession+Number&accession_num=AFK19481.1&coverage_map=0+51+9+136+21+50+11+206) | 3.24e+006 |
| uracil phosphoribosyltransferase | [AFK20636.1](http://smserver.sti.ua.es/millhtml/SM_instruct/servadmn.htm#update_acclinks?AFK20636.1) | 4 | 4 | 56.80 | [28.7](http://smserver.sti.ua.es/millbin/msdigest.cgi?missed_cleavages=2&msparams_dir=msparams_mill/&hide_protein_sequence=2&database=PA_haloferax_mediterranei_genbank&seqdb_dir=D:\SeqDB\&enzyme=Trypsin&access_method=Accession+Number&accession_num=AFK20636.1&coverage_map=0+25+10+96+55+40) | 2.56e+006 |
| putative hydrolase of the metallo-beta-lactamase superfamily | [AFK20411.1](http://smserver.sti.ua.es/millhtml/SM_instruct/servadmn.htm#update_acclinks?AFK20411.1) | 4 | 4 | 55.79 | [10.6](http://smserver.sti.ua.es/millbin/msdigest.cgi?missed_cleavages=2&msparams_dir=msparams_mill/&hide_protein_sequence=2&database=PA_haloferax_mediterranei_genbank&seqdb_dir=D:\SeqDB\&enzyme=Trypsin&access_method=Accession+Number&accession_num=AFK20411.1&coverage_map=0+49+16+189+9+4+7+11+16+149) | 4.54e+006 |
| CBS domain-containing protein | [AFK18713.2](http://smserver.sti.ua.es/millhtml/SM_instruct/servadmn.htm#update_acclinks?AFK18713.2) | 5 | 4 | 55.56 | [14.5](http://smserver.sti.ua.es/millbin/msdigest.cgi?missed_cleavages=2&msparams_dir=msparams_mill/&hide_protein_sequence=2&database=PA_haloferax_mediterranei_genbank&seqdb_dir=D:\SeqDB\&enzyme=Trypsin&access_method=Accession+Number&accession_num=AFK18713.2&coverage_map=0+136+11+15+11+52+7+65+24+44) | 4.38e+006 |
| hypothetical protein HFX_1243 | [AFK18956.1](http://smserver.sti.ua.es/millhtml/SM_instruct/servadmn.htm#update_acclinks?AFK18956.1) | 4 | 3 | 55.36 | [10.6](http://smserver.sti.ua.es/millbin/msdigest.cgi?missed_cleavages=2&msparams_dir=msparams_mill/&hide_protein_sequence=2&database=PA_haloferax_mediterranei_genbank&seqdb_dir=D:\SeqDB\&enzyme=Trypsin&access_method=Accession+Number&accession_num=AFK18956.1&coverage_map=0+114+40+222) | 8.30e+006 |
| metalloprotease | [AFK18012.1](http://smserver.sti.ua.es/millhtml/SM_instruct/servadmn.htm#update_acclinks?AFK18012.1) | 4 | 4 | 55.33 | [14.1](http://smserver.sti.ua.es/millbin/msdigest.cgi?missed_cleavages=2&msparams_dir=msparams_mill/&hide_protein_sequence=2&database=PA_haloferax_mediterranei_genbank&seqdb_dir=D:\SeqDB\&enzyme=Trypsin&access_method=Accession+Number&accession_num=AFK18012.1&coverage_map=0+5+10+241+18+70+27+19) | 3.56e+006 |
| hypothetical protein HFX_2114 | [AFK19805.1](http://smserver.sti.ua.es/millhtml/SM_instruct/servadmn.htm#update_acclinks?AFK19805.1) | 3 | 3 | 55.09 | [12.8](http://smserver.sti.ua.es/millbin/msdigest.cgi?missed_cleavages=2&msparams_dir=msparams_mill/&hide_protein_sequence=2&database=PA_haloferax_mediterranei_genbank&seqdb_dir=D:\SeqDB\&enzyme=Trypsin&access_method=Accession+Number&accession_num=AFK19805.1&coverage_map=0+57+17+39+21+170+11+67) | 2.56e+006 |
| glutaryl-CoA dehydrogenase | [AFK17952.2](http://smserver.sti.ua.es/millhtml/SM_instruct/servadmn.htm#update_acclinks?AFK17952.2) | 4 | 4 | 54.82 | [13.1](http://smserver.sti.ua.es/millbin/msdigest.cgi?missed_cleavages=2&msparams_dir=msparams_mill/&hide_protein_sequence=2&database=PA_haloferax_mediterranei_genbank&seqdb_dir=D:\SeqDB\&enzyme=Trypsin&access_method=Accession+Number&accession_num=AFK17952.2&coverage_map=0+72+17+120+13+14+10+12+11+118) | 1.69e+006 |
| phosphate ABC transporter ATP-binding protein | [AFK20072.1](http://smserver.sti.ua.es/millhtml/SM_instruct/servadmn.htm#update_acclinks?AFK20072.1) | 4 | 4 | 54.15 | [12.5](http://smserver.sti.ua.es/millbin/msdigest.cgi?missed_cleavages=2&msparams_dir=msparams_mill/&hide_protein_sequence=2&database=PA_haloferax_mediterranei_genbank&seqdb_dir=D:\SeqDB\&enzyme=Trypsin&access_method=Accession+Number&accession_num=AFK20072.1&coverage_map=0+98+17+30+11+121+9+10) | 3.44e+006 |
| ubiquinone/menaquinone biosynthesis methyltransferase | [AFK18034.2](http://smserver.sti.ua.es/millhtml/SM_instruct/servadmn.htm#update_acclinks?AFK18034.2) | 3 | 3 | 53.94 | [16.3](http://smserver.sti.ua.es/millbin/msdigest.cgi?missed_cleavages=2&msparams_dir=msparams_mill/&hide_protein_sequence=2&database=PA_haloferax_mediterranei_genbank&seqdb_dir=D:\SeqDB\&enzyme=Trypsin&access_method=Accession+Number&accession_num=AFK18034.2&coverage_map=0+48+17+76+10+45+7+5) | 6.87e+006 |
| ABC-type transport system ATP-binding protein | [AFK20086.1](http://smserver.sti.ua.es/millhtml/SM_instruct/servadmn.htm#update_acclinks?AFK20086.1) | 4 | 3 | 53.27 | [16.5](http://smserver.sti.ua.es/millbin/msdigest.cgi?missed_cleavages=2&msparams_dir=msparams_mill/&hide_protein_sequence=2&database=PA_haloferax_mediterranei_genbank&seqdb_dir=D:\SeqDB\&enzyme=Trypsin&access_method=Accession+Number&accession_num=AFK20086.1&coverage_map=0+70+21+48+17+73+6+31) | 4.49e+006 |
| 3-hydroxyacyl-CoA dehydrogenase | [AFK20502.1](http://smserver.sti.ua.es/millhtml/SM_instruct/servadmn.htm#update_acclinks?AFK20502.1) | 4 | 4 | 53.07 | [6.8](http://smserver.sti.ua.es/millbin/msdigest.cgi?missed_cleavages=2&msparams_dir=msparams_mill/&hide_protein_sequence=2&database=PA_haloferax_mediterranei_genbank&seqdb_dir=D:\SeqDB\&enzyme=Trypsin&access_method=Accession+Number&accession_num=AFK20502.1&coverage_map=0+44+11+6+12+258+14+25+8+280) | 1.45e+006 |
| molecular chaperone DnaK | [AFK19355.1](http://smserver.sti.ua.es/millhtml/SM_instruct/servadmn.htm#update_acclinks?AFK19355.1) | 4 | 4 | 52.60 | [9.7](http://smserver.sti.ua.es/millbin/msdigest.cgi?missed_cleavages=2&msparams_dir=msparams_mill/&hide_protein_sequence=2&database=PA_haloferax_mediterranei_genbank&seqdb_dir=D:\SeqDB\&enzyme=Trypsin&access_method=Accession+Number&accession_num=AFK19355.1&coverage_map=0+104+24+6+12+105+25+349) | 2.50e+006 |
| phosphonates ABC transporter permease protein | [AFK19844.1](http://smserver.sti.ua.es/millhtml/SM_instruct/servadmn.htm#update_acclinks?AFK19844.1) | 5 | 4 | 52.31 | [14.9](http://smserver.sti.ua.es/millbin/msdigest.cgi?missed_cleavages=2&msparams_dir=msparams_mill/&hide_protein_sequence=2&database=PA_haloferax_mediterranei_genbank&seqdb_dir=D:\SeqDB\&enzyme=Trypsin&access_method=Accession+Number&accession_num=AFK19844.1&coverage_map=0+53+8+49+7+36+25+90) | 3.19e+006 |
| enoyl-CoA hydratase | [AFK19171.2](http://smserver.sti.ua.es/millhtml/SM_instruct/servadmn.htm#update_acclinks?AFK19171.2) | 4 | 4 | 52.25 | [14.3](http://smserver.sti.ua.es/millbin/msdigest.cgi?missed_cleavages=2&msparams_dir=msparams_mill/&hide_protein_sequence=2&database=PA_haloferax_mediterranei_genbank&seqdb_dir=D:\SeqDB\&enzyme=Trypsin&access_method=Accession+Number&accession_num=AFK19171.2&coverage_map=0+49+10+88+11+1+7+55+9+27) | 3.85e+006 |
| putative iron transport protein | [AFK19393.1](http://smserver.sti.ua.es/millhtml/SM_instruct/servadmn.htm#update_acclinks?AFK19393.1) | 4 | 4 | 51.71 | [10.3](http://smserver.sti.ua.es/millbin/msdigest.cgi?missed_cleavages=2&msparams_dir=msparams_mill/&hide_protein_sequence=2&database=PA_haloferax_mediterranei_genbank&seqdb_dir=D:\SeqDB\&enzyme=Trypsin&access_method=Accession+Number&accession_num=AFK19393.1&coverage_map=0+131+15+53+10+129+7+49+10) | 3.65e+006 |
| methylmalonyl-CoA decarboxylase alpha chain | [AFK20161.1](http://smserver.sti.ua.es/millhtml/SM_instruct/servadmn.htm#update_acclinks?AFK20161.1) | 4 | 4 | 51.71 | [8.9](http://smserver.sti.ua.es/millbin/msdigest.cgi?missed_cleavages=2&msparams_dir=msparams_mill/&hide_protein_sequence=2&database=PA_haloferax_mediterranei_genbank&seqdb_dir=D:\SeqDB\&enzyme=Trypsin&access_method=Accession+Number&accession_num=AFK20161.1&coverage_map=0+266+14+49+13+134+13+2+6+19) | 3.14e+006 |
| putative sugar ABC transporter permease protein | [AFK20384.1](http://smserver.sti.ua.es/millhtml/SM_instruct/servadmn.htm#update_acclinks?AFK20384.1) | 3 | 3 | 51.47 | [10.9](http://smserver.sti.ua.es/millbin/msdigest.cgi?missed_cleavages=2&msparams_dir=msparams_mill/&hide_protein_sequence=2&database=PA_haloferax_mediterranei_genbank&seqdb_dir=D:\SeqDB\&enzyme=Trypsin&access_method=Accession+Number&accession_num=AFK20384.1&coverage_map=0+68+23+216+12) | 7.19e+006 |
| 3-ketoacyl-acyl carrier protein reductase (PhaB) (plasmid) | [AFK21048.1](http://smserver.sti.ua.es/millhtml/SM_instruct/servadmn.htm#update_acclinks?AFK21048.1) | 3 | 3 | 50.80 | [19.7](http://smserver.sti.ua.es/millbin/msdigest.cgi?missed_cleavages=2&msparams_dir=msparams_mill/&hide_protein_sequence=2&database=PA_haloferax_mediterranei_genbank&seqdb_dir=D:\SeqDB\&enzyme=Trypsin&access_method=Accession+Number&accession_num=AFK21048.1&coverage_map=0+110+14+13+23+53+12+23) | 2.89e+006 |
| hypothetical protein HFX_0697 | [AFK18420.1](http://smserver.sti.ua.es/millhtml/SM_instruct/servadmn.htm#update_acclinks?AFK18420.1) | 5 | 3 | 50.68 | [45.1](http://smserver.sti.ua.es/millbin/msdigest.cgi?missed_cleavages=2&msparams_dir=msparams_mill/&hide_protein_sequence=2&database=PA_haloferax_mediterranei_genbank&seqdb_dir=D:\SeqDB\&enzyme=Trypsin&access_method=Accession+Number&accession_num=AFK18420.1&coverage_map=0+38+6+7+36+6) | 1.87e+007 |
| gas-vesicle operon protein gvpH | [AFK19407.1](http://smserver.sti.ua.es/millhtml/SM_instruct/servadmn.htm#update_acclinks?AFK19407.1) | 4 | 3 | 50.64 | [23.1](http://smserver.sti.ua.es/millbin/msdigest.cgi?missed_cleavages=2&msparams_dir=msparams_mill/&hide_protein_sequence=2&database=PA_haloferax_mediterranei_genbank&seqdb_dir=D:\SeqDB\&enzyme=Trypsin&access_method=Accession+Number&accession_num=AFK19407.1&coverage_map=0+144+50+22) | 2.89e+006 |
| hypothetical protein HFX_6434 (plasmid) | [AFK21553.1](http://smserver.sti.ua.es/millhtml/SM_instruct/servadmn.htm#update_acclinks?AFK21553.1) | 4 | 3 | 49.62 | [10.3](http://smserver.sti.ua.es/millbin/msdigest.cgi?missed_cleavages=2&msparams_dir=msparams_mill/&hide_protein_sequence=2&database=PA_haloferax_mediterranei_genbank&seqdb_dir=D:\SeqDB\&enzyme=Trypsin&access_method=Accession+Number&accession_num=AFK21553.1&coverage_map=0+69+10+14+9+181+18+58) | 1.12e+007 |
| cellulase, endo-1,3(4)-beta-glucanase, peptidase M42 family protein / endoglucanase | [AFK20447.1](http://smserver.sti.ua.es/millhtml/SM_instruct/servadmn.htm#update_acclinks?AFK20447.1) | 3 | 3 | 49.44 | [9.3](http://smserver.sti.ua.es/millbin/msdigest.cgi?missed_cleavages=2&msparams_dir=msparams_mill/&hide_protein_sequence=2&database=PA_haloferax_mediterranei_genbank&seqdb_dir=D:\SeqDB\&enzyme=Trypsin&access_method=Accession+Number&accession_num=AFK20447.1&coverage_map=0+84+9+86+11+57+13+94) | 1.36e+006 |
| proteasome beta subunit | [AFK19329.1](http://smserver.sti.ua.es/millhtml/SM_instruct/servadmn.htm#update_acclinks?AFK19329.1) | 4 | 3 | 49.08 | [17.1](http://smserver.sti.ua.es/millbin/msdigest.cgi?missed_cleavages=2&msparams_dir=msparams_mill/&hide_protein_sequence=2&database=PA_haloferax_mediterranei_genbank&seqdb_dir=D:\SeqDB\&enzyme=Trypsin&access_method=Accession+Number&accession_num=AFK19329.1&coverage_map=0+56+12+21+20+126+10) | 5.73e+006 |
| NADH dehydrogenase, subunit H (ubiquinone) | [AFK18697.1](http://smserver.sti.ua.es/millhtml/SM_instruct/servadmn.htm#update_acclinks?AFK18697.1) | 4 | 3 | 48.67 | [9.4](http://smserver.sti.ua.es/millbin/msdigest.cgi?missed_cleavages=2&msparams_dir=msparams_mill/&hide_protein_sequence=2&database=PA_haloferax_mediterranei_genbank&seqdb_dir=D:\SeqDB\&enzyme=Trypsin&access_method=Accession+Number&accession_num=AFK18697.1&coverage_map=0+52+8+5+15+238+10+21) | 2.58e+007 |
| anthranilate phosphoribosyltransferase | [AFK19965.1](http://smserver.sti.ua.es/millhtml/SM_instruct/servadmn.htm#update_acclinks?AFK19965.1) | 3 | 3 | 47.98 | [13.2](http://smserver.sti.ua.es/millbin/msdigest.cgi?missed_cleavages=2&msparams_dir=msparams_mill/&hide_protein_sequence=2&database=PA_haloferax_mediterranei_genbank&seqdb_dir=D:\SeqDB\&enzyme=Trypsin&access_method=Accession+Number&accession_num=AFK19965.1&coverage_map=0+42+19+246+12+18+16+2) | 1.08e+006 |
| S-adenosylmethionine synthetase | [AFK19445.1](http://smserver.sti.ua.es/millhtml/SM_instruct/servadmn.htm#update_acclinks?AFK19445.1) | 3 | 3 | 47.65 | [8.7](http://smserver.sti.ua.es/millbin/msdigest.cgi?missed_cleavages=2&msparams_dir=msparams_mill/&hide_protein_sequence=2&database=PA_haloferax_mediterranei_genbank&seqdb_dir=D:\SeqDB\&enzyme=Trypsin&access_method=Accession+Number&accession_num=AFK19445.1&coverage_map=0+14+12+211+13+132+10+10) | 1.14e+006 |
| hypothetical protein HFX_6075 (plasmid) | [AFK21202.1](http://smserver.sti.ua.es/millhtml/SM_instruct/servadmn.htm#update_acclinks?AFK21202.1) | 3 | 3 | 47.52 | [33.3](http://smserver.sti.ua.es/millbin/msdigest.cgi?missed_cleavages=2&msparams_dir=msparams_mill/&hide_protein_sequence=2&database=PA_haloferax_mediterranei_genbank&seqdb_dir=D:\SeqDB\&enzyme=Trypsin&access_method=Accession+Number&accession_num=AFK21202.1&coverage_map=0+2+15+24+13+30) | 4.90e+006 |
| hypothetical protein HFX_1178 | [AFK18891.1](http://smserver.sti.ua.es/millhtml/SM_instruct/servadmn.htm#update_acclinks?AFK18891.1) | 4 | 3 | 47.15 | [58.5](http://smserver.sti.ua.es/millbin/msdigest.cgi?missed_cleavages=2&msparams_dir=msparams_mill/&hide_protein_sequence=2&database=PA_haloferax_mediterranei_genbank&seqdb_dir=D:\SeqDB\&enzyme=Trypsin&access_method=Accession+Number&accession_num=AFK18891.1&coverage_map=0+21+21+5+27+8) | 1.52e+006 |
| hypothetical protein HFX_5167 (plasmid) | [AFK21001.1](http://smserver.sti.ua.es/millhtml/SM_instruct/servadmn.htm#update_acclinks?AFK21001.1) | 3 | 3 | 46.53 | [16](http://smserver.sti.ua.es/millbin/msdigest.cgi?missed_cleavages=2&msparams_dir=msparams_mill/&hide_protein_sequence=2&database=PA_haloferax_mediterranei_genbank&seqdb_dir=D:\SeqDB\&enzyme=Trypsin&access_method=Accession+Number&accession_num=AFK21001.1&coverage_map=0+83+13+44+25+92+6+12) | 1.04e+006 |
| hypothetical protein HFX_2040 | [AFK19732.2](http://smserver.sti.ua.es/millhtml/SM_instruct/servadmn.htm#update_acclinks?AFK19732.2) | 3 | 3 | 45.83 | [10.2](http://smserver.sti.ua.es/millbin/msdigest.cgi?missed_cleavages=2&msparams_dir=msparams_mill/&hide_protein_sequence=2&database=PA_haloferax_mediterranei_genbank&seqdb_dir=D:\SeqDB\&enzyme=Trypsin&access_method=Accession+Number&accession_num=AFK19732.2&coverage_map=0+107+12+47+14+2+9+150) | 7.15e+005 |
| hypothetical protein HFX_2772 | [AFK20448.1](http://smserver.sti.ua.es/millhtml/SM_instruct/servadmn.htm#update_acclinks?AFK20448.1) | 5 | 3 | 45.61 | [26.4](http://smserver.sti.ua.es/millbin/msdigest.cgi?missed_cleavages=2&msparams_dir=msparams_mill/&hide_protein_sequence=2&database=PA_haloferax_mediterranei_genbank&seqdb_dir=D:\SeqDB\&enzyme=Trypsin&access_method=Accession+Number&accession_num=AFK20448.1&coverage_map=0+22+6+53+21) | 1.98e+007 |
| ABC-type cobalamin/Iron(III)-siderophore transport systems, substrate-binding protein | [AFK18834.1](http://smserver.sti.ua.es/millhtml/SM_instruct/servadmn.htm#update_acclinks?AFK18834.1) | 3 | 3 | 45.24 | [11.2](http://smserver.sti.ua.es/millbin/msdigest.cgi?missed_cleavages=2&msparams_dir=msparams_mill/&hide_protein_sequence=2&database=PA_haloferax_mediterranei_genbank&seqdb_dir=D:\SeqDB\&enzyme=Trypsin&access_method=Accession+Number&accession_num=AFK18834.1&coverage_map=0+54+18+131+14+66+10+79) | 2.31e+006 |
| Ca2+-transporting ATPase | [AFK18654.1](http://smserver.sti.ua.es/millhtml/SM_instruct/servadmn.htm#update_acclinks?AFK18654.1) | 3 | 3 | 45.14 | [3.8](http://smserver.sti.ua.es/millbin/msdigest.cgi?missed_cleavages=2&msparams_dir=msparams_mill/&hide_protein_sequence=2&database=PA_haloferax_mediterranei_genbank&seqdb_dir=D:\SeqDB\&enzyme=Trypsin&access_method=Accession+Number&accession_num=AFK18654.1&coverage_map=0+235+10+208+9+3+15+412) | 1.71e+006 |
| hypothetical protein HFX_0671 | [AFK18394.1](http://smserver.sti.ua.es/millhtml/SM_instruct/servadmn.htm#update_acclinks?AFK18394.1) | 4 | 3 | 45.12 | [20.4](http://smserver.sti.ua.es/millbin/msdigest.cgi?missed_cleavages=2&msparams_dir=msparams_mill/&hide_protein_sequence=2&database=PA_haloferax_mediterranei_genbank&seqdb_dir=D:\SeqDB\&enzyme=Trypsin&access_method=Accession+Number&accession_num=AFK18394.1&coverage_map=0+12+14+179+38+11) | 4.52e+006 |
| nitrite reductase (NO-forming) | [AFK19872.1](http://smserver.sti.ua.es/millhtml/SM_instruct/servadmn.htm#update_acclinks?AFK19872.1) | 4 | 3 | 43.76 | [10.8](http://smserver.sti.ua.es/millbin/msdigest.cgi?missed_cleavages=2&msparams_dir=msparams_mill/&hide_protein_sequence=2&database=PA_haloferax_mediterranei_genbank&seqdb_dir=D:\SeqDB\&enzyme=Trypsin&access_method=Accession+Number&accession_num=AFK19872.1&coverage_map=0+50+18+182+13+62+8+26) | 2.24e+006 |
| sugar phosphotransferase system (PTS) IIB component | [AFK19266.1](http://smserver.sti.ua.es/millhtml/SM_instruct/servadmn.htm#update_acclinks?AFK19266.1) | 3 | 3 | 43.30 | [29.8](http://smserver.sti.ua.es/millbin/msdigest.cgi?missed_cleavages=2&msparams_dir=msparams_mill/&hide_protein_sequence=2&database=PA_haloferax_mediterranei_genbank&seqdb_dir=D:\SeqDB\&enzyme=Trypsin&access_method=Accession+Number&accession_num=AFK19266.1&coverage_map=0+2+27+54+21+57) | 1.79e+006 |
| hypothetical protein HFX_1804 | [AFK19508.1](http://smserver.sti.ua.es/millhtml/SM_instruct/servadmn.htm#update_acclinks?AFK19508.1) | 3 | 3 | 43.23 | [20.7](http://smserver.sti.ua.es/millbin/msdigest.cgi?missed_cleavages=2&msparams_dir=msparams_mill/&hide_protein_sequence=2&database=PA_haloferax_mediterranei_genbank&seqdb_dir=D:\SeqDB\&enzyme=Trypsin&access_method=Accession+Number&accession_num=AFK19508.1&coverage_map=0+9+7+12+15+67+11+38) | 2.64e+006 |
| glycosyltransferase | [AFK19379.1](http://smserver.sti.ua.es/millhtml/SM_instruct/servadmn.htm#update_acclinks?AFK19379.1) | 3 | 3 | 43.08 | [15.7](http://smserver.sti.ua.es/millbin/msdigest.cgi?missed_cleavages=2&msparams_dir=msparams_mill/&hide_protein_sequence=2&database=PA_haloferax_mediterranei_genbank&seqdb_dir=D:\SeqDB\&enzyme=Trypsin&access_method=Accession+Number&accession_num=AFK19379.1&coverage_map=0+100+16+13+8+66+12+13) | 5.86e+006 |
| purK operon protein / membrane-bound mannosyltransferase | [AFK18691.1](http://smserver.sti.ua.es/millhtml/SM_instruct/servadmn.htm#update_acclinks?AFK18691.1) | 3 | 3 | 42.15 | [6.3](http://smserver.sti.ua.es/millbin/msdigest.cgi?missed_cleavages=2&msparams_dir=msparams_mill/&hide_protein_sequence=2&database=PA_haloferax_mediterranei_genbank&seqdb_dir=D:\SeqDB\&enzyme=Trypsin&access_method=Accession+Number&accession_num=AFK18691.1&coverage_map=0+47+7+108+18+33+12+359) | 1.26e+006 |
| NADH dehydrogenase-like complex, subunit I | [AFK18698.1](http://smserver.sti.ua.es/millhtml/SM_instruct/servadmn.htm#update_acclinks?AFK18698.1) | 3 | 3 | 42.06 | [16.3](http://smserver.sti.ua.es/millbin/msdigest.cgi?missed_cleavages=2&msparams_dir=msparams_mill/&hide_protein_sequence=2&database=PA_haloferax_mediterranei_genbank&seqdb_dir=D:\SeqDB\&enzyme=Trypsin&access_method=Accession+Number&accession_num=AFK18698.1&coverage_map=0+41+5+75+6+12+14) | 3.25e+006 |
| putative ABC transporter permease protein | [AFK20385.1](http://smserver.sti.ua.es/millhtml/SM_instruct/servadmn.htm#update_acclinks?AFK20385.1) | 2 | 2 | 41.96 | [7.3](http://smserver.sti.ua.es/millbin/msdigest.cgi?missed_cleavages=2&msparams_dir=msparams_mill/&hide_protein_sequence=2&database=PA_haloferax_mediterranei_genbank&seqdb_dir=D:\SeqDB\&enzyme=Trypsin&access_method=Accession+Number&accession_num=AFK20385.1&coverage_map=0+73+18+148+7+95) | 5.20e+006 |
| acyl-CoA synthetase | [AFK19702.1](http://smserver.sti.ua.es/millhtml/SM_instruct/servadmn.htm#update_acclinks?AFK19702.1) | 3 | 3 | 41.90 | [5.9](http://smserver.sti.ua.es/millbin/msdigest.cgi?missed_cleavages=2&msparams_dir=msparams_mill/&hide_protein_sequence=2&database=PA_haloferax_mediterranei_genbank&seqdb_dir=D:\SeqDB\&enzyme=Trypsin&access_method=Accession+Number&accession_num=AFK19702.1&coverage_map=0+150+11+265+9+8+20+204) | 1.65e+006 |
| hypothetical protein HFX_2509 | [AFK20191.2](http://smserver.sti.ua.es/millhtml/SM_instruct/servadmn.htm#update_acclinks?AFK20191.2) | 3 | 3 | 41.51 | [7.8](http://smserver.sti.ua.es/millbin/msdigest.cgi?missed_cleavages=2&msparams_dir=msparams_mill/&hide_protein_sequence=2&database=PA_haloferax_mediterranei_genbank&seqdb_dir=D:\SeqDB\&enzyme=Trypsin&access_method=Accession+Number&accession_num=AFK20191.2&coverage_map=0+19+9+37+8+98+7+127) | 3.43e+006 |
| superoxide dismutase, Fe-Mn family | [AFK20579.1](http://smserver.sti.ua.es/millhtml/SM_instruct/servadmn.htm#update_acclinks?AFK20579.1) | 3 | 3 | 41.45 | [16](http://smserver.sti.ua.es/millbin/msdigest.cgi?missed_cleavages=2&msparams_dir=msparams_mill/&hide_protein_sequence=2&database=PA_haloferax_mediterranei_genbank&seqdb_dir=D:\SeqDB\&enzyme=Trypsin&access_method=Accession+Number&accession_num=AFK20579.1&coverage_map=0+50+13+34+12+81+7+2) | 1.46e+006 |
| superoxide dismutase, Fe-Mn family (plasmid) | [AFK21528.1](http://smserver.sti.ua.es/millhtml/SM_instruct/servadmn.htm#update_acclinks?AFK21528.1) | 2 | 2 | 24.76 | [10](http://smserver.sti.ua.es/millbin/msdigest.cgi?missed_cleavages=2&msparams_dir=msparams_mill/&hide_protein_sequence=2&database=PA_haloferax_mediterranei_genbank&seqdb_dir=D:\SeqDB\&enzyme=Trypsin&access_method=Accession+Number&accession_num=AFK21528.1&coverage_map=0+51+13+127+7+2) | 1.04e+006 |
| propionyl-CoA carboxylase carboxyltransferase component | [AFK19220.2](http://smserver.sti.ua.es/millhtml/SM_instruct/servadmn.htm#update_acclinks?AFK19220.2) | 3 | 3 | 40.79 | [5.1](http://smserver.sti.ua.es/millbin/msdigest.cgi?missed_cleavages=2&msparams_dir=msparams_mill/&hide_protein_sequence=2&database=PA_haloferax_mediterranei_genbank&seqdb_dir=D:\SeqDB\&enzyme=Trypsin&access_method=Accession+Number&accession_num=AFK19220.2&coverage_map=0+372+8+68+12+50+10+62) | 1.02e+006 |
| universal stress protein UspA-like protein | [AFK19887.1](http://smserver.sti.ua.es/millhtml/SM_instruct/servadmn.htm#update_acclinks?AFK19887.1) | 3 | 3 | 40.74 | [9.5](http://smserver.sti.ua.es/millbin/msdigest.cgi?missed_cleavages=2&msparams_dir=msparams_mill/&hide_protein_sequence=2&database=PA_haloferax_mediterranei_genbank&seqdb_dir=D:\SeqDB\&enzyme=Trypsin&access_method=Accession+Number&accession_num=AFK19887.1&coverage_map=0+63+8+45+9+71+11+86) | 3.27e+006 |
| sec-independent protein translocase component TatC2 | [AFK17925.1](http://smserver.sti.ua.es/millhtml/SM_instruct/servadmn.htm#update_acclinks?AFK17925.1) | 3 | 3 | 40.72 | [5.2](http://smserver.sti.ua.es/millbin/msdigest.cgi?missed_cleavages=2&msparams_dir=msparams_mill/&hide_protein_sequence=2&database=PA_haloferax_mediterranei_genbank&seqdb_dir=D:\SeqDB\&enzyme=Trypsin&access_method=Accession+Number&accession_num=AFK17925.1&coverage_map=0+301+14+49+12+88+12+241) | 1.18e+006 |
| acyl-CoA synthetase (plasmid) | [AFK20965.1](http://smserver.sti.ua.es/millhtml/SM_instruct/servadmn.htm#update_acclinks?AFK20965.1) | 3 | 3 | 40.35 | [4.5](http://smserver.sti.ua.es/millbin/msdigest.cgi?missed_cleavages=2&msparams_dir=msparams_mill/&hide_protein_sequence=2&database=PA_haloferax_mediterranei_genbank&seqdb_dir=D:\SeqDB\&enzyme=Trypsin&access_method=Accession+Number&accession_num=AFK20965.1&coverage_map=0+120+10+96+11+374+9+44) | 6.85e+005 |
| N-methylhydantoinase B (ATP-hydrolyzing) (plasmid) | [AFK21025.1](http://smserver.sti.ua.es/millhtml/SM_instruct/servadmn.htm#update_acclinks?AFK21025.1) | 3 | 3 | 39.75 | [5.5](http://smserver.sti.ua.es/millbin/msdigest.cgi?missed_cleavages=2&msparams_dir=msparams_mill/&hide_protein_sequence=2&database=PA_haloferax_mediterranei_genbank&seqdb_dir=D:\SeqDB\&enzyme=Trypsin&access_method=Accession+Number&accession_num=AFK21025.1&coverage_map=0+181+13+235+7+20+13+124) | 1.15e+006 |
| NADH dehydrogenase, subunit M (ubiquinone) | [AFK18703.1](http://smserver.sti.ua.es/millhtml/SM_instruct/servadmn.htm#update_acclinks?AFK18703.1) | 3 | 3 | 39.66 | [5.5](http://smserver.sti.ua.es/millbin/msdigest.cgi?missed_cleavages=2&msparams_dir=msparams_mill/&hide_protein_sequence=2&database=PA_haloferax_mediterranei_genbank&seqdb_dir=D:\SeqDB\&enzyme=Trypsin&access_method=Accession+Number&accession_num=AFK18703.1&coverage_map=0+267+19+170+9+44) | 8.30e+006 |
| hypothetical protein HFX_0728 | [AFK18451.1](http://smserver.sti.ua.es/millhtml/SM_instruct/servadmn.htm#update_acclinks?AFK18451.1) | 3 | 3 | 39.19 | [19.4](http://smserver.sti.ua.es/millbin/msdigest.cgi?missed_cleavages=2&msparams_dir=msparams_mill/&hide_protein_sequence=2&database=PA_haloferax_mediterranei_genbank&seqdb_dir=D:\SeqDB\&enzyme=Trypsin&access_method=Accession+Number&accession_num=AFK18451.1&coverage_map=0+6+10+5+7+51+9+46) | 1.28e+006 |
| putative membrane-associated Zn-dependent protease | [AFK19648.2](http://smserver.sti.ua.es/millhtml/SM_instruct/servadmn.htm#update_acclinks?AFK19648.2) | 3 | 3 | 39.10 | [5](http://smserver.sti.ua.es/millbin/msdigest.cgi?missed_cleavages=2&msparams_dir=msparams_mill/&hide_protein_sequence=2&database=PA_haloferax_mediterranei_genbank&seqdb_dir=D:\SeqDB\&enzyme=Trypsin&access_method=Accession+Number&accession_num=AFK19648.2&coverage_map=0+329+8+36+13+165+9+37) | 9.71e+005 |
| putative signal peptide peptidase SppA | [AFK18576.1](http://smserver.sti.ua.es/millhtml/SM_instruct/servadmn.htm#update_acclinks?AFK18576.1) | 2 | 2 | 39.05 | [15.6](http://smserver.sti.ua.es/millbin/msdigest.cgi?missed_cleavages=2&msparams_dir=msparams_mill/&hide_protein_sequence=2&database=PA_haloferax_mediterranei_genbank&seqdb_dir=D:\SeqDB\&enzyme=Trypsin&access_method=Accession+Number&accession_num=AFK18576.1&coverage_map=0+90+28+161+24+30) | 1.70e+006 |
| putative phosphate acetyltransferase | [AFK18715.1](http://smserver.sti.ua.es/millhtml/SM_instruct/servadmn.htm#update_acclinks?AFK18715.1) | 3 | 3 | 38.88 | [9](http://smserver.sti.ua.es/millbin/msdigest.cgi?missed_cleavages=2&msparams_dir=msparams_mill/&hide_protein_sequence=2&database=PA_haloferax_mediterranei_genbank&seqdb_dir=D:\SeqDB\&enzyme=Trypsin&access_method=Accession+Number&accession_num=AFK18715.1&coverage_map=0+15+12+140+8+62+13+114) | 2.39e+006 |
| protein-disulfide isomerase | [AFK18965.1](http://smserver.sti.ua.es/millhtml/SM_instruct/servadmn.htm#update_acclinks?AFK18965.1) | 4 | 2 | 38.69 | [13.8](http://smserver.sti.ua.es/millbin/msdigest.cgi?missed_cleavages=2&msparams_dir=msparams_mill/&hide_protein_sequence=2&database=PA_haloferax_mediterranei_genbank&seqdb_dir=D:\SeqDB\&enzyme=Trypsin&access_method=Accession+Number&accession_num=AFK18965.1&coverage_map=0+99+14+59+19+48) | 1.89e+006 |
| hypothetical protein HFX_2876 | [AFK20546.1](http://smserver.sti.ua.es/millhtml/SM_instruct/servadmn.htm#update_acclinks?AFK20546.1) | 3 | 2 | 38.26 | [11.2](http://smserver.sti.ua.es/millbin/msdigest.cgi?missed_cleavages=2&msparams_dir=msparams_mill/&hide_protein_sequence=2&database=PA_haloferax_mediterranei_genbank&seqdb_dir=D:\SeqDB\&enzyme=Trypsin&access_method=Accession+Number&accession_num=AFK20546.1&coverage_map=1+10+135+18+77) | 1.85e+007 |
| RecJ-like exonuclease | [AFK20553.1](http://smserver.sti.ua.es/millhtml/SM_instruct/servadmn.htm#update_acclinks?AFK20553.1) | 3 | 3 | 38.24 | [4](http://smserver.sti.ua.es/millbin/msdigest.cgi?missed_cleavages=2&msparams_dir=msparams_mill/&hide_protein_sequence=2&database=PA_haloferax_mediterranei_genbank&seqdb_dir=D:\SeqDB\&enzyme=Trypsin&access_method=Accession+Number&accession_num=AFK20553.1&coverage_map=0+351+10+28+7+288+12+26) | 7.59e+005 |
| dihydroorotate dehydrogenase 2 | [AFK20600.1](http://smserver.sti.ua.es/millhtml/SM_instruct/servadmn.htm#update_acclinks?AFK20600.1) | 2 | 2 | 38.24 | [12](http://smserver.sti.ua.es/millbin/msdigest.cgi?missed_cleavages=2&msparams_dir=msparams_mill/&hide_protein_sequence=2&database=PA_haloferax_mediterranei_genbank&seqdb_dir=D:\SeqDB\&enzyme=Trypsin&access_method=Accession+Number&accession_num=AFK20600.1&coverage_map=0+55+22+122+21+137) | 1.54e+006 |
| hypothetical protein HFX_1273 | [AFK18986.1](http://smserver.sti.ua.es/millhtml/SM_instruct/servadmn.htm#update_acclinks?AFK18986.1) | 2 | 2 | 38.05 | [12.1](http://smserver.sti.ua.es/millbin/msdigest.cgi?missed_cleavages=2&msparams_dir=msparams_mill/&hide_protein_sequence=2&database=PA_haloferax_mediterranei_genbank&seqdb_dir=D:\SeqDB\&enzyme=Trypsin&access_method=Accession+Number&accession_num=AFK18986.1&coverage_map=0+55+13+82+12+43) | 5.34e+006 |
| phytoene dehydrogenase (phytoene desaturase) | [AFK18509.1](http://smserver.sti.ua.es/millhtml/SM_instruct/servadmn.htm#update_acclinks?AFK18509.1) | 3 | 3 | 37.90 | [7.7](http://smserver.sti.ua.es/millbin/msdigest.cgi?missed_cleavages=2&msparams_dir=msparams_mill/&hide_protein_sequence=2&database=PA_haloferax_mediterranei_genbank&seqdb_dir=D:\SeqDB\&enzyme=Trypsin&access_method=Accession+Number&accession_num=AFK18509.1&coverage_map=0+54+13+25+8+161+12+153) | 1.16e+006 |
| ferredoxin:NAD+ oxidoreductase | [AFK20041.1](http://smserver.sti.ua.es/millhtml/SM_instruct/servadmn.htm#update_acclinks?AFK20041.1) | 2 | 2 | 37.71 | [7.5](http://smserver.sti.ua.es/millbin/msdigest.cgi?missed_cleavages=2&msparams_dir=msparams_mill/&hide_protein_sequence=2&database=PA_haloferax_mediterranei_genbank&seqdb_dir=D:\SeqDB\&enzyme=Trypsin&access_method=Accession+Number&accession_num=AFK20041.1&coverage_map=0+180+16+167+15+35) | 1.60e+006 |
| acyl-CoA synthetase | [AFK19349.1](http://smserver.sti.ua.es/millhtml/SM_instruct/servadmn.htm#update_acclinks?AFK19349.1) | 3 | 3 | 37.48 | [4.2](http://smserver.sti.ua.es/millbin/msdigest.cgi?missed_cleavages=2&msparams_dir=msparams_mill/&hide_protein_sequence=2&database=PA_haloferax_mediterranei_genbank&seqdb_dir=D:\SeqDB\&enzyme=Trypsin&access_method=Accession+Number&accession_num=AFK19349.1&coverage_map=0+371+12+99+6+48+10+118) | 9.61e+005 |
| sialidase-1 | [AFK20474.1](http://smserver.sti.ua.es/millhtml/SM_instruct/servadmn.htm#update_acclinks?AFK20474.1) | 2 | 2 | 37.25 | [6.2](http://smserver.sti.ua.es/millbin/msdigest.cgi?missed_cleavages=2&msparams_dir=msparams_mill/&hide_protein_sequence=2&database=PA_haloferax_mediterranei_genbank&seqdb_dir=D:\SeqDB\&enzyme=Trypsin&access_method=Accession+Number&accession_num=AFK20474.1&coverage_map=0+98+22+46+12+363) | 1.58e+006 |
| NADH dehydrogenase, subunit N (ubiquinone) | [AFK18704.1](http://smserver.sti.ua.es/millhtml/SM_instruct/servadmn.htm#update_acclinks?AFK18704.1) | 2 | 2 | 36.97 | [3.5](http://smserver.sti.ua.es/millbin/msdigest.cgi?missed_cleavages=2&msparams_dir=msparams_mill/&hide_protein_sequence=2&database=PA_haloferax_mediterranei_genbank&seqdb_dir=D:\SeqDB\&enzyme=Trypsin&access_method=Accession+Number&accession_num=AFK18704.1&coverage_map=0+260+9+184+9+44) | 3.88e+006 |
| universal stress protein UspA-like protein | [AFK18989.1](http://smserver.sti.ua.es/millhtml/SM_instruct/servadmn.htm#update_acclinks?AFK18989.1) | 3 | 2 | 36.81 | [26.2](http://smserver.sti.ua.es/millbin/msdigest.cgi?missed_cleavages=2&msparams_dir=msparams_mill/&hide_protein_sequence=2&database=PA_haloferax_mediterranei_genbank&seqdb_dir=D:\SeqDB\&enzyme=Trypsin&access_method=Accession+Number&accession_num=AFK18989.1&coverage_map=0+43+28+45+9+16) | 4.11e+006 |
| oxidoreductase | [AFK18724.1](http://smserver.sti.ua.es/millhtml/SM_instruct/servadmn.htm#update_acclinks?AFK18724.1) | 2 | 2 | 36.71 | [6.7](http://smserver.sti.ua.es/millbin/msdigest.cgi?missed_cleavages=2&msparams_dir=msparams_mill/&hide_protein_sequence=2&database=PA_haloferax_mediterranei_genbank&seqdb_dir=D:\SeqDB\&enzyme=Trypsin&access_method=Accession+Number&accession_num=AFK18724.1&coverage_map=0+14+10+95+13+208) | 6.18e+005 |
| aminopeptidase | [AFK18528.1](http://smserver.sti.ua.es/millhtml/SM_instruct/servadmn.htm#update_acclinks?AFK18528.1) | 2 | 2 | 36.69 | [5.2](http://smserver.sti.ua.es/millbin/msdigest.cgi?missed_cleavages=2&msparams_dir=msparams_mill/&hide_protein_sequence=2&database=PA_haloferax_mediterranei_genbank&seqdb_dir=D:\SeqDB\&enzyme=Trypsin&access_method=Accession+Number&accession_num=AFK18528.1&coverage_map=0+54+11+229+12+129) | 8.99e+005 |
| hypothetical protein HFX_1684 | [AFK19390.1](http://smserver.sti.ua.es/millhtml/SM_instruct/servadmn.htm#update_acclinks?AFK19390.1) | 3 | 2 | 36.48 | [21.6](http://smserver.sti.ua.es/millbin/msdigest.cgi?missed_cleavages=2&msparams_dir=msparams_mill/&hide_protein_sequence=2&database=PA_haloferax_mediterranei_genbank&seqdb_dir=D:\SeqDB\&enzyme=Trypsin&access_method=Accession+Number&accession_num=AFK19390.1&coverage_map=0+39+24+48) | 1.49e+007 |
| CBS domain-containing protein | [AFK17996.1](http://smserver.sti.ua.es/millhtml/SM_instruct/servadmn.htm#update_acclinks?AFK17996.1) | 2 | 2 | 36.39 | [6.6](http://smserver.sti.ua.es/millbin/msdigest.cgi?missed_cleavages=2&msparams_dir=msparams_mill/&hide_protein_sequence=2&database=PA_haloferax_mediterranei_genbank&seqdb_dir=D:\SeqDB\&enzyme=Trypsin&access_method=Accession+Number&accession_num=AFK17996.1&coverage_map=0+293+31+141) | 1.49e+006 |
| hypothetical protein HFX_2260 | [AFK19948.1](http://smserver.sti.ua.es/millhtml/SM_instruct/servadmn.htm#update_acclinks?AFK19948.1) | 2 | 2 | 36.34 | [19.4](http://smserver.sti.ua.es/millbin/msdigest.cgi?missed_cleavages=2&msparams_dir=msparams_mill/&hide_protein_sequence=2&database=PA_haloferax_mediterranei_genbank&seqdb_dir=D:\SeqDB\&enzyme=Trypsin&access_method=Accession+Number&accession_num=AFK19948.1&coverage_map=0+152+48+47) | 1.09e+006 |
| ornithine cyclodeaminase | [AFK18155.1](http://smserver.sti.ua.es/millhtml/SM_instruct/servadmn.htm#update_acclinks?AFK18155.1) | 2 | 2 | 36.15 | [8.7](http://smserver.sti.ua.es/millbin/msdigest.cgi?missed_cleavages=2&msparams_dir=msparams_mill/&hide_protein_sequence=2&database=PA_haloferax_mediterranei_genbank&seqdb_dir=D:\SeqDB\&enzyme=Trypsin&access_method=Accession+Number&accession_num=AFK18155.1&coverage_map=0+156+29+147) | 1.03e+006 |
| electron transfer flavoprotein beta subunit | [AFK18030.1](http://smserver.sti.ua.es/millhtml/SM_instruct/servadmn.htm#update_acclinks?AFK18030.1) | 4 | 2 | 36.06 | [12.1](http://smserver.sti.ua.es/millbin/msdigest.cgi?missed_cleavages=2&msparams_dir=msparams_mill/&hide_protein_sequence=2&database=PA_haloferax_mediterranei_genbank&seqdb_dir=D:\SeqDB\&enzyme=Trypsin&access_method=Accession+Number&accession_num=AFK18030.1&coverage_map=0+8+15+60+17+164) | 2.89e+006 |
| hypothetical protein HFX_1141 | [AFK18857.1](http://smserver.sti.ua.es/millhtml/SM_instruct/servadmn.htm#update_acclinks?AFK18857.1) | 3 | 2 | 36.06 | [31.6](http://smserver.sti.ua.es/millbin/msdigest.cgi?missed_cleavages=2&msparams_dir=msparams_mill/&hide_protein_sequence=2&database=PA_haloferax_mediterranei_genbank&seqdb_dir=D:\SeqDB\&enzyme=Trypsin&access_method=Accession+Number&accession_num=AFK18857.1&coverage_map=0+20+12+17+20+32) | 1.96e+006 |
| serine hydroxymethyltransferase | [AFK20524.1](http://smserver.sti.ua.es/millhtml/SM_instruct/servadmn.htm#update_acclinks?AFK20524.1) | 2 | 2 | 35.37 | [5.7](http://smserver.sti.ua.es/millbin/msdigest.cgi?missed_cleavages=2&msparams_dir=msparams_mill/&hide_protein_sequence=2&database=PA_haloferax_mediterranei_genbank&seqdb_dir=D:\SeqDB\&enzyme=Trypsin&access_method=Accession+Number&accession_num=AFK20524.1&coverage_map=0+7+16+355+8+29) | 1.34e+006 |
| adenylosuccinate synthetase | [AFK18856.1](http://smserver.sti.ua.es/millhtml/SM_instruct/servadmn.htm#update_acclinks?AFK18856.1) | 2 | 2 | 35.02 | [6.5](http://smserver.sti.ua.es/millbin/msdigest.cgi?missed_cleavages=2&msparams_dir=msparams_mill/&hide_protein_sequence=2&database=PA_haloferax_mediterranei_genbank&seqdb_dir=D:\SeqDB\&enzyme=Trypsin&access_method=Accession+Number&accession_num=AFK18856.1&coverage_map=0+75+19+222+10+117) | 1.18e+006 |
| nitrite reductase copper containing protein | [AFK19882.1](http://smserver.sti.ua.es/millhtml/SM_instruct/servadmn.htm#update_acclinks?AFK19882.1) | 2 | 2 | 34.98 | [6.4](http://smserver.sti.ua.es/millbin/msdigest.cgi?missed_cleavages=2&msparams_dir=msparams_mill/&hide_protein_sequence=2&database=PA_haloferax_mediterranei_genbank&seqdb_dir=D:\SeqDB\&enzyme=Trypsin&access_method=Accession+Number&accession_num=AFK19882.1&coverage_map=0+230+12+70+13+61) | 9.31e+005 |
| serine protease inhibitor family protein | [AFK20013.1](http://smserver.sti.ua.es/millhtml/SM_instruct/servadmn.htm#update_acclinks?AFK20013.1) | 2 | 2 | 34.92 | [6.8](http://smserver.sti.ua.es/millbin/msdigest.cgi?missed_cleavages=2&msparams_dir=msparams_mill/&hide_protein_sequence=2&database=PA_haloferax_mediterranei_genbank&seqdb_dir=D:\SeqDB\&enzyme=Trypsin&access_method=Accession+Number&accession_num=AFK20013.1&coverage_map=0+123+21+178+10+121) | 3.56e+006 |
| glycosyl transferase (plasmid) | [AFK21270.1](http://smserver.sti.ua.es/millhtml/SM_instruct/servadmn.htm#update_acclinks?AFK21270.1) | 2 | 2 | 34.39 | [7.2](http://smserver.sti.ua.es/millbin/msdigest.cgi?missed_cleavages=2&msparams_dir=msparams_mill/&hide_protein_sequence=2&database=PA_haloferax_mediterranei_genbank&seqdb_dir=D:\SeqDB\&enzyme=Trypsin&access_method=Accession+Number&accession_num=AFK21270.1&coverage_map=0+204+10+21+12+57) | 1.32e+006 |
| geranylgeranyl hydrogenase-like protein / electron-transferring-flavoprotein dehydrogenase | [AFK19181.1](http://smserver.sti.ua.es/millhtml/SM_instruct/servadmn.htm#update_acclinks?AFK19181.1) | 2 | 2 | 34.34 | [4.8](http://smserver.sti.ua.es/millbin/msdigest.cgi?missed_cleavages=2&msparams_dir=msparams_mill/&hide_protein_sequence=2&database=PA_haloferax_mediterranei_genbank&seqdb_dir=D:\SeqDB\&enzyme=Trypsin&access_method=Accession+Number&accession_num=AFK19181.1&coverage_map=0+265+8+168+14+1) | 1.76e+006 |
| cytochrome c oxidase polypeptide I | [AFK18663.1](http://smserver.sti.ua.es/millhtml/SM_instruct/servadmn.htm#update_acclinks?AFK18663.1) | 2 | 2 | 34.33 | [2.5](http://smserver.sti.ua.es/millbin/msdigest.cgi?missed_cleavages=2&msparams_dir=msparams_mill/&hide_protein_sequence=2&database=PA_haloferax_mediterranei_genbank&seqdb_dir=D:\SeqDB\&enzyme=Trypsin&access_method=Accession+Number&accession_num=AFK18663.1&coverage_map=0+328+7+105+8+141) | 1.20e+007 |
| hypothetical protein HFX_1139 | [AFK18855.1](http://smserver.sti.ua.es/millhtml/SM_instruct/servadmn.htm#update_acclinks?AFK18855.1) | 3 | 2 | 33.73 | [55.7](http://smserver.sti.ua.es/millbin/msdigest.cgi?missed_cleavages=2&msparams_dir=msparams_mill/&hide_protein_sequence=2&database=PA_haloferax_mediterranei_genbank&seqdb_dir=D:\SeqDB\&enzyme=Trypsin&access_method=Accession+Number&accession_num=AFK18855.1&coverage_map=0+25+34+2) | 5.68e+006 |
| hypothetical protein HFX_0940 | [AFK18659.1](http://smserver.sti.ua.es/millhtml/SM_instruct/servadmn.htm#update_acclinks?AFK18659.1) | 2 | 2 | 33.36 | [7.5](http://smserver.sti.ua.es/millbin/msdigest.cgi?missed_cleavages=2&msparams_dir=msparams_mill/&hide_protein_sequence=2&database=PA_haloferax_mediterranei_genbank&seqdb_dir=D:\SeqDB\&enzyme=Trypsin&access_method=Accession+Number&accession_num=AFK18659.1&coverage_map=0+131+20+113) | 3.30e+006 |
| ABC-type transport system ATP-binding protein | [AFK18720.1](http://smserver.sti.ua.es/millhtml/SM_instruct/servadmn.htm#update_acclinks?AFK18720.1) | 2 | 2 | 32.98 | [12.1](http://smserver.sti.ua.es/millbin/msdigest.cgi?missed_cleavages=2&msparams_dir=msparams_mill/&hide_protein_sequence=2&database=PA_haloferax_mediterranei_genbank&seqdb_dir=D:\SeqDB\&enzyme=Trypsin&access_method=Accession+Number&accession_num=AFK18720.1&coverage_map=1+12+184+27+90) | 9.41e+005 |
| 3-hydroxyacyl-CoA dehydrogenase | [AFK19216.2](http://smserver.sti.ua.es/millhtml/SM_instruct/servadmn.htm#update_acclinks?AFK19216.2) | 2 | 2 | 32.65 | [8](http://smserver.sti.ua.es/millbin/msdigest.cgi?missed_cleavages=2&msparams_dir=msparams_mill/&hide_protein_sequence=2&database=PA_haloferax_mediterranei_genbank&seqdb_dir=D:\SeqDB\&enzyme=Trypsin&access_method=Accession+Number&accession_num=AFK19216.2&coverage_map=0+74+12+109+11+80) | 4.60e+006 |
| glucose-1-phosphate thymidylyltransferase | [AFK19833.1](http://smserver.sti.ua.es/millhtml/SM_instruct/servadmn.htm#update_acclinks?AFK19833.1) | 2 | 2 | 32.61 | [6.6](http://smserver.sti.ua.es/millbin/msdigest.cgi?missed_cleavages=2&msparams_dir=msparams_mill/&hide_protein_sequence=2&database=PA_haloferax_mediterranei_genbank&seqdb_dir=D:\SeqDB\&enzyme=Trypsin&access_method=Accession+Number&accession_num=AFK19833.1&coverage_map=0+196+14+95+10+48) | 1.27e+006 |
| ferredoxin (2Fe-2S) | [AFK20674.1](http://smserver.sti.ua.es/millhtml/SM_instruct/servadmn.htm#update_acclinks?AFK20674.1) | 2 | 2 | 32.59 | [19.3](http://smserver.sti.ua.es/millbin/msdigest.cgi?missed_cleavages=2&msparams_dir=msparams_mill/&hide_protein_sequence=2&database=PA_haloferax_mediterranei_genbank&seqdb_dir=D:\SeqDB\&enzyme=Trypsin&access_method=Accession+Number&accession_num=AFK20674.1&coverage_map=0+65+12+23+13+16) | 2.45e+006 |
| hypothetical protein HFX_5285 (plasmid) | [AFK21116.1](http://smserver.sti.ua.es/millhtml/SM_instruct/servadmn.htm#update_acclinks?AFK21116.1) | 2 | 2 | 32.58 | [13.4](http://smserver.sti.ua.es/millbin/msdigest.cgi?missed_cleavages=2&msparams_dir=msparams_mill/&hide_protein_sequence=2&database=PA_haloferax_mediterranei_genbank&seqdb_dir=D:\SeqDB\&enzyme=Trypsin&access_method=Accession+Number&accession_num=AFK21116.1&coverage_map=0+206+13+3+20+3) | 9.54e+005 |
| hypothetical protein HFX_1650 | [AFK19356.1](http://smserver.sti.ua.es/millhtml/SM_instruct/servadmn.htm#update_acclinks?AFK19356.1) | 2 | 2 | 32.48 | [9.6](http://smserver.sti.ua.es/millbin/msdigest.cgi?missed_cleavages=2&msparams_dir=msparams_mill/&hide_protein_sequence=2&database=PA_haloferax_mediterranei_genbank&seqdb_dir=D:\SeqDB\&enzyme=Trypsin&access_method=Accession+Number&accession_num=AFK19356.1&coverage_map=0+143+25+92) | 1.35e+006 |
| carbohydrate ABC transporter substrate-binding protein, CUT1 family | [AFK20566.1](http://smserver.sti.ua.es/millhtml/SM_instruct/servadmn.htm#update_acclinks?AFK20566.1) | 2 | 2 | 32.11 | [5.2](http://smserver.sti.ua.es/millbin/msdigest.cgi?missed_cleavages=2&msparams_dir=msparams_mill/&hide_protein_sequence=2&database=PA_haloferax_mediterranei_genbank&seqdb_dir=D:\SeqDB\&enzyme=Trypsin&access_method=Accession+Number&accession_num=AFK20566.1&coverage_map=0+218+14+133+10+79) | 3.64e+006 |
| enoyl-CoA hydratase (plasmid) | [AFK21050.1](http://smserver.sti.ua.es/millhtml/SM_instruct/servadmn.htm#update_acclinks?AFK21050.1) | 2 | 2 | 31.71 | [14.6](http://smserver.sti.ua.es/millbin/msdigest.cgi?missed_cleavages=2&msparams_dir=msparams_mill/&hide_protein_sequence=2&database=PA_haloferax_mediterranei_genbank&seqdb_dir=D:\SeqDB\&enzyme=Trypsin&access_method=Accession+Number&accession_num=AFK21050.1&coverage_map=0+132+17+23+15+32) | 1.61e+006 |
| DNA-binding ferritin-like protein (oxidative damage protectant) | [AFK19391.2](http://smserver.sti.ua.es/millhtml/SM_instruct/servadmn.htm#update_acclinks?AFK19391.2) | 2 | 2 | 31.48 | [20.4](http://smserver.sti.ua.es/millbin/msdigest.cgi?missed_cleavages=2&msparams_dir=msparams_mill/&hide_protein_sequence=2&database=PA_haloferax_mediterranei_genbank&seqdb_dir=D:\SeqDB\&enzyme=Trypsin&access_method=Accession+Number&accession_num=AFK19391.2&coverage_map=0+115+37+29) | 2.14e+006 |
| copper-transporting ATPase CopA (plasmid) | [AFK21064.1](http://smserver.sti.ua.es/millhtml/SM_instruct/servadmn.htm#update_acclinks?AFK21064.1) | 2 | 2 | 31.46 | [3.7](http://smserver.sti.ua.es/millbin/msdigest.cgi?missed_cleavages=2&msparams_dir=msparams_mill/&hide_protein_sequence=2&database=PA_haloferax_mediterranei_genbank&seqdb_dir=D:\SeqDB\&enzyme=Trypsin&access_method=Accession+Number&accession_num=AFK21064.1&coverage_map=0+585+12+61+17+103) | 1.99e+005 |
| hsp20-type chaperone (plasmid) | [AFK20982.1](http://smserver.sti.ua.es/millhtml/SM_instruct/servadmn.htm#update_acclinks?AFK20982.1) | 2 | 2 | 31.42 | [15.6](http://smserver.sti.ua.es/millbin/msdigest.cgi?missed_cleavages=2&msparams_dir=msparams_mill/&hide_protein_sequence=2&database=PA_haloferax_mediterranei_genbank&seqdb_dir=D:\SeqDB\&enzyme=Trypsin&access_method=Accession+Number&accession_num=AFK20982.1&coverage_map=0+86+11+38+12) | 5.76e+005 |
| ABC transporter ATP-binding protein | [AFK18549.1](http://smserver.sti.ua.es/millhtml/SM_instruct/servadmn.htm#update_acclinks?AFK18549.1) | 2 | 2 | 31.35 | [7.6](http://smserver.sti.ua.es/millbin/msdigest.cgi?missed_cleavages=2&msparams_dir=msparams_mill/&hide_protein_sequence=2&database=PA_haloferax_mediterranei_genbank&seqdb_dir=D:\SeqDB\&enzyme=Trypsin&access_method=Accession+Number&accession_num=AFK18549.1&coverage_map=0+12+11+61+12+205) | 1.22e+006 |
| cell division protein ftsZ | [AFK19949.2](http://smserver.sti.ua.es/millhtml/SM_instruct/servadmn.htm#update_acclinks?AFK19949.2) | 2 | 2 | 31.29 | [7.3](http://smserver.sti.ua.es/millbin/msdigest.cgi?missed_cleavages=2&msparams_dir=msparams_mill/&hide_protein_sequence=2&database=PA_haloferax_mediterranei_genbank&seqdb_dir=D:\SeqDB\&enzyme=Trypsin&access_method=Accession+Number&accession_num=AFK19949.2&coverage_map=0+294+14+31+15+41) | 5.88e+005 |
| electron transfer flavoprotein beta subunit | [AFK20417.2](http://smserver.sti.ua.es/millhtml/SM_instruct/servadmn.htm#update_acclinks?AFK20417.2) | 2 | 2 | 31.28 | [10.6](http://smserver.sti.ua.es/millbin/msdigest.cgi?missed_cleavages=2&msparams_dir=msparams_mill/&hide_protein_sequence=2&database=PA_haloferax_mediterranei_genbank&seqdb_dir=D:\SeqDB\&enzyme=Trypsin&access_method=Accession+Number&accession_num=AFK20417.2&coverage_map=0+40+10+18+21+201) | 2.40e+006 |
| halocyanin precursor-like protein | [AFK18843.1](http://smserver.sti.ua.es/millhtml/SM_instruct/servadmn.htm#update_acclinks?AFK18843.1) | 2 | 2 | 31.14 | [15.6](http://smserver.sti.ua.es/millbin/msdigest.cgi?missed_cleavages=2&msparams_dir=msparams_mill/&hide_protein_sequence=2&database=PA_haloferax_mediterranei_genbank&seqdb_dir=D:\SeqDB\&enzyme=Trypsin&access_method=Accession+Number&accession_num=AFK18843.1&coverage_map=0+51+8+89+18) | 5.16e+006 |
| putative extracellular solute binding protein (plasmid) | [AFK21314.1](http://smserver.sti.ua.es/millhtml/SM_instruct/servadmn.htm#update_acclinks?AFK21314.1) | 3 | 2 | 30.95 | [5](http://smserver.sti.ua.es/millbin/msdigest.cgi?missed_cleavages=2&msparams_dir=msparams_mill/&hide_protein_sequence=2&database=PA_haloferax_mediterranei_genbank&seqdb_dir=D:\SeqDB\&enzyme=Trypsin&access_method=Accession+Number&accession_num=AFK21314.1&coverage_map=0+412+23+18) | 1.34e+006 |
| NADH dehydrogenase, subunit L (ubiquinone) | [AFK18702.1](http://smserver.sti.ua.es/millhtml/SM_instruct/servadmn.htm#update_acclinks?AFK18702.1) | 2 | 2 | 30.88 | [4.8](http://smserver.sti.ua.es/millbin/msdigest.cgi?missed_cleavages=2&msparams_dir=msparams_mill/&hide_protein_sequence=2&database=PA_haloferax_mediterranei_genbank&seqdb_dir=D:\SeqDB\&enzyme=Trypsin&access_method=Accession+Number&accession_num=AFK18702.1&coverage_map=0+174+6+436+27+35) | 6.16e+006 |
| tryptophanase | [AFK17753.2](http://smserver.sti.ua.es/millhtml/SM_instruct/servadmn.htm#update_acclinks?AFK17753.2) | 2 | 2 | 30.81 | [5.8](http://smserver.sti.ua.es/millbin/msdigest.cgi?missed_cleavages=2&msparams_dir=msparams_mill/&hide_protein_sequence=2&database=PA_haloferax_mediterranei_genbank&seqdb_dir=D:\SeqDB\&enzyme=Trypsin&access_method=Accession+Number&accession_num=AFK17753.2&coverage_map=0+277+26+145) | 8.10e+005 |
| hypothetical protein HFX_0214 | [AFK17955.1](http://smserver.sti.ua.es/millhtml/SM_instruct/servadmn.htm#update_acclinks?AFK17955.1) | 3 | 2 | 30.78 | [8.4](http://smserver.sti.ua.es/millbin/msdigest.cgi?missed_cleavages=2&msparams_dir=msparams_mill/&hide_protein_sequence=2&database=PA_haloferax_mediterranei_genbank&seqdb_dir=D:\SeqDB\&enzyme=Trypsin&access_method=Accession+Number&accession_num=AFK17955.1&coverage_map=0+123+30+204) | 6.59e+006 |
| stress response protein | [AFK18921.1](http://smserver.sti.ua.es/millhtml/SM_instruct/servadmn.htm#update_acclinks?AFK18921.1) | 2 | 2 | 30.74 | [15.1](http://smserver.sti.ua.es/millbin/msdigest.cgi?missed_cleavages=2&msparams_dir=msparams_mill/&hide_protein_sequence=2&database=PA_haloferax_mediterranei_genbank&seqdb_dir=D:\SeqDB\&enzyme=Trypsin&access_method=Accession+Number&accession_num=AFK18921.1&coverage_map=0+42+13+64+9+17) | 1.26e+006 |
| hypothetical protein HFX_1590 | [AFK19296.1](http://smserver.sti.ua.es/millhtml/SM_instruct/servadmn.htm#update_acclinks?AFK19296.1) | 2 | 2 | 30.70 | [2.9](http://smserver.sti.ua.es/millbin/msdigest.cgi?missed_cleavages=2&msparams_dir=msparams_mill/&hide_protein_sequence=2&database=PA_haloferax_mediterranei_genbank&seqdb_dir=D:\SeqDB\&enzyme=Trypsin&access_method=Accession+Number&accession_num=AFK19296.1&coverage_map=0+221+7+59+8+220) | 3.01e+006 |
| hypothetical protein HFX_0016 | [AFK17760.1](http://smserver.sti.ua.es/millhtml/SM_instruct/servadmn.htm#update_acclinks?AFK17760.1) | 2 | 2 | 30.57 | [11.9](http://smserver.sti.ua.es/millbin/msdigest.cgi?missed_cleavages=2&msparams_dir=msparams_mill/&hide_protein_sequence=2&database=PA_haloferax_mediterranei_genbank&seqdb_dir=D:\SeqDB\&enzyme=Trypsin&access_method=Accession+Number&accession_num=AFK17760.1&coverage_map=0+39+12+19+11+111) | 9.86e+005 |
| hypothetical protein HFX_1138 | [AFK18854.1](http://smserver.sti.ua.es/millhtml/SM_instruct/servadmn.htm#update_acclinks?AFK18854.1) | 2 | 2 | 30.39 | [13.5](http://smserver.sti.ua.es/millbin/msdigest.cgi?missed_cleavages=2&msparams_dir=msparams_mill/&hide_protein_sequence=2&database=PA_haloferax_mediterranei_genbank&seqdb_dir=D:\SeqDB\&enzyme=Trypsin&access_method=Accession+Number&accession_num=AFK18854.1&coverage_map=0+27+10+33+16+106) | 2.06e+006 |
| sulfatase arylsulfatase A-like protein | [AFK19826.1](http://smserver.sti.ua.es/millhtml/SM_instruct/servadmn.htm#update_acclinks?AFK19826.1) | 2 | 2 | 30.18 | [4.4](http://smserver.sti.ua.es/millbin/msdigest.cgi?missed_cleavages=2&msparams_dir=msparams_mill/&hide_protein_sequence=2&database=PA_haloferax_mediterranei_genbank&seqdb_dir=D:\SeqDB\&enzyme=Trypsin&access_method=Accession+Number&accession_num=AFK19826.1&coverage_map=0+173+12+123+8+132) | 2.38e+006 |
| endoglucanase | [AFK18519.1](http://smserver.sti.ua.es/millhtml/SM_instruct/servadmn.htm#update_acclinks?AFK18519.1) | 2 | 2 | 30.11 | [7.1](http://smserver.sti.ua.es/millbin/msdigest.cgi?missed_cleavages=2&msparams_dir=msparams_mill/&hide_protein_sequence=2&database=PA_haloferax_mediterranei_genbank&seqdb_dir=D:\SeqDB\&enzyme=Trypsin&access_method=Accession+Number&accession_num=AFK18519.1&coverage_map=0+132+14+30+11+161) | 1.36e+006 |
| hypothetical protein HFX_0813 | [AFK18535.1](http://smserver.sti.ua.es/millhtml/SM_instruct/servadmn.htm#update_acclinks?AFK18535.1) | 2 | 2 | 30.00 | [13.7](http://smserver.sti.ua.es/millbin/msdigest.cgi?missed_cleavages=2&msparams_dir=msparams_mill/&hide_protein_sequence=2&database=PA_haloferax_mediterranei_genbank&seqdb_dir=D:\SeqDB\&enzyme=Trypsin&access_method=Accession+Number&accession_num=AFK18535.1&coverage_map=0+78+11+34+7+1) | 3.58e+006 |

Tabla S2. Representative proteins identified in the crude extract using Triton X-100 (20% w/v) from 3 LC-MS/MS runs.

| **Protein Name** | **Database**  **Accession** | **Spectra** | **Distinct**  **Peptides** | **Distinct**  **Summed**  **MS/MS Search**  **Score** | **% AA**  **Coverage** | **Total Protein Spectral Intensity** |
| --- | --- | --- | --- | --- | --- | --- |
| SPFH domain, Band 7 family protein | AFK18490.1 | 26 | 23 | 412.83 | 61.5 | 1.82e+008 |
| SPFH domain, Band 7 family protein | AFK17777.1 | 14 | 12 | 194.21 | 36.5 | 4.33e+007 |
| NADH dehydrogenase, subunit CD (ubiquinone) | AFK18696.1 | 26 | 21 | 345.12 | 50 | 7.64e+007 |
| NADH dehydrogenase, subunit D (ubiquinone) | AFK18684.1 | 7 | 5 | 84.06 | 8.3 | 2.05e+007 |
| Dimethylsulfoxide reductase | AFK19896.1 | 21 | 21 | 333.00 | 35.7 | 1.68e+007 |
| Dimethylsulfoxide reductase | AFK19898.1 | 21 | 19 | 307.07 | 31.7 | 1.54e+007 |
| Succinate dehydrogenase, subunit A (flavoprotein) | AFK20495.1 | 22 | 18 | 315.95 | 45.2 | 4.73e+007 |
| A-type ATP synthase subunit A | AFK18041.1 | 27 | 17 | 299.09 | 38.3 | 7.98e+007 |
| Thermosome, beta subunit | AFK18158.2 | 25 | 17 | 285.26 | 41.1 | 3.31e+007 |
| Nitrous-oxide reductase (plasmid) | AFK20926.1 | 21 | 16 | 263.70 | 26.2 | 9.36e+007 |
| Thermosome alpha subunit | AFK18461.1 | 20 | 15 | 263.30 | 43.1 | 1.68e+007 |
| Poly(3-hydroxyalkanoate) synthase subunit PhaC (plasmid) | AFK21054.1 | 20 | 14 | 258.26 | 41 | 3.87e+007 |
| Serine protease (plasmid) | AFK21203.1 | 21 | 15 | 252.64 | 38.8 | 1.47e+008 |
| Dipeptide ABC transporter dipeptide-binding protein | AFK17806.1 | 15 | 14 | 245.78 | 25.3 | 4.34e+007 |
| A-type ATP synthase subunit C | AFK18039.1 | 20 | 15 | 242.40 | 59.7 | 5.21e+007 |
| A-type ATP synthase subunit B | AFK18042.1 | 21 | 14 | 239.76 | 44.8 | 8.40e+007 |
| FAD dependent oxidoreductase | AFK19621.1 | 15 | 14 | 228.61 | 39.1 | 1.23e+007 |
| Dipeptide/oligopeptide/nickel ABC transporter periplasmic substrate-binding protein | AFK18790.1 | 16 | 14 | 221.56 | 31.2 | 8.57e+006 |
| Nucleoside-binding protein | AFK19185.1 | 16 | 12 | 214.50 | 41.7 | 2.02e+007 |
| A-type ATP synthase subunit I | AFK18036.1 | 16 | 12 | 201.11 | 20.5 | 3.69e+007 |
| Sugar ABC transporter substrate binding protein | AFK20386.1 | 13 | 11 | 194.32 | 32.2 | 3.04e+007 |
| Potassium transport protein kefC | AFK19701.1 | 17 | 12 | 189.75 | 24.5 | 1.19e+007 |
| Periplasmic solute binding protein | AFK20085.1 | 13 | 11 | 186.53 | 36 | 9.40e+006 |
| Transmembrane oligosaccharyl transferase / dolichyl-diphosphooligosaccharide--protein glycosyltransferase | AFK19298.1 | 13 | 12 | 186.07 | 13.7 | 1.59e+007 |
| AAA-type ATPase (transitional ATPase-like protein) | AFK20074.1 | 13 | 12 | 184.10 | 24.2 | 7.28e+006 |
| Poly(3-hydroxyalkanoate) synthase subunit PhaE (plasmid) | AFK21053.1 | 25 | 9 | 181.07 | 71.4 | 1.59e+008 |
| Dipeptide ABC transporter ATP-binding protein | AFK18330.2 | 14 | 10 | 180.90 | 26.3 | 2.76e+007 |
| Thermosome, alpha subunit | AFK17883.2 | 14 | 11 | 180.87 | 28.3 | 1.36e+007 |
| ABC-type dipeptide/oligopeptide/nickel transport system, substrate-binding protein | AFK19559.1 | 11 | 11 | 175.35 | 25.7 | 7.09e+006 |
| Glutamate dehydrogenase (NAD(P)+) | AFK19225.1 | 11 | 10 | 166.21 | 38.6 | 7.64e+006 |
| Hypothetical protein HFX_6053 (plasmid) | AFK21180.1 | 12 | 11 | 164.38 | 53.2 | 8.17e+006 |
| PBS lyase HEAT-like repeat protein | AFK18736.1 | 12 | 9 | 164.04 | 31.4 | 3.01e+007 |
| CBS domain-containing protein | AFK20079.1 | 14 | 10 | 159.04 | 32.8 | 3.02e+007 |
| Aldehyde dehydrogenase (NAD+) | AFK18912.1 | 11 | 10 | 158.34 | 24.8 | 7.53e+006 |
| Halocyanin hcpG | AFK18949.1 | 12 | 9 | 156.44 | 16.1 | 1.60e+007 |
| Oxidoreductase | AFK18898.2 | 10 | 9 | 155.67 | 41.8 | 3.97e+006 |
| Membrane protease subunit, stomatin/prohibitin | AFK17891.1 | 10 | 9 | 151.96 | 30.1 | 1.08e+007 |
| AAA-type ATPase (transitional ATPase-like protein) | AFK20391.1 | 12 | 10 | 151.58 | 14.5 | 1.09e+007 |
| AAA-type ATPase (transitional ATPase-like protein) | AFK19115.1 | 3 | 3 | 35.34 | 5.3 | 5.53e+005 |
| NADH dehydrogenase | AFK19343.1 | 9 | 9 | 150.39 | 37.2 | 7.04e+006 |
| Gluconate dehydratase | AFK19260.1 | 10 | 9 | 148.39 | 29.1 | 7.26e+006 |
| Hypothetical protein HFX_1529 | AFK19236.1 | 11 | 9 | 140.87 | 34.3 | 6.56e+006 |
| Hypothetical protein HFX_1777 | AFK19482.1 | 9 | 8 | 139.90 | 73 | 3.61e+007 |
| Arylsulfatase | AFK18199.1 | 11 | 10 | 139.81 | 25.9 | 6.72e+006 |
| Hypothetical protein HFX_2220 | AFK19909.2 | 11 | 7 | 136.19 | 4.9 | 6.07e+007 |
| A-type ATP synthase subunit D | AFK18044.1 | 11 | 8 | 135.61 | 47.8 | 1.11e+007 |
| Putative hydrolase or acyltransferase of alpha/beta superfamily | AFK18525.1 | 9 | 8 | 133.91 | 49.3 | 1.54e+007 |
| Isocitrate dehydrogenase (NADP+) | AFK20291.1 | 10 | 9 | 133.18 | 32.6 | 5.83e+006 |
| Glycosyl transferase | AFK19287.1 | 9 | 8 | 133.00 | 28.9 | 4.86e+006 |
| Putative phosphonate ABC transporter, periplasmic phosphonate-binding protein | AFK19847.1 | 9 | 7 | 131.51 | 25.3 | 8.29e+006 |
| Gas-vesicle operon protein gvpC | AFK19401.1 | 10 | 7 | 129.44 | 28.8 | 1.31e+007 |
| Hypothetical protein HFX_2459 | AFK20144.2 | 14 | 7 | 126.81 | 13 | 1.07e+008 |
| A-type ATP synthase subunit H | AFK18035.1 | 8 | 7 | 126.73 | 40.9 | 7.02e+006 |
| Cytochrome b subunit of nitric oxide reductase | AFK19877.1 | 10 | 7 | 126.10 | 14.5 | 1.15e+007 |
| Ca2+-transporting ATPase | AFK18654.1 | 8 | 8 | 125.96 | 15.8 | 2.43e+006 |
| Dipeptide/oligopeptide/nickel ABC transporter ATP-binding protein | AFK17802.1 | 12 | 9 | 125.78 | 21.9 | 8.72e+006 |
| Dipeptide/oligopeptide/nickel ABC transporter ATP-binding protein | AFK18794.1 | 2 | 2 | 25.80 | 5 | 9.44e+005 |
| Pyruvate--ferredoxin oxidoreductase, alpha subunit | AFK19081.1 | 8 | 8 | 125.77 | 17.9 | 3.54e+006 |
| Enoyl-CoA hydratase (plasmid) | AFK21050.1 | 13 | 8 | 125.47 | 58.9 | 2.29e+007 |
| A-type ATP synthase subunit E | AFK18038.1 | 13 | 7 | 125.09 | 52.5 | 2.86e+007 |
| Hypothetical protein HFX_1679 | AFK19385.1 | 9 | 9 | 124.41 | 22.4 | 3.01e+006 |
| ABC-type dipeptide/oligopeptide/nickel transport system, substrate-binding protein (plasmid) | AFK21027.1 | 9 | 8 | 122.71 | 16.9 | 1.32e+007 |
| Short chain dehydrogenase/ reductase | AFK18407.1 | 8 | 8 | 122.28 | 42.3 | 3.81e+006 |
| Flavin-dependent dehydrogenase | AFK20419.1 | 9 | 8 | 120.55 | 23.4 | 1.02e+007 |
| Hypothetical protein HFX_2509 | AFK20191.2 | 8 | 8 | 120.25 | 32.7 | 6.66e+006 |
| 3-ketoacyl-acyl carrier protein reductase (PhaB) (plasmid) | AFK21048.1 | 9 | 7 | 119.22 | 39.1 | 1.20e+007 |
| Nitrate reductase alpha chain (plasmid) | AFK20939.1 | 8 | 8 | 119.17 | 11.7 | 2.58e+006 |
| Glutamine synthetase | AFK17986.2 | 10 | 7 | 118.08 | 26.3 | 9.51e+006 |
| Succinate dehydrogenase, subunit B (iron-sulfur protein) | AFK20496.1 | 8 | 7 | 114.61 | 22.1 | 8.77e+006 |
| Nitrate reductase beta chain (plasmid) | AFK20938.1 | 10 | 8 | 112.14 | 24.4 | 3.27e+006 |
| ABC-type dipeptide/oligopeptide/nickel transport system, substrate binding protein | AFK20131.1 | 9 | 8 | 110.90 | 20.1 | 4.31e+006 |
| Sulfatase arylsulfatase A-like protein | AFK18425.2 | 8 | 8 | 110.68 | 15.1 | 4.71e+006 |
| Sulfatase arylsulfatase A-like protein | AFK19826.1 | 4 | 4 | 50.72 | 9.5 | 3.38e+006 |
| Putative cation-transporting ATPase | AFK18658.2 | 7 | 7 | 109.48 | 12.8 | 2.08e+006 |
| Hypothetical protein HFX_1249 | AFK18962.1 | 8 | 8 | 109.48 | 21.7 | 3.40e+006 |
| Nitrite reductase (NO-forming) | AFK19872.1 | 7 | 6 | 108.21 | 20.8 | 6.04e+007 |
| 2-oxoglutarate ferredoxin oxidoreductase, subunit alpha | AFK18583.1 | 8 | 7 | 108.01 | 15 | 5.25e+006 |
| Aldehyde dehydrogenase | AFK20313.1 | 8 | 7 | 108.01 | 19 | 3.72e+006 |
| Hypothetical protein HFX_6273 (plasmid) | AFK21396.2 | 7 | 7 | 107.41 | 23.3 | 2.91e+006 |
| CBS domain-containing protein | AFK17996.1 | 8 | 6 | 106.44 | 19.5 | 4.56e+006 |
| Hypothetical protein HFX_6060 (plasmid) | AFK21187.1 | 7 | 7 | 104.79 | 25.3 | 1.73e+006 |
| Acyl-CoA synthetase | AFK18591.1 | 7 | 7 | 104.55 | 14.9 | 8.83e+005 |
| Acyl-CoA synthetase (plasmid) | AFK20965.1 | 3 | 3 | 36.12 | 4.8 | 4.17e+005 |
| Aconitate hydratase | AFK18238.1 | 8 | 6 | 103.87 | 10.6 | 8.26e+006 |
| NADH dehydrogenase, subunit B (ubiquinone) | AFK18695.1 | 9 | 6 | 103.65 | 33.4 | 1.72e+007 |
| Urocanate hydratase (plasmid) | AFK21450.1 | 6 | 6 | 103.49 | 16.2 | 1.67e+006 |
| Orotate phosphoribosyltransferase-like protein/conserved Entner-Douderoff pathway protein | AFK18805.1 | 8 | 7 | 102.33 | 39 | 3.43e+006 |
| Hypothetical protein HFX_1776 | AFK19481.1 | 7 | 7 | 102.01 | 15 | 9.43e+006 |
| Prepilin signal peptidase | AFK20651.1 | 7 | 7 | 101.73 | 24 | 5.35e+006 |
| Poly(3-hydroxyalkanoate) granule-associated 12 kDa protein (plasmid) | AFK21051.1 | 8 | 5 | 101.10 | 77.2 | 5.70e+007 |
| Molecular chaperone DnaK | AFK19355.1 | 6 | 6 | 100.93 | 16.4 | 2.21e+006 |
| Hypothetical protein HFX_0293 | AFK18032.2 | 7 | 7 | 100.55 | 18.3 | 1.89e+006 |
| Catalase (including: peroxidase) | AFK19564.1 | 6 | 6 | 99.39 | 11.6 | 4.86e+006 |
| CBS domain-containing protein | AFK18713.2 | 7 | 7 | 97.53 | 25.2 | 2.45e+006 |
| ABC transporter ATP-binding protein | AFK20685.1 | 7 | 7 | 96.76 | 33.3 | 2.85e+006 |
| Dihydrolipoamide S-acyltransferase (pyruvate dehydrogenase E2 component) + | AFK20618.1 | 7 | 7 | 94.55 | 17 | 5.49e+006 |
| ABC-type dipeptide/oligopeptide/nickel transport systems, ATP-binding protein I & II | AFK18329.1 | 7 | 7 | 94.45 | 10.9 | 1.41e+006 |
| Phosphonates ABC transporter ATP-binding protein | AFK19846.1 | 7 | 6 | 94.10 | 34 | 5.68e+006 |
| Hypothetical protein HFX_2088 | AFK19779.1 | 7 | 6 | 92.94 | 20.2 | 2.21e+006 |
| Branched-chain/neutral amino acids amide ABC transporter periplasmic substrate-binding protein | AFK19529.1 | 7 | 5 | 92.49 | 19.9 | 9.04e+006 |
| Stress response protein | AFK20336.2 | 6 | 6 | 92.47 | 28.8 | 6.29e+006 |
| Zinc-transporting ATPase (plasmid) | AFK21399.1 | 6 | 6 | 92.33 | 10.6 | 2.54e+006 |
| Proline dehydrogenase | AFK18055.1 | 6 | 6 | 91.23 | 25 | 5.49e+006 |
| ABC-type iron(III) transport system,substrate-binding protein | AFK19499.1 | 9 | 5 | 90.78 | 23.2 | 1.21e+007 |
| purK operon protein / membrane-bound mannosyltransferase | AFK18691.1 | 7 | 6 | 90.72 | 13.1 | 2.24e+006 |
| Pyruvate dehydrogenase E1 component subunit beta | AFK20617.1 | 7 | 5 | 90.40 | 21.7 | 6.80e+006 |
| Molybdopterin oxidoreductase | AFK19899.1 | 6 | 5 | 90.39 | 22.7 | 8.23e+006 |
| Phenylacetic acid degradation protein PaaC (plasmid) | AFK21497.1 | 6 | 6 | 89.78 | 27.5 | 2.26e+006 |
| Carbohydrate ABC transporter substrate-binding protein, CUT1 family | AFK20566.1 | 6 | 5 | 89.25 | 21.5 | 6.84e+006 |
| Halocyanin precursor-like protein | AFK19879.1 | 6 | 5 | 88.97 | 26.5 | 1.52e+007 |
| Iron-sulfur protein (4Fe-4S) | AFK17960.2 | 7 | 7 | 88.70 | 10.6 | 6.10e+006 |
| Hypothetical protein HFX_2555 | AFK20236.1 | 6 | 6 | 88.38 | 23.5 | 7.21e+006 |
| Sugar ABC transporter ATP-binding protein (UGPC) | AFK20563.1 | 7 | 6 | 88.32 | 25.8 | 3.05e+006 |
| Phosphohexomutase (phosphoglucomutase, phosphomannomutase) | AFK17941.1 | 6 | 6 | 88.05 | 15.7 | 2.95e+006 |
| Hypothetical protein HFX_1575 | AFK19282.2 | 6 | 5 | 85.06 | 31.5 | 1.06e+007 |
| Phosphoenolpyruvate synthase / pyruvate, water dikinase | AFK18505.1 | 6 | 6 | 84.92 | 7.9 | 2.29e+006 |
| S-adenosylmethionine-dependent methyltransferase-like protein | AFK18456.1 | 6 | 6 | 84.78 | 26.9 | 2.57e+006 |
| Phytoene dehydrogenase (phytoene desaturase) | AFK18509.1 | 6 | 6 | 83.82 | 20.1 | 1.97e+006 |
| Molybdate transport protein | AFK19675.1 | 5 | 5 | 82.51 | 18.5 | 9.33e+005 |
| Sugar ABC transporter ATP-binding protein | AFK20383.1 | 7 | 5 | 82.43 | 19.7 | 6.86e+006 |
| Protein-disulfide isomerase | AFK18965.1 | 4 | 4 | 82.27 | 29.7 | 2.19e+006 |
| Hypothetical protein HFX_2780 | AFK20456.1 | 6 | 5 | 82.24 | 24.6 | 1.90e+006 |
| Preprotein translocase subunit SecY | AFK20246.1 | 9 | 6 | 82.18 | 13 | 1.05e+007 |
| Glutamate/aspartate transport protein | AFK19350.1 | 7 | 6 | 81.86 | 17.7 | 5.00e+006 |
| Phosphopyruvate hydratase | AFK20460.1 | 5 | 5 | 81.59 | 21.3 | 3.03e+006 |
| Preprotein translocase subunit SecD | AFK19761.1 | 5 | 5 | 80.89 | 18.5 | 5.38e+006 |
| Nitrite reductase copper containing protein | AFK19882.1 | 6 | 5 | 80.16 | 17.3 | 4.77e+006 |
| Gas-vesicle operon protein gvpH | AFK19407.1 | 5 | 4 | 79.99 | 30 | 3.84e+006 |
| Pyridoxal biosynthesis lyase PdxS | AFK20032.1 | 6 | 6 | 79.90 | 22.1 | 3.34e+006 |
| Hypothetical protein HFX_6074 (plasmid) | AFK21201.1 | 8 | 4 | 79.80 | 36.1 | 1.33e+007 |
| Hypothetical protein HFX_6434 (plasmid) | AFK21553.1 | 6 | 5 | 79.23 | 16.9 | 2.59e+006 |
| Phosphate ABC transporter ATP-binding protein | AFK20072.1 | 6 | 6 | 79.13 | 18.9 | 3.95e+006 |
| Putative hydrolase or acyltransferase of alpha/beta superfamily | AFK19333.1 | 5 | 5 | 78.52 | 26.3 | 4.89e+006 |
| Aspartate aminotransferase | AFK18689.1 | 5 | 5 | 78.36 | 22.3 | 1.34e+006 |
| Glycerol kinase | AFK19308.1 | 5 | 5 | 77.77 | 17.1 | 1.14e+006 |
| Dihydrolipoamide dehydrogenase | AFK20619.1 | 5 | 5 | 77.35 | 13.8 | 3.03e+006 |
| Phosphate ABC transporter periplasmic substrate-binding protein | AFK20069.1 | 5 | 5 | 76.85 | 14.8 | 2.54e+006 |
| 4-aminobutyrate aminotransferase | AFK19853.1 | 6 | 5 | 76.63 | 18.3 | 4.04e+006 |
| Putative signal transduction protein with CBS domains (plasmid) | AFK21146.1 | 5 | 5 | 76.55 | 11.8 | 3.11e+006 |
| Hypothetical protein HFX_1950 | AFK19646.1 | 5 | 5 | 76.03 | 36.8 | 2.83e+006 |
| Proteasome subunit alpha | AFK18815.1 | 6 | 6 | 75.73 | 22.2 | 8.89e+006 |
| Molybdopterin oxidoreductase | AFK19900.1 | 4 | 4 | 75.68 | 10.2 | 8.97e+006 |
| Putative mechanosensitive ion channel | AFK19964.1 | 7 | 4 | 74.75 | 26.6 | 3.34e+006 |
| Oxidoreductase | AFK18724.1 | 6 | 5 | 73.90 | 19.1 | 1.39e+006 |
| Biotin carboxylase | AFK20173.1 | 5 | 5 | 73.51 | 9.9 | 2.16e+006 |
| IMP dehydrogenase | AFK18995.1 | 5 | 5 | 73.44 | 12.4 | 1.50e+006 |
| ABC transporter ATP-binding protein (plasmid) | AFK20923.1 | 5 | 5 | 73.34 | 21.1 | 3.06e+006 |
| Hypothetical protein HFX_1590 | AFK19296.1 | 5 | 5 | 73.19 | 12.8 | 2.26e+006 |
| Putative hydrolase of the metallo-beta-lactamase superfamily | AFK20411.1 | 5 | 5 | 73.09 | 12.2 | 3.86e+006 |
| Aconitate hydratase | AFK19741.1 | 5 | 5 | 72.92 | 10.5 | 1.81e+006 |
| Geranylgeranyl hydrogenase-like protein / electron-transferring-flavoprotein dehydrogenase | AFK19181.1 | 5 | 5 | 71.74 | 12.2 | 2.79e+006 |
| Enoyl-CoA hydratase | AFK19171.2 | 5 | 5 | 71.56 | 22.5 | 3.22e+006 |
| Putative phosphate acetyltransferase | AFK18715.1 | 5 | 5 | 71.40 | 18.4 | 2.27e+006 |
| Hypothetical protein HFX_1239 | AFK18952.1 | 5 | 4 | 71.15 | 25.3 | 1.18e+006 |
| Lysophospholipase | AFK20242.1 | 5 | 4 | 71.01 | 16.9 | 3.92e+006 |
| Sec-independent protein translocase component TatC2 | AFK17925.1 | 4 | 4 | 70.77 | 6.6 | 1.19e+006 |
| Glutaryl-CoA dehydrogenase | AFK17952.2 | 4 | 4 | 70.40 | 16.7 | 1.02e+006 |
| Putative membrane-associated Zn-dependent protease | AFK19648.2 | 5 | 5 | 70.06 | 9 | 1.79e+006 |
| Hypothetical protein HFX_1766 | AFK19471.1 | 5 | 5 | 69.34 | 31 | 5.86e+006 |
| Hypothetical protein HFX_5285 (plasmid) | AFK21116.1 | 5 | 5 | 68.78 | 28.9 | 2.90e+006 |
| Tryptophanase | AFK17753.2 | 4 | 4 | 68.67 | 16 | 1.19e+006 |
| Electron transfer flavoprotein alpha subunit | AFK20418.1 | 5 | 5 | 68.64 | 10.2 | 2.69e+006 |
| Branched-chain-amino-acid aminotransferase | AFK18053.1 | 6 | 5 | 68.61 | 23.7 | 1.46e+006 |
| ABC-type branched-chain amino acid transport systems, substrate-binding protein | AFK20482.1 | 5 | 4 | 68.06 | 18.4 | 3.52e+006 |
| Transport ATPase (substrate arsenite) | AFK18406.1 | 4 | 4 | 67.76 | 14.3 | 2.08e+006 |
| Citrate (si)-synthase | AFK18167.1 | 5 | 5 | 67.65 | 14.7 | 1.53e+006 |
| ABC-type transport system ATP-binding/permease protein | AFK18212.1 | 5 | 5 | 66.57 | 7.9 | 1.64e+006 |
| Metalloprotease | AFK18012.1 | 5 | 4 | 66.45 | 14.1 | 2.53e+006 |
| Hypothetical protein HFX_6278 (plasmid) | AFK21400.1 | 5 | 4 | 65.90 | 41.7 | 5.38e+006 |
| Pyruvate--ferredoxin oxidoreductase, beta subunit | AFK19080.1 | 4 | 4 | 65.82 | 16.9 | 3.52e+006 |
| Stress response protein | AFK18921.1 | 5 | 5 | 65.71 | 43.4 | 4.15e+006 |
| Menaquinol--cytochrome-c reductase (cytochrome bc complex) cytochrome b/c subunit | AFK18533.1 | 5 | 4 | 65.61 | 17.7 | 7.01e+006 |
| Cytochrome bc1 complex cytochrome b/c subunit | AFK18497.1 | 3 | 3 | 47.39 | 11.4 | 4.65e+006 |
| Hypothetical protein HFX_1586 | AFK19292.1 | 5 | 5 | 65.47 | 14.3 | 2.45e+006 |
| Regulatory protein PrrC | AFK18961.1 | 4 | 3 | 65.26 | 29.1 | 3.40e+006 |
| Poly(3-hydroxyalkanoate) granule-associated protein(phasin) (plasmid) | AFK21052.1 | 9 | 4 | 65.02 | 24.6 | 1.80e+008 |
| ATP-dependent protease Lon | AFK18465.1 | 5 | 4 | 64.65 | 8 | 1.36e+006 |
| Oligopeptide ABC transporter ATPase component | AFK17803.1 | 4 | 4 | 64.50 | 15.1 | 2.36e+006 |
| Amino acid-binding protein (plasmid) | AFK21485.1 | 4 | 4 | 64.49 | 12.4 | 9.47e+005 |
| Hypothetical protein HFX_1692 | AFK19398.1 | 4 | 4 | 64.32 | 13.9 | 9.04e+005 |
| 3-oxoacyl-[acyl-carrier protein] reductase | AFK19230.1 | 5 | 4 | 64.27 | 21.4 | 5.48e+006 |
| Hypothetical protein HFX_2807 | AFK20480.1 | 5 | 4 | 64.16 | 32 | 4.34e+006 |
| Ornithine cyclodeaminase | AFK18155.1 | 6 | 4 | 63.75 | 17.7 | 2.29e+006 |
| Phosphoserine phosphatase | AFK20621.1 | 4 | 4 | 63.31 | 27.3 | 1.23e+006 |
| Oxidoreductase | AFK18675.1 | 4 | 4 | 62.73 | 21.8 | 1.25e+006 |
| Dihydroorotate dehydrogenase 2 | AFK20600.1 | 4 | 4 | 62.14 | 24.6 | 1.31e+006 |
| 2-methylcitrate dehydratase (plasmid) | AFK21002.1 | 4 | 4 | 61.74 | 14 | 7.86e+005 |
| Pyruvate kinase | AFK18496.2 | 5 | 5 | 61.41 | 10.7 | 1.32e+006 |
| Cellulase, endo-1,3(4)-beta-glucanase, peptidase M42 family protein / endoglucanase | AFK20447.1 | 4 | 4 | 61.20 | 12.7 | 1.14e+006 |
| Ribulose-bisphosphate carboxylase large chain | AFK18685.1 | 4 | 4 | 61.19 | 11.8 | 3.27e+006 |
| Hypothetical protein HFX_0697 | AFK18420.1 | 4 | 4 | 60.79 | 52.6 | 1.09e+007 |
| Glucose-1-phosphate thymidylyltransferase | AFK19833.1 | 4 | 4 | 60.67 | 11.5 | 1.58e+006 |
| Oxidoreductase (glycolate oxidase iron-sulfur subunit) | AFK19493.1 | 4 | 4 | 59.87 | 4 | 1.60e+006 |
| Electron transfer flavoprotein alpha subunit | AFK19622.1 | 5 | 4 | 59.51 | 13.9 | 1.60e+006 |
| Cytochrome c oxidase polypeptide I | AFK18663.1 | 5 | 4 | 59.40 | 7.6 | 1.94e+007 |
| Succinate--CoA ligase beta subunit (ADP-forming) | AFK20155.1 | 4 | 4 | 59.37 | 14.1 | 1.67e+006 |
| GMP synthase (glutamine-hydrolysing) | AFK20320.1 | 4 | 4 | 59.06 | 14.7 | 1.43e+006 |
| Gas-vesicle operon protein gvpA | AFK19402.1 | 4 | 3 | 59.05 | 57.6 | 2.38e+007 |
| Putative permease | AFK19808.1 | 4 | 4 | 58.65 | 21.2 | 9.09e+005 |
| RecJ-like exonuclease | AFK20553.1 | 4 | 4 | 58.46 | 8.1 | 1.17e+006 |
| Ferredoxin (2Fe-2S) | AFK19572.1 | 5 | 4 | 58.34 | 33.5 | 3.40e+006 |
| 3-hydroxyacyl-CoA dehydrogenase | AFK20502.1 | 4 | 4 | 58.29 | 9.2 | 1.76e+006 |
| Hypothetical protein HFX_2772 | AFK20448.1 | 6 | 4 | 58.24 | 53.9 | 8.97e+006 |
| Putative acylaminoacyl-peptidase | AFK18673.1 | 4 | 4 | 58.13 | 6.4 | 8.54e+005 |
| Oligopeptide ABC transporter permease protein | AFK17804.1 | 4 | 4 | 57.69 | 12.3 | 3.12e+006 |
| Stress response protein (plasmid) | AFK21518.1 | 4 | 4 | 57.68 | 17.6 | 2.61e+006 |
| Endoglucanase | AFK18519.1 | 4 | 4 | 57.53 | 16 | 1.33e+006 |
| NADH dehydrogenase-like complex, subunit I | AFK18698.1 | 5 | 4 | 57.46 | 40.5 | 2.28e+006 |
| Hypothetical protein HFX_0482 | AFK18214.1 | 3 | 3 | 57.12 | 32.8 | 9.92e+005 |
| NADH dehydrogenase, subunit M (ubiquinone) | AFK18703.1 | 5 | 4 | 56.88 | 7.6 | 8.35e+006 |
| FKBP-type peptidylprolyl isomerase 2 | AFK19430.2 | 3 | 3 | 56.73 | 12.2 | 6.92e+006 |
| Phosphonates ABC transporter permease protein | AFK19844.1 | 5 | 4 | 56.69 | 17.5 | 2.11e+006 |
| Acyl-CoA synthetase | AFK19702.1 | 4 | 4 | 56.61 | 5.9 | 1.89e+006 |
| Hypothetical protein HFX_5081 (plasmid) | AFK20916.2 | 4 | 3 | 56.60 | 11 | 9.97e+006 |
| Phosphoenolpyruvate carboxylase | AFK20315.2 | 4 | 4 | 56.38 | 5.2 | 6.23e+005 |
| Superoxide dismutase, Fe-Mn family | AFK20579.1 | 3 | 3 | 56.28 | 16 | 3.95e+006 |
| Superoxide dismutase, Fe-Mn family (plasmid) | AFK21528.1 | 3 | 3 | 53.69 | 16 | 2.63e+006 |
| Hypothetical protein HFX_1243 | AFK18956.1 | 6 | 3 | 56.23 | 10.6 | 1.85e+007 |
| Hypothetical protein HFX_5167 (plasmid) | AFK21001.1 | 4 | 3 | 55.83 | 19.2 | 2.86e+006 |
| FAD-dependent pyridine nucleotide-disulfide oxidoreductase | AFK17859.1 | 3 | 3 | 55.74 | 10.7 | 9.99e+005 |
| Dipeptidyl aminopeptidase/acylaminoacyl peptidase | AFK18522.1 | 4 | 4 | 55.49 | 9 | 9.17e+005 |
| Hypothetical protein HFX_1272 | AFK18985.1 | 4 | 4 | 55.39 | 25.2 | 4.83e+006 |
| Acyl-CoA synthetase, acetate--CoA ligase-like protein (ADP-forming) | AFK18716.1 | 5 | 4 | 55.14 | 7 | 1.25e+006 |
| Sulfatase-like protein | AFK19290.1 | 5 | 4 | 54.82 | 10.5 | 2.30e+006 |
| Citryl-CoA lyase (citrate lyase beta subunit / ATP citrate synthase beta subunit) | AFK19224.2 | 3 | 3 | 54.78 | 19.3 | 1.53e+005 |
| Succinate--CoA ligase, alpha subunit (ADP-forming) | AFK20154.1 | 3 | 3 | 53.94 | 19 | 7.92e+005 |
| NADH dehydrogenase, subunit H (ubiquinone) | AFK18697.1 | 4 | 3 | 53.53 | 9.4 | 2.65e+007 |
| Cyclase/dehydrase | AFK17928.1 | 4 | 4 | 52.72 | 15.8 | 1.83e+006 |
| N-methylhydantoinase B (ATP-hydrolyzing) (plasmid) | AFK21025.1 | 4 | 4 | 52.56 | 7.9 | 1.11e+006 |
| Immunogenic protein | AFK20638.1 | 4 | 3 | 52.20 | 12.7 | 2.29e+007 |
| Putative monovalent cation/H+ antiporter subunit E | AFK18782.1 | 4 | 4 | 52.14 | 17.6 | 9.07e+005 |
| Putative iron transport protein | AFK19393.1 | 4 | 3 | 51.75 | 11.3 | 2.63e+006 |
| Carboxypeptidase | AFK18120.1 | 4 | 4 | 51.71 | 10.7 | 1.06e+006 |
| Ferredoxin (2Fe-2S) | AFK20674.1 | 3 | 3 | 51.64 | 34.8 | 6.23e+006 |
| Putative patatin-like phospholipase (plasmid) | AFK21580.1 | 6 | 3 | 51.43 | 12.7 | 3.15e+006 |
| Hypothetical protein HFX_0118 | AFK17860.1 | 4 | 3 | 51.17 | 38.6 | 7.63e+005 |
| Hypothetical protein HFX_2876 | AFK20546.1 | 3 | 3 | 50.96 | 20.8 | 1.61e+007 |
| Hypothetical protein HFX_5226 (plasmid) | AFK21058.1 | 4 | 3 | 50.73 | 50.4 | 5.76e+007 |
| ABC-type glutamine/glutamate/polar amino acids transport system, ATP-binding protein | AFK20124.1 | 3 | 3 | 50.52 | 13.6 | 1.71e+006 |
| Hypothetical protein HFX_0195 | AFK17936.1 | 5 | 3 | 50.40 | 52.7 | 9.06e+006 |
| Hemerythrin HHE cation binding region (plasmid) | AFK20978.1 | 3 | 3 | 50.08 | 17.4 | 7.51e+005 |
| DMSO reductase family type II enzyme, heme b subunit (plasmid) | AFK20937.1 | 3 | 3 | 49.88 | 13.4 | 1.03e+006 |
| Hypothetical protein HFX_5272 (plasmid) | AFK21103.1 | 4 | 4 | 49.79 | 21.4 | 1.86e+006 |
| N-acyl-L-amino acid amidohydrolase | AFK19179.1 | 3 | 3 | 49.69 | 10.3 | 1.12e+006 |
| Aldehyde reductase | AFK18579.1 | 3 | 3 | 49.41 | 13 | 1.29e+006 |
| ABC-type dipeptide/oligopeptide/nickel transport system, substrate binding protein (plasmid) | AFK20740.1 | 3 | 3 | 49.36 | 7.4 | 1.20e+006 |
| 3-hydroxyacyl-CoA dehydrogenase | AFK19216.2 | 3 | 3 | 48.90 | 10.1 | 4.08e+006 |
| NADH dehydrogenase 32K chain-like protein | AFK17846.1 | 4 | 4 | 48.50 | 14.5 | 7.77e+005 |
| Hypothetical protein HFX_0292 | AFK18031.1 | 4 | 3 | 48.14 | 10.2 | 2.75e+006 |
| Menaquinol--cytochrome-c reductase (cytochrome bc complex) cytochrome b subunit | AFK18534.1 | 4 | 3 | 48.00 | 14.7 | 1.41e+007 |
| Putative cationic amino acid transport protein (plasmid) | AFK21046.1 | 3 | 3 | 47.99 | 6 | 6.45e+005 |
| Universal stress protein UspA-like protein | AFK19887.1 | 4 | 3 | 47.97 | 14.6 | 3.67e+006 |
| Branched-chain amino acid ABC transporter ATP-binding protein | AFK19533.1 | 3 | 3 | 47.53 | 17.6 | 6.57e+005 |
| ABC-type transport system ATP-binding protein | AFK18720.1 | 3 | 3 | 47.10 | 17.2 | 1.79e+006 |
| Hsp20-type chaperone | AFK18151.1 | 3 | 3 | 47.08 | 22.9 | 4.90e+006 |
| Ubiquinone/menaquinone biosynthesis methyltransferase | AFK18034.2 | 4 | 3 | 46.44 | 19.7 | 2.97e+006 |
| Cystathionine synthase/lyase (cystathionine gamma-synthase, cystathionine gamma-lyase, cystathionine beta-lyase) | AFK20603.1 | 3 | 3 | 46.44 | 8.9 | 9.79e+005 |
| Ribose ABC transporter ATP-binding protein | AFK19184.1 | 3 | 3 | 46.25 | 7.5 | 9.84e+005 |
| Putative inosine monophosphate dehydrogenase | AFK17799.1 | 4 | 4 | 46.15 | 21.8 | 1.01e+006 |
| Hypothetical protein HFX_0728 | AFK18451.1 | 3 | 3 | 46.02 | 28.3 | 1.04e+006 |
| Putative Rieske iron-sulfur protein (plasmid) | AFK20942.1 | 3 | 3 | 46.02 | 28.6 | 9.83e+005 |
| Hypothetical protein HFX_0671 | AFK18394.1 | 5 | 3 | 46.01 | 20.4 | 4.27e+006 |
| Menaquinol--cytochrome-c reductase | AFK20314.1 | 5 | 3 | 45.96 | 13.5 | 1.51e+007 |
| N-methylhydantoinase A (ATP-hydrolyzing) (plasmid) | AFK21026.1 | 3 | 3 | 45.48 | 6.7 | 4.55e+005 |
| Signal sequence peptidase | AFK17747.2 | 3 | 3 | 45.42 | 12.3 | 1.12e+006 |
| Nucleoside diphosphate kinase | AFK20428.1 | 3 | 3 | 45.38 | 26.6 | 2.53e+006 |
| Thiosulfate sulfurtransferase | AFK17767.2 | 3 | 3 | 45.27 | 15 | 3.33e+006 |
| Glycosyltransferase (dolichyl-phosphate beta-D-mannosyltransferase) | AFK19286.1 | 3 | 3 | 45.17 | 13.9 | 1.46e+006 |
| Ubiquinone biosynthesis transmembrane protein | AFK19999.1 | 3 | 3 | 45.02 | 6.4 | 8.40e+005 |
| Hypothetical protein HFX_0662 | AFK18386.1 | 3 | 3 | 44.91 | 5.7 | 8.08e+005 |
| ABC-type transport system involved in Fe-S cluster assembly, permease protein II | AFK18551.1 | 4 | 3 | 44.82 | 10.9 | 1.43e+006 |
| Nonhistone chromosomal protein | AFK20598.1 | 3 | 3 | 44.68 | 25 | 8.03e+005 |
| Hypothetical protein HFX_0680 | AFK18403.2 | 3 | 3 | 44.54 | 13.3 | 4.58e+005 |
| Fumarate hydratase, class II | AFK20567.2 | 3 | 3 | 44.50 | 7 | 8.24e+005 |
| MaoC family protein (plasmid) | AFK21063.1 | 3 | 3 | 44.44 | 35.2 | 4.86e+006 |
| Poly(3-hydroxyalkanoate) synthase subunit PhaC | AFK20356.1 | 3 | 3 | 44.39 | 10.4 | 8.06e+005 |
| NADH dehydrogenase, subunit L (ubiquinone) | AFK18702.1 | 4 | 3 | 44.06 | 4.4 | 4.01e+006 |
| Hypothetical protein HFX_2976 | AFK20640.1 | 3 | 3 | 43.98 | 3 | 1.98e+006 |
| Hypothetical protein HFX_1953 | AFK19649.1 | 3 | 3 | 43.79 | 5.8 | 5.12e+005 |
| Signal transduction histidine kinase / two-component system, OmpR family, sensor histidine kinase CreC (plasmid) | AFK21044.1 | 3 | 3 | 43.76 | 7.8 | 8.42e+005 |
| Quinoprotein glucose dehydrogenase | AFK18721.1 | 3 | 3 | 43.20 | 7.6 | 9.47e+005 |
| Hypothetical protein HFX_2039 | AFK19731.2 | 3 | 3 | 43.01 | 11.7 | 7.84e+005 |
| Sialidase-1 | AFK20474.1 | 3 | 3 | 42.72 | 9 | 6.84e+005 |
| Hypothetical protein HFX_2758 | AFK20435.1 | 3 | 3 | 42.61 | 17.3 | 7.09e+006 |
| Hypothetical protein HFX_0661 | AFK18385.1 | 4 | 3 | 42.42 | 27.5 | 1.20e+007 |
| 4Fe-S protein | AFK19488.1 | 3 | 3 | 42.38 | 5.7 | 4.65e+005 |
| Acyl-CoA synthetase | AFK19839.1 | 3 | 3 | 42.38 | 7.9 | 7.86e+005 |
| Prefoldin beta subunit | AFK18347.1 | 2 | 2 | 41.96 | 25.8 | 2.17e+006 |
| Putative sugar ABC transporter permease protein | AFK20384.1 | 3 | 3 | 41.62 | 10.6 | 3.30e+006 |
| Hypothetical protein HFX_2182 | AFK19871.1 | 3 | 3 | 41.51 | 12.1 | 8.81e+005 |
| ABC-type copper transport system, permease protein | AFK20684.1 | 3 | 3 | 41.47 | 13.3 | 1.43e+006 |
| Aldehyde dehydrogenase (NAD+) (plasmid) | AFK21494.1 | 3 | 3 | 41.19 | 9.1 | 5.80e+005 |
| Acyl-CoA dehydrogenase | AFK18868.1 | 3 | 3 | 41.02 | 11.3 | 4.33e+005 |
| Universal stress protein | AFK18810.1 | 3 | 2 | 40.97 | 19 | 7.73e+005 |
| Carbonic anhydrase | AFK18667.1 | 3 | 3 | 40.96 | 16 | 7.23e+005 |
| Hypothetical protein HFX_6067 (plasmid) | AFK21194.1 | 4 | 3 | 40.89 | 8.4 | 1.33e+006 |
| NADH dehydrogenase/oxidoreductase-like protein | AFK19950.1 | 3 | 2 | 40.57 | 10.2 | 3.78e+006 |
| Hypothetical protein HFX_1684 | AFK19390.1 | 2 | 2 | 40.52 | 21.6 | 7.58e+006 |
| Menaquinone biosynthesis methyltransferase UbiE | AFK20539.1 | 3 | 3 | 40.30 | 21.2 | 8.27e+005 |
| Hypothetical protein HFX_2180 | AFK19869.1 | 4 | 3 | 40.21 | 8.7 | 3.36e+006 |
| A-type ATP synthase subunit F | AFK18040.1 | 3 | 2 | 39.37 | 36.7 | 4.30e+006 |
| Hypothetical protein HFX_0016 | AFK17760.1 | 3 | 3 | 39.00 | 15.1 | 1.23e+006 |
| Hypothetical protein HFX_1141 | AFK18857.1 | 2 | 2 | 38.52 | 31.6 | 1.92e+006 |
| F420-dependent NADP reductase | AFK18135.1 | 2 | 2 | 38.47 | 12.1 | 9.22e+005 |
| Phosphonates ABC transporter permease protein | AFK19845.1 | 3 | 3 | 38.36 | 17.4 | 1.78e+006 |
| UpsA domain-containing protein | AFK19975.2 | 4 | 3 | 38.25 | 26.7 | 1.44e+006 |
| Short-chain dehydrogenase / reductase SDR / glucose 1-dehydrogenase | AFK20158.1 | 3 | 3 | 37.90 | 16.1 | 2.77e+005 |
| Hypothetical protein HFX_0214 | AFK17955.1 | 4 | 2 | 37.89 | 8.4 | 5.22e+006 |
| Oxidoreductase (geranylgeranyl hydrogenase-like protein) | AFK19602.1 | 3 | 3 | 37.67 | 8.7 | 4.62e+005 |
| Na+/H+ antiporter | AFK19178.1 | 2 | 2 | 37.56 | 6.3 | 2.33e+005 |
| Methylmalonyl-CoA decarboxylase alpha chain | AFK20161.1 | 3 | 3 | 37.55 | 7.5 | 6.99e+005 |
| 2-oxoglutarate ferredoxin oxidoreductase, subunit beta | AFK18582.1 | 3 | 3 | 37.45 | 9.7 | 8.79e+005 |
| S-adenosylmethionine synthetase | AFK19445.1 | 2 | 2 | 37.11 | 6.2 | 2.25e+005 |
| Hypothetical protein HFX_0940 | AFK18659.1 | 2 | 2 | 37.03 | 9.4 | 2.83e+006 |
| Glutamate-1-semialdehyde aminotransferase | AFK17832.1 | 2 | 2 | 36.77 | 6.2 | 7.08e+005 |
| Halocyanin precursor-like protein | AFK18536.1 | 2 | 2 | 36.71 | 15.8 | 4.95e+006 |
| Halocyanin precursor-like protein | AFK18518.1 | 2 | 2 | 36.71 | 16.5 | 4.95e+006 |
| Peptidyl-prolyl cis-trans isomerase B (cyclophilin B) | AFK19961.1 | 2 | 2 | 36.67 | 23.8 | 1.07e+006 |
| Acyl-CoA synthetase | AFK19349.1 | 3 | 3 | 36.61 | 5.4 | 4.48e+005 |
| ABC-type glutamine/glutamate/polar amino acids transport system, substrate-binding protein | AFK20126.1 | 3 | 2 | 36.59 | 10.5 | 2.82e+006 |
| Hypothetical protein HFX_1589 | AFK19295.1 | 3 | 3 | 36.55 | 6.6 | 1.22e+006 |
| NADPH-dependent FMN reductase | AFK20556.2 | 2 | 2 | 35.75 | 24.4 | 1.88e+005 |
| Malate dehydrogenase | AFK20690.2 | 2 | 2 | 35.73 | 11.1 | 4.78e+005 |
| Ferredoxin:NAD+ oxidoreductase | AFK20041.1 | 2 | 2 | 35.55 | 7.5 | 8.11e+005 |
| Hypothetical protein HFX_0639 | AFK18363.1 | 2 | 2 | 35.46 | 20.9 | 1.13e+006 |
| Inorganic pyrophosphatase | AFK18410.1 | 3 | 3 | 35.22 | 20.3 | 1.74e+006 |
| ABC transporter ATP-binding protein | AFK18549.1 | 2 | 2 | 35.10 | 7.6 | 7.31e+005 |
| Gas-vesicle operon protein gvpF | AFK19405.1 | 2 | 2 | 34.77 | 15 | 3.80e+005 |
| Hypothetical protein HFX_1000 | AFK18718.1 | 2 | 2 | 34.61 | 22.3 | 2.43e+006 |
| Hypothetical protein HFX_1656 | AFK19362.1 | 2 | 2 | 34.58 | 20 | 6.82e+005 |
| Hypothetical protein HFX_0491 | AFK18222.1 | 3 | 2 | 34.15 | 14 | 4.21e+006 |
| Dipeptide ABC transporter permease | AFK17805.1 | 3 | 2 | 34.12 | 10 | 1.36e+006 |
| Hypothetical protein HFX_6075 (plasmid) | AFK21202.1 | 2 | 2 | 34.07 | 22.6 | 1.17e+007 |
| Copper-binding plastocyanin like protein (plasmid) | AFK20927.1 | 3 | 2 | 34.00 | 20.8 | 1.90e+006 |
| hypothetical protein HFX_0366 | AFK18102.2 | 2 | 2 | 33.98 | 31.3 | 1.16e+006 |
| Putative intracellular protease | AFK19063.1 | 2 | 2 | 33.91 | 8 | 5.92e+006 |
| Hypothetical protein HFX_1448 | AFK19156.1 | 4 | 2 | 33.89 | 12.7 | 3.62e+006 |
| Hypothetical protein HFX_0620 | AFK18344.1 | 3 | 3 | 33.68 | 5.4 | 6.55e+005 |
| Phosphate ABC transporter permease | AFK20070.1 | 2 | 2 | 33.64 | 8.1 | 1.06e+006 |
| Hypothetical protein HFX_0856 | AFK18577.1 | 3 | 3 | 33.53 | 7.9 | 6.35e+005 |
| Proteasome beta subunit | AFK19329.1 | 2 | 2 | 33.52 | 13 | 1.34e+006 |
| Hypothetical protein HFX_2040 | AFK19732.2 | 2 | 2 | 33.39 | 7.6 | 6.06e+005 |
| Ferritin-like protein | AFK17959.1 | 2 | 2 | 33.32 | 30 | 9.40e+005 |
| ABC-type transport system ATP-binding protein | AFK20086.1 | 2 | 2 | 33.30 | 14.2 | 1.01e+006 |
| Protein of unknown function DUF1486 | AFK20429.1 | 2 | 2 | 33.08 | 15.7 | 8.55e+005 |
| Electron transfer flavoprotein alpha-subunit | AFK18029.1 | 2 | 2 | 32.99 | 13.5 | 9.38e+005 |
| Putative membrane protein | AFK17823.1 | 2 | 2 | 32.84 | 15.7 | 6.01e+005 |
| Cell division protein ftsZ | AFK19949.2 | 2 | 2 | 32.84 | 7.3 | 5.99e+005 |
| Copper-transporting ATPase CopA (plasmid) | AFK21064.1 | 2 | 2 | 32.81 | 3.5 | 6.92e+005 |
| Diphosphomevalonate decarboxylase | AFK19194.1 | 2 | 2 | 32.76 | 6.1 | 8.48e+005 |
| Aminopeptidase | AFK18528.1 | 3 | 2 | 32.71 | 5.9 | 8.58e+005 |
| Short chain dehydrogenase | AFK20232.1 | 2 | 2 | 32.68 | 11.7 | 7.26e+005 |
| ArsR family regulatory protein | AFK19320.1 | 3 | 2 | 32.60 | 18.9 | 1.14e+006 |
| Glutamate dehydrogenase (NAD(P)+) | AFK19867.1 | 2 | 2 | 32.45 | 8.6 | 8.21e+005 |
| Histidine ammonia-lyase (plasmid) | AFK21453.1 | 2 | 2 | 32.44 | 4.5 | 5.54e+005 |
| Mechanosensitive ion channel | AFK19459.1 | 2 | 2 | 32.39 | 7.7 | 1.15e+006 |
| Anthranilate phosphoribosyltransferase | AFK19965.1 | 2 | 2 | 32.31 | 8.7 | 1.10e+006 |
| Dodecin | AFK20375.1 | 2 | 2 | 32.09 | 30.8 | 5.62e+006 |
| Methanol dehydrogenase regulatory protein | AFK18978.1 | 2 | 2 | 32.07 | 7.7 | 8.26e+005 |
| Malate dehydrogenase (oxaloacetate-decarboxylating)(NADP+) | AFK20129.1 | 3 | 2 | 32.03 | 4.6 | 6.87e+005 |
| Ribose-1,5-bisphosphate isomerase (ribulose-bisphosphate forming) | AFK18682.1 | 3 | 3 | 31.95 | 9.7 | 5.21e+005 |
| NADPH2:quinone reductase | AFK20343.1 | 2 | 2 | 31.86 | 7.5 | 8.86e+005 |
| Ribose 5-phosphate isomerase | AFK17935.1 | 2 | 2 | 31.62 | 11.7 | 2.71e+005 |
| Thioredoxin | AFK18964.1 | 2 | 2 | 31.32 | 10.4 | 1.13e+006 |
| Heat shock protein HtpX | AFK17854.2 | 3 | 2 | 31.31 | 9.5 | 1.52e+006 |
| Hypothetical protein HFX_0813 | AFK18535.1 | 3 | 2 | 31.25 | 24.4 | 6.76e+005 |
| Methionine aminopeptidase | AFK20296.1 | 3 | 2 | 31.07 | 5.7 | 4.07e+006 |
| Serine protease inhibitor family protein | AFK20013.1 | 2 | 2 | 30.71 | 4.8 | 1.24e+006 |
| Hypothetical protein HFX_0694 | AFK18417.1 | 2 | 2 | 30.67 | 36.4 | 3.81e+005 |
| Hypothetical protein HFX_1182 | AFK18895.1 | 2 | 2 | 30.59 | 16.6 | 1.11e+006 |
| PLP-dependent aminotransferase (aspartate aminotransferase) | AFK19365.1 | 2 | 2 | 30.48 | 7.2 | 5.33e+005 |
| Glycine dehydrogenase subunit 2 | AFK20092.1 | 2 | 2 | 30.43 | 8.8 | 2.40e+005 |
| Endoribonuclease L-PSP | AFK18161.1 | 2 | 2 | 30.31 | 25 | 6.46e+005 |
| Na+/Ca2+-exchanging protein | AFK18397.1 | 2 | 2 | 30.27 | 6.6 | 5.76e+005 |
| Halocyanin precursor-like protein | AFK18843.1 | 2 | 2 | 30.11 | 15.6 | 2.93e+006 |
| Cytochrome b/b6 (plasmid) | AFK20941.1 | 2 | 2 | 30.00 | 3.9 | 1.36e+006 |

Table S3. Representative proteins identified in the micelles with Nar and Nir activities obtained after the use of DEAE-Sepharose CL-6B from 3 LC-MS/MS runs.

| **Protein name** | **Database**  **Accession** | **Spectra** | **Distinct**  **Peptides** | **Distinct**  **Summed**  **MS/MS Search**  **Score** | **% AA**  **Coverage** | **Total Protein Spectral Intensity** |
| --- | --- | --- | --- | --- | --- | --- |
| NADH dehydrogenase, subunit CD (ubiquinone) | [AFK18696.1](http://smserver.sti.ua.es/millhtml/SM_instruct/servadmn.htm#update_acclinks?AFK18696.1) | 40 | 28 | 473.42 | [63.7](http://smserver.sti.ua.es/millbin/msdigest.cgi?missed_cleavages=2&msparams_dir=msparams_mill/&hide_protein_sequence=2&database=PA_haloferax_mediterranei_genbank&seqdb_dir=D:\SeqDB\&enzyme=Trypsin&access_method=Accession+Number&accession_num=AFK18696.1&coverage_map=0+20+15+22+59+7+17+39+57+9+42+28+16+7+104+13+37+6+8+51) | 2.23e+008 |
| NADH dehydrogenase, subunit D (ubiquinone) | [AFK18684.1](http://smserver.sti.ua.es/millhtml/SM_instruct/servadmn.htm#update_acclinks?AFK18684.1) | 6 | 5 | 86.21 | [7.7](http://smserver.sti.ua.es/millbin/msdigest.cgi?missed_cleavages=2&msparams_dir=msparams_mill/&hide_protein_sequence=2&database=PA_haloferax_mediterranei_genbank&seqdb_dir=D:\SeqDB\&enzyme=Trypsin&access_method=Accession+Number&accession_num=AFK18684.1&coverage_map=0+312+16+7+18+51+9+141) | 6.07e+007 |
| A-type ATP synthase subunit A | [AFK18041.1](http://smserver.sti.ua.es/millhtml/SM_instruct/servadmn.htm#update_acclinks?AFK18041.1) | 34 | 22 | 410.90 | [47](http://smserver.sti.ua.es/millbin/msdigest.cgi?missed_cleavages=2&msparams_dir=msparams_mill/&hide_protein_sequence=2&database=PA_haloferax_mediterranei_genbank&seqdb_dir=D:\SeqDB\&enzyme=Trypsin&access_method=Accession+Number&accession_num=AFK18041.1&coverage_map=0+10+16+79+19+96+10+23+86+11+24+40+12+35+37+3+30+7+19+3+23+3) | 1.49e+008 |
| Nitrous-oxide reductase (plasmid) | [AFK20926.1](http://smserver.sti.ua.es/millhtml/SM_instruct/servadmn.htm#update_acclinks?AFK20926.1) | 29 | 23 | 381.40 | [38.6](http://smserver.sti.ua.es/millbin/msdigest.cgi?missed_cleavages=2&msparams_dir=msparams_mill/&hide_protein_sequence=2&database=PA_haloferax_mediterranei_genbank&seqdb_dir=D:\SeqDB\&enzyme=Trypsin&access_method=Accession+Number&accession_num=AFK20926.1&coverage_map=0+122+8+14+34+108+20+7+9+8+25+18+19+16+78+5+21+34+11+9+28+69+6+1) | 3.24e+008 |
| Succinate dehydrogenase, subunit A (flavoprotein) | [AFK20495.1](http://smserver.sti.ua.es/millhtml/SM_instruct/servadmn.htm#update_acclinks?AFK20495.1) | 28 | 19 | 361.17 | [48.9](http://smserver.sti.ua.es/millbin/msdigest.cgi?missed_cleavages=2&msparams_dir=msparams_mill/&hide_protein_sequence=2&database=PA_haloferax_mediterranei_genbank&seqdb_dir=D:\SeqDB\&enzyme=Trypsin&access_method=Accession+Number&accession_num=AFK20495.1&coverage_map=1+17+22+15+14+16+23+35+4+135+5+22+73+14+93+19+23+29+56) | 1.19e+008 |
| A-type ATP synthase subunit B | [AFK18042.1](http://smserver.sti.ua.es/millhtml/SM_instruct/servadmn.htm#update_acclinks?AFK18042.1) | 30 | 19 | 352.32 | [59.8](http://smserver.sti.ua.es/millbin/msdigest.cgi?missed_cleavages=2&msparams_dir=msparams_mill/&hide_protein_sequence=2&database=PA_haloferax_mediterranei_genbank&seqdb_dir=D:\SeqDB\&enzyme=Trypsin&access_method=Accession+Number&accession_num=AFK18042.1&coverage_map=0+39+25+12+65+3+20+45+15+18+19+13+16+44+60+9+39+5+21) | 2.35e+008 |
| Poly(3-hydroxyalkanoate) synthase subunit PhaC (plasmid) | [AFK21054.1](http://smserver.sti.ua.es/millhtml/SM_instruct/servadmn.htm#update_acclinks?AFK21054.1) | 27 | 17 | 331.66 | [54.8](http://smserver.sti.ua.es/millbin/msdigest.cgi?missed_cleavages=2&msparams_dir=msparams_mill/&hide_protein_sequence=2&database=PA_haloferax_mediterranei_genbank&seqdb_dir=D:\SeqDB\&enzyme=Trypsin&access_method=Accession+Number&accession_num=AFK21054.1&coverage_map=0+13+11+7+11+20+74+32+60+3+55+56+16+91+43) | 7.84e+007 |
| Dipeptide ABC transporter dipeptide-binding protein | [AFK17806.1](http://smserver.sti.ua.es/millhtml/SM_instruct/servadmn.htm#update_acclinks?AFK17806.1) | 23 | 18 | 316.83 | [42.6](http://smserver.sti.ua.es/millbin/msdigest.cgi?missed_cleavages=2&msparams_dir=msparams_mill/&hide_protein_sequence=2&database=PA_haloferax_mediterranei_genbank&seqdb_dir=D:\SeqDB\&enzyme=Trypsin&access_method=Accession+Number&accession_num=AFK17806.1&coverage_map=0+60+35+57+37+64+21+22+36+39+9+4+20+5+9+5+22+23+12+56+12+10+49+8) | 9.91e+007 |
| Dipeptide ABC transporter ATP-binding protein | [AFK18330.2](http://smserver.sti.ua.es/millhtml/SM_instruct/servadmn.htm#update_acclinks?AFK18330.2) | 24 | 17 | 313.78 | [51](http://smserver.sti.ua.es/millbin/msdigest.cgi?missed_cleavages=2&msparams_dir=msparams_mill/&hide_protein_sequence=2&database=PA_haloferax_mediterranei_genbank&seqdb_dir=D:\SeqDB\&enzyme=Trypsin&access_method=Accession+Number&accession_num=AFK18330.2&coverage_map=0+68+58+4+17+52+126+53+25+93+51+10+15) | 1.47e+008 |
| Thermosome, beta subunit | [AFK18158.2](http://smserver.sti.ua.es/millhtml/SM_instruct/servadmn.htm#update_acclinks?AFK18158.2) | 24 | 19 | 306.80 | [48.3](http://smserver.sti.ua.es/millbin/msdigest.cgi?missed_cleavages=2&msparams_dir=msparams_mill/&hide_protein_sequence=2&database=PA_haloferax_mediterranei_genbank&seqdb_dir=D:\SeqDB\&enzyme=Trypsin&access_method=Accession+Number&accession_num=AFK18158.2&coverage_map=0+4+16+32+20+39+17+8+22+15+9+88+10+2+28+1+6+11+21+5+68+11+35+34+16+36) | 3.79e+007 |
| Nucleoside-binding protein | [AFK19185.1](http://smserver.sti.ua.es/millhtml/SM_instruct/servadmn.htm#update_acclinks?AFK19185.1) | 22 | 17 | 298.10 | [64.4](http://smserver.sti.ua.es/millbin/msdigest.cgi?missed_cleavages=2&msparams_dir=msparams_mill/&hide_protein_sequence=2&database=PA_haloferax_mediterranei_genbank&seqdb_dir=D:\SeqDB\&enzyme=Trypsin&access_method=Accession+Number&accession_num=AFK19185.1&coverage_map=0+84+23+1+20+4+61+2+45+2+32+2+45+38+15) | 1.05e+008 |
| A-type ATP synthase subunit C | [AFK18039.1](http://smserver.sti.ua.es/millhtml/SM_instruct/servadmn.htm#update_acclinks?AFK18039.1) | 21 | 16 | 291.58 | [65.5](http://smserver.sti.ua.es/millbin/msdigest.cgi?missed_cleavages=2&msparams_dir=msparams_mill/&hide_protein_sequence=2&database=PA_haloferax_mediterranei_genbank&seqdb_dir=D:\SeqDB\&enzyme=Trypsin&access_method=Accession+Number&accession_num=AFK18039.1&coverage_map=0+20+10+4+61+15+28+45+17+3+10+10+36+9+50+14+16) | 9.91e+007 |
| Periplasmic solute binding protein | [AFK20085.1](http://smserver.sti.ua.es/millhtml/SM_instruct/servadmn.htm#update_acclinks?AFK20085.1) | 19 | 16 | 290.21 | [58.9](http://smserver.sti.ua.es/millbin/msdigest.cgi?missed_cleavages=2&msparams_dir=msparams_mill/&hide_protein_sequence=2&database=PA_haloferax_mediterranei_genbank&seqdb_dir=D:\SeqDB\&enzyme=Trypsin&access_method=Accession+Number&accession_num=AFK20085.1&coverage_map=0+78+94+2+22+7+17+3+17+27+9+14+37+13+15+3) | 5.40e+007 |
| A-type ATP synthase subunit I | [AFK18036.1](http://smserver.sti.ua.es/millhtml/SM_instruct/servadmn.htm#update_acclinks?AFK18036.1) | 22 | 15 | 282.21 | [28.1](http://smserver.sti.ua.es/millbin/msdigest.cgi?missed_cleavages=2&msparams_dir=msparams_mill/&hide_protein_sequence=2&database=PA_haloferax_mediterranei_genbank&seqdb_dir=D:\SeqDB\&enzyme=Trypsin&access_method=Accession+Number&accession_num=AFK18036.1&coverage_map=0+16+46+8+12+2+24+51+18+55+79+291+8+88+9+7+9+6) | 1.24e+008 |
| SPFH domain, Band 7 family protein | [AFK18490.1](http://smserver.sti.ua.es/millhtml/SM_instruct/servadmn.htm#update_acclinks?AFK18490.1) | 23 | 15 | 274.67 | [49](http://smserver.sti.ua.es/millbin/msdigest.cgi?missed_cleavages=2&msparams_dir=msparams_mill/&hide_protein_sequence=2&database=PA_haloferax_mediterranei_genbank&seqdb_dir=D:\SeqDB\&enzyme=Trypsin&access_method=Accession+Number&accession_num=AFK18490.1&coverage_map=0+42+9+1+30+6+14+27+15+9+12+7+20+2+11+22+17+22+21+26+60+53) | 9.63e+007 |
| SPFH domain, Band 7 family protein | [AFK17777.1](http://smserver.sti.ua.es/millhtml/SM_instruct/servadmn.htm#update_acclinks?AFK17777.1) | 5 | 4 | 63.54 | [10.8](http://smserver.sti.ua.es/millbin/msdigest.cgi?missed_cleavages=2&msparams_dir=msparams_mill/&hide_protein_sequence=2&database=PA_haloferax_mediterranei_genbank&seqdb_dir=D:\SeqDB\&enzyme=Trypsin&access_method=Accession+Number&accession_num=AFK17777.1&coverage_map=0+155+21+24+12+29+11+153) | 6.38e+006 |
| Nitrate reductase alpha chain (plasmid) | [AFK20939.1](http://smserver.sti.ua.es/millhtml/SM_instruct/servadmn.htm#update_acclinks?AFK20939.1) | 17 | 17 | 269.39 | [24.7](http://smserver.sti.ua.es/millbin/msdigest.cgi?missed_cleavages=2&msparams_dir=msparams_mill/&hide_protein_sequence=2&database=PA_haloferax_mediterranei_genbank&seqdb_dir=D:\SeqDB\&enzyme=Trypsin&access_method=Accession+Number&accession_num=AFK20939.1&coverage_map=0+140+11+35+22+84+11+49+10+53+22+65+27+50+10+35+10+46+7+5+8+12+27+33+22+36+20+18+37+79) | 2.09e+007 |
| Thermosome, alpha subunit | [AFK17883.2](http://smserver.sti.ua.es/millhtml/SM_instruct/servadmn.htm#update_acclinks?AFK17883.2) | 18 | 15 | 268.32 | [44.4](http://smserver.sti.ua.es/millbin/msdigest.cgi?missed_cleavages=2&msparams_dir=msparams_mill/&hide_protein_sequence=2&database=PA_haloferax_mediterranei_genbank&seqdb_dir=D:\SeqDB\&enzyme=Trypsin&access_method=Accession+Number&accession_num=AFK17883.2&coverage_map=0+20+15+17+20+66+20+15+74+28+37+17+22+36+10+33+35+34+16+45) | 2.54e+007 |
| Gas-vesicle operon protein gvpC | [AFK19401.1](http://smserver.sti.ua.es/millhtml/SM_instruct/servadmn.htm#update_acclinks?AFK19401.1) | 23 | 13 | 263.40 | [49](http://smserver.sti.ua.es/millbin/msdigest.cgi?missed_cleavages=2&msparams_dir=msparams_mill/&hide_protein_sequence=2&database=PA_haloferax_mediterranei_genbank&seqdb_dir=D:\SeqDB\&enzyme=Trypsin&access_method=Accession+Number&accession_num=AFK19401.1&coverage_map=0+14+126+49+30+7+14+124+17) | 7.05e+007 |
| Sugar ABC transporter substrate binding protein | [AFK20386.1](http://smserver.sti.ua.es/millhtml/SM_instruct/servadmn.htm#update_acclinks?AFK20386.1) | 20 | 14 | 248.53 | [44](http://smserver.sti.ua.es/millbin/msdigest.cgi?missed_cleavages=2&msparams_dir=msparams_mill/&hide_protein_sequence=2&database=PA_haloferax_mediterranei_genbank&seqdb_dir=D:\SeqDB\&enzyme=Trypsin&access_method=Accession+Number&accession_num=AFK20386.1&coverage_map=0+86+84+53+9+33+24+3+45+41+25+48+38+22) | 9.18e+007 |
| Poly(3-hydroxyalkanoate) synthase subunit PhaE (plasmid) | [AFK21053.1](http://smserver.sti.ua.es/millhtml/SM_instruct/servadmn.htm#update_acclinks?AFK21053.1) | 30 | 11 | 238.40 | [71.4](http://smserver.sti.ua.es/millbin/msdigest.cgi?missed_cleavages=2&msparams_dir=msparams_mill/&hide_protein_sequence=2&database=PA_haloferax_mediterranei_genbank&seqdb_dir=D:\SeqDB\&enzyme=Trypsin&access_method=Accession+Number&accession_num=AFK21053.1&coverage_map=0+5+21+36+100+11+9) | 4.58e+008 |
| Iron-sulfur protein (4Fe-4S) | [AFK17960.2](http://smserver.sti.ua.es/millhtml/SM_instruct/servadmn.htm#update_acclinks?AFK17960.2) | 18 | 14 | 237.96 | [30.2](http://smserver.sti.ua.es/millbin/msdigest.cgi?missed_cleavages=2&msparams_dir=msparams_mill/&hide_protein_sequence=2&database=PA_haloferax_mediterranei_genbank&seqdb_dir=D:\SeqDB\&enzyme=Trypsin&access_method=Accession+Number&accession_num=AFK17960.2&coverage_map=0+46+18+230+10+20+7+88+36+10+25+38+52+13+11+31+30+1+24+14) | 3.69e+007 |
| PBS lyase HEAT-like repeat protein | [AFK18736.1](http://smserver.sti.ua.es/millhtml/SM_instruct/servadmn.htm#update_acclinks?AFK18736.1) | 17 | 13 | 233.77 | [39](http://smserver.sti.ua.es/millbin/msdigest.cgi?missed_cleavages=2&msparams_dir=msparams_mill/&hide_protein_sequence=2&database=PA_haloferax_mediterranei_genbank&seqdb_dir=D:\SeqDB\&enzyme=Trypsin&access_method=Accession+Number&accession_num=AFK18736.1&coverage_map=0+78+22+96+16+48+10+12+24+4+26+5+53+6+13+7) | 1.04e+008 |
| Serine protease (plasmid) | [AFK21203.1](http://smserver.sti.ua.es/millhtml/SM_instruct/servadmn.htm#update_acclinks?AFK21203.1) | 17 | 15 | 233.41 | [37.6](http://smserver.sti.ua.es/millbin/msdigest.cgi?missed_cleavages=2&msparams_dir=msparams_mill/&hide_protein_sequence=2&database=PA_haloferax_mediterranei_genbank&seqdb_dir=D:\SeqDB\&enzyme=Trypsin&access_method=Accession+Number&accession_num=AFK21203.1&coverage_map=0+15+11+2+38+88+14+95+14+6+20+42+28+2+33+33+8+23+26+46+25+7) | 5.11e+007 |
| Halocyanin hcpG | [AFK18949.1](http://smserver.sti.ua.es/millhtml/SM_instruct/servadmn.htm#update_acclinks?AFK18949.1) | 15 | 13 | 224.11 | [31.2](http://smserver.sti.ua.es/millbin/msdigest.cgi?missed_cleavages=2&msparams_dir=msparams_mill/&hide_protein_sequence=2&database=PA_haloferax_mediterranei_genbank&seqdb_dir=D:\SeqDB\&enzyme=Trypsin&access_method=Accession+Number&accession_num=AFK18949.1&coverage_map=0+70+12+30+17+5+22+2+17+139+19+33+35+7+21+78+15+6+36+19+62+173) | 1.96e+007 |
| Dimethylsulfoxide reductase | [AFK19896.1](http://smserver.sti.ua.es/millhtml/SM_instruct/servadmn.htm#update_acclinks?AFK19896.1) | 14 | 13 | 217.75 | [21.3](http://smserver.sti.ua.es/millbin/msdigest.cgi?missed_cleavages=2&msparams_dir=msparams_mill/&hide_protein_sequence=2&database=PA_haloferax_mediterranei_genbank&seqdb_dir=D:\SeqDB\&enzyme=Trypsin&access_method=Accession+Number&accession_num=AFK19896.1&coverage_map=0+45+15+24+10+27+37+103+14+37+17+320+43+17+13+5+9+13+19+61) | 1.14e+007 |
| Dimethylsulfoxide reductase | [AFK19898.1](http://smserver.sti.ua.es/millhtml/SM_instruct/servadmn.htm#update_acclinks?AFK19898.1) | 6 | 6 | 88.59 | [7.9](http://smserver.sti.ua.es/millbin/msdigest.cgi?missed_cleavages=2&msparams_dir=msparams_mill/&hide_protein_sequence=2&database=PA_haloferax_mediterranei_genbank&seqdb_dir=D:\SeqDB\&enzyme=Trypsin&access_method=Accession+Number&accession_num=AFK19898.1&coverage_map=0+85+10+157+24+11+10+16+8+166+14+330) | 4.19e+006 |
| Dipeptide/oligopeptide/nickel ABC transporter periplasmic substrate-binding protein | [AFK18790.1](http://smserver.sti.ua.es/millhtml/SM_instruct/servadmn.htm#update_acclinks?AFK18790.1) | 14 | 13 | 212.68 | [29.7](http://smserver.sti.ua.es/millbin/msdigest.cgi?missed_cleavages=2&msparams_dir=msparams_mill/&hide_protein_sequence=2&database=PA_haloferax_mediterranei_genbank&seqdb_dir=D:\SeqDB\&enzyme=Trypsin&access_method=Accession+Number&accession_num=AFK18790.1&coverage_map=0+80+12+77+25+38+20+57+18+42+9+3+28+20+18+3+7+8+25+59+9+47+13) | 1.59e+007 |
| Oxidoreductase | [AFK18898.2](http://smserver.sti.ua.es/millhtml/SM_instruct/servadmn.htm#update_acclinks?AFK18898.2) | 12 | 12 | 194.49 | [66.2](http://smserver.sti.ua.es/millbin/msdigest.cgi?missed_cleavages=2&msparams_dir=msparams_mill/&hide_protein_sequence=2&database=PA_haloferax_mediterranei_genbank&seqdb_dir=D:\SeqDB\&enzyme=Trypsin&access_method=Accession+Number&accession_num=AFK18898.2&coverage_map=0+19+13+8+10+6+27+12+24+25+10+1+21+2+12+1+21+7+8+4+52+16) | 1.71e+007 |
| Aconitate hydratase | [AFK18238.1](http://smserver.sti.ua.es/millhtml/SM_instruct/servadmn.htm#update_acclinks?AFK18238.1) | 13 | 12 | 185.41 | [18.2](http://smserver.sti.ua.es/millbin/msdigest.cgi?missed_cleavages=2&msparams_dir=msparams_mill/&hide_protein_sequence=2&database=PA_haloferax_mediterranei_genbank&seqdb_dir=D:\SeqDB\&enzyme=Trypsin&access_method=Accession+Number&accession_num=AFK18238.1&coverage_map=0+21+44+20+19+149+23+7+17+32+9+235+18+194+22+5+17+94) | 1.82e+007 |
| Proline dehydrogenase | [AFK18055.1](http://smserver.sti.ua.es/millhtml/SM_instruct/servadmn.htm#update_acclinks?AFK18055.1) | 14 | 11 | 185.22 | [53.7](http://smserver.sti.ua.es/millbin/msdigest.cgi?missed_cleavages=2&msparams_dir=msparams_mill/&hide_protein_sequence=2&database=PA_haloferax_mediterranei_genbank&seqdb_dir=D:\SeqDB\&enzyme=Trypsin&access_method=Accession+Number&accession_num=AFK18055.1&coverage_map=0+8+36+48+34+51+53+5+18+12+9+5) | 3.65e+007 |
| Proline dehydrogenase | [AFK18914.1](http://smserver.sti.ua.es/millhtml/SM_instruct/servadmn.htm#update_acclinks?AFK18914.1) | 5 | 5 | 66.54 | [25.4](http://smserver.sti.ua.es/millbin/msdigest.cgi?missed_cleavages=2&msparams_dir=msparams_mill/&hide_protein_sequence=2&database=PA_haloferax_mediterranei_genbank&seqdb_dir=D:\SeqDB\&enzyme=Trypsin&access_method=Accession+Number&accession_num=AFK18914.1&coverage_map=0+44+15+124+47+35+9+5) | 3.23e+006 |
| Catalase (including: peroxidase) | [AFK19564.1](http://smserver.sti.ua.es/millhtml/SM_instruct/servadmn.htm#update_acclinks?AFK19564.1) | 14 | 12 | 185.15 | [22.8](http://smserver.sti.ua.es/millbin/msdigest.cgi?missed_cleavages=2&msparams_dir=msparams_mill/&hide_protein_sequence=2&database=PA_haloferax_mediterranei_genbank&seqdb_dir=D:\SeqDB\&enzyme=Trypsin&access_method=Accession+Number&accession_num=AFK19564.1&coverage_map=0+116+6+43+12+271+10+12+74+42+19+51+8+3+10+2+14+11+10) | 1.91e+007 |
| ABC-type iron(III) transport system,substrate-binding protein | [AFK19499.1](http://smserver.sti.ua.es/millhtml/SM_instruct/servadmn.htm#update_acclinks?AFK19499.1) | 14 | 9 | 181.55 | [48.5](http://smserver.sti.ua.es/millbin/msdigest.cgi?missed_cleavages=2&msparams_dir=msparams_mill/&hide_protein_sequence=2&database=PA_haloferax_mediterranei_genbank&seqdb_dir=D:\SeqDB\&enzyme=Trypsin&access_method=Accession+Number&accession_num=AFK19499.1&coverage_map=0+63+83+7+10+18+48+61+47+50) | 5.63e+007 |
| Thermosome alpha subunit | [AFK18461.1](http://smserver.sti.ua.es/millhtml/SM_instruct/servadmn.htm#update_acclinks?AFK18461.1) | 14 | 11 | 181.45 | [30.7](http://smserver.sti.ua.es/millbin/msdigest.cgi?missed_cleavages=2&msparams_dir=msparams_mill/&hide_protein_sequence=2&database=PA_haloferax_mediterranei_genbank&seqdb_dir=D:\SeqDB\&enzyme=Trypsin&access_method=Accession+Number&accession_num=AFK18461.1&coverage_map=0+164+12+27+40+55+14+39+17+19+32+11+35+5+11+43) | 1.02e+007 |
| A-type ATP synthase subunit D | [AFK18044.1](http://smserver.sti.ua.es/millhtml/SM_instruct/servadmn.htm#update_acclinks?AFK18044.1) | 18 | 11 | 179.68 | [54.8](http://smserver.sti.ua.es/millbin/msdigest.cgi?missed_cleavages=2&msparams_dir=msparams_mill/&hide_protein_sequence=2&database=PA_haloferax_mediterranei_genbank&seqdb_dir=D:\SeqDB\&enzyme=Trypsin&access_method=Accession+Number&accession_num=AFK18044.1&coverage_map=0+32+19+34+24+8+36+1+9+1+23+27+14) | 2.88e+007 |
| Hypothetical protein HFX_1776 | [AFK19481.1](http://smserver.sti.ua.es/millhtml/SM_instruct/servadmn.htm#update_acclinks?AFK19481.1) | 11 | 11 | 176.84 | [34.5](http://smserver.sti.ua.es/millbin/msdigest.cgi?missed_cleavages=2&msparams_dir=msparams_mill/&hide_protein_sequence=2&database=PA_haloferax_mediterranei_genbank&seqdb_dir=D:\SeqDB\&enzyme=Trypsin&access_method=Accession+Number&accession_num=AFK19481.1&coverage_map=0+43+6+14+17+63+18+35+21+50+11+6+9+24+25+30+32+26+13+1+15+25) | 1.56e+007 |
| Electron transfer flavoprotein alpha subunit | [AFK20418.1](http://smserver.sti.ua.es/millhtml/SM_instruct/servadmn.htm#update_acclinks?AFK20418.1) | 11 | 11 | 175.20 | [27.9](http://smserver.sti.ua.es/millbin/msdigest.cgi?missed_cleavages=2&msparams_dir=msparams_mill/&hide_protein_sequence=2&database=PA_haloferax_mediterranei_genbank&seqdb_dir=D:\SeqDB\&enzyme=Trypsin&access_method=Accession+Number&accession_num=AFK20418.1&coverage_map=0+19+17+4+10+135+43+7+15+36+33+77+26+50+12+58+10+42) | 1.22e+007 |
| A-type ATP synthase subunit E | [AFK18038.1](http://smserver.sti.ua.es/millhtml/SM_instruct/servadmn.htm#update_acclinks?AFK18038.1) | 17 | 9 | 174.26 | [68.5](http://smserver.sti.ua.es/millbin/msdigest.cgi?missed_cleavages=2&msparams_dir=msparams_mill/&hide_protein_sequence=2&database=PA_haloferax_mediterranei_genbank&seqdb_dir=D:\SeqDB\&enzyme=Trypsin&access_method=Accession+Number&accession_num=AFK18038.1&coverage_map=0+17+40+27+17+5+58+2+18+10) | 6.89e+007 |
| Phenylacetic acid degradation protein PaaC (plasmid) | [AFK21497.1](http://smserver.sti.ua.es/millhtml/SM_instruct/servadmn.htm#update_acclinks?AFK21497.1) | 11 | 9 | 172.21 | [57.3](http://smserver.sti.ua.es/millbin/msdigest.cgi?missed_cleavages=2&msparams_dir=msparams_mill/&hide_protein_sequence=2&database=PA_haloferax_mediterranei_genbank&seqdb_dir=D:\SeqDB\&enzyme=Trypsin&access_method=Accession+Number&accession_num=AFK21497.1&coverage_map=0+24+40+26+20+9+17+28+33+8+30+2+16+19) | 1.18e+007 |
| Succinate dehydrogenase, subunit B (iron-sulfur protein) | [AFK20496.1](http://smserver.sti.ua.es/millhtml/SM_instruct/servadmn.htm#update_acclinks?AFK20496.1) | 12 | 11 | 171.19 | [37.8](http://smserver.sti.ua.es/millbin/msdigest.cgi?missed_cleavages=2&msparams_dir=msparams_mill/&hide_protein_sequence=2&database=PA_haloferax_mediterranei_genbank&seqdb_dir=D:\SeqDB\&enzyme=Trypsin&access_method=Accession+Number&accession_num=AFK20496.1&coverage_map=0+49+9+15+32+4+16+4+22+79+6+8+26+23) | 2.43e+007 |
| Hypothetical protein HFX_1777 | [AFK19482.1](http://smserver.sti.ua.es/millhtml/SM_instruct/servadmn.htm#update_acclinks?AFK19482.1) | 12 | 9 | 168.49 | [73](http://smserver.sti.ua.es/millbin/msdigest.cgi?missed_cleavages=2&msparams_dir=msparams_mill/&hide_protein_sequence=2&database=PA_haloferax_mediterranei_genbank&seqdb_dir=D:\SeqDB\&enzyme=Trypsin&access_method=Accession+Number&accession_num=AFK19482.1&coverage_map=0+13+11+3+27+22+65) | 1.35e+008 |
| A-type ATP synthase subunit H | [AFK18035.1](http://smserver.sti.ua.es/millhtml/SM_instruct/servadmn.htm#update_acclinks?AFK18035.1) | 11 | 9 | 166.35 | [69](http://smserver.sti.ua.es/millbin/msdigest.cgi?missed_cleavages=2&msparams_dir=msparams_mill/&hide_protein_sequence=2&database=PA_haloferax_mediterranei_genbank&seqdb_dir=D:\SeqDB\&enzyme=Trypsin&access_method=Accession+Number&accession_num=AFK18035.1&coverage_map=0+11+25+7+14+5+7+2+30+9) | 4.47e+007 |
| ABC-type dipeptide/oligopeptide/nickel transport system, substrate binding protein | [AFK20131.1](http://smserver.sti.ua.es/millhtml/SM_instruct/servadmn.htm#update_acclinks?AFK20131.1) | 10 | 9 | 163.61 | [31.9](http://smserver.sti.ua.es/millbin/msdigest.cgi?missed_cleavages=2&msparams_dir=msparams_mill/&hide_protein_sequence=2&database=PA_haloferax_mediterranei_genbank&seqdb_dir=D:\SeqDB\&enzyme=Trypsin&access_method=Accession+Number&accession_num=AFK20131.1&coverage_map=0+81+34+133+9+8+38+93+23+2+16+34+53+17) | 1.96e+007 |
| Glutamate dehydrogenase (NAD(P)+) | [AFK19225.1](http://smserver.sti.ua.es/millhtml/SM_instruct/servadmn.htm#update_acclinks?AFK19225.1) | 12 | 9 | 161.60 | [39.3](http://smserver.sti.ua.es/millbin/msdigest.cgi?missed_cleavages=2&msparams_dir=msparams_mill/&hide_protein_sequence=2&database=PA_haloferax_mediterranei_genbank&seqdb_dir=D:\SeqDB\&enzyme=Trypsin&access_method=Accession+Number&accession_num=AFK19225.1&coverage_map=0+37+19+84+12+6+18+23+57+75+26+16+32+12) | 1.34e+007 |
| Branched-chain-amino-acid aminotransferase | [AFK18053.1](http://smserver.sti.ua.es/millhtml/SM_instruct/servadmn.htm#update_acclinks?AFK18053.1) | 12 | 10 | 159.45 | [49.6](http://smserver.sti.ua.es/millbin/msdigest.cgi?missed_cleavages=2&msparams_dir=msparams_mill/&hide_protein_sequence=2&database=PA_haloferax_mediterranei_genbank&seqdb_dir=D:\SeqDB\&enzyme=Trypsin&access_method=Accession+Number&accession_num=AFK18053.1&coverage_map=0+43+12+7+28+88+27+3+60+1+28+15) | 1.29e+007 |
| Glycerol kinase | [AFK19308.1](http://smserver.sti.ua.es/millhtml/SM_instruct/servadmn.htm#update_acclinks?AFK19308.1) | 11 | 11 | 156.84 | [26.3](http://smserver.sti.ua.es/millbin/msdigest.cgi?missed_cleavages=2&msparams_dir=msparams_mill/&hide_protein_sequence=2&database=PA_haloferax_mediterranei_genbank&seqdb_dir=D:\SeqDB\&enzyme=Trypsin&access_method=Accession+Number&accession_num=AFK19308.1&coverage_map=0+84+23+1+18+57+37+152+8+9+29+54+20+20) | 6.56e+006 |
| Flavin-dependent dehydrogenase | [AFK20419.1](http://smserver.sti.ua.es/millhtml/SM_instruct/servadmn.htm#update_acclinks?AFK20419.1) | 10 | 9 | 150.52 | [26.3](http://smserver.sti.ua.es/millbin/msdigest.cgi?missed_cleavages=2&msparams_dir=msparams_mill/&hide_protein_sequence=2&database=PA_haloferax_mediterranei_genbank&seqdb_dir=D:\SeqDB\&enzyme=Trypsin&access_method=Accession+Number&accession_num=AFK20419.1&coverage_map=0+81+16+30+17+4+29+18+13+22+9+67+19+48+28+44+15+94) | 1.01e+007 |
| Dihydrolipoamide S-acyltransferase (pyruvate dehydrogenase E2 component) | [AFK20618.1](http://smserver.sti.ua.es/millhtml/SM_instruct/servadmn.htm#update_acclinks?AFK20618.1) | 11 | 10 | 149.87 | [24.2](http://smserver.sti.ua.es/millbin/msdigest.cgi?missed_cleavages=2&msparams_dir=msparams_mill/&hide_protein_sequence=2&database=PA_haloferax_mediterranei_genbank&seqdb_dir=D:\SeqDB\&enzyme=Trypsin&access_method=Accession+Number&accession_num=AFK20618.1&coverage_map=0+112+8+4+17+7+18+12+14+99+21+22+7+65+7+39+9+1+11+13+9+5) | 1.38e+007 |
| Immunogenic protein | [AFK20638.1](http://smserver.sti.ua.es/millhtml/SM_instruct/servadmn.htm#update_acclinks?AFK20638.1) | 13 | 8 | 149.49 | [48.8](http://smserver.sti.ua.es/millbin/msdigest.cgi?missed_cleavages=2&msparams_dir=msparams_mill/&hide_protein_sequence=2&database=PA_haloferax_mediterranei_genbank&seqdb_dir=D:\SeqDB\&enzyme=Trypsin&access_method=Accession+Number&accession_num=AFK20638.1&coverage_map=0+57+111+42+37+69+16+4) | 1.25e+008 |
| Acyl-CoA synthetase | [AFK18591.1](http://smserver.sti.ua.es/millhtml/SM_instruct/servadmn.htm#update_acclinks?AFK18591.1) | 10 | 10 | 149.37 | [19](http://smserver.sti.ua.es/millbin/msdigest.cgi?missed_cleavages=2&msparams_dir=msparams_mill/&hide_protein_sequence=2&database=PA_haloferax_mediterranei_genbank&seqdb_dir=D:\SeqDB\&enzyme=Trypsin&access_method=Accession+Number&accession_num=AFK18591.1&coverage_map=0+79+10+22+9+39+20+7+10+10+20+122+7+33+12+119+23+105+15) | 6.95e+006 |
| Acyl-CoA synthetase (plasmid) | [AFK20963.1](http://smserver.sti.ua.es/millhtml/SM_instruct/servadmn.htm#update_acclinks?AFK20963.1) | 3 | 3 | 44.68 | [4.9](http://smserver.sti.ua.es/millbin/msdigest.cgi?missed_cleavages=2&msparams_dir=msparams_mill/&hide_protein_sequence=2&database=PA_haloferax_mediterranei_genbank&seqdb_dir=D:\SeqDB\&enzyme=Trypsin&access_method=Accession+Number&accession_num=AFK20963.1&coverage_map=0+111+9+265+12+124+11+119) | 3.28e+006 |
| Acyl-CoA synthetase (plasmid) | [AFK20965.1](http://smserver.sti.ua.es/millhtml/SM_instruct/servadmn.htm#update_acclinks?AFK20965.1) | 5 | 5 | 73.25 | [8.1](http://smserver.sti.ua.es/millbin/msdigest.cgi?missed_cleavages=2&msparams_dir=msparams_mill/&hide_protein_sequence=2&database=PA_haloferax_mediterranei_genbank&seqdb_dir=D:\SeqDB\&enzyme=Trypsin&access_method=Accession+Number&accession_num=AFK20965.1&coverage_map=0+111+9+66+10+192+12+119+23+122) | 3.77e+006 |
| Phosphohexomutase (phosphoglucomutase, phosphomannomutase) | [AFK17941.1](http://smserver.sti.ua.es/millhtml/SM_instruct/servadmn.htm#update_acclinks?AFK17941.1) | 10 | 10 | 145.64 | [35.2](http://smserver.sti.ua.es/millbin/msdigest.cgi?missed_cleavages=2&msparams_dir=msparams_mill/&hide_protein_sequence=2&database=PA_haloferax_mediterranei_genbank&seqdb_dir=D:\SeqDB\&enzyme=Trypsin&access_method=Accession+Number&accession_num=AFK17941.1&coverage_map=0+12+10+126+50+45+8+26+10+24+17+30+12+12+16+12+19+2+19+7) | 4.71e+006 |
| 2-methylcitrate dehydratase (plasmid) | [AFK21002.1](http://smserver.sti.ua.es/millhtml/SM_instruct/servadmn.htm#update_acclinks?AFK21002.1) | 9 | 9 | 142.00 | [34.7](http://smserver.sti.ua.es/millbin/msdigest.cgi?missed_cleavages=2&msparams_dir=msparams_mill/&hide_protein_sequence=2&database=PA_haloferax_mediterranei_genbank&seqdb_dir=D:\SeqDB\&enzyme=Trypsin&access_method=Accession+Number&accession_num=AFK21002.1&coverage_map=0+12+16+2+21+8+26+61+42+128+18+35+24+32+9+15) | 7.44e+006 |
| Molecular chaperone DnaK | [AFK19355.1](http://smserver.sti.ua.es/millhtml/SM_instruct/servadmn.htm#update_acclinks?AFK19355.1) | 9 | 8 | 141.80 | [19.8](http://smserver.sti.ua.es/millbin/msdigest.cgi?missed_cleavages=2&msparams_dir=msparams_mill/&hide_protein_sequence=2&database=PA_haloferax_mediterranei_genbank&seqdb_dir=D:\SeqDB\&enzyme=Trypsin&access_method=Accession+Number&accession_num=AFK19355.1&coverage_map=0+36+14+7+12+35+24+6+12+143+30+79+15+106+17+89) | 1.00e+007 |
| Aldehyde dehydrogenase (NAD+) | [AFK18912.1](http://smserver.sti.ua.es/millhtml/SM_instruct/servadmn.htm#update_acclinks?AFK18912.1) | 10 | 10 | 141.17 | [26.6](http://smserver.sti.ua.es/millbin/msdigest.cgi?missed_cleavages=2&msparams_dir=msparams_mill/&hide_protein_sequence=2&database=PA_haloferax_mediterranei_genbank&seqdb_dir=D:\SeqDB\&enzyme=Trypsin&access_method=Accession+Number&accession_num=AFK18912.1&coverage_map=0+39+21+7+12+2+10+10+18+100+19+24+16+25+6+76+15+72+18+17) | 6.16e+006 |
| Phosphoserine phosphatase | [AFK20621.1](http://smserver.sti.ua.es/millhtml/SM_instruct/servadmn.htm#update_acclinks?AFK20621.1) | 9 | 8 | 141.02 | [66.6](http://smserver.sti.ua.es/millbin/msdigest.cgi?missed_cleavages=2&msparams_dir=msparams_mill/&hide_protein_sequence=2&database=PA_haloferax_mediterranei_genbank&seqdb_dir=D:\SeqDB\&enzyme=Trypsin&access_method=Accession+Number&accession_num=AFK20621.1&coverage_map=0+37+13+4+29+25+16+3+73+3+13) | 5.40e+006 |
| Molybdate transport protein | [AFK19675.1](http://smserver.sti.ua.es/millhtml/SM_instruct/servadmn.htm#update_acclinks?AFK19675.1) | 9 | 8 | 137.84 | [30.4](http://smserver.sti.ua.es/millbin/msdigest.cgi?missed_cleavages=2&msparams_dir=msparams_mill/&hide_protein_sequence=2&database=PA_haloferax_mediterranei_genbank&seqdb_dir=D:\SeqDB\&enzyme=Trypsin&access_method=Accession+Number&accession_num=AFK19675.1&coverage_map=0+87+42+24+18+9+15+7+20+28+12+89) | 8.38e+006 |
| Stress response protein | [AFK20336.2](http://smserver.sti.ua.es/millhtml/SM_instruct/servadmn.htm#update_acclinks?AFK20336.2) | 12 | 7 | 137.45 | [37](http://smserver.sti.ua.es/millbin/msdigest.cgi?missed_cleavages=2&msparams_dir=msparams_mill/&hide_protein_sequence=2&database=PA_haloferax_mediterranei_genbank&seqdb_dir=D:\SeqDB\&enzyme=Trypsin&access_method=Accession+Number&accession_num=AFK20336.2&coverage_map=0+21+14+62+24+30+17+4+36+6+13+54) | 1.74e+007 |
| Potassium transport protein kefC | [AFK19701.1](http://smserver.sti.ua.es/millhtml/SM_instruct/servadmn.htm#update_acclinks?AFK19701.1) | 11 | 9 | 133.58 | [16](http://smserver.sti.ua.es/millbin/msdigest.cgi?missed_cleavages=2&msparams_dir=msparams_mill/&hide_protein_sequence=2&database=PA_haloferax_mediterranei_genbank&seqdb_dir=D:\SeqDB\&enzyme=Trypsin&access_method=Accession+Number&accession_num=AFK19701.1&coverage_map=0+214+14+109+9+73+8+8+22+48+25+30+17+38+7+13) | 7.24e+006 |
| AAA-type ATPase (transitional ATPase-like protein) | [AFK20074.1](http://smserver.sti.ua.es/millhtml/SM_instruct/servadmn.htm#update_acclinks?AFK20074.1) | 11 | 10 | 132.85 | [15.9](http://smserver.sti.ua.es/millbin/msdigest.cgi?missed_cleavages=2&msparams_dir=msparams_mill/&hide_protein_sequence=2&database=PA_haloferax_mediterranei_genbank&seqdb_dir=D:\SeqDB\&enzyme=Trypsin&access_method=Accession+Number&accession_num=AFK20074.1&coverage_map=0+22+10+276+15+27+21+79+8+56+14+24+19+53+12+39+19+48) | 3.33e+006 |
| Phosphopyruvate hydratase | [AFK20460.1](http://smserver.sti.ua.es/millhtml/SM_instruct/servadmn.htm#update_acclinks?AFK20460.1) | 10 | 8 | 132.49 | [30.8](http://smserver.sti.ua.es/millbin/msdigest.cgi?missed_cleavages=2&msparams_dir=msparams_mill/&hide_protein_sequence=2&database=PA_haloferax_mediterranei_genbank&seqdb_dir=D:\SeqDB\&enzyme=Trypsin&access_method=Accession+Number&accession_num=AFK20460.1&coverage_map=0+16+18+33+18+25+19+50+8+41+23+43+15+45+22+23) | 1.18e+007 |
| Hypothetical protein HFX_1529 | [AFK19236.1](http://smserver.sti.ua.es/millhtml/SM_instruct/servadmn.htm#update_acclinks?AFK19236.1) | 10 | 8 | 131.14 | [29.9](http://smserver.sti.ua.es/millbin/msdigest.cgi?missed_cleavages=2&msparams_dir=msparams_mill/&hide_protein_sequence=2&database=PA_haloferax_mediterranei_genbank&seqdb_dir=D:\SeqDB\&enzyme=Trypsin&access_method=Accession+Number&accession_num=AFK19236.1&coverage_map=0+117+16+2+13+34+12+9+25+75+17+37+39+11) | 7.64e+006 |
| ABC-type transport system ATP-binding protein | [AFK18720.1](http://smserver.sti.ua.es/millhtml/SM_instruct/servadmn.htm#update_acclinks?AFK18720.1) | 7 | 7 | 127.73 | [49.5](http://smserver.sti.ua.es/millbin/msdigest.cgi?missed_cleavages=2&msparams_dir=msparams_mill/&hide_protein_sequence=2&database=PA_haloferax_mediterranei_genbank&seqdb_dir=D:\SeqDB\&enzyme=Trypsin&access_method=Accession+Number&accession_num=AFK18720.1&coverage_map=0+48+25+2+13+4+42+30+59+21+16+53) | 7.35e+006 |
| Hypothetical protein HFX_0491 | [AFK18222.1](http://smserver.sti.ua.es/millhtml/SM_instruct/servadmn.htm#update_acclinks?AFK18222.1) | 10 | 7 | 126.94 | [53](http://smserver.sti.ua.es/millbin/msdigest.cgi?missed_cleavages=2&msparams_dir=msparams_mill/&hide_protein_sequence=2&database=PA_haloferax_mediterranei_genbank&seqdb_dir=D:\SeqDB\&enzyme=Trypsin&access_method=Accession+Number&accession_num=AFK18222.1&coverage_map=0+51+15+7+12+10+60+9) | 2.54e+007 |
| Hypothetical protein HFX_1590 | [AFK19296.1](http://smserver.sti.ua.es/millhtml/SM_instruct/servadmn.htm#update_acclinks?AFK19296.1) | 9 | 8 | 126.77 | [27.7](http://smserver.sti.ua.es/millbin/msdigest.cgi?missed_cleavages=2&msparams_dir=msparams_mill/&hide_protein_sequence=2&database=PA_haloferax_mediterranei_genbank&seqdb_dir=D:\SeqDB\&enzyme=Trypsin&access_method=Accession+Number&accession_num=AFK19296.1&coverage_map=0+88+38+44+21+4+21+71+8+1+26+42+12+26+17+96) | 9.75e+006 |
| RecJ-like exonuclease | [AFK20553.1](http://smserver.sti.ua.es/millhtml/SM_instruct/servadmn.htm#update_acclinks?AFK20553.1) | 9 | 9 | 124.89 | [19.5](http://smserver.sti.ua.es/millbin/msdigest.cgi?missed_cleavages=2&msparams_dir=msparams_mill/&hide_protein_sequence=2&database=PA_haloferax_mediterranei_genbank&seqdb_dir=D:\SeqDB\&enzyme=Trypsin&access_method=Accession+Number&accession_num=AFK20553.1&coverage_map=0+241+9+28+12+33+38+139+20+53+39+55+8+32+15) | 3.45e+006 |
| Putative phosphonate ABC transporter, periplasmic phosphonate-binding protein | [AFK19847.1](http://smserver.sti.ua.es/millhtml/SM_instruct/servadmn.htm#update_acclinks?AFK19847.1) | 8 | 7 | 123.69 | [26.1](http://smserver.sti.ua.es/millbin/msdigest.cgi?missed_cleavages=2&msparams_dir=msparams_mill/&hide_protein_sequence=2&database=PA_haloferax_mediterranei_genbank&seqdb_dir=D:\SeqDB\&enzyme=Trypsin&access_method=Accession+Number&accession_num=AFK19847.1&coverage_map=0+108+24+16+18+39+19+65+11+2+28+53) | 2.01e+007 |
| Nitrite reductase copper containing protein | [AFK19882.1](http://smserver.sti.ua.es/millhtml/SM_instruct/servadmn.htm#update_acclinks?AFK19882.1) | 12 | 8 | 123.51 | [25.1](http://smserver.sti.ua.es/millbin/msdigest.cgi?missed_cleavages=2&msparams_dir=msparams_mill/&hide_protein_sequence=2&database=PA_haloferax_mediterranei_genbank&seqdb_dir=D:\SeqDB\&enzyme=Trypsin&access_method=Accession+Number&accession_num=AFK19882.1&coverage_map=0+150+14+29+11+4+14+8+12+2+10+4+17+21+6+10+13+61) | 3.91e+007 |
| Hypothetical protein HFX_6060 (plasmid) | [AFK21187.1](http://smserver.sti.ua.es/millhtml/SM_instruct/servadmn.htm#update_acclinks?AFK21187.1) | 10 | 7 | 123.04 | [25.9](http://smserver.sti.ua.es/millbin/msdigest.cgi?missed_cleavages=2&msparams_dir=msparams_mill/&hide_protein_sequence=2&database=PA_haloferax_mediterranei_genbank&seqdb_dir=D:\SeqDB\&enzyme=Trypsin&access_method=Accession+Number&accession_num=AFK21187.1&coverage_map=0+67+5+79+24+44+20+16+23+12+16+33) | 9.06e+006 |
| Arylsulfatase | [AFK18199.1](http://smserver.sti.ua.es/millhtml/SM_instruct/servadmn.htm#update_acclinks?AFK18199.1) | 8 | 8 | 122.75 | [29](http://smserver.sti.ua.es/millbin/msdigest.cgi?missed_cleavages=2&msparams_dir=msparams_mill/&hide_protein_sequence=2&database=PA_haloferax_mediterranei_genbank&seqdb_dir=D:\SeqDB\&enzyme=Trypsin&access_method=Accession+Number&accession_num=AFK18199.1&coverage_map=0+84+27+11+7+104+25+8+27+37+33+44+14+37) | 6.84e+006 |
| Geranylgeranyl hydrogenase-like protein / electron-transferring-flavoprotein dehydrogenase | [AFK19181.1](http://smserver.sti.ua.es/millhtml/SM_instruct/servadmn.htm#update_acclinks?AFK19181.1) | 8 | 8 | 122.66 | [27.1](http://smserver.sti.ua.es/millbin/msdigest.cgi?missed_cleavages=2&msparams_dir=msparams_mill/&hide_protein_sequence=2&database=PA_haloferax_mediterranei_genbank&seqdb_dir=D:\SeqDB\&enzyme=Trypsin&access_method=Accession+Number&accession_num=AFK19181.1&coverage_map=0+26+10+16+11+16+23+89+32+42+8+140+26+2+14+1) | 1.25e+007 |
| Molybdopterin oxidoreductase | [AFK19899.1](http://smserver.sti.ua.es/millhtml/SM_instruct/servadmn.htm#update_acclinks?AFK19899.1) | 10 | 7 | 122.34 | [30.7](http://smserver.sti.ua.es/millbin/msdigest.cgi?missed_cleavages=2&msparams_dir=msparams_mill/&hide_protein_sequence=2&database=PA_haloferax_mediterranei_genbank&seqdb_dir=D:\SeqDB\&enzyme=Trypsin&access_method=Accession+Number&accession_num=AFK19899.1&coverage_map=0+12+12+57+21+5+19+27+32+88) | 2.38e+007 |
| S-adenosylmethionine-dependent methyltransferase-like protein | [AFK18456.1](http://smserver.sti.ua.es/millhtml/SM_instruct/servadmn.htm#update_acclinks?AFK18456.1) | 9 | 8 | 121.45 | [38](http://smserver.sti.ua.es/millbin/msdigest.cgi?missed_cleavages=2&msparams_dir=msparams_mill/&hide_protein_sequence=2&database=PA_haloferax_mediterranei_genbank&seqdb_dir=D:\SeqDB\&enzyme=Trypsin&access_method=Accession+Number&accession_num=AFK18456.1&coverage_map=0+33+24+47+25+10+11+3+12+9+24+54) | 1.39e+007 |
| Enoyl-CoA hydratase (plasmid) | [AFK21050.1](http://smserver.sti.ua.es/millhtml/SM_instruct/servadmn.htm#update_acclinks?AFK21050.1) | 11 | 7 | 119.95 | [48.4](http://smserver.sti.ua.es/millbin/msdigest.cgi?missed_cleavages=2&msparams_dir=msparams_mill/&hide_protein_sequence=2&database=PA_haloferax_mediterranei_genbank&seqdb_dir=D:\SeqDB\&enzyme=Trypsin&access_method=Accession+Number&accession_num=AFK21050.1&coverage_map=0+42+37+37+12+4+57+30) | 4.85e+007 |
| Pyruvate--ferredoxin oxidoreductase, beta subunit | [AFK19080.1](http://smserver.sti.ua.es/millhtml/SM_instruct/servadmn.htm#update_acclinks?AFK19080.1) | 7 | 7 | 119.89 | [37.8](http://smserver.sti.ua.es/millbin/msdigest.cgi?missed_cleavages=2&msparams_dir=msparams_mill/&hide_protein_sequence=2&database=PA_haloferax_mediterranei_genbank&seqdb_dir=D:\SeqDB\&enzyme=Trypsin&access_method=Accession+Number&accession_num=AFK19080.1&coverage_map=0+66+16+9+49+101+23+16+30+2) | 5.55e+006 |
| Phytoene dehydrogenase (phytoene desaturase) | [AFK18509.1](http://smserver.sti.ua.es/millhtml/SM_instruct/servadmn.htm#update_acclinks?AFK18509.1) | 9 | 8 | 119.81 | [26.7](http://smserver.sti.ua.es/millbin/msdigest.cgi?missed_cleavages=2&msparams_dir=msparams_mill/&hide_protein_sequence=2&database=PA_haloferax_mediterranei_genbank&seqdb_dir=D:\SeqDB\&enzyme=Trypsin&access_method=Accession+Number&accession_num=AFK18509.1&coverage_map=0+20+14+20+13+1+23+1+18+22+14+48+20+47+12+153) | 6.29e+006 |
| Poly(3-hydroxyalkanoate) granule-associated 12 kDa protein (plasmid) | [AFK21051.1](http://smserver.sti.ua.es/millhtml/SM_instruct/servadmn.htm#update_acclinks?AFK21051.1) | 8 | 6 | 118.95 | [77.2](http://smserver.sti.ua.es/millbin/msdigest.cgi?missed_cleavages=2&msparams_dir=msparams_mill/&hide_protein_sequence=2&database=PA_haloferax_mediterranei_genbank&seqdb_dir=D:\SeqDB\&enzyme=Trypsin&access_method=Accession+Number&accession_num=AFK21051.1&coverage_map=0+9+58+8+27+8) | 4.68e+007 |
| CBS domain-containing protein | [AFK17996.1](http://smserver.sti.ua.es/millhtml/SM_instruct/servadmn.htm#update_acclinks?AFK17996.1) | 8 | 7 | 117.73 | [21.2](http://smserver.sti.ua.es/millbin/msdigest.cgi?missed_cleavages=2&msparams_dir=msparams_mill/&hide_protein_sequence=2&database=PA_haloferax_mediterranei_genbank&seqdb_dir=D:\SeqDB\&enzyme=Trypsin&access_method=Accession+Number&accession_num=AFK17996.1&coverage_map=0+140+13+28+27+56+18+4+25+98+16+40) | 9.08e+006 |
| Glutamine synthetase | [AFK17986.2](http://smserver.sti.ua.es/millhtml/SM_instruct/servadmn.htm#update_acclinks?AFK17986.2) | 7 | 7 | 115.36 | [25.2](http://smserver.sti.ua.es/millbin/msdigest.cgi?missed_cleavages=2&msparams_dir=msparams_mill/&hide_protein_sequence=2&database=PA_haloferax_mediterranei_genbank&seqdb_dir=D:\SeqDB\&enzyme=Trypsin&access_method=Accession+Number&accession_num=AFK17986.2&coverage_map=0+54+18+7+14+9+19+188+16+47+9+21+26+10+13+5) | 2.90e+006 |
| Glycosyl transferase | [AFK19287.1](http://smserver.sti.ua.es/millhtml/SM_instruct/servadmn.htm#update_acclinks?AFK19287.1) | 9 | 8 | 114.33 | [30.7](http://smserver.sti.ua.es/millbin/msdigest.cgi?missed_cleavages=2&msparams_dir=msparams_mill/&hide_protein_sequence=2&database=PA_haloferax_mediterranei_genbank&seqdb_dir=D:\SeqDB\&enzyme=Trypsin&access_method=Accession+Number&accession_num=AFK19287.1&coverage_map=0+44+16+7+29+6+13+34+60+175) | 2.54e+006 |
| IMP dehydrogenase | [AFK18995.1](http://smserver.sti.ua.es/millhtml/SM_instruct/servadmn.htm#update_acclinks?AFK18995.1) | 8 | 7 | 113.34 | [17.4](http://smserver.sti.ua.es/millbin/msdigest.cgi?missed_cleavages=2&msparams_dir=msparams_mill/&hide_protein_sequence=2&database=PA_haloferax_mediterranei_genbank&seqdb_dir=D:\SeqDB\&enzyme=Trypsin&access_method=Accession+Number&accession_num=AFK18995.1&coverage_map=0+75+9+82+12+5+10+12+13+13+14+32+29+192) | 5.52e+006 |
| Nitrite reductase (NO-forming) | [AFK19872.1](http://smserver.sti.ua.es/millhtml/SM_instruct/servadmn.htm#update_acclinks?AFK19872.1) | 10 | 6 | 112.23 | [20.8](http://smserver.sti.ua.es/millbin/msdigest.cgi?missed_cleavages=2&msparams_dir=msparams_mill/&hide_protein_sequence=2&database=PA_haloferax_mediterranei_genbank&seqdb_dir=D:\SeqDB\&enzyme=Trypsin&access_method=Accession+Number&accession_num=AFK19872.1&coverage_map=0+50+18+161+34+62+8+11+15) | 1.53e+008 |
| Pyruvate--ferredoxin oxidoreductase, alpha subunit | [AFK19081.1](http://smserver.sti.ua.es/millhtml/SM_instruct/servadmn.htm#update_acclinks?AFK19081.1) | 7 | 6 | 111.45 | [16.1](http://smserver.sti.ua.es/millbin/msdigest.cgi?missed_cleavages=2&msparams_dir=msparams_mill/&hide_protein_sequence=2&database=PA_haloferax_mediterranei_genbank&seqdb_dir=D:\SeqDB\&enzyme=Trypsin&access_method=Accession+Number&accession_num=AFK19081.1&coverage_map=0+31+9+25+16+73+20+151+45+167+12+82) | 7.90e+006 |
| FAD dependent oxidoreductase | [AFK19621.1](http://smserver.sti.ua.es/millhtml/SM_instruct/servadmn.htm#update_acclinks?AFK19621.1) | 7 | 7 | 110.91 | [22.7](http://smserver.sti.ua.es/millbin/msdigest.cgi?missed_cleavages=2&msparams_dir=msparams_mill/&hide_protein_sequence=2&database=PA_haloferax_mediterranei_genbank&seqdb_dir=D:\SeqDB\&enzyme=Trypsin&access_method=Accession+Number&accession_num=AFK19621.1&coverage_map=0+47+10+20+12+68+52+143+21+25+9+50) | 4.49e+006 |
| ABC-type glutamine/glutamate/polar amino acids transport system, substrate-binding protein | [AFK20126.1](http://smserver.sti.ua.es/millhtml/SM_instruct/servadmn.htm#update_acclinks?AFK20126.1) | 7 | 6 | 109.41 | [38.8](http://smserver.sti.ua.es/millbin/msdigest.cgi?missed_cleavages=2&msparams_dir=msparams_mill/&hide_protein_sequence=2&database=PA_haloferax_mediterranei_genbank&seqdb_dir=D:\SeqDB\&enzyme=Trypsin&access_method=Accession+Number&accession_num=AFK20126.1&coverage_map=0+71+33+19+35+38+18+8+10+15) | 6.38e+006 |
| Halocyanin precursor-like protein | [AFK19879.1](http://smserver.sti.ua.es/millhtml/SM_instruct/servadmn.htm#update_acclinks?AFK19879.1) | 7 | 6 | 107.30 | [35.7](http://smserver.sti.ua.es/millbin/msdigest.cgi?missed_cleavages=2&msparams_dir=msparams_mill/&hide_protein_sequence=2&database=PA_haloferax_mediterranei_genbank&seqdb_dir=D:\SeqDB\&enzyme=Trypsin&access_method=Accession+Number&accession_num=AFK19879.1&coverage_map=0+52+87+74+22+28+22+81) | 3.41e+007 |
| Sulfatase arylsulfatase A-like protein | [AFK18425.2](http://smserver.sti.ua.es/millhtml/SM_instruct/servadmn.htm#update_acclinks?AFK18425.2) | 8 | 7 | 106.95 | [18.9](http://smserver.sti.ua.es/millbin/msdigest.cgi?missed_cleavages=2&msparams_dir=msparams_mill/&hide_protein_sequence=2&database=PA_haloferax_mediterranei_genbank&seqdb_dir=D:\SeqDB\&enzyme=Trypsin&access_method=Accession+Number&accession_num=AFK18425.2&coverage_map=0+82+13+35+12+85+8+72+8+19+12+14+21+4+11+53) | 1.74e+007 |
| Sulfatase arylsulfatase A-like protein | [AFK19826.1](http://smserver.sti.ua.es/millhtml/SM_instruct/servadmn.htm#update_acclinks?AFK19826.1) | 2 | 2 | 33.86 | [4.2](http://smserver.sti.ua.es/millbin/msdigest.cgi?missed_cleavages=2&msparams_dir=msparams_mill/&hide_protein_sequence=2&database=PA_haloferax_mediterranei_genbank&seqdb_dir=D:\SeqDB\&enzyme=Trypsin&access_method=Accession+Number&accession_num=AFK19826.1&coverage_map=0+308+8+70+11+51) | 1.03e+007 |
| Hypothetical protein HFX_2807 | [AFK20480.1](http://smserver.sti.ua.es/millhtml/SM_instruct/servadmn.htm#update_acclinks?AFK20480.1) | 8 | 6 | 106.04 | [71.6](http://smserver.sti.ua.es/millbin/msdigest.cgi?missed_cleavages=2&msparams_dir=msparams_mill/&hide_protein_sequence=2&database=PA_haloferax_mediterranei_genbank&seqdb_dir=D:\SeqDB\&enzyme=Trypsin&access_method=Accession+Number&accession_num=AFK20480.1&coverage_map=0+18+33+5+24+7+19) | 2.92e+007 |
| Gluconate dehydratase | [AFK19260.1](http://smserver.sti.ua.es/millhtml/SM_instruct/servadmn.htm#update_acclinks?AFK19260.1) | 9 | 7 | 104.63 | [26.9](http://smserver.sti.ua.es/millbin/msdigest.cgi?missed_cleavages=2&msparams_dir=msparams_mill/&hide_protein_sequence=2&database=PA_haloferax_mediterranei_genbank&seqdb_dir=D:\SeqDB\&enzyme=Trypsin&access_method=Accession+Number&accession_num=AFK19260.1&coverage_map=0+18+12+27+25+23+28+41+22+5+8+84+16+103) | 1.46e+007 |
| Aldehyde reductase | [AFK18579.1](http://smserver.sti.ua.es/millhtml/SM_instruct/servadmn.htm#update_acclinks?AFK18579.1) | 6 | 6 | 103.73 | [35.5](http://smserver.sti.ua.es/millbin/msdigest.cgi?missed_cleavages=2&msparams_dir=msparams_mill/&hide_protein_sequence=2&database=PA_haloferax_mediterranei_genbank&seqdb_dir=D:\SeqDB\&enzyme=Trypsin&access_method=Accession+Number&accession_num=AFK18579.1&coverage_map=0+79+16+43+12+27+47+21+23+8) | 5.50e+006 |
| Putative hydrolase or acyltransferase of alpha/beta superfamily | [AFK18525.1](http://smserver.sti.ua.es/millhtml/SM_instruct/servadmn.htm#update_acclinks?AFK18525.1) | 7 | 7 | 102.61 | [40.2](http://smserver.sti.ua.es/millbin/msdigest.cgi?missed_cleavages=2&msparams_dir=msparams_mill/&hide_protein_sequence=2&database=PA_haloferax_mediterranei_genbank&seqdb_dir=D:\SeqDB\&enzyme=Trypsin&access_method=Accession+Number&accession_num=AFK18525.1&coverage_map=0+5+21+1+16+2+33+74+9+20+45+82) | 1.19e+007 |
| Hypothetical protein HFX_1575 | [AFK19282.2](http://smserver.sti.ua.es/millhtml/SM_instruct/servadmn.htm#update_acclinks?AFK19282.2) | 8 | 6 | 102.03 | [39.9](http://smserver.sti.ua.es/millbin/msdigest.cgi?missed_cleavages=2&msparams_dir=msparams_mill/&hide_protein_sequence=2&database=PA_haloferax_mediterranei_genbank&seqdb_dir=D:\SeqDB\&enzyme=Trypsin&access_method=Accession+Number&accession_num=AFK19282.2&coverage_map=0+77+46+4+36+14+9+42) | 1.34e+007 |
| Hypothetical protein HFX_1766 | [AFK19471.1](http://smserver.sti.ua.es/millhtml/SM_instruct/servadmn.htm#update_acclinks?AFK19471.1) | 7 | 7 | 101.15 | [47.2](http://smserver.sti.ua.es/millbin/msdigest.cgi?missed_cleavages=2&msparams_dir=msparams_mill/&hide_protein_sequence=2&database=PA_haloferax_mediterranei_genbank&seqdb_dir=D:\SeqDB\&enzyme=Trypsin&access_method=Accession+Number&accession_num=AFK19471.1&coverage_map=0+62+9+13+28+2+23+27+14+2+28+8) | 2.31e+007 |
| Cytochrome b subunit of nitric oxide reductase | [AFK19877.1](http://smserver.sti.ua.es/millhtml/SM_instruct/servadmn.htm#update_acclinks?AFK19877.1) | 8 | 5 | 99.58 | [14.3](http://smserver.sti.ua.es/millbin/msdigest.cgi?missed_cleavages=2&msparams_dir=msparams_mill/&hide_protein_sequence=2&database=PA_haloferax_mediterranei_genbank&seqdb_dir=D:\SeqDB\&enzyme=Trypsin&access_method=Accession+Number&accession_num=AFK19877.1&coverage_map=0+104+22+149+50+372+37+27) | 8.25e+006 |
| AAA-type ATPase (transitional ATPase-like protein) | [AFK20391.1](http://smserver.sti.ua.es/millhtml/SM_instruct/servadmn.htm#update_acclinks?AFK20391.1) | 7 | 7 | 98.29 | [11.1](http://smserver.sti.ua.es/millbin/msdigest.cgi?missed_cleavages=2&msparams_dir=msparams_mill/&hide_protein_sequence=2&database=PA_haloferax_mediterranei_genbank&seqdb_dir=D:\SeqDB\&enzyme=Trypsin&access_method=Accession+Number&accession_num=AFK20391.1&coverage_map=0+15+7+71+10+206+26+304+8+16+14+8+19+50) | 3.34e+006 |
| Hypothetical protein HFX_2555 | [AFK20236.1](http://smserver.sti.ua.es/millhtml/SM_instruct/servadmn.htm#update_acclinks?AFK20236.1) | 7 | 7 | 98.17 | [22.3](http://smserver.sti.ua.es/millbin/msdigest.cgi?missed_cleavages=2&msparams_dir=msparams_mill/&hide_protein_sequence=2&database=PA_haloferax_mediterranei_genbank&seqdb_dir=D:\SeqDB\&enzyme=Trypsin&access_method=Accession+Number&accession_num=AFK20236.1&coverage_map=0+83+22+14+12+86+9+27+32+51) | 8.84e+006 |
| UpsA domain-containing protein | [AFK19975.2](http://smserver.sti.ua.es/millhtml/SM_instruct/servadmn.htm#update_acclinks?AFK19975.2) | 6 | 6 | 97.58 | [47.1](http://smserver.sti.ua.es/millbin/msdigest.cgi?missed_cleavages=2&msparams_dir=msparams_mill/&hide_protein_sequence=2&database=PA_haloferax_mediterranei_genbank&seqdb_dir=D:\SeqDB\&enzyme=Trypsin&access_method=Accession+Number&accession_num=AFK19975.2&coverage_map=0+29+22+35+27+11+18) | 6.91e+006 |
| Phosphate ABC transporter periplasmic substrate-binding protein | [AFK20069.1](http://smserver.sti.ua.es/millhtml/SM_instruct/servadmn.htm#update_acclinks?AFK20069.1) | 7 | 6 | 96.28 | [26.7](http://smserver.sti.ua.es/millbin/msdigest.cgi?missed_cleavages=2&msparams_dir=msparams_mill/&hide_protein_sequence=2&database=PA_haloferax_mediterranei_genbank&seqdb_dir=D:\SeqDB\&enzyme=Trypsin&access_method=Accession+Number&accession_num=AFK20069.1&coverage_map=0+85+51+167+41) | 7.18e+006 |
| Tryptophanase | [AFK17753.2](http://smserver.sti.ua.es/millhtml/SM_instruct/servadmn.htm#update_acclinks?AFK17753.2) | 6 | 6 | 96.25 | [23.6](http://smserver.sti.ua.es/millbin/msdigest.cgi?missed_cleavages=2&msparams_dir=msparams_mill/&hide_protein_sequence=2&database=PA_haloferax_mediterranei_genbank&seqdb_dir=D:\SeqDB\&enzyme=Trypsin&access_method=Accession+Number&accession_num=AFK17753.2&coverage_map=0+62+19+15+27+115+14+25+16+10+11+59+19+56) | 7.72e+006 |
| Nitrate reductase beta chain (plasmid) | [AFK20938.1](http://smserver.sti.ua.es/millhtml/SM_instruct/servadmn.htm#update_acclinks?AFK20938.1) | 7 | 7 | 95.18 | [19.3](http://smserver.sti.ua.es/millbin/msdigest.cgi?missed_cleavages=2&msparams_dir=msparams_mill/&hide_protein_sequence=2&database=PA_haloferax_mediterranei_genbank&seqdb_dir=D:\SeqDB\&enzyme=Trypsin&access_method=Accession+Number&accession_num=AFK20938.1&coverage_map=0+40+9+118+13+5+9+104+20+7+17+10) | 9.26e+006 |
| Dihydrolipoamide dehydrogenase | [AFK20619.1](http://smserver.sti.ua.es/millhtml/SM_instruct/servadmn.htm#update_acclinks?AFK20619.1) | 8 | 6 | 94.77 | [20](http://smserver.sti.ua.es/millbin/msdigest.cgi?missed_cleavages=2&msparams_dir=msparams_mill/&hide_protein_sequence=2&database=PA_haloferax_mediterranei_genbank&seqdb_dir=D:\SeqDB\&enzyme=Trypsin&access_method=Accession+Number&accession_num=AFK20619.1&coverage_map=0+27+13+51+13+48+18+101+7+19+9+134+35) | 3.66e+006 |
| Putative cation-transporting ATPase | [AFK18658.2](http://smserver.sti.ua.es/millhtml/SM_instruct/servadmn.htm#update_acclinks?AFK18658.2) | 6 | 6 | 94.76 | [11.6](http://smserver.sti.ua.es/millbin/msdigest.cgi?missed_cleavages=2&msparams_dir=msparams_mill/&hide_protein_sequence=2&database=PA_haloferax_mediterranei_genbank&seqdb_dir=D:\SeqDB\&enzyme=Trypsin&access_method=Accession+Number&accession_num=AFK18658.2&coverage_map=0+71+14+40+19+180+22+59+12+109+16+114+11+143) | 1.76e+006 |
| NADH dehydrogenase | [AFK19343.1](http://smserver.sti.ua.es/millhtml/SM_instruct/servadmn.htm#update_acclinks?AFK19343.1) | 6 | 5 | 94.27 | [27.4](http://smserver.sti.ua.es/millbin/msdigest.cgi?missed_cleavages=2&msparams_dir=msparams_mill/&hide_protein_sequence=2&database=PA_haloferax_mediterranei_genbank&seqdb_dir=D:\SeqDB\&enzyme=Trypsin&access_method=Accession+Number&accession_num=AFK19343.1&coverage_map=0+51+16+84+21+33+16+62+38+31+18+27) | 2.88e+006 |
| ABC-type dipeptide/oligopeptide/nickel transport system, substrate binding protein (plasmid) | [AFK20740.1](http://smserver.sti.ua.es/millhtml/SM_instruct/servadmn.htm#update_acclinks?AFK20740.1) | 8 | 6 | 92.84 | [14.8](http://smserver.sti.ua.es/millbin/msdigest.cgi?missed_cleavages=2&msparams_dir=msparams_mill/&hide_protein_sequence=2&database=PA_haloferax_mediterranei_genbank&seqdb_dir=D:\SeqDB\&enzyme=Trypsin&access_method=Accession+Number&accession_num=AFK20740.1&coverage_map=0+91+8+48+40+9+8+19+13+44+17+282) | 4.71e+006 |
| Hypothetical protein HFX_1586 | [AFK19292.1](http://smserver.sti.ua.es/millhtml/SM_instruct/servadmn.htm#update_acclinks?AFK19292.1) | 7 | 7 | 92.08 | [20.1](http://smserver.sti.ua.es/millbin/msdigest.cgi?missed_cleavages=2&msparams_dir=msparams_mill/&hide_protein_sequence=2&database=PA_haloferax_mediterranei_genbank&seqdb_dir=D:\SeqDB\&enzyme=Trypsin&access_method=Accession+Number&accession_num=AFK19292.1&coverage_map=0+115+63+110+21+8+20+180) | 5.20e+006 |
| Putative mechanosensitive ion channel | [AFK19964.1](http://smserver.sti.ua.es/millhtml/SM_instruct/servadmn.htm#update_acclinks?AFK19964.1) | 9 | 5 | 91.95 | [31.6](http://smserver.sti.ua.es/millbin/msdigest.cgi?missed_cleavages=2&msparams_dir=msparams_mill/&hide_protein_sequence=2&database=PA_haloferax_mediterranei_genbank&seqdb_dir=D:\SeqDB\&enzyme=Trypsin&access_method=Accession+Number&accession_num=AFK19964.1&coverage_map=0+53+7+99+30+1+17+9+41+43) | 1.11e+007 |
| Hypothetical protein HFX_2088 | [AFK19779.1](http://smserver.sti.ua.es/millhtml/SM_instruct/servadmn.htm#update_acclinks?AFK19779.1) | 7 | 6 | 91.57 | [23.3](http://smserver.sti.ua.es/millbin/msdigest.cgi?missed_cleavages=2&msparams_dir=msparams_mill/&hide_protein_sequence=2&database=PA_haloferax_mediterranei_genbank&seqdb_dir=D:\SeqDB\&enzyme=Trypsin&access_method=Accession+Number&accession_num=AFK19779.1&coverage_map=0+200+10+22+18+36+19+5+11+21+32+11) | 3.04e+006 |
| Hypothetical protein HFX_1589 | [AFK19295.1](http://smserver.sti.ua.es/millhtml/SM_instruct/servadmn.htm#update_acclinks?AFK19295.1) | 5 | 5 | 91.00 | [17.5](http://smserver.sti.ua.es/millbin/msdigest.cgi?missed_cleavages=2&msparams_dir=msparams_mill/&hide_protein_sequence=2&database=PA_haloferax_mediterranei_genbank&seqdb_dir=D:\SeqDB\&enzyme=Trypsin&access_method=Accession+Number&accession_num=AFK19295.1&coverage_map=0+94+25+169+16+21+9+53+45+108) | 6.28e+006 |
| NADH dehydrogenase, subunit B (ubiquinone) | [AFK18695.1](http://smserver.sti.ua.es/millhtml/SM_instruct/servadmn.htm#update_acclinks?AFK18695.1) | 9 | 6 | 90.67 | [35.1](http://smserver.sti.ua.es/millbin/msdigest.cgi?missed_cleavages=2&msparams_dir=msparams_mill/&hide_protein_sequence=2&database=PA_haloferax_mediterranei_genbank&seqdb_dir=D:\SeqDB\&enzyme=Trypsin&access_method=Accession+Number&accession_num=AFK18695.1&coverage_map=0+36+12+3+6+30+7+4+14+71+43+7) | 3.53e+007 |
| 3-hydroxyacyl-CoA dehydrogenase | [AFK19216.2](http://smserver.sti.ua.es/millhtml/SM_instruct/servadmn.htm#update_acclinks?AFK19216.2) | 7 | 5 | 90.66 | [24.8](http://smserver.sti.ua.es/millbin/msdigest.cgi?missed_cleavages=2&msparams_dir=msparams_mill/&hide_protein_sequence=2&database=PA_haloferax_mediterranei_genbank&seqdb_dir=D:\SeqDB\&enzyme=Trypsin&access_method=Accession+Number&accession_num=AFK19216.2&coverage_map=0+74+27+61+44+80) | 2.17e+007 |
| Hypothetical protein HFX_6278 (plasmid) | [AFK21400.1](http://smserver.sti.ua.es/millhtml/SM_instruct/servadmn.htm#update_acclinks?AFK21400.1) | 7 | 5 | 89.96 | [41.7](http://smserver.sti.ua.es/millbin/msdigest.cgi?missed_cleavages=2&msparams_dir=msparams_mill/&hide_protein_sequence=2&database=PA_haloferax_mediterranei_genbank&seqdb_dir=D:\SeqDB\&enzyme=Trypsin&access_method=Accession+Number&accession_num=AFK21400.1&coverage_map=0+65+18+9+35) | 2.25e+007 |
| Hypothetical protein HFX_2509 | [AFK20191.2](http://smserver.sti.ua.es/millhtml/SM_instruct/servadmn.htm#update_acclinks?AFK20191.2) | 6 | 6 | 87.81 | [17.7](http://smserver.sti.ua.es/millbin/msdigest.cgi?missed_cleavages=2&msparams_dir=msparams_mill/&hide_protein_sequence=2&database=PA_haloferax_mediterranei_genbank&seqdb_dir=D:\SeqDB\&enzyme=Trypsin&access_method=Accession+Number&accession_num=AFK20191.2&coverage_map=0+7+43+4+11+240) | 8.85e+006 |
| Phosphonates ABC transporter ATP-binding protein | [AFK19846.1](http://smserver.sti.ua.es/millhtml/SM_instruct/servadmn.htm#update_acclinks?AFK19846.1) | 6 | 5 | 86.33 | [32.5](http://smserver.sti.ua.es/millbin/msdigest.cgi?missed_cleavages=2&msparams_dir=msparams_mill/&hide_protein_sequence=2&database=PA_haloferax_mediterranei_genbank&seqdb_dir=D:\SeqDB\&enzyme=Trypsin&access_method=Accession+Number&accession_num=AFK19846.1&coverage_map=0+93+11+28+19+14+28+22+28+21) | 5.34e+006 |
| Hypothetical protein HFX_6053 (plasmid) | [AFK21180.1](http://smserver.sti.ua.es/millhtml/SM_instruct/servadmn.htm#update_acclinks?AFK21180.1) | 7 | 6 | 86.27 | [25.2](http://smserver.sti.ua.es/millbin/msdigest.cgi?missed_cleavages=2&msparams_dir=msparams_mill/&hide_protein_sequence=2&database=PA_haloferax_mediterranei_genbank&seqdb_dir=D:\SeqDB\&enzyme=Trypsin&access_method=Accession+Number&accession_num=AFK21180.1&coverage_map=0+21+30+99+17+75+23+32+19+37) | 4.00e+006 |
| Electron transfer flavoprotein beta subunit | [AFK20417.2](http://smserver.sti.ua.es/millhtml/SM_instruct/servadmn.htm#update_acclinks?AFK20417.2) | 6 | 5 | 85.13 | [28.6](http://smserver.sti.ua.es/millbin/msdigest.cgi?missed_cleavages=2&msparams_dir=msparams_mill/&hide_protein_sequence=2&database=PA_haloferax_mediterranei_genbank&seqdb_dir=D:\SeqDB\&enzyme=Trypsin&access_method=Accession+Number&accession_num=AFK20417.2&coverage_map=0+30+20+18+21+73+31+18+11+68) | 4.98e+006 |
| Hemerythrin HHE cation binding region (plasmid) | [AFK20978.1](http://smserver.sti.ua.es/millhtml/SM_instruct/servadmn.htm#update_acclinks?AFK20978.1) | 6 | 5 | 84.41 | [34.4](http://smserver.sti.ua.es/millbin/msdigest.cgi?missed_cleavages=2&msparams_dir=msparams_mill/&hide_protein_sequence=2&database=PA_haloferax_mediterranei_genbank&seqdb_dir=D:\SeqDB\&enzyme=Trypsin&access_method=Accession+Number&accession_num=AFK20978.1&coverage_map=0+64+44+17+39+77) | 8.25e+006 |
| NADH dehydrogenase-like complex, subunit I | [AFK18698.1](http://smserver.sti.ua.es/millhtml/SM_instruct/servadmn.htm#update_acclinks?AFK18698.1) | 5 | 5 | 83.72 | [50.9](http://smserver.sti.ua.es/millbin/msdigest.cgi?missed_cleavages=2&msparams_dir=msparams_mill/&hide_protein_sequence=2&database=PA_haloferax_mediterranei_genbank&seqdb_dir=D:\SeqDB\&enzyme=Trypsin&access_method=Accession+Number&accession_num=AFK18698.1&coverage_map=0+13+22+11+24+16+24+17+8+18) | 9.82e+006 |
| NADH dehydrogenase 32K chain-like protein | [AFK17846.1](http://smserver.sti.ua.es/millhtml/SM_instruct/servadmn.htm#update_acclinks?AFK17846.1) | 6 | 6 | 83.69 | [25.4](http://smserver.sti.ua.es/millbin/msdigest.cgi?missed_cleavages=2&msparams_dir=msparams_mill/&hide_protein_sequence=2&database=PA_haloferax_mediterranei_genbank&seqdb_dir=D:\SeqDB\&enzyme=Trypsin&access_method=Accession+Number&accession_num=AFK17846.1&coverage_map=0+2+12+74+24+11+17+87+10+36+16+21) | 4.83e+006 |
| Branched-chain/neutral amino acids amide ABC transporter periplasmic substrate-binding protein | [AFK19529.1](http://smserver.sti.ua.es/millhtml/SM_instruct/servadmn.htm#update_acclinks?AFK19529.1) | 6 | 6 | 83.51 | [19.4](http://smserver.sti.ua.es/millbin/msdigest.cgi?missed_cleavages=2&msparams_dir=msparams_mill/&hide_protein_sequence=2&database=PA_haloferax_mediterranei_genbank&seqdb_dir=D:\SeqDB\&enzyme=Trypsin&access_method=Accession+Number&accession_num=AFK19529.1&coverage_map=0+69+28+167+39+39+16+68) | 1.09e+007 |
| NADH dehydrogenase, subunit H (ubiquinone) | [AFK18697.1](http://smserver.sti.ua.es/millhtml/SM_instruct/servadmn.htm#update_acclinks?AFK18697.1) | 6 | 5 | 83.45 | [13.1](http://smserver.sti.ua.es/millbin/msdigest.cgi?missed_cleavages=2&msparams_dir=msparams_mill/&hide_protein_sequence=2&database=PA_haloferax_mediterranei_genbank&seqdb_dir=D:\SeqDB\&enzyme=Trypsin&access_method=Accession+Number&accession_num=AFK18697.1&coverage_map=0+52+28+74+8+156+10+21) | 1.45e+007 |
| Isocitrate dehydrogenase (NADP+) | [AFK20291.1](http://smserver.sti.ua.es/millhtml/SM_instruct/servadmn.htm#update_acclinks?AFK20291.1) | 6 | 6 | 83.09 | [18.1](http://smserver.sti.ua.es/millbin/msdigest.cgi?missed_cleavages=2&msparams_dir=msparams_mill/&hide_protein_sequence=2&database=PA_haloferax_mediterranei_genbank&seqdb_dir=D:\SeqDB\&enzyme=Trypsin&access_method=Accession+Number&accession_num=AFK20291.1&coverage_map=0+50+11+37+12+99+19+51+12+90+13+16+9) | 3.30e+006 |
| Electron transfer flavoprotein alpha-subunit | [AFK18029.1](http://smserver.sti.ua.es/millhtml/SM_instruct/servadmn.htm#update_acclinks?AFK18029.1) | 6 | 5 | 82.38 | [38.4](http://smserver.sti.ua.es/millbin/msdigest.cgi?missed_cleavages=2&msparams_dir=msparams_mill/&hide_protein_sequence=2&database=PA_haloferax_mediterranei_genbank&seqdb_dir=D:\SeqDB\&enzyme=Trypsin&access_method=Accession+Number&accession_num=AFK18029.1&coverage_map=0+137+43+4+61+14+18+40) | 5.84e+006 |
| ABC transporter ATP-binding protein | [AFK18549.1](http://smserver.sti.ua.es/millhtml/SM_instruct/servadmn.htm#update_acclinks?AFK18549.1) | 5 | 5 | 82.38 | [21.2](http://smserver.sti.ua.es/millbin/msdigest.cgi?missed_cleavages=2&msparams_dir=msparams_mill/&hide_protein_sequence=2&database=PA_haloferax_mediterranei_genbank&seqdb_dir=D:\SeqDB\&enzyme=Trypsin&access_method=Accession+Number&accession_num=AFK18549.1&coverage_map=0+12+11+61+12+92+11+56+17+3+13+13) | 3.55e+006 |
| Hypothetical protein HFX_5226 (plasmid) | [AFK21058.1](http://smserver.sti.ua.es/millhtml/SM_instruct/servadmn.htm#update_acclinks?AFK21058.1) | 11 | 4 | 81.77 | [71.2](http://smserver.sti.ua.es/millbin/msdigest.cgi?missed_cleavages=2&msparams_dir=msparams_mill/&hide_protein_sequence=2&database=PA_haloferax_mediterranei_genbank&seqdb_dir=D:\SeqDB\&enzyme=Trypsin&access_method=Accession+Number&accession_num=AFK21058.1&coverage_map=0+29+72) | 1.52e+008 |
| Stress response protein (plasmid) | [AFK21518.1](http://smserver.sti.ua.es/millhtml/SM_instruct/servadmn.htm#update_acclinks?AFK21518.1) | 5 | 5 | 80.51 | [22.4](http://smserver.sti.ua.es/millbin/msdigest.cgi?missed_cleavages=2&msparams_dir=msparams_mill/&hide_protein_sequence=2&database=PA_haloferax_mediterranei_genbank&seqdb_dir=D:\SeqDB\&enzyme=Trypsin&access_method=Accession+Number&accession_num=AFK21518.1&coverage_map=0+43+16+69+10+13+9+81+18+5+12+13) | 1.10e+007 |
| Hypothetical protein HFX_0671 | [AFK18394.1](http://smserver.sti.ua.es/millhtml/SM_instruct/servadmn.htm#update_acclinks?AFK18394.1) | 6 | 5 | 80.04 | [24.8](http://smserver.sti.ua.es/millbin/msdigest.cgi?missed_cleavages=2&msparams_dir=msparams_mill/&hide_protein_sequence=2&database=PA_haloferax_mediterranei_genbank&seqdb_dir=D:\SeqDB\&enzyme=Trypsin&access_method=Accession+Number&accession_num=AFK18394.1&coverage_map=0+12+25+168+38+11) | 5.61e+006 |
| Transmembrane oligosaccharyl transferase / dolichyl-diphosphooligosaccharide--protein glycosyltransferase | [AFK19298.1](http://smserver.sti.ua.es/millhtml/SM_instruct/servadmn.htm#update_acclinks?AFK19298.1) | 6 | 6 | 79.58 | [7.3](http://smserver.sti.ua.es/millbin/msdigest.cgi?missed_cleavages=2&msparams_dir=msparams_mill/&hide_protein_sequence=2&database=PA_haloferax_mediterranei_genbank&seqdb_dir=D:\SeqDB\&enzyme=Trypsin&access_method=Accession+Number&accession_num=AFK19298.1&coverage_map=0+49+16+57+22+57+11+550+9+62+8+31+11+164) | 1.17e+006 |
| Naphthoate synthase | [AFK19237.1](http://smserver.sti.ua.es/millhtml/SM_instruct/servadmn.htm#update_acclinks?AFK19237.1) | 6 | 6 | 78.55 | [22.6](http://smserver.sti.ua.es/millbin/msdigest.cgi?missed_cleavages=2&msparams_dir=msparams_mill/&hide_protein_sequence=2&database=PA_haloferax_mediterranei_genbank&seqdb_dir=D:\SeqDB\&enzyme=Trypsin&access_method=Accession+Number&accession_num=AFK19237.1&coverage_map=0+27+8+9+19+2+16+43+8+143+18+12) | 4.68e+006 |
| Ferredoxin (2Fe-2S) | [AFK20674.1](http://smserver.sti.ua.es/millhtml/SM_instruct/servadmn.htm#update_acclinks?AFK20674.1) | 5 | 4 | 78.17 | [37.2](http://smserver.sti.ua.es/millbin/msdigest.cgi?missed_cleavages=2&msparams_dir=msparams_mill/&hide_protein_sequence=2&database=PA_haloferax_mediterranei_genbank&seqdb_dir=D:\SeqDB\&enzyme=Trypsin&access_method=Accession+Number&accession_num=AFK20674.1&coverage_map=0+65+48+16) | 2.37e+007 |
| Orotate phosphoribosyltransferase-like protein/conserved Entner-Douderoff pathway protein | [AFK18805.1](http://smserver.sti.ua.es/millhtml/SM_instruct/servadmn.htm#update_acclinks?AFK18805.1) | 6 | 5 | 77.81 | [29](http://smserver.sti.ua.es/millbin/msdigest.cgi?missed_cleavages=2&msparams_dir=msparams_mill/&hide_protein_sequence=2&database=PA_haloferax_mediterranei_genbank&seqdb_dir=D:\SeqDB\&enzyme=Trypsin&access_method=Accession+Number&accession_num=AFK18805.1&coverage_map=0+2+15+4+20+59+11+61+15+23) | 4.82e+006 |
| Prefoldin beta subunit | [AFK18347.1](http://smserver.sti.ua.es/millhtml/SM_instruct/servadmn.htm#update_acclinks?AFK18347.1) | 6 | 4 | 76.90 | [48.3](http://smserver.sti.ua.es/millbin/msdigest.cgi?missed_cleavages=2&msparams_dir=msparams_mill/&hide_protein_sequence=2&database=PA_haloferax_mediterranei_genbank&seqdb_dir=D:\SeqDB\&enzyme=Trypsin&access_method=Accession+Number&accession_num=AFK18347.1&coverage_map=0+12+18+11+14+3+28+38) | 5.17e+006 |
| Thiosulfate sulfurtransferase | [AFK17767.2](http://smserver.sti.ua.es/millhtml/SM_instruct/servadmn.htm#update_acclinks?AFK17767.2) | 6 | 5 | 76.79 | [25.8](http://smserver.sti.ua.es/millbin/msdigest.cgi?missed_cleavages=2&msparams_dir=msparams_mill/&hide_protein_sequence=2&database=PA_haloferax_mediterranei_genbank&seqdb_dir=D:\SeqDB\&enzyme=Trypsin&access_method=Accession+Number&accession_num=AFK17767.2&coverage_map=0+8+24+115+7+12+29+34+14+43) | 8.27e+006 |
| S-adenosyl-L-homocysteine hydrolase | [AFK17910.1](http://smserver.sti.ua.es/millhtml/SM_instruct/servadmn.htm#update_acclinks?AFK17910.1) | 5 | 5 | 76.77 | [22.4](http://smserver.sti.ua.es/millbin/msdigest.cgi?missed_cleavages=2&msparams_dir=msparams_mill/&hide_protein_sequence=2&database=PA_haloferax_mediterranei_genbank&seqdb_dir=D:\SeqDB\&enzyme=Trypsin&access_method=Accession+Number&accession_num=AFK17910.1&coverage_map=0+40+23+170+16+20+12+64+29+28+16+10) | 1.96e+006 |
| Membrane protease subunit, stomatin/prohibitin | [AFK17891.1](http://smserver.sti.ua.es/millhtml/SM_instruct/servadmn.htm#update_acclinks?AFK17891.1) | 6 | 5 | 76.04 | [20.4](http://smserver.sti.ua.es/millbin/msdigest.cgi?missed_cleavages=2&msparams_dir=msparams_mill/&hide_protein_sequence=2&database=PA_haloferax_mediterranei_genbank&seqdb_dir=D:\SeqDB\&enzyme=Trypsin&access_method=Accession+Number&accession_num=AFK17891.1&coverage_map=0+91+17+4+8+40+14+26+15+36+14+67) | 6.48e+006 |
| Putative hydrolase or acyltransferase of alpha/beta superfamily | [AFK19333.1](http://smserver.sti.ua.es/millhtml/SM_instruct/servadmn.htm#update_acclinks?AFK19333.1) | 5 | 5 | 75.96 | [29.2](http://smserver.sti.ua.es/millbin/msdigest.cgi?missed_cleavages=2&msparams_dir=msparams_mill/&hide_protein_sequence=2&database=PA_haloferax_mediterranei_genbank&seqdb_dir=D:\SeqDB\&enzyme=Trypsin&access_method=Accession+Number&accession_num=AFK19333.1&coverage_map=0+16+21+5+11+5+18+127+19+11+12+32) | 2.99e+006 |
| Halocyanin precursor-like protein | [AFK18843.1](http://smserver.sti.ua.es/millhtml/SM_instruct/servadmn.htm#update_acclinks?AFK18843.1) | 4 | 4 | 74.56 | [46.3](http://smserver.sti.ua.es/millbin/msdigest.cgi?missed_cleavages=2&msparams_dir=msparams_mill/&hide_protein_sequence=2&database=PA_haloferax_mediterranei_genbank&seqdb_dir=D:\SeqDB\&enzyme=Trypsin&access_method=Accession+Number&accession_num=AFK18843.1&coverage_map=0+51+8+38+69) | 2.06e+007 |
| Metalloprotease | [AFK18012.1](http://smserver.sti.ua.es/millhtml/SM_instruct/servadmn.htm#update_acclinks?AFK18012.1) | 7 | 5 | 74.25 | [16.1](http://smserver.sti.ua.es/millbin/msdigest.cgi?missed_cleavages=2&msparams_dir=msparams_mill/&hide_protein_sequence=2&database=PA_haloferax_mediterranei_genbank&seqdb_dir=D:\SeqDB\&enzyme=Trypsin&access_method=Accession+Number&accession_num=AFK18012.1&coverage_map=0+5+10+241+18+5+8+57+27+19) | 6.03e+006 |
| Phosphate ABC transporter ATP-binding protein | [AFK20072.1](http://smserver.sti.ua.es/millhtml/SM_instruct/servadmn.htm#update_acclinks?AFK20072.1) | 5 | 5 | 73.78 | [15.2](http://smserver.sti.ua.es/millbin/msdigest.cgi?missed_cleavages=2&msparams_dir=msparams_mill/&hide_protein_sequence=2&database=PA_haloferax_mediterranei_genbank&seqdb_dir=D:\SeqDB\&enzyme=Trypsin&access_method=Accession+Number&accession_num=AFK20072.1&coverage_map=0+107+8+2+13+26+14+4+10+112) | 3.03e+006 |
| Putative iron transport protein | [AFK19393.1](http://smserver.sti.ua.es/millhtml/SM_instruct/servadmn.htm#update_acclinks?AFK19393.1) | 5 | 4 | 73.39 | [18.8](http://smserver.sti.ua.es/millbin/msdigest.cgi?missed_cleavages=2&msparams_dir=msparams_mill/&hide_protein_sequence=2&database=PA_haloferax_mediterranei_genbank&seqdb_dir=D:\SeqDB\&enzyme=Trypsin&access_method=Accession+Number&accession_num=AFK19393.1&coverage_map=0+199+10+59+51+27+15+43) | 4.80e+006 |
| Gas-vesicle operon protein gvpA | [AFK19402.1](http://smserver.sti.ua.es/millhtml/SM_instruct/servadmn.htm#update_acclinks?AFK19402.1) | 6 | 4 | 73.29 | [57.6](http://smserver.sti.ua.es/millbin/msdigest.cgi?missed_cleavages=2&msparams_dir=msparams_mill/&hide_protein_sequence=2&database=PA_haloferax_mediterranei_genbank&seqdb_dir=D:\SeqDB\&enzyme=Trypsin&access_method=Accession+Number&accession_num=AFK19402.1&coverage_map=0+15+45+18) | 2.80e+007 |
| Superoxide dismutase, Fe-Mn family | [AFK20579.1](http://smserver.sti.ua.es/millhtml/SM_instruct/servadmn.htm#update_acclinks?AFK20579.1) | 5 | 4 | 72.85 | [26.6](http://smserver.sti.ua.es/millbin/msdigest.cgi?missed_cleavages=2&msparams_dir=msparams_mill/&hide_protein_sequence=2&database=PA_haloferax_mediterranei_genbank&seqdb_dir=D:\SeqDB\&enzyme=Trypsin&access_method=Accession+Number&accession_num=AFK20579.1&coverage_map=0+50+13+34+12+62+28) | 2.17e+007 |
| Superoxide dismutase, Fe-Mn family (plasmid) | [AFK21528.1](http://smserver.sti.ua.es/millhtml/SM_instruct/servadmn.htm#update_acclinks?AFK21528.1) | 4 | 3 | 56.81 | [20.5](http://smserver.sti.ua.es/millbin/msdigest.cgi?missed_cleavages=2&msparams_dir=msparams_mill/&hide_protein_sequence=2&database=PA_haloferax_mediterranei_genbank&seqdb_dir=D:\SeqDB\&enzyme=Trypsin&access_method=Accession+Number&accession_num=AFK21528.1&coverage_map=0+51+13+108+28) | 1.65e+007 |
| 4-aminobutyrate aminotransferase | [AFK19853.1](http://smserver.sti.ua.es/millhtml/SM_instruct/servadmn.htm#update_acclinks?AFK19853.1) | 5 | 5 | 72.71 | [16.7](http://smserver.sti.ua.es/millbin/msdigest.cgi?missed_cleavages=2&msparams_dir=msparams_mill/&hide_protein_sequence=2&database=PA_haloferax_mediterranei_genbank&seqdb_dir=D:\SeqDB\&enzyme=Trypsin&access_method=Accession+Number&accession_num=AFK19853.1&coverage_map=0+170+9+189+13+3+19+4+33+2) | 4.83e+006 |
| 4-aminobutyrate aminotransferase | [AFK20535.2](http://smserver.sti.ua.es/millhtml/SM_instruct/servadmn.htm#update_acclinks?AFK20535.2) | 2 | 2 | 27.87 | [4.2](http://smserver.sti.ua.es/millbin/msdigest.cgi?missed_cleavages=2&msparams_dir=msparams_mill/&hide_protein_sequence=2&database=PA_haloferax_mediterranei_genbank&seqdb_dir=D:\SeqDB\&enzyme=Trypsin&access_method=Accession+Number&accession_num=AFK20535.2&coverage_map=0+170+9+244+10+17) | 4.02e+006 |
| Protein-disulfide isomerase | [AFK18965.1](http://smserver.sti.ua.es/millhtml/SM_instruct/servadmn.htm#update_acclinks?AFK18965.1) | 6 | 4 | 71.66 | [29.7](http://smserver.sti.ua.es/millbin/msdigest.cgi?missed_cleavages=2&msparams_dir=msparams_mill/&hide_protein_sequence=2&database=PA_haloferax_mediterranei_genbank&seqdb_dir=D:\SeqDB\&enzyme=Trypsin&access_method=Accession+Number&accession_num=AFK18965.1&coverage_map=0+99+14+21+57+48) | 1.21e+007 |
| NADH dehydrogenase/oxidoreductase-like protein | [AFK19950.1](http://smserver.sti.ua.es/millhtml/SM_instruct/servadmn.htm#update_acclinks?AFK19950.1) | 7 | 4 | 71.52 | [28.5](http://smserver.sti.ua.es/millbin/msdigest.cgi?missed_cleavages=2&msparams_dir=msparams_mill/&hide_protein_sequence=2&database=PA_haloferax_mediterranei_genbank&seqdb_dir=D:\SeqDB\&enzyme=Trypsin&access_method=Accession+Number&accession_num=AFK19950.1&coverage_map=0+107+19+10+26+60+41+38) | 6.92e+006 |
| Hypothetical protein HFX_1239 | [AFK18952.1](http://smserver.sti.ua.es/millhtml/SM_instruct/servadmn.htm#update_acclinks?AFK18952.1) | 5 | 4 | 70.80 | [26.9](http://smserver.sti.ua.es/millbin/msdigest.cgi?missed_cleavages=2&msparams_dir=msparams_mill/&hide_protein_sequence=2&database=PA_haloferax_mediterranei_genbank&seqdb_dir=D:\SeqDB\&enzyme=Trypsin&access_method=Accession+Number&accession_num=AFK18952.1&coverage_map=0+7+17+71+17+58+18+5) | 4.78e+006 |
| Serine protein kinase | [AFK20509.1](http://smserver.sti.ua.es/millhtml/SM_instruct/servadmn.htm#update_acclinks?AFK20509.1) | 5 | 5 | 70.78 | [8.9](http://smserver.sti.ua.es/millbin/msdigest.cgi?missed_cleavages=2&msparams_dir=msparams_mill/&hide_protein_sequence=2&database=PA_haloferax_mediterranei_genbank&seqdb_dir=D:\SeqDB\&enzyme=Trypsin&access_method=Accession+Number&accession_num=AFK20509.1&coverage_map=0+298+10+25+10+70+14+39+16+52+12+144) | 1.45e+006 |
| Thioredoxin | [AFK18964.1](http://smserver.sti.ua.es/millhtml/SM_instruct/servadmn.htm#update_acclinks?AFK18964.1) | 6 | 4 | 70.55 | [34.8](http://smserver.sti.ua.es/millbin/msdigest.cgi?missed_cleavages=2&msparams_dir=msparams_mill/&hide_protein_sequence=2&database=PA_haloferax_mediterranei_genbank&seqdb_dir=D:\SeqDB\&enzyme=Trypsin&access_method=Accession+Number&accession_num=AFK18964.1&coverage_map=0+72+58+46+9+7) | 8.22e+006 |
| Electron transfer flavoprotein alpha subunit | [AFK19622.1](http://smserver.sti.ua.es/millhtml/SM_instruct/servadmn.htm#update_acclinks?AFK19622.1) | 6 | 4 | 70.55 | [16.3](http://smserver.sti.ua.es/millbin/msdigest.cgi?missed_cleavages=2&msparams_dir=msparams_mill/&hide_protein_sequence=2&database=PA_haloferax_mediterranei_genbank&seqdb_dir=D:\SeqDB\&enzyme=Trypsin&access_method=Accession+Number&accession_num=AFK19622.1&coverage_map=0+67+13+15+14+57+16+66+12+77) | 4.73e+006 |
| Xaa-Pro aminopeptidase | [AFK19088.1](http://smserver.sti.ua.es/millhtml/SM_instruct/servadmn.htm#update_acclinks?AFK19088.1) | 5 | 5 | 70.30 | [25.8](http://smserver.sti.ua.es/millbin/msdigest.cgi?missed_cleavages=2&msparams_dir=msparams_mill/&hide_protein_sequence=2&database=PA_haloferax_mediterranei_genbank&seqdb_dir=D:\SeqDB\&enzyme=Trypsin&access_method=Accession+Number&accession_num=AFK19088.1&coverage_map=0+95+47+51+11+64+43+80) | 1.76e+006 |
| Putative phosphate acetyltransferase | [AFK18715.1](http://smserver.sti.ua.es/millhtml/SM_instruct/servadmn.htm#update_acclinks?AFK18715.1) | 4 | 4 | 70.09 | [15.6](http://smserver.sti.ua.es/millbin/msdigest.cgi?missed_cleavages=2&msparams_dir=msparams_mill/&hide_protein_sequence=2&database=PA_haloferax_mediterranei_genbank&seqdb_dir=D:\SeqDB\&enzyme=Trypsin&access_method=Accession+Number&accession_num=AFK18715.1&coverage_map=0+15+12+148+32+30+13+114) | 4.19e+006 |
| Aspartate aminotransferase | [AFK18689.1](http://smserver.sti.ua.es/millhtml/SM_instruct/servadmn.htm#update_acclinks?AFK18689.1) | 5 | 4 | 69.88 | [17.7](http://smserver.sti.ua.es/millbin/msdigest.cgi?missed_cleavages=2&msparams_dir=msparams_mill/&hide_protein_sequence=2&database=PA_haloferax_mediterranei_genbank&seqdb_dir=D:\SeqDB\&enzyme=Trypsin&access_method=Accession+Number&accession_num=AFK18689.1&coverage_map=0+141+68+175) | 3.35e+006 |
| S-adenosylmethionine synthetase | [AFK19445.1](http://smserver.sti.ua.es/millhtml/SM_instruct/servadmn.htm#update_acclinks?AFK19445.1) | 4 | 4 | 69.62 | [14.1](http://smserver.sti.ua.es/millbin/msdigest.cgi?missed_cleavages=2&msparams_dir=msparams_mill/&hide_protein_sequence=2&database=PA_haloferax_mediterranei_genbank&seqdb_dir=D:\SeqDB\&enzyme=Trypsin&access_method=Accession+Number&accession_num=AFK19445.1&coverage_map=0+14+12+211+13+110+32+10) | 1.30e+006 |
| Acetyl-CoA acetyltransferase (plasmid) | [AFK21178.1](http://smserver.sti.ua.es/millhtml/SM_instruct/servadmn.htm#update_acclinks?AFK21178.1) | 5 | 5 | 69.01 | [24.2](http://smserver.sti.ua.es/millbin/msdigest.cgi?missed_cleavages=2&msparams_dir=msparams_mill/&hide_protein_sequence=2&database=PA_haloferax_mediterranei_genbank&seqdb_dir=D:\SeqDB\&enzyme=Trypsin&access_method=Accession+Number&accession_num=AFK21178.1&coverage_map=0+11+56+26+8+147+31+113) | 3.85e+006 |
| Ornithine cyclodeaminase | [AFK18155.1](http://smserver.sti.ua.es/millhtml/SM_instruct/servadmn.htm#update_acclinks?AFK18155.1) | 5 | 4 | 68.51 | [21](http://smserver.sti.ua.es/millbin/msdigest.cgi?missed_cleavages=2&msparams_dir=msparams_mill/&hide_protein_sequence=2&database=PA_haloferax_mediterranei_genbank&seqdb_dir=D:\SeqDB\&enzyme=Trypsin&access_method=Accession+Number&accession_num=AFK18155.1&coverage_map=0+55+17+98+41+19+12+90) | 4.16e+006 |
| Dolichyl-phosphate beta-D-mannosyltransferase | [AFK19835.1](http://smserver.sti.ua.es/millhtml/SM_instruct/servadmn.htm#update_acclinks?AFK19835.1) | 4 | 4 | 67.92 | [18.4](http://smserver.sti.ua.es/millbin/msdigest.cgi?missed_cleavages=2&msparams_dir=msparams_mill/&hide_protein_sequence=2&database=PA_haloferax_mediterranei_genbank&seqdb_dir=D:\SeqDB\&enzyme=Trypsin&access_method=Accession+Number&accession_num=AFK19835.1&coverage_map=0+3+51+84+14+200) | 2.58e+006 |
| Acyl-CoA dehydrogenase | [AFK20404.1](http://smserver.sti.ua.es/millhtml/SM_instruct/servadmn.htm#update_acclinks?AFK20404.1) | 4 | 4 | 67.87 | [17.3](http://smserver.sti.ua.es/millbin/msdigest.cgi?missed_cleavages=2&msparams_dir=msparams_mill/&hide_protein_sequence=2&database=PA_haloferax_mediterranei_genbank&seqdb_dir=D:\SeqDB\&enzyme=Trypsin&access_method=Accession+Number&accession_num=AFK20404.1&coverage_map=0+18+16+173+13+25+25+16+12+83) | 2.87e+006 |
| Phosphoenolpyruvate synthase / pyruvate, water dikinase | [AFK18505.1](http://smserver.sti.ua.es/millhtml/SM_instruct/servadmn.htm#update_acclinks?AFK18505.1) | 5 | 5 | 67.61 | [7.6](http://smserver.sti.ua.es/millbin/msdigest.cgi?missed_cleavages=2&msparams_dir=msparams_mill/&hide_protein_sequence=2&database=PA_haloferax_mediterranei_genbank&seqdb_dir=D:\SeqDB\&enzyme=Trypsin&access_method=Accession+Number&accession_num=AFK18505.1&coverage_map=0+10+10+81+12+225+12+129+13+22+11+230) | 7.54e+005 |
| Dipeptide/oligopeptide/nickel ABC transporter ATP-binding protein | [AFK17802.1](http://smserver.sti.ua.es/millhtml/SM_instruct/servadmn.htm#update_acclinks?AFK17802.1) | 5 | 5 | 66.63 | [13.6](http://smserver.sti.ua.es/millbin/msdigest.cgi?missed_cleavages=2&msparams_dir=msparams_mill/&hide_protein_sequence=2&database=PA_haloferax_mediterranei_genbank&seqdb_dir=D:\SeqDB\&enzyme=Trypsin&access_method=Accession+Number&accession_num=AFK17802.1&coverage_map=0+80+11+34+8+51+25+108+11+21+7+99) | 4.03e+006 |
| Carbohydrate ABC transporter substrate-binding protein, CUT1 family | [AFK20566.1](http://smserver.sti.ua.es/millhtml/SM_instruct/servadmn.htm#update_acclinks?AFK20566.1) | 3 | 3 | 66.12 | [13.6](http://smserver.sti.ua.es/millbin/msdigest.cgi?missed_cleavages=2&msparams_dir=msparams_mill/&hide_protein_sequence=2&database=PA_haloferax_mediterranei_genbank&seqdb_dir=D:\SeqDB\&enzyme=Trypsin&access_method=Accession+Number&accession_num=AFK20566.1&coverage_map=0+185+47+71+15+136) | 1.15e+007 |
| Hypothetical protein HFX_5081 (plasmid) | [AFK20916.2](http://smserver.sti.ua.es/millhtml/SM_instruct/servadmn.htm#update_acclinks?AFK20916.2) | 5 | 4 | 66.01 | [16.2](http://smserver.sti.ua.es/millbin/msdigest.cgi?missed_cleavages=2&msparams_dir=msparams_mill/&hide_protein_sequence=2&database=PA_haloferax_mediterranei_genbank&seqdb_dir=D:\SeqDB\&enzyme=Trypsin&access_method=Accession+Number&accession_num=AFK20916.2&coverage_map=0+2+17+221+16+12+8+28+12+11) | 1.33e+007 |
| Hypothetical protein HFX_2220 | [AFK19909.2](http://smserver.sti.ua.es/millhtml/SM_instruct/servadmn.htm#update_acclinks?AFK19909.2) | 4 | 4 | 65.81 | [2.6](http://smserver.sti.ua.es/millbin/msdigest.cgi?missed_cleavages=2&msparams_dir=msparams_mill/&hide_protein_sequence=2&database=PA_haloferax_mediterranei_genbank&seqdb_dir=D:\SeqDB\&enzyme=Trypsin&access_method=Accession+Number&accession_num=AFK19909.2&coverage_map=0+2045+12+25+19+22+29+93) | 1.90e+006 |
| Hypothetical protein HFX_0728 | [AFK18451.1](http://smserver.sti.ua.es/millhtml/SM_instruct/servadmn.htm#update_acclinks?AFK18451.1) | 5 | 5 | 65.54 | [37.3](http://smserver.sti.ua.es/millbin/msdigest.cgi?missed_cleavages=2&msparams_dir=msparams_mill/&hide_protein_sequence=2&database=PA_haloferax_mediterranei_genbank&seqdb_dir=D:\SeqDB\&enzyme=Trypsin&access_method=Accession+Number&accession_num=AFK18451.1&coverage_map=0+21+7+1+20+49+23+13) | 7.86e+006 |
| Hypothetical protein HFX_2616 | [AFK20294.1](http://smserver.sti.ua.es/millhtml/SM_instruct/servadmn.htm#update_acclinks?AFK20294.1) | 5 | 5 | 65.50 | [27.2](http://smserver.sti.ua.es/millbin/msdigest.cgi?missed_cleavages=2&msparams_dir=msparams_mill/&hide_protein_sequence=2&database=PA_haloferax_mediterranei_genbank&seqdb_dir=D:\SeqDB\&enzyme=Trypsin&access_method=Accession+Number&accession_num=AFK20294.1&coverage_map=0+46+24+8+9+33+15+34+10+34) | 1.84e+006 |
| Inorganic pyrophosphatase | [AFK18410.1](http://smserver.sti.ua.es/millhtml/SM_instruct/servadmn.htm#update_acclinks?AFK18410.1) | 5 | 5 | 65.44 | [31.6](http://smserver.sti.ua.es/millbin/msdigest.cgi?missed_cleavages=2&msparams_dir=msparams_mill/&hide_protein_sequence=2&database=PA_haloferax_mediterranei_genbank&seqdb_dir=D:\SeqDB\&enzyme=Trypsin&access_method=Accession+Number&accession_num=AFK18410.1&coverage_map=0+29+33+54+12+3+11+35) | 7.54e+006 |
| Hypothetical protein HFX_0661 | [AFK18385.1](http://smserver.sti.ua.es/millhtml/SM_instruct/servadmn.htm#update_acclinks?AFK18385.1) | 5 | 4 | 64.54 | [64.3](http://smserver.sti.ua.es/millbin/msdigest.cgi?missed_cleavages=2&msparams_dir=msparams_mill/&hide_protein_sequence=2&database=PA_haloferax_mediterranei_genbank&seqdb_dir=D:\SeqDB\&enzyme=Trypsin&access_method=Accession+Number&accession_num=AFK18385.1&coverage_map=0+3+10+28+46) | 1.61e+007 |
| 3-oxoacyl-[acyl-carrier protein] reductase | [AFK19230.1](http://smserver.sti.ua.es/millhtml/SM_instruct/servadmn.htm#update_acclinks?AFK19230.1) | 6 | 4 | 64.27 | [20.6](http://smserver.sti.ua.es/millbin/msdigest.cgi?missed_cleavages=2&msparams_dir=msparams_mill/&hide_protein_sequence=2&database=PA_haloferax_mediterranei_genbank&seqdb_dir=D:\SeqDB\&enzyme=Trypsin&access_method=Accession+Number&accession_num=AFK19230.1&coverage_map=0+7+13+148+28+9+11+36) | 7.49e+006 |
| FKBP-type peptidylprolyl isomerase 2 | [AFK19430.2](http://smserver.sti.ua.es/millhtml/SM_instruct/servadmn.htm#update_acclinks?AFK19430.2) | 4 | 4 | 63.44 | [15.4](http://smserver.sti.ua.es/millbin/msdigest.cgi?missed_cleavages=2&msparams_dir=msparams_mill/&hide_protein_sequence=2&database=PA_haloferax_mediterranei_genbank&seqdb_dir=D:\SeqDB\&enzyme=Trypsin&access_method=Accession+Number&accession_num=AFK19430.2&coverage_map=0+115+28+2+10+88+10+58) | 1.03e+007 |
| Electron transfer flavoprotein beta subunit | [AFK18030.1](http://smserver.sti.ua.es/millhtml/SM_instruct/servadmn.htm#update_acclinks?AFK18030.1) | 5 | 3 | 63.23 | [22.7](http://smserver.sti.ua.es/millbin/msdigest.cgi?missed_cleavages=2&msparams_dir=msparams_mill/&hide_protein_sequence=2&database=PA_haloferax_mediterranei_genbank&seqdb_dir=D:\SeqDB\&enzyme=Trypsin&access_method=Accession+Number&accession_num=AFK18030.1&coverage_map=0+8+15+60+17+62+28+74) | 5.91e+006 |
| Cell division protein FtsZ | [AFK18399.1](http://smserver.sti.ua.es/millhtml/SM_instruct/servadmn.htm#update_acclinks?AFK18399.1) | 4 | 4 | 63.22 | [18.5](http://smserver.sti.ua.es/millbin/msdigest.cgi?missed_cleavages=2&msparams_dir=msparams_mill/&hide_protein_sequence=2&database=PA_haloferax_mediterranei_genbank&seqdb_dir=D:\SeqDB\&enzyme=Trypsin&access_method=Accession+Number&accession_num=AFK18399.1&coverage_map=0+75+19+95+15+26+20+92+16+19) | 1.72e+006 |
| 2-oxoglutarate ferredoxin oxidoreductase, subunit alpha | [AFK18583.1](http://smserver.sti.ua.es/millhtml/SM_instruct/servadmn.htm#update_acclinks?AFK18583.1) | 4 | 4 | 62.88 | [11.6](http://smserver.sti.ua.es/millbin/msdigest.cgi?missed_cleavages=2&msparams_dir=msparams_mill/&hide_protein_sequence=2&database=PA_haloferax_mediterranei_genbank&seqdb_dir=D:\SeqDB\&enzyme=Trypsin&access_method=Accession+Number&accession_num=AFK18583.1&coverage_map=0+67+31+142+23+67+14+242) | 1.61e+006 |
| A-type ATP synthase subunit F | [AFK18040.1](http://smserver.sti.ua.es/millhtml/SM_instruct/servadmn.htm#update_acclinks?AFK18040.1) | 7 | 3 | 62.42 | [57.5](http://smserver.sti.ua.es/millbin/msdigest.cgi?missed_cleavages=2&msparams_dir=msparams_mill/&hide_protein_sequence=2&database=PA_haloferax_mediterranei_genbank&seqdb_dir=D:\SeqDB\&enzyme=Trypsin&access_method=Accession+Number&accession_num=AFK18040.1&coverage_map=0+23+42+8+19+14) | 1.81e+007 |
| Sulfatase-like protein | [AFK19290.1](http://smserver.sti.ua.es/millhtml/SM_instruct/servadmn.htm#update_acclinks?AFK19290.1) | 4 | 4 | 62.39 | [18.7](http://smserver.sti.ua.es/millbin/msdigest.cgi?missed_cleavages=2&msparams_dir=msparams_mill/&hide_protein_sequence=2&database=PA_haloferax_mediterranei_genbank&seqdb_dir=D:\SeqDB\&enzyme=Trypsin&access_method=Accession+Number&accession_num=AFK19290.1&coverage_map=0+55+25+50+6+126+36+19+18+119) | 1.98e+006 |
| Hypothetical protein HFX_0940 | [AFK18659.1](http://smserver.sti.ua.es/millhtml/SM_instruct/servadmn.htm#update_acclinks?AFK18659.1) | 4 | 4 | 62.27 | [15.9](http://smserver.sti.ua.es/millbin/msdigest.cgi?missed_cleavages=2&msparams_dir=msparams_mill/&hide_protein_sequence=2&database=PA_haloferax_mediterranei_genbank&seqdb_dir=D:\SeqDB\&enzyme=Trypsin&access_method=Accession+Number&accession_num=AFK18659.1&coverage_map=0+45+13+3+9+61+20+113) | 4.22e+006 |
| Citrate (si)-synthase | [AFK18167.1](http://smserver.sti.ua.es/millhtml/SM_instruct/servadmn.htm#update_acclinks?AFK18167.1) | 4 | 4 | 62.11 | [12.9](http://smserver.sti.ua.es/millbin/msdigest.cgi?missed_cleavages=2&msparams_dir=msparams_mill/&hide_protein_sequence=2&database=PA_haloferax_mediterranei_genbank&seqdb_dir=D:\SeqDB\&enzyme=Trypsin&access_method=Accession+Number&accession_num=AFK18167.1&coverage_map=0+32+28+14+11+48+10+236) | 6.02e+006 |
| Poly(3-hydroxyalkanoate) granule-associated protein(phasin) (plasmid) | [AFK21052.1](http://smserver.sti.ua.es/millhtml/SM_instruct/servadmn.htm#update_acclinks?AFK21052.1) | 8 | 4 | 62.01 | [24.6](http://smserver.sti.ua.es/millbin/msdigest.cgi?missed_cleavages=2&msparams_dir=msparams_mill/&hide_protein_sequence=2&database=PA_haloferax_mediterranei_genbank&seqdb_dir=D:\SeqDB\&enzyme=Trypsin&access_method=Accession+Number&accession_num=AFK21052.1&coverage_map=0+24+10+4+7+18+21+70) | 1.88e+008 |
| Proteasome subunit alpha | [AFK18815.1](http://smserver.sti.ua.es/millhtml/SM_instruct/servadmn.htm#update_acclinks?AFK18815.1) | 4 | 4 | 61.97 | [18.2](http://smserver.sti.ua.es/millbin/msdigest.cgi?missed_cleavages=2&msparams_dir=msparams_mill/&hide_protein_sequence=2&database=PA_haloferax_mediterranei_genbank&seqdb_dir=D:\SeqDB\&enzyme=Trypsin&access_method=Accession+Number&accession_num=AFK18815.1&coverage_map=0+12+18+27+11+3+17+164) | 4.41e+006 |
| Serine protease inhibitor family protein | [AFK20013.1](http://smserver.sti.ua.es/millhtml/SM_instruct/servadmn.htm#update_acclinks?AFK20013.1) | 4 | 4 | 61.64 | [13.6](http://smserver.sti.ua.es/millbin/msdigest.cgi?missed_cleavages=2&msparams_dir=msparams_mill/&hide_protein_sequence=2&database=PA_haloferax_mediterranei_genbank&seqdb_dir=D:\SeqDB\&enzyme=Trypsin&access_method=Accession+Number&accession_num=AFK20013.1&coverage_map=0+206+43+73+19+112) | 6.76e+006 |
| Copper-binding plastocyanin like protein (plasmid) | [AFK20927.1](http://smserver.sti.ua.es/millhtml/SM_instruct/servadmn.htm#update_acclinks?AFK20927.1) | 5 | 3 | 61.54 | [36.9](http://smserver.sti.ua.es/millbin/msdigest.cgi?missed_cleavages=2&msparams_dir=msparams_mill/&hide_protein_sequence=2&database=PA_haloferax_mediterranei_genbank&seqdb_dir=D:\SeqDB\&enzyme=Trypsin&access_method=Accession+Number&accession_num=AFK20927.1&coverage_map=0+81+48+6+16+22) | 1.08e+007 |
| Glycine dehydrogenase subunit 1 | [AFK20093.1](http://smserver.sti.ua.es/millhtml/SM_instruct/servadmn.htm#update_acclinks?AFK20093.1) | 4 | 4 | 61.36 | [12.7](http://smserver.sti.ua.es/millbin/msdigest.cgi?missed_cleavages=2&msparams_dir=msparams_mill/&hide_protein_sequence=2&database=PA_haloferax_mediterranei_genbank&seqdb_dir=D:\SeqDB\&enzyme=Trypsin&access_method=Accession+Number&accession_num=AFK20093.1&coverage_map=0+312+9+8+15+12+13+23+20+35) | 4.87e+006 |
| Putative sulfatase | [AFK19294.1](http://smserver.sti.ua.es/millhtml/SM_instruct/servadmn.htm#update_acclinks?AFK19294.1) | 4 | 4 | 61.01 | [12.7](http://smserver.sti.ua.es/millbin/msdigest.cgi?missed_cleavages=2&msparams_dir=msparams_mill/&hide_protein_sequence=2&database=PA_haloferax_mediterranei_genbank&seqdb_dir=D:\SeqDB\&enzyme=Trypsin&access_method=Accession+Number&accession_num=AFK19294.1&coverage_map=0+5+13+214+14+35+10+161+25+9) | 1.02e+006 |
| Phospho-adenylyl-sulfate reductase | [AFK18802.1](http://smserver.sti.ua.es/millhtml/SM_instruct/servadmn.htm#update_acclinks?AFK18802.1) | 4 | 4 | 60.91 | [19.8](http://smserver.sti.ua.es/millbin/msdigest.cgi?missed_cleavages=2&msparams_dir=msparams_mill/&hide_protein_sequence=2&database=PA_haloferax_mediterranei_genbank&seqdb_dir=D:\SeqDB\&enzyme=Trypsin&access_method=Accession+Number&accession_num=AFK18802.1&coverage_map=0+143+45+7+9+102+10+7) | 5.26e+006 |
| CBS domain-containing protein | [AFK20079.1](http://smserver.sti.ua.es/millhtml/SM_instruct/servadmn.htm#update_acclinks?AFK20079.1) | 4 | 4 | 60.73 | [14.4](http://smserver.sti.ua.es/millbin/msdigest.cgi?missed_cleavages=2&msparams_dir=msparams_mill/&hide_protein_sequence=2&database=PA_haloferax_mediterranei_genbank&seqdb_dir=D:\SeqDB\&enzyme=Trypsin&access_method=Accession+Number&accession_num=AFK20079.1&coverage_map=0+33+17+88+9+17+16+101+13+86) | 3.35e+006 |
| Prefoldin alpha subunit | [AFK17872.1](http://smserver.sti.ua.es/millhtml/SM_instruct/servadmn.htm#update_acclinks?AFK17872.1) | 5 | 4 | 60.56 | [47.7](http://smserver.sti.ua.es/millbin/msdigest.cgi?missed_cleavages=2&msparams_dir=msparams_mill/&hide_protein_sequence=2&database=PA_haloferax_mediterranei_genbank&seqdb_dir=D:\SeqDB\&enzyme=Trypsin&access_method=Accession+Number&accession_num=AFK17872.1&coverage_map=0+37+75+45) | 6.94e+006 |
| NADH dehydrogenase, subunit M (ubiquinone) | [AFK18703.1](http://smserver.sti.ua.es/millhtml/SM_instruct/servadmn.htm#update_acclinks?AFK18703.1) | 4 | 4 | 60.07 | [11.9](http://smserver.sti.ua.es/millbin/msdigest.cgi?missed_cleavages=2&msparams_dir=msparams_mill/&hide_protein_sequence=2&database=PA_haloferax_mediterranei_genbank&seqdb_dir=D:\SeqDB\&enzyme=Trypsin&access_method=Accession+Number&accession_num=AFK18703.1&coverage_map=0+236+50+85+11+127) | 6.33e+006 |
| ABC transporter ATP-binding protein | [AFK20685.1](http://smserver.sti.ua.es/millhtml/SM_instruct/servadmn.htm#update_acclinks?AFK20685.1) | 4 | 4 | 60.06 | [14.9](http://smserver.sti.ua.es/millbin/msdigest.cgi?missed_cleavages=2&msparams_dir=msparams_mill/&hide_protein_sequence=2&database=PA_haloferax_mediterranei_genbank&seqdb_dir=D:\SeqDB\&enzyme=Trypsin&access_method=Accession+Number&accession_num=AFK20685.1&coverage_map=0+76+14+2+13+130+21+65) | 1.55e+006 |
| Adenylosuccinate synthetase | [AFK18856.1](http://smserver.sti.ua.es/millhtml/SM_instruct/servadmn.htm#update_acclinks?AFK18856.1) | 4 | 4 | 60.03 | [13](http://smserver.sti.ua.es/millbin/msdigest.cgi?missed_cleavages=2&msparams_dir=msparams_mill/&hide_protein_sequence=2&database=PA_haloferax_mediterranei_genbank&seqdb_dir=D:\SeqDB\&enzyme=Trypsin&access_method=Accession+Number&accession_num=AFK18856.1&coverage_map=0+15+16+44+10+231+10+4+22+91) | 2.18e+006 |
| Hypothetical protein HFX_0292 | [AFK18031.1](http://smserver.sti.ua.es/millhtml/SM_instruct/servadmn.htm#update_acclinks?AFK18031.1) | 5 | 4 | 59.98 | [13.1](http://smserver.sti.ua.es/millbin/msdigest.cgi?missed_cleavages=2&msparams_dir=msparams_mill/&hide_protein_sequence=2&database=PA_haloferax_mediterranei_genbank&seqdb_dir=D:\SeqDB\&enzyme=Trypsin&access_method=Accession+Number&accession_num=AFK18031.1&coverage_map=0+57+8+134+15+104+11+16+16+19) | 5.08e+006 |
| Hypothetical protein HFX_1249 | [AFK18962.1](http://smserver.sti.ua.es/millhtml/SM_instruct/servadmn.htm#update_acclinks?AFK18962.1) | 4 | 4 | 58.90 | [10.1](http://smserver.sti.ua.es/millbin/msdigest.cgi?missed_cleavages=2&msparams_dir=msparams_mill/&hide_protein_sequence=2&database=PA_haloferax_mediterranei_genbank&seqdb_dir=D:\SeqDB\&enzyme=Trypsin&access_method=Accession+Number&accession_num=AFK18962.1&coverage_map=0+138+12+48+11+158+9+17+12+27) | 2.83e+006 |
| Hypothetical protein HFX_2289 | [AFK19976.1](http://smserver.sti.ua.es/millhtml/SM_instruct/servadmn.htm#update_acclinks?AFK19976.1) | 5 | 4 | 58.90 | [75](http://smserver.sti.ua.es/millbin/msdigest.cgi?missed_cleavages=2&msparams_dir=msparams_mill/&hide_protein_sequence=2&database=PA_haloferax_mediterranei_genbank&seqdb_dir=D:\SeqDB\&enzyme=Trypsin&access_method=Accession+Number&accession_num=AFK19976.1&coverage_map=0+3+19+4+32+10) | 3.89e+006 |
| Hypothetical protein HFX_6434 (plasmid) | [AFK21553.1](http://smserver.sti.ua.es/millhtml/SM_instruct/servadmn.htm#update_acclinks?AFK21553.1) | 5 | 4 | 58.69 | [13.9](http://smserver.sti.ua.es/millbin/msdigest.cgi?missed_cleavages=2&msparams_dir=msparams_mill/&hide_protein_sequence=2&database=PA_haloferax_mediterranei_genbank&seqdb_dir=D:\SeqDB\&enzyme=Trypsin&access_method=Accession+Number&accession_num=AFK21553.1&coverage_map=0+69+10+1+22+181+18+58) | 4.24e+006 |
| Enoyl-CoA hydratase | [AFK19171.2](http://smserver.sti.ua.es/millhtml/SM_instruct/servadmn.htm#update_acclinks?AFK19171.2) | 5 | 4 | 58.18 | [17.8](http://smserver.sti.ua.es/millbin/msdigest.cgi?missed_cleavages=2&msparams_dir=msparams_mill/&hide_protein_sequence=2&database=PA_haloferax_mediterranei_genbank&seqdb_dir=D:\SeqDB\&enzyme=Trypsin&access_method=Accession+Number&accession_num=AFK19171.2&coverage_map=0+30+19+98+11+1+7+55+9+27) | 4.33e+006 |
| ABC transporter ATP-binding protein (plasmid) | [AFK20923.1](http://smserver.sti.ua.es/millhtml/SM_instruct/servadmn.htm#update_acclinks?AFK20923.1) | 4 | 4 | 57.86 | [15.1](http://smserver.sti.ua.es/millbin/msdigest.cgi?missed_cleavages=2&msparams_dir=msparams_mill/&hide_protein_sequence=2&database=PA_haloferax_mediterranei_genbank&seqdb_dir=D:\SeqDB\&enzyme=Trypsin&access_method=Accession+Number&accession_num=AFK20923.1&coverage_map=0+112+17+11+26+132+7+26) | 1.22e+007 |
| Phosphate transport system regulatory protein PhoU | [AFK20073.1](http://smserver.sti.ua.es/millhtml/SM_instruct/servadmn.htm#update_acclinks?AFK20073.1) | 5 | 3 | 57.56 | [30.5](http://smserver.sti.ua.es/millbin/msdigest.cgi?missed_cleavages=2&msparams_dir=msparams_mill/&hide_protein_sequence=2&database=PA_haloferax_mediterranei_genbank&seqdb_dir=D:\SeqDB\&enzyme=Trypsin&access_method=Accession+Number&accession_num=AFK20073.1&coverage_map=0+9+18+28+24+14+27+106) | 2.51e+006 |
| Ferredoxin (2Fe-2S) | [AFK19572.1](http://smserver.sti.ua.es/millhtml/SM_instruct/servadmn.htm#update_acclinks?AFK19572.1) | 7 | 3 | 57.27 | [22.1](http://smserver.sti.ua.es/millbin/msdigest.cgi?missed_cleavages=2&msparams_dir=msparams_mill/&hide_protein_sequence=2&database=PA_haloferax_mediterranei_genbank&seqdb_dir=D:\SeqDB\&enzyme=Trypsin&access_method=Accession+Number&accession_num=AFK19572.1&coverage_map=0+26+17+1+15+113+11+11) | 2.07e+007 |
| Endoglucanase | [AFK18519.1](http://smserver.sti.ua.es/millhtml/SM_instruct/servadmn.htm#update_acclinks?AFK18519.1) | 4 | 4 | 57.08 | [17.5](http://smserver.sti.ua.es/millbin/msdigest.cgi?missed_cleavages=2&msparams_dir=msparams_mill/&hide_protein_sequence=2&database=PA_haloferax_mediterranei_genbank&seqdb_dir=D:\SeqDB\&enzyme=Trypsin&access_method=Accession+Number&accession_num=AFK18519.1&coverage_map=0+9+14+17+14+19+19+40+14+202) | 1.72e+006 |
| Succinate--CoA ligase, alpha subunit (ADP-forming) | [AFK20154.1](http://smserver.sti.ua.es/millhtml/SM_instruct/servadmn.htm#update_acclinks?AFK20154.1) | 4 | 4 | 56.98 | [22.4](http://smserver.sti.ua.es/millbin/msdigest.cgi?missed_cleavages=2&msparams_dir=msparams_mill/&hide_protein_sequence=2&database=PA_haloferax_mediterranei_genbank&seqdb_dir=D:\SeqDB\&enzyme=Trypsin&access_method=Accession+Number&accession_num=AFK20154.1&coverage_map=0+10+12+112+34+76+19+26) | 2.87e+006 |
| Uracil phosphoribosyltransferase | [AFK20636.1](http://smserver.sti.ua.es/millhtml/SM_instruct/servadmn.htm#update_acclinks?AFK20636.1) | 5 | 4 | 56.62 | [30.9](http://smserver.sti.ua.es/millbin/msdigest.cgi?missed_cleavages=2&msparams_dir=msparams_mill/&hide_protein_sequence=2&database=PA_haloferax_mediterranei_genbank&seqdb_dir=D:\SeqDB\&enzyme=Trypsin&access_method=Accession+Number&accession_num=AFK20636.1&coverage_map=0+25+10+39+12+52+48+40) | 2.16e+006 |
| Imidazolonepropionase (plasmid) | [AFK21452.1](http://smserver.sti.ua.es/millhtml/SM_instruct/servadmn.htm#update_acclinks?AFK21452.1) | 4 | 4 | 56.62 | [13.3](http://smserver.sti.ua.es/millbin/msdigest.cgi?missed_cleavages=2&msparams_dir=msparams_mill/&hide_protein_sequence=2&database=PA_haloferax_mediterranei_genbank&seqdb_dir=D:\SeqDB\&enzyme=Trypsin&access_method=Accession+Number&accession_num=AFK21452.1&coverage_map=0+150+12+62+9+76+22+22+12+47) | 8.40e+005 |
| Haloalkane dehalogenase | [AFK20076.1](http://smserver.sti.ua.es/millhtml/SM_instruct/servadmn.htm#update_acclinks?AFK20076.1) | 4 | 4 | 56.54 | [18.5](http://smserver.sti.ua.es/millbin/msdigest.cgi?missed_cleavages=2&msparams_dir=msparams_mill/&hide_protein_sequence=2&database=PA_haloferax_mediterranei_genbank&seqdb_dir=D:\SeqDB\&enzyme=Trypsin&access_method=Accession+Number&accession_num=AFK20076.1&coverage_map=0+54+17+112+6+78+34+6) | 1.34e+006 |
| Malate dehydrogenase | [AFK20690.2](http://smserver.sti.ua.es/millhtml/SM_instruct/servadmn.htm#update_acclinks?AFK20690.2) | 3 | 3 | 56.32 | [11.8](http://smserver.sti.ua.es/millbin/msdigest.cgi?missed_cleavages=2&msparams_dir=msparams_mill/&hide_protein_sequence=2&database=PA_haloferax_mediterranei_genbank&seqdb_dir=D:\SeqDB\&enzyme=Trypsin&access_method=Accession+Number&accession_num=AFK20690.2&coverage_map=0+24+13+149+23+95) | 2.42e+006 |
| Short chain dehydrogenase/ reductase | [AFK18407.1](http://smserver.sti.ua.es/millhtml/SM_instruct/servadmn.htm#update_acclinks?AFK18407.1) | 4 | 4 | 55.95 | [20.2](http://smserver.sti.ua.es/millbin/msdigest.cgi?missed_cleavages=2&msparams_dir=msparams_mill/&hide_protein_sequence=2&database=PA_haloferax_mediterranei_genbank&seqdb_dir=D:\SeqDB\&enzyme=Trypsin&access_method=Accession+Number&accession_num=AFK18407.1&coverage_map=0+4+13+18+28+58+16+144) | 1.67e+006 |
| Hypothetical protein HFX_2459 | [AFK20144.2](http://smserver.sti.ua.es/millhtml/SM_instruct/servadmn.htm#update_acclinks?AFK20144.2) | 3 | 3 | 55.35 | [4.5](http://smserver.sti.ua.es/millbin/msdigest.cgi?missed_cleavages=2&msparams_dir=msparams_mill/&hide_protein_sequence=2&database=PA_haloferax_mediterranei_genbank&seqdb_dir=D:\SeqDB\&enzyme=Trypsin&access_method=Accession+Number&accession_num=AFK20144.2&coverage_map=0+288+12+291+29+284) | 8.39e+006 |
| N-acyl-L-amino acid amidohydrolase | [AFK19179.1](http://smserver.sti.ua.es/millhtml/SM_instruct/servadmn.htm#update_acclinks?AFK19179.1) | 4 | 4 | 55.32 | [14.5](http://smserver.sti.ua.es/millbin/msdigest.cgi?missed_cleavages=2&msparams_dir=msparams_mill/&hide_protein_sequence=2&database=PA_haloferax_mediterranei_genbank&seqdb_dir=D:\SeqDB\&enzyme=Trypsin&access_method=Accession+Number&accession_num=AFK19179.1&coverage_map=0+41+17+42+33+276+12+6) | 1.66e+006 |
| Oxidoreductase | [AFK20381.1](http://smserver.sti.ua.es/millhtml/SM_instruct/servadmn.htm#update_acclinks?AFK20381.1) | 3 | 3 | 55.22 | [11.6](http://smserver.sti.ua.es/millbin/msdigest.cgi?missed_cleavages=2&msparams_dir=msparams_mill/&hide_protein_sequence=2&database=PA_haloferax_mediterranei_genbank&seqdb_dir=D:\SeqDB\&enzyme=Trypsin&access_method=Accession+Number&accession_num=AFK20381.1&coverage_map=0+141+13+153+29+26) | 8.42e+005 |
| Hypothetical protein HFX_2343 | [AFK20030.1](http://smserver.sti.ua.es/millhtml/SM_instruct/servadmn.htm#update_acclinks?AFK20030.1) | 4 | 4 | 55.18 | [13.6](http://smserver.sti.ua.es/millbin/msdigest.cgi?missed_cleavages=2&msparams_dir=msparams_mill/&hide_protein_sequence=2&database=PA_haloferax_mediterranei_genbank&seqdb_dir=D:\SeqDB\&enzyme=Trypsin&access_method=Accession+Number&accession_num=AFK20030.1&coverage_map=0+6+13+100+12+6+6+127+17+64) | 2.27e+006 |
| Sugar ABC transporter ATP-binding protein | [AFK20383.1](http://smserver.sti.ua.es/millhtml/SM_instruct/servadmn.htm#update_acclinks?AFK20383.1) | 6 | 3 | 54.78 | [12.9](http://smserver.sti.ua.es/millbin/msdigest.cgi?missed_cleavages=2&msparams_dir=msparams_mill/&hide_protein_sequence=2&database=PA_haloferax_mediterranei_genbank&seqdb_dir=D:\SeqDB\&enzyme=Trypsin&access_method=Accession+Number&accession_num=AFK20383.1&coverage_map=0+116+13+21+17+97+21+110) | 1.03e+007 |
| Glycine dehydrogenase subunit 2 | [AFK20092.1](http://smserver.sti.ua.es/millhtml/SM_instruct/servadmn.htm#update_acclinks?AFK20092.1) | 3 | 3 | 54.59 | [14.1](http://smserver.sti.ua.es/millbin/msdigest.cgi?missed_cleavages=2&msparams_dir=msparams_mill/&hide_protein_sequence=2&database=PA_haloferax_mediterranei_genbank&seqdb_dir=D:\SeqDB\&enzyme=Trypsin&access_method=Accession+Number&accession_num=AFK20092.1&coverage_map=0+10+11+201+29+169+27+26) | 1.30e+006 |
| Anthranilate phosphoribosyltransferase | [AFK19965.1](http://smserver.sti.ua.es/millhtml/SM_instruct/servadmn.htm#update_acclinks?AFK19965.1) | 3 | 3 | 54.32 | [13.2](http://smserver.sti.ua.es/millbin/msdigest.cgi?missed_cleavages=2&msparams_dir=msparams_mill/&hide_protein_sequence=2&database=PA_haloferax_mediterranei_genbank&seqdb_dir=D:\SeqDB\&enzyme=Trypsin&access_method=Accession+Number&accession_num=AFK19965.1&coverage_map=0+42+19+246+12+18+16+2) | 3.40e+006 |
| Hypothetical protein HFX_2260 | [AFK19948.1](http://smserver.sti.ua.es/millhtml/SM_instruct/servadmn.htm#update_acclinks?AFK19948.1) | 3 | 3 | 53.19 | [23.4](http://smserver.sti.ua.es/millbin/msdigest.cgi?missed_cleavages=2&msparams_dir=msparams_mill/&hide_protein_sequence=2&database=PA_haloferax_mediterranei_genbank&seqdb_dir=D:\SeqDB\&enzyme=Trypsin&access_method=Accession+Number&accession_num=AFK19948.1&coverage_map=0+29+12+113+46+47) | 1.81e+006 |
| Hypothetical protein HFX_1464 | [AFK19172.1](http://smserver.sti.ua.es/millhtml/SM_instruct/servadmn.htm#update_acclinks?AFK19172.1) | 4 | 4 | 52.98 | [22.8](http://smserver.sti.ua.es/millbin/msdigest.cgi?missed_cleavages=2&msparams_dir=msparams_mill/&hide_protein_sequence=2&database=PA_haloferax_mediterranei_genbank&seqdb_dir=D:\SeqDB\&enzyme=Trypsin&access_method=Accession+Number&accession_num=AFK19172.1&coverage_map=0+94+11+34+27) | 1.81e+006 |
| Putative inosine monophosphate dehydrogenase | [AFK17799.1](http://smserver.sti.ua.es/millhtml/SM_instruct/servadmn.htm#update_acclinks?AFK17799.1) | 4 | 4 | 52.75 | [16.5](http://smserver.sti.ua.es/millbin/msdigest.cgi?missed_cleavages=2&msparams_dir=msparams_mill/&hide_protein_sequence=2&database=PA_haloferax_mediterranei_genbank&seqdb_dir=D:\SeqDB\&enzyme=Trypsin&access_method=Accession+Number&accession_num=AFK17799.1&coverage_map=0+22+10+64+21+19+16+132) | 1.02e+006 |
| Nucleoside diphosphate kinase | [AFK20428.1](http://smserver.sti.ua.es/millhtml/SM_instruct/servadmn.htm#update_acclinks?AFK20428.1) | 3 | 3 | 52.65 | [26.6](http://smserver.sti.ua.es/millbin/msdigest.cgi?missed_cleavages=2&msparams_dir=msparams_mill/&hide_protein_sequence=2&database=PA_haloferax_mediterranei_genbank&seqdb_dir=D:\SeqDB\&enzyme=Trypsin&access_method=Accession+Number&accession_num=AFK20428.1&coverage_map=0+18+9+62+16+25+16+8) | 6.24e+006 |
| Universal stress protein | [AFK18810.1](http://smserver.sti.ua.es/millhtml/SM_instruct/servadmn.htm#update_acclinks?AFK18810.1) | 4 | 3 | 52.32 | [26](http://smserver.sti.ua.es/millbin/msdigest.cgi?missed_cleavages=2&msparams_dir=msparams_mill/&hide_protein_sequence=2&database=PA_haloferax_mediterranei_genbank&seqdb_dir=D:\SeqDB\&enzyme=Trypsin&access_method=Accession+Number&accession_num=AFK18810.1&coverage_map=0+41+27+48+10+16) | 1.18e+006 |
| Hypothetical protein HFX_0482 | [AFK18214.1](http://smserver.sti.ua.es/millhtml/SM_instruct/servadmn.htm#update_acclinks?AFK18214.1) | 6 | 3 | 52.29 | [32.8](http://smserver.sti.ua.es/millbin/msdigest.cgi?missed_cleavages=2&msparams_dir=msparams_mill/&hide_protein_sequence=2&database=PA_haloferax_mediterranei_genbank&seqdb_dir=D:\SeqDB\&enzyme=Trypsin&access_method=Accession+Number&accession_num=AFK18214.1&coverage_map=0+20+49+80) | 5.29e+006 |
| Glutamate dehydrogenase (NAD(P)+) | [AFK19867.1](http://smserver.sti.ua.es/millhtml/SM_instruct/servadmn.htm#update_acclinks?AFK19867.1) | 4 | 4 | 52.21 | [9.7](http://smserver.sti.ua.es/millbin/msdigest.cgi?missed_cleavages=2&msparams_dir=msparams_mill/&hide_protein_sequence=2&database=PA_haloferax_mediterranei_genbank&seqdb_dir=D:\SeqDB\&enzyme=Trypsin&access_method=Accession+Number&accession_num=AFK19867.1&coverage_map=0+40+14+16+12+133+7+195+10+12) | 3.00e+006 |
| Putative signal transduction protein with CBS domains (plasmid) | [AFK21146.1](http://smserver.sti.ua.es/millhtml/SM_instruct/servadmn.htm#update_acclinks?AFK21146.1) | 4 | 4 | 51.85 | [11.3](http://smserver.sti.ua.es/millbin/msdigest.cgi?missed_cleavages=2&msparams_dir=msparams_mill/&hide_protein_sequence=2&database=PA_haloferax_mediterranei_genbank&seqdb_dir=D:\SeqDB\&enzyme=Trypsin&access_method=Accession+Number&accession_num=AFK21146.1&coverage_map=0+120+10+200+15+49+8+7+20+36) | 1.15e+006 |
| Menaquinol--cytochrome-c reductase | [AFK20314.1](http://smserver.sti.ua.es/millhtml/SM_instruct/servadmn.htm#update_acclinks?AFK20314.1) | 4 | 3 | 51.84 | [13.5](http://smserver.sti.ua.es/millbin/msdigest.cgi?missed_cleavages=2&msparams_dir=msparams_mill/&hide_protein_sequence=2&database=PA_haloferax_mediterranei_genbank&seqdb_dir=D:\SeqDB\&enzyme=Trypsin&access_method=Accession+Number&accession_num=AFK20314.1&coverage_map=0+65+16+106+14+20+8+52) | 1.05e+007 |
| Ribulose-bisphosphate carboxylase large chain | [AFK18685.1](http://smserver.sti.ua.es/millhtml/SM_instruct/servadmn.htm#update_acclinks?AFK18685.1) | 4 | 4 | 51.73 | [14.2](http://smserver.sti.ua.es/millbin/msdigest.cgi?missed_cleavages=2&msparams_dir=msparams_mill/&hide_protein_sequence=2&database=PA_haloferax_mediterranei_genbank&seqdb_dir=D:\SeqDB\&enzyme=Trypsin&access_method=Accession+Number&accession_num=AFK18685.1&coverage_map=0+148+19+59+16+42+8+112+18+7) | 7.28e+005 |
| Putative intracellular protease | [AFK19063.1](http://smserver.sti.ua.es/millhtml/SM_instruct/servadmn.htm#update_acclinks?AFK19063.1) | 3 | 3 | 51.14 | [21.7](http://smserver.sti.ua.es/millbin/msdigest.cgi?missed_cleavages=2&msparams_dir=msparams_mill/&hide_protein_sequence=2&database=PA_haloferax_mediterranei_genbank&seqdb_dir=D:\SeqDB\&enzyme=Trypsin&access_method=Accession+Number&accession_num=AFK19063.1&coverage_map=0+63+43+92) | 1.10e+007 |
| Hypothetical protein HFX_2180 | [AFK19869.1](http://smserver.sti.ua.es/millhtml/SM_instruct/servadmn.htm#update_acclinks?AFK19869.1) | 4 | 3 | 51.09 | [8.7](http://smserver.sti.ua.es/millbin/msdigest.cgi?missed_cleavages=2&msparams_dir=msparams_mill/&hide_protein_sequence=2&database=PA_haloferax_mediterranei_genbank&seqdb_dir=D:\SeqDB\&enzyme=Trypsin&access_method=Accession+Number&accession_num=AFK19869.1&coverage_map=0+227+26+20+14+168) | 2.99e+006 |
| Thioredoxin | [AFK18139.1](http://smserver.sti.ua.es/millhtml/SM_instruct/servadmn.htm#update_acclinks?AFK18139.1) | 3 | 3 | 50.82 | [51.1](http://smserver.sti.ua.es/millbin/msdigest.cgi?missed_cleavages=2&msparams_dir=msparams_mill/&hide_protein_sequence=2&database=PA_haloferax_mediterranei_genbank&seqdb_dir=D:\SeqDB\&enzyme=Trypsin&access_method=Accession+Number&accession_num=AFK18139.1&coverage_map=0+4+13+20+32+19) | 1.94e+006 |
| Ferredoxin:NAD+ oxidoreductase | [AFK20041.1](http://smserver.sti.ua.es/millhtml/SM_instruct/servadmn.htm#update_acclinks?AFK20041.1) | 3 | 3 | 50.28 | [16.2](http://smserver.sti.ua.es/millbin/msdigest.cgi?missed_cleavages=2&msparams_dir=msparams_mill/&hide_protein_sequence=2&database=PA_haloferax_mediterranei_genbank&seqdb_dir=D:\SeqDB\&enzyme=Trypsin&access_method=Accession+Number&accession_num=AFK20041.1&coverage_map=0+70+32+96+20+145+15+35) | 1.38e+006 |
| Dihydroxyacetone kinase, DhaK subunit | [AFK19313.1](http://smserver.sti.ua.es/millhtml/SM_instruct/servadmn.htm#update_acclinks?AFK19313.1) | 3 | 3 | 49.81 | [17.8](http://smserver.sti.ua.es/millbin/msdigest.cgi?missed_cleavages=2&msparams_dir=msparams_mill/&hide_protein_sequence=2&database=PA_haloferax_mediterranei_genbank&seqdb_dir=D:\SeqDB\&enzyme=Trypsin&access_method=Accession+Number&accession_num=AFK19313.1&coverage_map=0+3+23+121+10+41+26+107) | 1.90e+006 |
| Gas-vesicle operon protein gvpH | [AFK19407.1](http://smserver.sti.ua.es/millhtml/SM_instruct/servadmn.htm#update_acclinks?AFK19407.1) | 3 | 3 | 49.59 | [17.1](http://smserver.sti.ua.es/millbin/msdigest.cgi?missed_cleavages=2&msparams_dir=msparams_mill/&hide_protein_sequence=2&database=PA_haloferax_mediterranei_genbank&seqdb_dir=D:\SeqDB\&enzyme=Trypsin&access_method=Accession+Number&accession_num=AFK19407.1&coverage_map=0+61+15+96+22+22) | 5.81e+006 |
| Proteasome subunit alpha | [AFK20587.1](http://smserver.sti.ua.es/millhtml/SM_instruct/servadmn.htm#update_acclinks?AFK20587.1) | 3 | 3 | 49.53 | [12.2](http://smserver.sti.ua.es/millbin/msdigest.cgi?missed_cleavages=2&msparams_dir=msparams_mill/&hide_protein_sequence=2&database=PA_haloferax_mediterranei_genbank&seqdb_dir=D:\SeqDB\&enzyme=Trypsin&access_method=Accession+Number&accession_num=AFK20587.1&coverage_map=0+21+8+5+8+106+14+82) | 2.05e+006 |
| Adenylate kinase | [AFK20192.2](http://smserver.sti.ua.es/millhtml/SM_instruct/servadmn.htm#update_acclinks?AFK20192.2) | 3 | 3 | 49.35 | [21.1](http://smserver.sti.ua.es/millbin/msdigest.cgi?missed_cleavages=2&msparams_dir=msparams_mill/&hide_protein_sequence=2&database=PA_haloferax_mediterranei_genbank&seqdb_dir=D:\SeqDB\&enzyme=Trypsin&access_method=Accession+Number&accession_num=AFK20192.2&coverage_map=0+4+11+6+17+126+16+28) | 2.36e+006 |
| Protein of unknown function DUF1486 | [AFK20429.1](http://smserver.sti.ua.es/millhtml/SM_instruct/servadmn.htm#update_acclinks?AFK20429.1) | 4 | 3 | 49.32 | [22.4](http://smserver.sti.ua.es/millbin/msdigest.cgi?missed_cleavages=2&msparams_dir=msparams_mill/&hide_protein_sequence=2&database=PA_haloferax_mediterranei_genbank&seqdb_dir=D:\SeqDB\&enzyme=Trypsin&access_method=Accession+Number&accession_num=AFK20429.1&coverage_map=0+54+11+42+11+5+15+27) | 2.38e+006 |
| FAD-dependent pyridine nucleotide-disulfide oxidoreductase | [AFK17859.1](http://smserver.sti.ua.es/millhtml/SM_instruct/servadmn.htm#update_acclinks?AFK17859.1) | 3 | 3 | 49.27 | [11.5](http://smserver.sti.ua.es/millbin/msdigest.cgi?missed_cleavages=2&msparams_dir=msparams_mill/&hide_protein_sequence=2&database=PA_haloferax_mediterranei_genbank&seqdb_dir=D:\SeqDB\&enzyme=Trypsin&access_method=Accession+Number&accession_num=AFK17859.1&coverage_map=0+4+16+94+10+54+18+185) | 1.04e+006 |
| Phenylacetate-CoA oxygenase subunit PaaA (plasmid) | [AFK21499.2](http://smserver.sti.ua.es/millhtml/SM_instruct/servadmn.htm#update_acclinks?AFK21499.2) | 3 | 3 | 49.27 | [11.4](http://smserver.sti.ua.es/millbin/msdigest.cgi?missed_cleavages=2&msparams_dir=msparams_mill/&hide_protein_sequence=2&database=PA_haloferax_mediterranei_genbank&seqdb_dir=D:\SeqDB\&enzyme=Trypsin&access_method=Accession+Number&accession_num=AFK21499.2&coverage_map=0+66+13+121+13+21+10+70) | 1.94e+006 |
| Aminopeptidase (leucyl aminopeptidase, aminopeptidase T) | [AFK18178.1](http://smserver.sti.ua.es/millhtml/SM_instruct/servadmn.htm#update_acclinks?AFK18178.1) | 4 | 3 | 48.95 | [14.1](http://smserver.sti.ua.es/millbin/msdigest.cgi?missed_cleavages=2&msparams_dir=msparams_mill/&hide_protein_sequence=2&database=PA_haloferax_mediterranei_genbank&seqdb_dir=D:\SeqDB\&enzyme=Trypsin&access_method=Accession+Number&accession_num=AFK18178.1&coverage_map=0+38+20+76+13+86+12+74) | 1.00e+006 |
| Hypothetical protein HFX_0970 | [AFK18688.1](http://smserver.sti.ua.es/millhtml/SM_instruct/servadmn.htm#update_acclinks?AFK18688.1) | 3 | 3 | 48.74 | [7.9](http://smserver.sti.ua.es/millbin/msdigest.cgi?missed_cleavages=2&msparams_dir=msparams_mill/&hide_protein_sequence=2&database=PA_haloferax_mediterranei_genbank&seqdb_dir=D:\SeqDB\&enzyme=Trypsin&access_method=Accession+Number&accession_num=AFK18688.1&coverage_map=0+104+13+83+11+82+12+146) | 1.24e+006 |
| Monoamine oxidase regulatory protein (plasmid) | [AFK21482.1](http://smserver.sti.ua.es/millhtml/SM_instruct/servadmn.htm#update_acclinks?AFK21482.1) | 3 | 3 | 48.52 | [26.9](http://smserver.sti.ua.es/millbin/msdigest.cgi?missed_cleavages=2&msparams_dir=msparams_mill/&hide_protein_sequence=2&database=PA_haloferax_mediterranei_genbank&seqdb_dir=D:\SeqDB\&enzyme=Trypsin&access_method=Accession+Number&accession_num=AFK21482.1&coverage_map=0+48+28+44+14+22) | 1.31e+006 |
| Proteasome beta subunit | [AFK19329.1](http://smserver.sti.ua.es/millhtml/SM_instruct/servadmn.htm#update_acclinks?AFK19329.1) | 4 | 3 | 48.01 | [20.8](http://smserver.sti.ua.es/millbin/msdigest.cgi?missed_cleavages=2&msparams_dir=msparams_mill/&hide_protein_sequence=2&database=PA_haloferax_mediterranei_genbank&seqdb_dir=D:\SeqDB\&enzyme=Trypsin&access_method=Accession+Number&accession_num=AFK19329.1&coverage_map=0+56+12+21+20+9+19+108) | 2.82e+006 |
| NADH dehydrogenase, subunit L (ubiquinone) | [AFK18702.1](http://smserver.sti.ua.es/millhtml/SM_instruct/servadmn.htm#update_acclinks?AFK18702.1) | 5 | 3 | 47.62 | [7.8](http://smserver.sti.ua.es/millbin/msdigest.cgi?missed_cleavages=2&msparams_dir=msparams_mill/&hide_protein_sequence=2&database=PA_haloferax_mediterranei_genbank&seqdb_dir=D:\SeqDB\&enzyme=Trypsin&access_method=Accession+Number&accession_num=AFK18702.1&coverage_map=0+590+53+35) | 2.22e+006 |
| Hypothetical protein HFX_2780 | [AFK20456.1](http://smserver.sti.ua.es/millhtml/SM_instruct/servadmn.htm#update_acclinks?AFK20456.1) | 3 | 3 | 47.62 | [12.8](http://smserver.sti.ua.es/millbin/msdigest.cgi?missed_cleavages=2&msparams_dir=msparams_mill/&hide_protein_sequence=2&database=PA_haloferax_mediterranei_genbank&seqdb_dir=D:\SeqDB\&enzyme=Trypsin&access_method=Accession+Number&accession_num=AFK20456.1&coverage_map=0+61+18+76+11+32+12+110) | 9.52e+005 |
| Oxidoreductase | [AFK18724.1](http://smserver.sti.ua.es/millhtml/SM_instruct/servadmn.htm#update_acclinks?AFK18724.1) | 3 | 3 | 47.41 | [12.6](http://smserver.sti.ua.es/millbin/msdigest.cgi?missed_cleavages=2&msparams_dir=msparams_mill/&hide_protein_sequence=2&database=PA_haloferax_mediterranei_genbank&seqdb_dir=D:\SeqDB\&enzyme=Trypsin&access_method=Accession+Number&accession_num=AFK18724.1&coverage_map=0+14+10+245+33+38) | 8.85e+005 |
| Ribose-1,5-bisphosphate isomerase (ribulose-bisphosphate forming) | [AFK18682.1](http://smserver.sti.ua.es/millhtml/SM_instruct/servadmn.htm#update_acclinks?AFK18682.1) | 4 | 4 | 47.31 | [17.9](http://smserver.sti.ua.es/millbin/msdigest.cgi?missed_cleavages=2&msparams_dir=msparams_mill/&hide_protein_sequence=2&database=PA_haloferax_mediterranei_genbank&seqdb_dir=D:\SeqDB\&enzyme=Trypsin&access_method=Accession+Number&accession_num=AFK18682.1&coverage_map=0+11+22+126+26+96+9+27) | 1.43e+006 |
| DMSO reductase family type II enzyme, heme b subunit (plasmid) | [AFK20937.1](http://smserver.sti.ua.es/millhtml/SM_instruct/servadmn.htm#update_acclinks?AFK20937.1) | 3 | 3 | 47.03 | [13.4](http://smserver.sti.ua.es/millbin/msdigest.cgi?missed_cleavages=2&msparams_dir=msparams_mill/&hide_protein_sequence=2&database=PA_haloferax_mediterranei_genbank&seqdb_dir=D:\SeqDB\&enzyme=Trypsin&access_method=Accession+Number&accession_num=AFK20937.1&coverage_map=0+100+10+32+27+107) | 1.43e+006 |
| Hypothetical protein HFX_2647 | [AFK20325.1](http://smserver.sti.ua.es/millhtml/SM_instruct/servadmn.htm#update_acclinks?AFK20325.1) | 3 | 3 | 46.86 | [11.7](http://smserver.sti.ua.es/millbin/msdigest.cgi?missed_cleavages=2&msparams_dir=msparams_mill/&hide_protein_sequence=2&database=PA_haloferax_mediterranei_genbank&seqdb_dir=D:\SeqDB\&enzyme=Trypsin&access_method=Accession+Number&accession_num=AFK20325.1&coverage_map=0+20+14+122+10+2+11+119) | 2.06e+006 |
| Hypothetical protein HFX_0016 | [AFK17760.1](http://smserver.sti.ua.es/millhtml/SM_instruct/servadmn.htm#update_acclinks?AFK17760.1) | 3 | 3 | 46.79 | [19.7](http://smserver.sti.ua.es/millbin/msdigest.cgi?missed_cleavages=2&msparams_dir=msparams_mill/&hide_protein_sequence=2&database=PA_haloferax_mediterranei_genbank&seqdb_dir=D:\SeqDB\&enzyme=Trypsin&access_method=Accession+Number&accession_num=AFK17760.1&coverage_map=0+60+21+38+17+56) | 2.50e+006 |
| ABC-type transport system ATP-binding protein | [AFK20086.1](http://smserver.sti.ua.es/millhtml/SM_instruct/servadmn.htm#update_acclinks?AFK20086.1) | 4 | 3 | 46.69 | [16.5](http://smserver.sti.ua.es/millbin/msdigest.cgi?missed_cleavages=2&msparams_dir=msparams_mill/&hide_protein_sequence=2&database=PA_haloferax_mediterranei_genbank&seqdb_dir=D:\SeqDB\&enzyme=Trypsin&access_method=Accession+Number&accession_num=AFK20086.1&coverage_map=0+70+21+48+17+73+6+31) | 2.86e+006 |
| Putative X-Pro dipeptidase | [AFK18714.1](http://smserver.sti.ua.es/millhtml/SM_instruct/servadmn.htm#update_acclinks?AFK18714.1) | 3 | 3 | 46.66 | [14.9](http://smserver.sti.ua.es/millbin/msdigest.cgi?missed_cleavages=2&msparams_dir=msparams_mill/&hide_protein_sequence=2&database=PA_haloferax_mediterranei_genbank&seqdb_dir=D:\SeqDB\&enzyme=Trypsin&access_method=Accession+Number&accession_num=AFK18714.1&coverage_map=0+119+19+78+15+33+21+83) | 7.20e+005 |
| Dodecin | [AFK20375.1](http://smserver.sti.ua.es/millhtml/SM_instruct/servadmn.htm#update_acclinks?AFK20375.1) | 3 | 3 | 46.57 | [69.1](http://smserver.sti.ua.es/millbin/msdigest.cgi?missed_cleavages=2&msparams_dir=msparams_mill/&hide_protein_sequence=2&database=PA_haloferax_mediterranei_genbank&seqdb_dir=D:\SeqDB\&enzyme=Trypsin&access_method=Accession+Number&accession_num=AFK20375.1&coverage_map=0+5+47+16) | 1.38e+007 |
| Succinate--CoA ligase beta subunit (ADP-forming) | [AFK20155.1](http://smserver.sti.ua.es/millhtml/SM_instruct/servadmn.htm#update_acclinks?AFK20155.1) | 3 | 3 | 46.33 | [11](http://smserver.sti.ua.es/millbin/msdigest.cgi?missed_cleavages=2&msparams_dir=msparams_mill/&hide_protein_sequence=2&database=PA_haloferax_mediterranei_genbank&seqdb_dir=D:\SeqDB\&enzyme=Trypsin&access_method=Accession+Number&accession_num=AFK20155.1&coverage_map=0+9+14+130+10+36+18+164) | 1.41e+006 |
| Acetyl-CoA C-ac(et)yltransferase | [AFK19698.1](http://smserver.sti.ua.es/millhtml/SM_instruct/servadmn.htm#update_acclinks?AFK19698.1) | 3 | 3 | 46.30 | [14.8](http://smserver.sti.ua.es/millbin/msdigest.cgi?missed_cleavages=2&msparams_dir=msparams_mill/&hide_protein_sequence=2&database=PA_haloferax_mediterranei_genbank&seqdb_dir=D:\SeqDB\&enzyme=Trypsin&access_method=Accession+Number&accession_num=AFK19698.1&coverage_map=0+25+34+49+13+41+9+206) | 9.25e+005 |
| Zinc-transporting ATPase (plasmid) | [AFK21399.1](http://smserver.sti.ua.es/millhtml/SM_instruct/servadmn.htm#update_acclinks?AFK21399.1) | 4 | 4 | 46.27 | [4.3](http://smserver.sti.ua.es/millbin/msdigest.cgi?missed_cleavages=2&msparams_dir=msparams_mill/&hide_protein_sequence=2&database=PA_haloferax_mediterranei_genbank&seqdb_dir=D:\SeqDB\&enzyme=Trypsin&access_method=Accession+Number&accession_num=AFK21399.1&coverage_map=0+78+11+351+15+279+12+136) | 9.77e+005 |
| Signal sequence peptidase | [AFK17747.2](http://smserver.sti.ua.es/millhtml/SM_instruct/servadmn.htm#update_acclinks?AFK17747.2) | 3 | 3 | 45.98 | [14.4](http://smserver.sti.ua.es/millbin/msdigest.cgi?missed_cleavages=2&msparams_dir=msparams_mill/&hide_protein_sequence=2&database=PA_haloferax_mediterranei_genbank&seqdb_dir=D:\SeqDB\&enzyme=Trypsin&access_method=Accession+Number&accession_num=AFK17747.2&coverage_map=0+79+28+78+14+92) | 2.20e+006 |
| Hypothetical protein HFX_1684 | [AFK19390.1](http://smserver.sti.ua.es/millhtml/SM_instruct/servadmn.htm#update_acclinks?AFK19390.1) | 4 | 2 | 45.97 | [21.6](http://smserver.sti.ua.es/millbin/msdigest.cgi?missed_cleavages=2&msparams_dir=msparams_mill/&hide_protein_sequence=2&database=PA_haloferax_mediterranei_genbank&seqdb_dir=D:\SeqDB\&enzyme=Trypsin&access_method=Accession+Number&accession_num=AFK19390.1&coverage_map=0+39+24+48) | 1.29e+007 |
| D-3-phosphoglycerate dehydrogenase | [AFK19716.1](http://smserver.sti.ua.es/millhtml/SM_instruct/servadmn.htm#update_acclinks?AFK19716.1) | 3 | 3 | 45.94 | [10](http://smserver.sti.ua.es/millbin/msdigest.cgi?missed_cleavages=2&msparams_dir=msparams_mill/&hide_protein_sequence=2&database=PA_haloferax_mediterranei_genbank&seqdb_dir=D:\SeqDB\&enzyme=Trypsin&access_method=Accession+Number&accession_num=AFK19716.1&coverage_map=0+5+9+39+13+116+9+117) | 1.33e+006 |
| Ca2+-transporting ATPase | [AFK18654.1](http://smserver.sti.ua.es/millhtml/SM_instruct/servadmn.htm#update_acclinks?AFK18654.1) | 3 | 3 | 45.62 | [4.3](http://smserver.sti.ua.es/millbin/msdigest.cgi?missed_cleavages=2&msparams_dir=msparams_mill/&hide_protein_sequence=2&database=PA_haloferax_mediterranei_genbank&seqdb_dir=D:\SeqDB\&enzyme=Trypsin&access_method=Accession+Number&accession_num=AFK18654.1&coverage_map=0+235+10+220+15+2+14+396) | 1.24e+006 |
| Arylsulfatase, choline-sulfatase | [AFK19291.1](http://smserver.sti.ua.es/millhtml/SM_instruct/servadmn.htm#update_acclinks?AFK19291.1) | 4 | 4 | 45.56 | [12.4](http://smserver.sti.ua.es/millbin/msdigest.cgi?missed_cleavages=2&msparams_dir=msparams_mill/&hide_protein_sequence=2&database=PA_haloferax_mediterranei_genbank&seqdb_dir=D:\SeqDB\&enzyme=Trypsin&access_method=Accession+Number&accession_num=AFK19291.1&coverage_map=0+9+9+35+23+96+11+25+13+228) | 2.45e+006 |
| ABC-type branched-chain amino acid transport systems, substrate-binding protein | [AFK20482.1](http://smserver.sti.ua.es/millhtml/SM_instruct/servadmn.htm#update_acclinks?AFK20482.1) | 3 | 3 | 45.02 | [13.4](http://smserver.sti.ua.es/millbin/msdigest.cgi?missed_cleavages=2&msparams_dir=msparams_mill/&hide_protein_sequence=2&database=PA_haloferax_mediterranei_genbank&seqdb_dir=D:\SeqDB\&enzyme=Trypsin&access_method=Accession+Number&accession_num=AFK20482.1&coverage_map=0+57+19+167+30+86+10+70) | 2.02e+006 |
| Urocanate hydratase (plasmid) | [AFK21450.1](http://smserver.sti.ua.es/millhtml/SM_instruct/servadmn.htm#update_acclinks?AFK21450.1) | 3 | 3 | 44.92 | [7.5](http://smserver.sti.ua.es/millbin/msdigest.cgi?missed_cleavages=2&msparams_dir=msparams_mill/&hide_protein_sequence=2&database=PA_haloferax_mediterranei_genbank&seqdb_dir=D:\SeqDB\&enzyme=Trypsin&access_method=Accession+Number&accession_num=AFK21450.1&coverage_map=0+376+13+56+18+77+13+32) | 1.44e+006 |
| Oligopeptide ABC transporter ATPase component | [AFK17803.1](http://smserver.sti.ua.es/millhtml/SM_instruct/servadmn.htm#update_acclinks?AFK17803.1) | 3 | 3 | 44.53 | [9.4](http://smserver.sti.ua.es/millbin/msdigest.cgi?missed_cleavages=2&msparams_dir=msparams_mill/&hide_protein_sequence=2&database=PA_haloferax_mediterranei_genbank&seqdb_dir=D:\SeqDB\&enzyme=Trypsin&access_method=Accession+Number&accession_num=AFK17803.1&coverage_map=0+29+11+19+11+5+11+264) | 1.36e+006 |
| Hypothetical protein HFX_1141 | [AFK18857.1](http://smserver.sti.ua.es/millhtml/SM_instruct/servadmn.htm#update_acclinks?AFK18857.1) | 5 | 3 | 44.53 | [37.6](http://smserver.sti.ua.es/millbin/msdigest.cgi?missed_cleavages=2&msparams_dir=msparams_mill/&hide_protein_sequence=2&database=PA_haloferax_mediterranei_genbank&seqdb_dir=D:\SeqDB\&enzyme=Trypsin&access_method=Accession+Number&accession_num=AFK18857.1&coverage_map=0+14+18+17+20+32) | 1.97e+007 |
| Hsp20-type chaperone | [AFK18151.1](http://smserver.sti.ua.es/millhtml/SM_instruct/servadmn.htm#update_acclinks?AFK18151.1) | 3 | 3 | 44.52 | [25.1](http://smserver.sti.ua.es/millbin/msdigest.cgi?missed_cleavages=2&msparams_dir=msparams_mill/&hide_protein_sequence=2&database=PA_haloferax_mediterranei_genbank&seqdb_dir=D:\SeqDB\&enzyme=Trypsin&access_method=Accession+Number&accession_num=AFK18151.1&coverage_map=0+3+12+3+9+95+13) | 6.39e+006 |
| Sialidase-1 | [AFK20474.1](http://smserver.sti.ua.es/millhtml/SM_instruct/servadmn.htm#update_acclinks?AFK20474.1) | 3 | 3 | 44.47 | [6.6](http://smserver.sti.ua.es/millbin/msdigest.cgi?missed_cleavages=2&msparams_dir=msparams_mill/&hide_protein_sequence=2&database=PA_haloferax_mediterranei_genbank&seqdb_dir=D:\SeqDB\&enzyme=Trypsin&access_method=Accession+Number&accession_num=AFK20474.1&coverage_map=0+166+12+193+9+40+15+106) | 2.21e+006 |
| Hypothetical protein HFX_0293 | [AFK18032.2](http://smserver.sti.ua.es/millhtml/SM_instruct/servadmn.htm#update_acclinks?AFK18032.2) | 3 | 3 | 44.28 | [10.8](http://smserver.sti.ua.es/millbin/msdigest.cgi?missed_cleavages=2&msparams_dir=msparams_mill/&hide_protein_sequence=2&database=PA_haloferax_mediterranei_genbank&seqdb_dir=D:\SeqDB\&enzyme=Trypsin&access_method=Accession+Number&accession_num=AFK18032.2&coverage_map=0+52+31+37+9+14+8+291) | 1.43e+006 |
| Serine hydroxymethyltransferase | [AFK20524.1](http://smserver.sti.ua.es/millhtml/SM_instruct/servadmn.htm#update_acclinks?AFK20524.1) | 3 | 3 | 44.17 | [7.9](http://smserver.sti.ua.es/millbin/msdigest.cgi?missed_cleavages=2&msparams_dir=msparams_mill/&hide_protein_sequence=2&database=PA_haloferax_mediterranei_genbank&seqdb_dir=D:\SeqDB\&enzyme=Trypsin&access_method=Accession+Number&accession_num=AFK20524.1&coverage_map=0+7+16+329+9+17+8+29) | 2.22e+006 |
| Putative monovalent cation/H+ antiporter subunit E | [AFK18782.1](http://smserver.sti.ua.es/millhtml/SM_instruct/servadmn.htm#update_acclinks?AFK18782.1) | 3 | 3 | 44.04 | [25.7](http://smserver.sti.ua.es/millbin/msdigest.cgi?missed_cleavages=2&msparams_dir=msparams_mill/&hide_protein_sequence=2&database=PA_haloferax_mediterranei_genbank&seqdb_dir=D:\SeqDB\&enzyme=Trypsin&access_method=Accession+Number&accession_num=AFK18782.1&coverage_map=0+54+33+1+18+92) | 1.08e+006 |
| Short-chain dehydrogenase / reductase SDR / glucose 1-dehydrogenase | [AFK20158.1](http://smserver.sti.ua.es/millhtml/SM_instruct/servadmn.htm#update_acclinks?AFK20158.1) | 3 | 3 | 43.63 | [17.4](http://smserver.sti.ua.es/millbin/msdigest.cgi?missed_cleavages=2&msparams_dir=msparams_mill/&hide_protein_sequence=2&database=PA_haloferax_mediterranei_genbank&seqdb_dir=D:\SeqDB\&enzyme=Trypsin&access_method=Accession+Number&accession_num=AFK20158.1&coverage_map=0+71+11+80+30+43) | 8.78e+005 |
| Carbonic anhydrase | [AFK18667.1](http://smserver.sti.ua.es/millhtml/SM_instruct/servadmn.htm#update_acclinks?AFK18667.1) | 3 | 3 | 43.24 | [14.7](http://smserver.sti.ua.es/millbin/msdigest.cgi?missed_cleavages=2&msparams_dir=msparams_mill/&hide_protein_sequence=2&database=PA_haloferax_mediterranei_genbank&seqdb_dir=D:\SeqDB\&enzyme=Trypsin&access_method=Accession+Number&accession_num=AFK18667.1&coverage_map=0+132+34+65) | 3.54e+006 |
| Putative acetyltransferase (plasmid) | [AFK21133.1](http://smserver.sti.ua.es/millhtml/SM_instruct/servadmn.htm#update_acclinks?AFK21133.1) | 3 | 3 | 43.18 | [23.5](http://smserver.sti.ua.es/millbin/msdigest.cgi?missed_cleavages=2&msparams_dir=msparams_mill/&hide_protein_sequence=2&database=PA_haloferax_mediterranei_genbank&seqdb_dir=D:\SeqDB\&enzyme=Trypsin&access_method=Accession+Number&accession_num=AFK21133.1&coverage_map=0+118+40+12) | 1.51e+006 |
| Cation efflux protein | [AFK17990.1](http://smserver.sti.ua.es/millhtml/SM_instruct/servadmn.htm#update_acclinks?AFK17990.1) | 3 | 3 | 43.14 | [10.8](http://smserver.sti.ua.es/millbin/msdigest.cgi?missed_cleavages=2&msparams_dir=msparams_mill/&hide_protein_sequence=2&database=PA_haloferax_mediterranei_genbank&seqdb_dir=D:\SeqDB\&enzyme=Trypsin&access_method=Accession+Number&accession_num=AFK17990.1&coverage_map=0+64+8+157+16+59+10) | 2.78e+006 |
| RecJ like exonuclease | [AFK18734.2](http://smserver.sti.ua.es/millhtml/SM_instruct/servadmn.htm#update_acclinks?AFK18734.2) | 3 | 3 | 43.00 | [4.8](http://smserver.sti.ua.es/millbin/msdigest.cgi?missed_cleavages=2&msparams_dir=msparams_mill/&hide_protein_sequence=2&database=PA_haloferax_mediterranei_genbank&seqdb_dir=D:\SeqDB\&enzyme=Trypsin&access_method=Accession+Number&accession_num=AFK18734.2&coverage_map=0+279+13+20+9+161+9+142) | 1.17e+006 |
| Hypothetical protein HFX_1679 | [AFK19385.1](http://smserver.sti.ua.es/millhtml/SM_instruct/servadmn.htm#update_acclinks?AFK19385.1) | 3 | 3 | 41.95 | [7.9](http://smserver.sti.ua.es/millbin/msdigest.cgi?missed_cleavages=2&msparams_dir=msparams_mill/&hide_protein_sequence=2&database=PA_haloferax_mediterranei_genbank&seqdb_dir=D:\SeqDB\&enzyme=Trypsin&access_method=Accession+Number&accession_num=AFK19385.1&coverage_map=0+64+12+110+19+165+9+125) | 1.89e+006 |
| ABC-type dipeptide/oligopeptide/nickel transport system, substrate-binding protein | [AFK19559.1](http://smserver.sti.ua.es/millhtml/SM_instruct/servadmn.htm#update_acclinks?AFK19559.1) | 3 | 3 | 41.82 | [6.3](http://smserver.sti.ua.es/millbin/msdigest.cgi?missed_cleavages=2&msparams_dir=msparams_mill/&hide_protein_sequence=2&database=PA_haloferax_mediterranei_genbank&seqdb_dir=D:\SeqDB\&enzyme=Trypsin&access_method=Accession+Number&accession_num=AFK19559.1&coverage_map=0+388+14+95+15+9+12+115) | 1.64e+006 |
| Methionine aminopeptidase | [AFK20296.1](http://smserver.sti.ua.es/millhtml/SM_instruct/servadmn.htm#update_acclinks?AFK20296.1) | 3 | 3 | 41.67 | [8.4](http://smserver.sti.ua.es/millbin/msdigest.cgi?missed_cleavages=2&msparams_dir=msparams_mill/&hide_protein_sequence=2&database=PA_haloferax_mediterranei_genbank&seqdb_dir=D:\SeqDB\&enzyme=Trypsin&access_method=Accession+Number&accession_num=AFK20296.1&coverage_map=0+136+10+109+15+26) | 3.94e+006 |
| Circadian regulator | [AFK18935.1](http://smserver.sti.ua.es/millhtml/SM_instruct/servadmn.htm#update_acclinks?AFK18935.1) | 3 | 3 | 41.66 | [14.6](http://smserver.sti.ua.es/millbin/msdigest.cgi?missed_cleavages=2&msparams_dir=msparams_mill/&hide_protein_sequence=2&database=PA_haloferax_mediterranei_genbank&seqdb_dir=D:\SeqDB\&enzyme=Trypsin&access_method=Accession+Number&accession_num=AFK18935.1&coverage_map=0+22+14+62+22+126) | 1.29e+006 |
| Biotin carboxylase | [AFK20173.1](http://smserver.sti.ua.es/millhtml/SM_instruct/servadmn.htm#update_acclinks?AFK20173.1) | 3 | 3 | 41.32 | [5.4](http://smserver.sti.ua.es/millbin/msdigest.cgi?missed_cleavages=2&msparams_dir=msparams_mill/&hide_protein_sequence=2&database=PA_haloferax_mediterranei_genbank&seqdb_dir=D:\SeqDB\&enzyme=Trypsin&access_method=Accession+Number&accession_num=AFK20173.1&coverage_map=0+145+13+246+11+7+9+170) | 2.71e+006 |
| Halocyanin precursor-like protein | [AFK18536.1](http://smserver.sti.ua.es/millhtml/SM_instruct/servadmn.htm#update_acclinks?AFK18536.1) | 2 | 2 | 41.30 | [15.8](http://smserver.sti.ua.es/millbin/msdigest.cgi?missed_cleavages=2&msparams_dir=msparams_mill/&hide_protein_sequence=2&database=PA_haloferax_mediterranei_genbank&seqdb_dir=D:\SeqDB\&enzyme=Trypsin&access_method=Accession+Number&accession_num=AFK18536.1&coverage_map=0+101+34+80) | 4.16e+006 |
| Halocyanin precursor-like protein | [AFK18518.1](http://smserver.sti.ua.es/millhtml/SM_instruct/servadmn.htm#update_acclinks?AFK18518.1) | 2 | 2 | 41.30 | [16.5](http://smserver.sti.ua.es/millbin/msdigest.cgi?missed_cleavages=2&msparams_dir=msparams_mill/&hide_protein_sequence=2&database=PA_haloferax_mediterranei_genbank&seqdb_dir=D:\SeqDB\&enzyme=Trypsin&access_method=Accession+Number&accession_num=AFK18518.1&coverage_map=0+98+34+73) | 4.16e+006 |
| Hypothetical protein HFX_2662 | [AFK20340.1](http://smserver.sti.ua.es/millhtml/SM_instruct/servadmn.htm#update_acclinks?AFK20340.1) | 3 | 3 | 41.19 | [14.6](http://smserver.sti.ua.es/millbin/msdigest.cgi?missed_cleavages=2&msparams_dir=msparams_mill/&hide_protein_sequence=2&database=PA_haloferax_mediterranei_genbank&seqdb_dir=D:\SeqDB\&enzyme=Trypsin&access_method=Accession+Number&accession_num=AFK20340.1&coverage_map=0+132+15+38+8+83+22+10) | 1.24e+006 |
| Hypothetical protein HFX_0588 | [AFK18313.1](http://smserver.sti.ua.es/millhtml/SM_instruct/servadmn.htm#update_acclinks?AFK18313.1) | 3 | 3 | 41.18 | [11.3](http://smserver.sti.ua.es/millbin/msdigest.cgi?missed_cleavages=2&msparams_dir=msparams_mill/&hide_protein_sequence=2&database=PA_haloferax_mediterranei_genbank&seqdb_dir=D:\SeqDB\&enzyme=Trypsin&access_method=Accession+Number&accession_num=AFK18313.1&coverage_map=0+148+14+17+13+57+12+83) | 1.49e+006 |
| Molybdopterin oxidoreductase | [AFK19900.1](http://smserver.sti.ua.es/millhtml/SM_instruct/servadmn.htm#update_acclinks?AFK19900.1) | 3 | 3 | 41.16 | [8.4](http://smserver.sti.ua.es/millbin/msdigest.cgi?missed_cleavages=2&msparams_dir=msparams_mill/&hide_protein_sequence=2&database=PA_haloferax_mediterranei_genbank&seqdb_dir=D:\SeqDB\&enzyme=Trypsin&access_method=Accession+Number&accession_num=AFK19900.1&coverage_map=0+119+11+35+12+35+15+222) | 1.83e+006 |
| Putative acylaminoacyl-peptidase | [AFK18673.1](http://smserver.sti.ua.es/millhtml/SM_instruct/servadmn.htm#update_acclinks?AFK18673.1) | 3 | 3 | 41.15 | [3.8](http://smserver.sti.ua.es/millbin/msdigest.cgi?missed_cleavages=2&msparams_dir=msparams_mill/&hide_protein_sequence=2&database=PA_haloferax_mediterranei_genbank&seqdb_dir=D:\SeqDB\&enzyme=Trypsin&access_method=Accession+Number&accession_num=AFK18673.1&coverage_map=0+77+7+286+10+311+10+9) | 6.18e+005 |
| ABC-type dipeptide/oligopeptide/nickel transport system, substrate-binding protein (plasmid) | [AFK21027.1](http://smserver.sti.ua.es/millhtml/SM_instruct/servadmn.htm#update_acclinks?AFK21027.1) | 4 | 3 | 41.09 | [5.9](http://smserver.sti.ua.es/millbin/msdigest.cgi?missed_cleavages=2&msparams_dir=msparams_mill/&hide_protein_sequence=2&database=PA_haloferax_mediterranei_genbank&seqdb_dir=D:\SeqDB\&enzyme=Trypsin&access_method=Accession+Number&accession_num=AFK21027.1&coverage_map=0+116+7+379+26+28) | 5.34e+006 |
| Cytochrome b/b6 (plasmid) | [AFK20941.1](http://smserver.sti.ua.es/millhtml/SM_instruct/servadmn.htm#update_acclinks?AFK20941.1) | 3 | 3 | 40.50 | [10.9](http://smserver.sti.ua.es/millbin/msdigest.cgi?missed_cleavages=2&msparams_dir=msparams_mill/&hide_protein_sequence=2&database=PA_haloferax_mediterranei_genbank&seqdb_dir=D:\SeqDB\&enzyme=Trypsin&access_method=Accession+Number&accession_num=AFK20941.1&coverage_map=0+392+11+28+42+12) | 9.75e+006 |
| Hypothetical protein HFX_0697 | [AFK18420.1](http://smserver.sti.ua.es/millhtml/SM_instruct/servadmn.htm#update_acclinks?AFK18420.1) | 4 | 2 | 40.36 | [38.7](http://smserver.sti.ua.es/millbin/msdigest.cgi?missed_cleavages=2&msparams_dir=msparams_mill/&hide_protein_sequence=2&database=PA_haloferax_mediterranei_genbank&seqdb_dir=D:\SeqDB\&enzyme=Trypsin&access_method=Accession+Number&accession_num=AFK18420.1&coverage_map=0+51+36+6) | 2.47e+007 |
| Hypothetical protein HFX_5167 (plasmid) | [AFK21001.1](http://smserver.sti.ua.es/millhtml/SM_instruct/servadmn.htm#update_acclinks?AFK21001.1) | 4 | 3 | 40.26 | [19.2](http://smserver.sti.ua.es/millbin/msdigest.cgi?missed_cleavages=2&msparams_dir=msparams_mill/&hide_protein_sequence=2&database=PA_haloferax_mediterranei_genbank&seqdb_dir=D:\SeqDB\&enzyme=Trypsin&access_method=Accession+Number&accession_num=AFK21001.1&coverage_map=0+83+13+44+40+95) | 3.09e+006 |
| Dipeptidyl aminopeptidase/acylaminoacyl peptidase | [AFK18522.1](http://smserver.sti.ua.es/millhtml/SM_instruct/servadmn.htm#update_acclinks?AFK18522.1) | 3 | 3 | 40.01 | [5.4](http://smserver.sti.ua.es/millbin/msdigest.cgi?missed_cleavages=2&msparams_dir=msparams_mill/&hide_protein_sequence=2&database=PA_haloferax_mediterranei_genbank&seqdb_dir=D:\SeqDB\&enzyme=Trypsin&access_method=Accession+Number&accession_num=AFK18522.1&coverage_map=0+219+9+177+10+133+14+44) | 1.18e+006 |
| Lysophospholipase | [AFK20242.1](http://smserver.sti.ua.es/millhtml/SM_instruct/servadmn.htm#update_acclinks?AFK20242.1) | 3 | 3 | 40.01 | [13](http://smserver.sti.ua.es/millbin/msdigest.cgi?missed_cleavages=2&msparams_dir=msparams_mill/&hide_protein_sequence=2&database=PA_haloferax_mediterranei_genbank&seqdb_dir=D:\SeqDB\&enzyme=Trypsin&access_method=Accession+Number&accession_num=AFK20242.1&coverage_map=0+98+20+31+14+71+7+72) | 9.96e+005 |
| Hypothetical protein HFX_0457 | [AFK18192.2](http://smserver.sti.ua.es/millhtml/SM_instruct/servadmn.htm#update_acclinks?AFK18192.2) | 3 | 3 | 39.92 | [20.9](http://smserver.sti.ua.es/millbin/msdigest.cgi?missed_cleavages=2&msparams_dir=msparams_mill/&hide_protein_sequence=2&database=PA_haloferax_mediterranei_genbank&seqdb_dir=D:\SeqDB\&enzyme=Trypsin&access_method=Accession+Number&accession_num=AFK18192.2&coverage_map=0+73+32+48) | 1.54e+006 |
| Zn-dependent hydrolase, glyoxylase | [AFK19254.1](http://smserver.sti.ua.es/millhtml/SM_instruct/servadmn.htm#update_acclinks?AFK19254.1) | 3 | 3 | 39.59 | [16.1](http://smserver.sti.ua.es/millbin/msdigest.cgi?missed_cleavages=2&msparams_dir=msparams_mill/&hide_protein_sequence=2&database=PA_haloferax_mediterranei_genbank&seqdb_dir=D:\SeqDB\&enzyme=Trypsin&access_method=Accession+Number&accession_num=AFK19254.1&coverage_map=0+64+14+104+19+43+28+106) | 5.31e+005 |
| Glutamate/aspartate transport protein | [AFK19350.1](http://smserver.sti.ua.es/millhtml/SM_instruct/servadmn.htm#update_acclinks?AFK19350.1) | 3 | 3 | 39.48 | [6.2](http://smserver.sti.ua.es/millbin/msdigest.cgi?missed_cleavages=2&msparams_dir=msparams_mill/&hide_protein_sequence=2&database=PA_haloferax_mediterranei_genbank&seqdb_dir=D:\SeqDB\&enzyme=Trypsin&access_method=Accession+Number&accession_num=AFK19350.1&coverage_map=0+14+7+239+20+155) | 1.67e+006 |
| Riboflavin synthase beta subunit (6,7-dimethyl-8-ribityllumazine synthase) | [AFK18690.1](http://smserver.sti.ua.es/millhtml/SM_instruct/servadmn.htm#update_acclinks?AFK18690.1) | 3 | 3 | 39.26 | [36.5](http://smserver.sti.ua.es/millbin/msdigest.cgi?missed_cleavages=2&msparams_dir=msparams_mill/&hide_protein_sequence=2&database=PA_haloferax_mediterranei_genbank&seqdb_dir=D:\SeqDB\&enzyme=Trypsin&access_method=Accession+Number&accession_num=AFK18690.1&coverage_map=0+10+10+35+39+40) | 7.25e+005 |
| Glutamate-1-semialdehyde aminotransferase | [AFK17832.1](http://smserver.sti.ua.es/millhtml/SM_instruct/servadmn.htm#update_acclinks?AFK17832.1) | 3 | 3 | 39.03 | [9.4](http://smserver.sti.ua.es/millbin/msdigest.cgi?missed_cleavages=2&msparams_dir=msparams_mill/&hide_protein_sequence=2&database=PA_haloferax_mediterranei_genbank&seqdb_dir=D:\SeqDB\&enzyme=Trypsin&access_method=Accession+Number&accession_num=AFK17832.1&coverage_map=0+12+14+84+14+139+14+168) | 1.07e+006 |
| Methylmalonyl-CoA decarboxylase alpha chain | [AFK20161.1](http://smserver.sti.ua.es/millhtml/SM_instruct/servadmn.htm#update_acclinks?AFK20161.1) | 3 | 3 | 38.69 | [7.9](http://smserver.sti.ua.es/millbin/msdigest.cgi?missed_cleavages=2&msparams_dir=msparams_mill/&hide_protein_sequence=2&database=PA_haloferax_mediterranei_genbank&seqdb_dir=D:\SeqDB\&enzyme=Trypsin&access_method=Accession+Number&accession_num=AFK20161.1&coverage_map=0+266+14+35+14+147+13+27) | 1.60e+006 |
| Hypothetical protein HFX_6351 (plasmid) | [AFK21472.1](http://smserver.sti.ua.es/millhtml/SM_instruct/servadmn.htm#update_acclinks?AFK21472.1) | 3 | 3 | 38.08 | [26.4](http://smserver.sti.ua.es/millbin/msdigest.cgi?missed_cleavages=2&msparams_dir=msparams_mill/&hide_protein_sequence=2&database=PA_haloferax_mediterranei_genbank&seqdb_dir=D:\SeqDB\&enzyme=Trypsin&access_method=Accession+Number&accession_num=AFK21472.1&coverage_map=0+3+19+46+9+29) | 1.64e+006 |
| Putative patatin-like phospholipase (plasmid) | [AFK21580.1](http://smserver.sti.ua.es/millhtml/SM_instruct/servadmn.htm#update_acclinks?AFK21580.1) | 3 | 3 | 38.06 | [13.7](http://smserver.sti.ua.es/millbin/msdigest.cgi?missed_cleavages=2&msparams_dir=msparams_mill/&hide_protein_sequence=2&database=PA_haloferax_mediterranei_genbank&seqdb_dir=D:\SeqDB\&enzyme=Trypsin&access_method=Accession+Number&accession_num=AFK21580.1&coverage_map=0+214+15+14+14+41+15+8) | 2.64e+006 |
| Sugar ABC transporter ATP-binding protein (UGPC) | [AFK20563.1](http://smserver.sti.ua.es/millhtml/SM_instruct/servadmn.htm#update_acclinks?AFK20563.1) | 3 | 2 | 37.96 | [10.2](http://smserver.sti.ua.es/millbin/msdigest.cgi?missed_cleavages=2&msparams_dir=msparams_mill/&hide_protein_sequence=2&database=PA_haloferax_mediterranei_genbank&seqdb_dir=D:\SeqDB\&enzyme=Trypsin&access_method=Accession+Number&accession_num=AFK20563.1&coverage_map=0+51+14+143+25+146) | 2.18e+006 |
| Hypothetical protein HFX_0936 | [AFK18655.1](http://smserver.sti.ua.es/millhtml/SM_instruct/servadmn.htm#update_acclinks?AFK18655.1) | 2 | 2 | 37.90 | [27.2](http://smserver.sti.ua.es/millbin/msdigest.cgi?missed_cleavages=2&msparams_dir=msparams_mill/&hide_protein_sequence=2&database=PA_haloferax_mediterranei_genbank&seqdb_dir=D:\SeqDB\&enzyme=Trypsin&access_method=Accession+Number&accession_num=AFK18655.1&coverage_map=0+20+16+83+24+4) | 6.09e+005 |
| Cytochrome c oxidase subunit II / ba3-type terminal oxidase subunit II | [AFK18662.1](http://smserver.sti.ua.es/millhtml/SM_instruct/servadmn.htm#update_acclinks?AFK18662.1) | 2 | 2 | 37.86 | [14.2](http://smserver.sti.ua.es/millbin/msdigest.cgi?missed_cleavages=2&msparams_dir=msparams_mill/&hide_protein_sequence=2&database=PA_haloferax_mediterranei_genbank&seqdb_dir=D:\SeqDB\&enzyme=Trypsin&access_method=Accession+Number&accession_num=AFK18662.1&coverage_map=0+62+26+94) | 1.85e+007 |
| Hypothetical protein HFX_1268 | [AFK18981.1](http://smserver.sti.ua.es/millhtml/SM_instruct/servadmn.htm#update_acclinks?AFK18981.1) | 3 | 3 | 37.66 | [4.1](http://smserver.sti.ua.es/millbin/msdigest.cgi?missed_cleavages=2&msparams_dir=msparams_mill/&hide_protein_sequence=2&database=PA_haloferax_mediterranei_genbank&seqdb_dir=D:\SeqDB\&enzyme=Trypsin&access_method=Accession+Number&accession_num=AFK18981.1&coverage_map=0+164+10+14+9+233+14+356) | 9.24e+005 |
| Acetyl transferase | [AFK20422.1](http://smserver.sti.ua.es/millhtml/SM_instruct/servadmn.htm#update_acclinks?AFK20422.1) | 2 | 2 | 37.45 | [16.8](http://smserver.sti.ua.es/millbin/msdigest.cgi?missed_cleavages=2&msparams_dir=msparams_mill/&hide_protein_sequence=2&database=PA_haloferax_mediterranei_genbank&seqdb_dir=D:\SeqDB\&enzyme=Trypsin&access_method=Accession+Number&accession_num=AFK20422.1&coverage_map=0+85+41+118) | 3.54e+006 |
| Aconitate hydratase | [AFK19741.1](http://smserver.sti.ua.es/millhtml/SM_instruct/servadmn.htm#update_acclinks?AFK19741.1) | 3 | 3 | 37.10 | [6.5](http://smserver.sti.ua.es/millbin/msdigest.cgi?missed_cleavages=2&msparams_dir=msparams_mill/&hide_protein_sequence=2&database=PA_haloferax_mediterranei_genbank&seqdb_dir=D:\SeqDB\&enzyme=Trypsin&access_method=Accession+Number&accession_num=AFK19741.1&coverage_map=0+437+16+24+19+7+8+144) | 1.05e+006 |
| Hypothetical protein HFX_2772 | [AFK20448.1](http://smserver.sti.ua.es/millhtml/SM_instruct/servadmn.htm#update_acclinks?AFK20448.1) | 4 | 2 | 36.82 | [20.5](http://smserver.sti.ua.es/millbin/msdigest.cgi?missed_cleavages=2&msparams_dir=msparams_mill/&hide_protein_sequence=2&database=PA_haloferax_mediterranei_genbank&seqdb_dir=D:\SeqDB\&enzyme=Trypsin&access_method=Accession+Number&accession_num=AFK20448.1&coverage_map=0+81+21) | 1.37e+007 |
| Hypothetical protein HFX_2513 | [AFK20195.1](http://smserver.sti.ua.es/millhtml/SM_instruct/servadmn.htm#update_acclinks?AFK20195.1) | 3 | 3 | 36.73 | [19](http://smserver.sti.ua.es/millbin/msdigest.cgi?missed_cleavages=2&msparams_dir=msparams_mill/&hide_protein_sequence=2&database=PA_haloferax_mediterranei_genbank&seqdb_dir=D:\SeqDB\&enzyme=Trypsin&access_method=Accession+Number&accession_num=AFK20195.1&coverage_map=0+70+10+25+15+11) | 1.69e+006 |
| PLP-dependent aminotransferase (aspartate aminotransferase) | [AFK19365.1](http://smserver.sti.ua.es/millhtml/SM_instruct/servadmn.htm#update_acclinks?AFK19365.1) | 3 | 3 | 36.72 | [9.9](http://smserver.sti.ua.es/millbin/msdigest.cgi?missed_cleavages=2&msparams_dir=msparams_mill/&hide_protein_sequence=2&database=PA_haloferax_mediterranei_genbank&seqdb_dir=D:\SeqDB\&enzyme=Trypsin&access_method=Accession+Number&accession_num=AFK19365.1&coverage_map=0+7+10+219+9+46+18+64) | 9.76e+005 |
| Hypothetical protein HFX_6273 (plasmid) | [AFK21396.2](http://smserver.sti.ua.es/millhtml/SM_instruct/servadmn.htm#update_acclinks?AFK21396.2) | 3 | 3 | 36.30 | [8.7](http://smserver.sti.ua.es/millbin/msdigest.cgi?missed_cleavages=2&msparams_dir=msparams_mill/&hide_protein_sequence=2&database=PA_haloferax_mediterranei_genbank&seqdb_dir=D:\SeqDB\&enzyme=Trypsin&access_method=Accession+Number&accession_num=AFK21396.2&coverage_map=0+167+11+17+7+56+13+85) | 1.11e+006 |
| 3-ketoacyl-acyl carrier protein reductase (PhaB) (plasmid) | [AFK21048.1](http://smserver.sti.ua.es/millhtml/SM_instruct/servadmn.htm#update_acclinks?AFK21048.1) | 3 | 3 | 36.05 | [12](http://smserver.sti.ua.es/millbin/msdigest.cgi?missed_cleavages=2&msparams_dir=msparams_mill/&hide_protein_sequence=2&database=PA_haloferax_mediterranei_genbank&seqdb_dir=D:\SeqDB\&enzyme=Trypsin&access_method=Accession+Number&accession_num=AFK21048.1&coverage_map=0+110+14+36+16+72) | 8.54e+006 |
| Gas-vesicle operon protein gvpF | [AFK19405.1](http://smserver.sti.ua.es/millhtml/SM_instruct/servadmn.htm#update_acclinks?AFK19405.1) | 2 | 2 | 36.04 | [15](http://smserver.sti.ua.es/millbin/msdigest.cgi?missed_cleavages=2&msparams_dir=msparams_mill/&hide_protein_sequence=2&database=PA_haloferax_mediterranei_genbank&seqdb_dir=D:\SeqDB\&enzyme=Trypsin&access_method=Accession+Number&accession_num=AFK19405.1&coverage_map=0+102+15+21+17+58) | 6.30e+005 |
| Phosphoribosylaminoimidazole synthetase | [AFK19325.1](http://smserver.sti.ua.es/millhtml/SM_instruct/servadmn.htm#update_acclinks?AFK19325.1) | 2 | 2 | 35.83 | [6.1](http://smserver.sti.ua.es/millbin/msdigest.cgi?missed_cleavages=2&msparams_dir=msparams_mill/&hide_protein_sequence=2&database=PA_haloferax_mediterranei_genbank&seqdb_dir=D:\SeqDB\&enzyme=Trypsin&access_method=Accession+Number&accession_num=AFK19325.1&coverage_map=0+215+10+85+10+4) | 1.63e+006 |
| Molybdopterin biosynthesis protein moeA | [AFK20001.1](http://smserver.sti.ua.es/millhtml/SM_instruct/servadmn.htm#update_acclinks?AFK20001.1) | 3 | 3 | 35.75 | [11](http://smserver.sti.ua.es/millbin/msdigest.cgi?missed_cleavages=2&msparams_dir=msparams_mill/&hide_protein_sequence=2&database=PA_haloferax_mediterranei_genbank&seqdb_dir=D:\SeqDB\&enzyme=Trypsin&access_method=Accession+Number&accession_num=AFK20001.1&coverage_map=0+45+17+25+13+22+15+269) | 8.82e+005 |
| NAD synthetase | [AFK19658.1](http://smserver.sti.ua.es/millhtml/SM_instruct/servadmn.htm#update_acclinks?AFK19658.1) | 3 | 3 | 35.74 | [10.1](http://smserver.sti.ua.es/millbin/msdigest.cgi?missed_cleavages=2&msparams_dir=msparams_mill/&hide_protein_sequence=2&database=PA_haloferax_mediterranei_genbank&seqdb_dir=D:\SeqDB\&enzyme=Trypsin&access_method=Accession+Number&accession_num=AFK19658.1&coverage_map=0+155+10+77+18+16) | 4.85e+006 |
| Carboxypeptidase | [AFK18120.1](http://smserver.sti.ua.es/millhtml/SM_instruct/servadmn.htm#update_acclinks?AFK18120.1) | 3 | 3 | 35.64 | [8.7](http://smserver.sti.ua.es/millbin/msdigest.cgi?missed_cleavages=2&msparams_dir=msparams_mill/&hide_protein_sequence=2&database=PA_haloferax_mediterranei_genbank&seqdb_dir=D:\SeqDB\&enzyme=Trypsin&access_method=Accession+Number&accession_num=AFK18120.1&coverage_map=0+124+14+79+12+248+18+7) | 1.27e+006 |
| Poly(3-hydroxyalkanoate) synthase subunit PhaC | [AFK20356.1](http://smserver.sti.ua.es/millhtml/SM_instruct/servadmn.htm#update_acclinks?AFK20356.1) | 3 | 3 | 35.63 | [9.7](http://smserver.sti.ua.es/millbin/msdigest.cgi?missed_cleavages=2&msparams_dir=msparams_mill/&hide_protein_sequence=2&database=PA_haloferax_mediterranei_genbank&seqdb_dir=D:\SeqDB\&enzyme=Trypsin&access_method=Accession+Number&accession_num=AFK20356.1&coverage_map=0+115+13+273+21+7+9+4) | 1.40e+006 |
| Acyl-CoA dehydrogenase | [AFK18868.1](http://smserver.sti.ua.es/millhtml/SM_instruct/servadmn.htm#update_acclinks?AFK18868.1) | 3 | 3 | 35.47 | [9.4](http://smserver.sti.ua.es/millbin/msdigest.cgi?missed_cleavages=2&msparams_dir=msparams_mill/&hide_protein_sequence=2&database=PA_haloferax_mediterranei_genbank&seqdb_dir=D:\SeqDB\&enzyme=Trypsin&access_method=Accession+Number&accession_num=AFK18868.1&coverage_map=0+13+14+242+14+38+8+51) | 8.14e+005 |
| Amino acid-binding protein (plasmid) | [AFK21485.1](http://smserver.sti.ua.es/millhtml/SM_instruct/servadmn.htm#update_acclinks?AFK21485.1) | 2 | 2 | 35.02 | [6.9](http://smserver.sti.ua.es/millbin/msdigest.cgi?missed_cleavages=2&msparams_dir=msparams_mill/&hide_protein_sequence=2&database=PA_haloferax_mediterranei_genbank&seqdb_dir=D:\SeqDB\&enzyme=Trypsin&access_method=Accession+Number&accession_num=AFK21485.1&coverage_map=0+282+15+41+14+67) | 4.86e+005 |
| Peptidyl-prolyl cis-trans isomerase B (cyclophilin B) | [AFK19961.1](http://smserver.sti.ua.es/millhtml/SM_instruct/servadmn.htm#update_acclinks?AFK19961.1) | 4 | 2 | 34.67 | [16.2](http://smserver.sti.ua.es/millbin/msdigest.cgi?missed_cleavages=2&msparams_dir=msparams_mill/&hide_protein_sequence=2&database=PA_haloferax_mediterranei_genbank&seqdb_dir=D:\SeqDB\&enzyme=Trypsin&access_method=Accession+Number&accession_num=AFK19961.1&coverage_map=0+13+11+40+17+91) | 8.27e+006 |
| Acyl-CoA synthetase | [AFK18955.2](http://smserver.sti.ua.es/millhtml/SM_instruct/servadmn.htm#update_acclinks?AFK18955.2) | 3 | 3 | 34.45 | [5.4](http://smserver.sti.ua.es/millbin/msdigest.cgi?missed_cleavages=2&msparams_dir=msparams_mill/&hide_protein_sequence=2&database=PA_haloferax_mediterranei_genbank&seqdb_dir=D:\SeqDB\&enzyme=Trypsin&access_method=Accession+Number&accession_num=AFK18955.2&coverage_map=0+62+12+205+9+33+8+206) | 9.33e+005 |
| Hypothetical protein HFX_2532 | [AFK20214.1](http://smserver.sti.ua.es/millhtml/SM_instruct/servadmn.htm#update_acclinks?AFK20214.1) | 3 | 3 | 33.88 | [10.2](http://smserver.sti.ua.es/millbin/msdigest.cgi?missed_cleavages=2&msparams_dir=msparams_mill/&hide_protein_sequence=2&database=PA_haloferax_mediterranei_genbank&seqdb_dir=D:\SeqDB\&enzyme=Trypsin&access_method=Accession+Number&accession_num=AFK20214.1&coverage_map=0+19+15+84+12+46+9+167) | 1.01e+006 |
| Mechanosensitive ion channel | [AFK19459.1](http://smserver.sti.ua.es/millhtml/SM_instruct/servadmn.htm#update_acclinks?AFK19459.1) | 2 | 2 | 33.73 | [7.7](http://smserver.sti.ua.es/millbin/msdigest.cgi?missed_cleavages=2&msparams_dir=msparams_mill/&hide_protein_sequence=2&database=PA_haloferax_mediterranei_genbank&seqdb_dir=D:\SeqDB\&enzyme=Trypsin&access_method=Accession+Number&accession_num=AFK19459.1&coverage_map=0+68+14+68+8+125) | 2.40e+006 |
| Ribose ABC transporter ATP-binding protein | [AFK19184.1](http://smserver.sti.ua.es/millhtml/SM_instruct/servadmn.htm#update_acclinks?AFK19184.1) | 3 | 3 | 33.64 | [5.2](http://smserver.sti.ua.es/millbin/msdigest.cgi?missed_cleavages=2&msparams_dir=msparams_mill/&hide_protein_sequence=2&database=PA_haloferax_mediterranei_genbank&seqdb_dir=D:\SeqDB\&enzyme=Trypsin&access_method=Accession+Number&accession_num=AFK19184.1&coverage_map=0+120+9+64+8+261+10+47) | 2.33e+006 |
| Stress response protein | [AFK18130.1](http://smserver.sti.ua.es/millhtml/SM_instruct/servadmn.htm#update_acclinks?AFK18130.1) | 2 | 2 | 33.63 | [20.8](http://smserver.sti.ua.es/millbin/msdigest.cgi?missed_cleavages=2&msparams_dir=msparams_mill/&hide_protein_sequence=2&database=PA_haloferax_mediterranei_genbank&seqdb_dir=D:\SeqDB\&enzyme=Trypsin&access_method=Accession+Number&accession_num=AFK18130.1&coverage_map=0+40+31+78) | 5.42e+005 |
| ABC-type dipeptide/oligopeptide/nickel transport systems, ATP-binding protein I & II | [AFK18329.1](http://smserver.sti.ua.es/millhtml/SM_instruct/servadmn.htm#update_acclinks?AFK18329.1) | 2 | 2 | 33.59 | [2.8](http://smserver.sti.ua.es/millbin/msdigest.cgi?missed_cleavages=2&msparams_dir=msparams_mill/&hide_protein_sequence=2&database=PA_haloferax_mediterranei_genbank&seqdb_dir=D:\SeqDB\&enzyme=Trypsin&access_method=Accession+Number&accession_num=AFK18329.1&coverage_map=0+203+12+303+14+366) | 5.39e+005 |
| Preprotein translocase subunit SecF | [AFK19760.1](http://smserver.sti.ua.es/millhtml/SM_instruct/servadmn.htm#update_acclinks?AFK19760.1) | 2 | 2 | 33.31 | [11.1](http://smserver.sti.ua.es/millbin/msdigest.cgi?missed_cleavages=2&msparams_dir=msparams_mill/&hide_protein_sequence=2&database=PA_haloferax_mediterranei_genbank&seqdb_dir=D:\SeqDB\&enzyme=Trypsin&access_method=Accession+Number&accession_num=AFK19760.1&coverage_map=0+58+22+130+10+67) | 5.02e+005 |
| Arginase | [AFK19340.1](http://smserver.sti.ua.es/millhtml/SM_instruct/servadmn.htm#update_acclinks?AFK19340.1) | 2 | 2 | 33.26 | [14.3](http://smserver.sti.ua.es/millbin/msdigest.cgi?missed_cleavages=2&msparams_dir=msparams_mill/&hide_protein_sequence=2&database=PA_haloferax_mediterranei_genbank&seqdb_dir=D:\SeqDB\&enzyme=Trypsin&access_method=Accession+Number&accession_num=AFK19340.1&coverage_map=0+6+12+247+31+3) | 1.38e+007 |
| Glycerol-3-phosphate dehydrogenase subunit B | [AFK19306.1](http://smserver.sti.ua.es/millhtml/SM_instruct/servadmn.htm#update_acclinks?AFK19306.1) | 2 | 2 | 33.21 | [9](http://smserver.sti.ua.es/millbin/msdigest.cgi?missed_cleavages=2&msparams_dir=msparams_mill/&hide_protein_sequence=2&database=PA_haloferax_mediterranei_genbank&seqdb_dir=D:\SeqDB\&enzyme=Trypsin&access_method=Accession+Number&accession_num=AFK19306.1&coverage_map=0+202+38+182) | 2.10e+005 |
| NADPH-dependent FMN reductase | [AFK20556.2](http://smserver.sti.ua.es/millhtml/SM_instruct/servadmn.htm#update_acclinks?AFK20556.2) | 2 | 2 | 33.16 | [24.4](http://smserver.sti.ua.es/millbin/msdigest.cgi?missed_cleavages=2&msparams_dir=msparams_mill/&hide_protein_sequence=2&database=PA_haloferax_mediterranei_genbank&seqdb_dir=D:\SeqDB\&enzyme=Trypsin&access_method=Accession+Number&accession_num=AFK20556.2&coverage_map=0+20+23+22+24+103) | 1.52e+006 |
| Hypothetical protein HFX_6067 (plasmid) | [AFK21194.1](http://smserver.sti.ua.es/millhtml/SM_instruct/servadmn.htm#update_acclinks?AFK21194.1) | 2 | 2 | 32.86 | [6.2](http://smserver.sti.ua.es/millbin/msdigest.cgi?missed_cleavages=2&msparams_dir=msparams_mill/&hide_protein_sequence=2&database=PA_haloferax_mediterranei_genbank&seqdb_dir=D:\SeqDB\&enzyme=Trypsin&access_method=Accession+Number&accession_num=AFK21194.1&coverage_map=0+156+11+112+15+120) | 8.23e+005 |
| Universal stress protein (plasmid) | [AFK21282.1](http://smserver.sti.ua.es/millhtml/SM_instruct/servadmn.htm#update_acclinks?AFK21282.1) | 2 | 2 | 32.83 | [15.6](http://smserver.sti.ua.es/millbin/msdigest.cgi?missed_cleavages=2&msparams_dir=msparams_mill/&hide_protein_sequence=2&database=PA_haloferax_mediterranei_genbank&seqdb_dir=D:\SeqDB\&enzyme=Trypsin&access_method=Accession+Number&accession_num=AFK21282.1&coverage_map=0+8+44+229) | 1.53e+006 |
| Methylmalonyl-CoA mutase, N-terminal domain protein | [AFK18588.1](http://smserver.sti.ua.es/millhtml/SM_instruct/servadmn.htm#update_acclinks?AFK18588.1) | 2 | 2 | 32.62 | [6.3](http://smserver.sti.ua.es/millbin/msdigest.cgi?missed_cleavages=2&msparams_dir=msparams_mill/&hide_protein_sequence=2&database=PA_haloferax_mediterranei_genbank&seqdb_dir=D:\SeqDB\&enzyme=Trypsin&access_method=Accession+Number&accession_num=AFK18588.1&coverage_map=0+219+15+93+21+218) | 5.58e+005 |
| Cell division protein ftsZ | [AFK19949.2](http://smserver.sti.ua.es/millhtml/SM_instruct/servadmn.htm#update_acclinks?AFK19949.2) | 2 | 2 | 32.57 | [7.3](http://smserver.sti.ua.es/millbin/msdigest.cgi?missed_cleavages=2&msparams_dir=msparams_mill/&hide_protein_sequence=2&database=PA_haloferax_mediterranei_genbank&seqdb_dir=D:\SeqDB\&enzyme=Trypsin&access_method=Accession+Number&accession_num=AFK19949.2&coverage_map=0+294+14+31+15+41) | 7.45e+005 |
| Halocyanin hcpH | [AFK18872.1](http://smserver.sti.ua.es/millhtml/SM_instruct/servadmn.htm#update_acclinks?AFK18872.1) | 2 | 2 | 32.43 | [28.3](http://smserver.sti.ua.es/millbin/msdigest.cgi?missed_cleavages=2&msparams_dir=msparams_mill/&hide_protein_sequence=2&database=PA_haloferax_mediterranei_genbank&seqdb_dir=D:\SeqDB\&enzyme=Trypsin&access_method=Accession+Number&accession_num=AFK18872.1&coverage_map=0+71+42+35) | 3.30e+006 |
| Universal stress protein UspA-like protein | [AFK18989.1](http://smserver.sti.ua.es/millhtml/SM_instruct/servadmn.htm#update_acclinks?AFK18989.1) | 2 | 2 | 32.41 | [26.2](http://smserver.sti.ua.es/millbin/msdigest.cgi?missed_cleavages=2&msparams_dir=msparams_mill/&hide_protein_sequence=2&database=PA_haloferax_mediterranei_genbank&seqdb_dir=D:\SeqDB\&enzyme=Trypsin&access_method=Accession+Number&accession_num=AFK18989.1&coverage_map=0+43+28+45+9+16) | 6.82e+006 |
| Hypothetical protein HFX_2853 | [AFK20523.1](http://smserver.sti.ua.es/millhtml/SM_instruct/servadmn.htm#update_acclinks?AFK20523.1) | 3 | 2 | 32.40 | [16.3](http://smserver.sti.ua.es/millbin/msdigest.cgi?missed_cleavages=2&msparams_dir=msparams_mill/&hide_protein_sequence=2&database=PA_haloferax_mediterranei_genbank&seqdb_dir=D:\SeqDB\&enzyme=Trypsin&access_method=Accession+Number&accession_num=AFK20523.1&coverage_map=0+95+19+40+26+95) | 5.59e+005 |
| Oligopeptide ABC transporter permease protein | [AFK17804.1](http://smserver.sti.ua.es/millhtml/SM_instruct/servadmn.htm#update_acclinks?AFK17804.1) | 2 | 2 | 32.32 | [4.8](http://smserver.sti.ua.es/millbin/msdigest.cgi?missed_cleavages=2&msparams_dir=msparams_mill/&hide_protein_sequence=2&database=PA_haloferax_mediterranei_genbank&seqdb_dir=D:\SeqDB\&enzyme=Trypsin&access_method=Accession+Number&accession_num=AFK17804.1&coverage_map=0+221+12+11+11+224) | 1.56e+006 |
| Ubiquinone/menaquinone biosynthesis methyltransferase | [AFK18034.2](http://smserver.sti.ua.es/millhtml/SM_instruct/servadmn.htm#update_acclinks?AFK18034.2) | 2 | 2 | 32.21 | [12.9](http://smserver.sti.ua.es/millbin/msdigest.cgi?missed_cleavages=2&msparams_dir=msparams_mill/&hide_protein_sequence=2&database=PA_haloferax_mediterranei_genbank&seqdb_dir=D:\SeqDB\&enzyme=Trypsin&access_method=Accession+Number&accession_num=AFK18034.2&coverage_map=0+48+17+76+10+57) | 2.92e+006 |
| Hsp20-type chaperone (plasmid) | [AFK20982.1](http://smserver.sti.ua.es/millhtml/SM_instruct/servadmn.htm#update_acclinks?AFK20982.1) | 2 | 2 | 31.89 | [17.6](http://smserver.sti.ua.es/millbin/msdigest.cgi?missed_cleavages=2&msparams_dir=msparams_mill/&hide_protein_sequence=2&database=PA_haloferax_mediterranei_genbank&seqdb_dir=D:\SeqDB\&enzyme=Trypsin&access_method=Accession+Number&accession_num=AFK20982.1&coverage_map=0+3+9+54+17+64) | 5.58e+005 |
| Hypothetical protein HFX_1448 | [AFK19156.1](http://smserver.sti.ua.es/millhtml/SM_instruct/servadmn.htm#update_acclinks?AFK19156.1) | 3 | 2 | 31.81 | [12.7](http://smserver.sti.ua.es/millbin/msdigest.cgi?missed_cleavages=2&msparams_dir=msparams_mill/&hide_protein_sequence=2&database=PA_haloferax_mediterranei_genbank&seqdb_dir=D:\SeqDB\&enzyme=Trypsin&access_method=Accession+Number&accession_num=AFK19156.1&coverage_map=0+25+28+167) | 4.23e+006 |
| Acyl-CoA dehydrogenase | [AFK19198.1](http://smserver.sti.ua.es/millhtml/SM_instruct/servadmn.htm#update_acclinks?AFK19198.1) | 2 | 2 | 31.71 | [9.1](http://smserver.sti.ua.es/millbin/msdigest.cgi?missed_cleavages=2&msparams_dir=msparams_mill/&hide_protein_sequence=2&database=PA_haloferax_mediterranei_genbank&seqdb_dir=D:\SeqDB\&enzyme=Trypsin&access_method=Accession+Number&accession_num=AFK19198.1&coverage_map=0+206+23+15+12+128) | 7.48e+005 |
| Endoribonuclease L-PSP | [AFK18161.1](http://smserver.sti.ua.es/millhtml/SM_instruct/servadmn.htm#update_acclinks?AFK18161.1) | 2 | 2 | 31.70 | [25](http://smserver.sti.ua.es/millbin/msdigest.cgi?missed_cleavages=2&msparams_dir=msparams_mill/&hide_protein_sequence=2&database=PA_haloferax_mediterranei_genbank&seqdb_dir=D:\SeqDB\&enzyme=Trypsin&access_method=Accession+Number&accession_num=AFK18161.1&coverage_map=0+52+22+29+10+15) | 9.98e+005 |
| Thioesterase (plasmid) | [AFK21493.1](http://smserver.sti.ua.es/millhtml/SM_instruct/servadmn.htm#update_acclinks?AFK21493.1) | 2 | 2 | 31.17 | [28.3](http://smserver.sti.ua.es/millbin/msdigest.cgi?missed_cleavages=2&msparams_dir=msparams_mill/&hide_protein_sequence=2&database=PA_haloferax_mediterranei_genbank&seqdb_dir=D:\SeqDB\&enzyme=Trypsin&access_method=Accession+Number&accession_num=AFK21493.1&coverage_map=0+78+12+7+24+6) | 9.94e+005 |
| Hypothetical protein HFX_1622 | [AFK19328.1](http://smserver.sti.ua.es/millhtml/SM_instruct/servadmn.htm#update_acclinks?AFK19328.1) | 2 | 2 | 30.96 | [23.7](http://smserver.sti.ua.es/millbin/msdigest.cgi?missed_cleavages=2&msparams_dir=msparams_mill/&hide_protein_sequence=2&database=PA_haloferax_mediterranei_genbank&seqdb_dir=D:\SeqDB\&enzyme=Trypsin&access_method=Accession+Number&accession_num=AFK19328.1&coverage_map=0+15+18+68+10+7) | 2.25e+006 |
| OsmC family protein | [AFK19097.2](http://smserver.sti.ua.es/millhtml/SM_instruct/servadmn.htm#update_acclinks?AFK19097.2) | 2 | 2 | 30.92 | [20.5](http://smserver.sti.ua.es/millbin/msdigest.cgi?missed_cleavages=2&msparams_dir=msparams_mill/&hide_protein_sequence=2&database=PA_haloferax_mediterranei_genbank&seqdb_dir=D:\SeqDB\&enzyme=Trypsin&access_method=Accession+Number&accession_num=AFK19097.2&coverage_map=0+21+15+91+14) | 3.88e+006 |
| Hsp20 type chaperone | [AFK18447.1](http://smserver.sti.ua.es/millhtml/SM_instruct/servadmn.htm#update_acclinks?AFK18447.1) | 2 | 2 | 30.79 | [21.6](http://smserver.sti.ua.es/millbin/msdigest.cgi?missed_cleavages=2&msparams_dir=msparams_mill/&hide_protein_sequence=2&database=PA_haloferax_mediterranei_genbank&seqdb_dir=D:\SeqDB\&enzyme=Trypsin&access_method=Accession+Number&accession_num=AFK18447.1&coverage_map=0+74+9+4+27+52) | 7.06e+005 |
| Uridine phosphorylase | [AFK20307.1](http://smserver.sti.ua.es/millhtml/SM_instruct/servadmn.htm#update_acclinks?AFK20307.1) | 2 | 2 | 30.78 | [10.3](http://smserver.sti.ua.es/millbin/msdigest.cgi?missed_cleavages=2&msparams_dir=msparams_mill/&hide_protein_sequence=2&database=PA_haloferax_mediterranei_genbank&seqdb_dir=D:\SeqDB\&enzyme=Trypsin&access_method=Accession+Number&accession_num=AFK20307.1&coverage_map=0+79+8+107+17+31) | 2.71e+006 |
| Hypothetical protein HFX_1243 | [AFK18956.1](http://smserver.sti.ua.es/millhtml/SM_instruct/servadmn.htm#update_acclinks?AFK18956.1) | 2 | 2 | 30.71 | [7.9](http://smserver.sti.ua.es/millbin/msdigest.cgi?missed_cleavages=2&msparams_dir=msparams_mill/&hide_protein_sequence=2&database=PA_haloferax_mediterranei_genbank&seqdb_dir=D:\SeqDB\&enzyme=Trypsin&access_method=Accession+Number&accession_num=AFK18956.1&coverage_map=0+114+17+10+13+222) | 6.01e+006 |
| Hypothetical protein HFX_5285 (plasmid) | [AFK21116.1](http://smserver.sti.ua.es/millhtml/SM_instruct/servadmn.htm#update_acclinks?AFK21116.1) | 2 | 2 | 30.59 | [18.7](http://smserver.sti.ua.es/millbin/msdigest.cgi?missed_cleavages=2&msparams_dir=msparams_mill/&hide_protein_sequence=2&database=PA_haloferax_mediterranei_genbank&seqdb_dir=D:\SeqDB\&enzyme=Trypsin&access_method=Accession+Number&accession_num=AFK21116.1&coverage_map=0+180+26+16+20+3) | 1.65e+006 |
| Hypothetical protein HFX_1139 | [AFK18855.1](http://smserver.sti.ua.es/millhtml/SM_instruct/servadmn.htm#update_acclinks?AFK18855.1) | 2 | 2 | 30.47 | [31.1](http://smserver.sti.ua.es/millbin/msdigest.cgi?missed_cleavages=2&msparams_dir=msparams_mill/&hide_protein_sequence=2&database=PA_haloferax_mediterranei_genbank&seqdb_dir=D:\SeqDB\&enzyme=Trypsin&access_method=Accession+Number&accession_num=AFK18855.1&coverage_map=0+15+19+27) | 1.11e+006 |
| Pyridoxal biosynthesis lyase PdxS | [AFK20032.1](http://smserver.sti.ua.es/millhtml/SM_instruct/servadmn.htm#update_acclinks?AFK20032.1) | 2 | 2 | 30.41 | [6.2](http://smserver.sti.ua.es/millbin/msdigest.cgi?missed_cleavages=2&msparams_dir=msparams_mill/&hide_protein_sequence=2&database=PA_haloferax_mediterranei_genbank&seqdb_dir=D:\SeqDB\&enzyme=Trypsin&access_method=Accession+Number&accession_num=AFK20032.1&coverage_map=0+146+9+116+10+21) | 6.92e+005 |
| Phosphonates ABC transporter permease protein | [AFK19844.1](http://smserver.sti.ua.es/millhtml/SM_instruct/servadmn.htm#update_acclinks?AFK19844.1) | 2 | 2 | 30.25 | [9.7](http://smserver.sti.ua.es/millbin/msdigest.cgi?missed_cleavages=2&msparams_dir=msparams_mill/&hide_protein_sequence=2&database=PA_haloferax_mediterranei_genbank&seqdb_dir=D:\SeqDB\&enzyme=Trypsin&access_method=Accession+Number&accession_num=AFK19844.1&coverage_map=0+53+8+99+18+90) | 6.86e+005 |

Table S4. Representative proteins identified in the micelles with Nar and Nir activities obtained after the use of HiPrep™ Q-Sepharose 16/10 FF from 3 LC-MS/MS runs.

| **Protein name** | **Database**  **Accession** | **Spectra** | **Distinct**  **Peptides** | **Distinct**  **Summed**  **MS/MS Search**  **Score** | **% AA**  **Coverage** | **Total Protein**  **Spectral Intensity** |
| --- | --- | --- | --- | --- | --- | --- |
| nitrate reductase alpha chain (plasmid) | [AFK20939.1](http://smserver.sti.ua.es/millhtml/SM_instruct/servadmn.htm#update_acclinks?AFK20939.1) | 28 | 22 | 380.05 | [34.4](http://smserver.sti.ua.es/millbin/msdigest.cgi?missed_cleavages=2&msparams_dir=msparams_mill/&hide_protein_sequence=2&database=PA_haloferax_mediterranei_genbank&seqdb_dir=D:\SeqDB\&enzyme=Trypsin&access_method=Accession+Number&accession_num=AFK20939.1&coverage_map=0+90+7+43+11+35+22+144+10+8+26+19+22+4+7+54+27+50+10+23+42+26+7+25+82+6+9+21+20+18+37+79) | 3.88e+007 |
| dipeptide ABC transporter ATP-binding protein | [AFK18330.2](http://smserver.sti.ua.es/millhtml/SM_instruct/servadmn.htm#update_acclinks?AFK18330.2) | 34 | 18 | 354.37 | [50.1](http://smserver.sti.ua.es/millbin/msdigest.cgi?missed_cleavages=2&msparams_dir=msparams_mill/&hide_protein_sequence=2&database=PA_haloferax_mediterranei_genbank&seqdb_dir=D:\SeqDB\&enzyme=Trypsin&access_method=Accession+Number&accession_num=AFK18330.2&coverage_map=0+68+58+4+17+42+136+53+25+93+51+25) | 3.10e+008 |
| nitrous-oxide reductase (plasmid) | [AFK20926.1](http://smserver.sti.ua.es/millhtml/SM_instruct/servadmn.htm#update_acclinks?AFK20926.1) | 25 | 23 | 349.62 | [38.2](http://smserver.sti.ua.es/millbin/msdigest.cgi?missed_cleavages=2&msparams_dir=msparams_mill/&hide_protein_sequence=2&database=PA_haloferax_mediterranei_genbank&seqdb_dir=D:\SeqDB\&enzyme=Trypsin&access_method=Accession+Number&accession_num=AFK20926.1&coverage_map=0+122+8+14+34+4+7+47+15+35+20+7+9+8+25+25+20+8+48+56+27+7+15+5+16+1+6+74+6+1) | 1.90e+008 |
| nucleoside-binding protein | [AFK19185.1](http://smserver.sti.ua.es/millhtml/SM_instruct/servadmn.htm#update_acclinks?AFK19185.1) | 23 | 16 | 297.84 | [59.3](http://smserver.sti.ua.es/millbin/msdigest.cgi?missed_cleavages=2&msparams_dir=msparams_mill/&hide_protein_sequence=2&database=PA_haloferax_mediterranei_genbank&seqdb_dir=D:\SeqDB\&enzyme=Trypsin&access_method=Accession+Number&accession_num=AFK19185.1&coverage_map=0+84+23+25+61+1+46+2+32+2+45+38+15) | 3.03e+008 |
| succinate dehydrogenase, subunit A (flavoprotein) | [AFK20495.1](http://smserver.sti.ua.es/millhtml/SM_instruct/servadmn.htm#update_acclinks?AFK20495.1) | 19 | 15 | 278.01 | [36.4](http://smserver.sti.ua.es/millbin/msdigest.cgi?missed_cleavages=2&msparams_dir=msparams_mill/&hide_protein_sequence=2&database=PA_haloferax_mediterranei_genbank&seqdb_dir=D:\SeqDB\&enzyme=Trypsin&access_method=Accession+Number&accession_num=AFK20495.1&coverage_map=0+17+17+34+16+36+22+4+42+33+43+22+22+73+14+93+13+29+29+16+6+34) | 7.91e+007 |
| proline dehydrogenase | [AFK18055.1](http://smserver.sti.ua.es/millhtml/SM_instruct/servadmn.htm#update_acclinks?AFK18055.1) | 21 | 15 | 271.67 | [67](http://smserver.sti.ua.es/millbin/msdigest.cgi?missed_cleavages=2&msparams_dir=msparams_mill/&hide_protein_sequence=2&database=PA_haloferax_mediterranei_genbank&seqdb_dir=D:\SeqDB\&enzyme=Trypsin&access_method=Accession+Number&accession_num=AFK18055.1&coverage_map=0+8+36+48+57+12+12+2+55+5+18+12+9+5) | 5.92e+007 |
| proline dehydrogenase | [AFK18914.1](http://smserver.sti.ua.es/millhtml/SM_instruct/servadmn.htm#update_acclinks?AFK18914.1) | 9 | 7 | 112.84 | [34.4](http://smserver.sti.ua.es/millbin/msdigest.cgi?missed_cleavages=2&msparams_dir=msparams_mill/&hide_protein_sequence=2&database=PA_haloferax_mediterranei_genbank&seqdb_dir=D:\SeqDB\&enzyme=Trypsin&access_method=Accession+Number&accession_num=AFK18914.1&coverage_map=0+44+15+124+47+5+25+5+9+5) | 1.21e+007 |
| A-type ATP synthase subunit E | [AFK18038.1](http://smserver.sti.ua.es/millhtml/SM_instruct/servadmn.htm#update_acclinks?AFK18038.1) | 32 | 12 | 245.46 | [72.1](http://smserver.sti.ua.es/millbin/msdigest.cgi?missed_cleavages=2&msparams_dir=msparams_mill/&hide_protein_sequence=2&database=PA_haloferax_mediterranei_genbank&seqdb_dir=D:\SeqDB\&enzyme=Trypsin&access_method=Accession+Number&accession_num=AFK18038.1&coverage_map=0+15+42+27+17+5+58+2+23+5) | 3.81e+008 |
| sugar ABC transporter substrate binding protein | [AFK20386.1](http://smserver.sti.ua.es/millhtml/SM_instruct/servadmn.htm#update_acclinks?AFK20386.1) | 14 | 12 | 216.60 | [38.5](http://smserver.sti.ua.es/millbin/msdigest.cgi?missed_cleavages=2&msparams_dir=msparams_mill/&hide_protein_sequence=2&database=PA_haloferax_mediterranei_genbank&seqdb_dir=D:\SeqDB\&enzyme=Trypsin&access_method=Accession+Number&accession_num=AFK20386.1&coverage_map=0+78+8+22+31+8+44+74+24+21+27+41+25+48+38+22) | 3.85e+007 |
| A-type ATP synthase subunit C | [AFK18039.1](http://smserver.sti.ua.es/millhtml/SM_instruct/servadmn.htm#update_acclinks?AFK18039.1) | 17 | 12 | 215.91 | [43.3](http://smserver.sti.ua.es/millbin/msdigest.cgi?missed_cleavages=2&msparams_dir=msparams_mill/&hide_protein_sequence=2&database=PA_haloferax_mediterranei_genbank&seqdb_dir=D:\SeqDB\&enzyme=Trypsin&access_method=Accession+Number&accession_num=AFK18039.1&coverage_map=0+20+10+4+61+15+17+56+17+3+10+29+17+9+19+61) | 7.89e+007 |
| A-type ATP synthase subunit I | [AFK18036.1](http://smserver.sti.ua.es/millhtml/SM_instruct/servadmn.htm#update_acclinks?AFK18036.1) | 15 | 11 | 214.94 | [16.1](http://smserver.sti.ua.es/millbin/msdigest.cgi?missed_cleavages=2&msparams_dir=msparams_mill/&hide_protein_sequence=2&database=PA_haloferax_mediterranei_genbank&seqdb_dir=D:\SeqDB\&enzyme=Trypsin&access_method=Accession+Number&accession_num=AFK18036.1&coverage_map=0+70+12+2+25+50+18+55+32+24+23+291+8+119) | 8.48e+007 |
| NADH dehydrogenase, subunit CD (ubiquinone) | [AFK18696.1](http://smserver.sti.ua.es/millhtml/SM_instruct/servadmn.htm#update_acclinks?AFK18696.1) | 18 | 13 | 214.59 | [30.7](http://smserver.sti.ua.es/millbin/msdigest.cgi?missed_cleavages=2&msparams_dir=msparams_mill/&hide_protein_sequence=2&database=PA_haloferax_mediterranei_genbank&seqdb_dir=D:\SeqDB\&enzyme=Trypsin&access_method=Accession+Number&accession_num=AFK18696.1&coverage_map=0+20+15+56+12+101+20+21+42+28+16+7+12+6+28+23+9+39+17+85) | 3.52e+007 |
| NADH dehydrogenase, subunit D (ubiquinone) | [AFK18684.1](http://smserver.sti.ua.es/millhtml/SM_instruct/servadmn.htm#update_acclinks?AFK18684.1) | 6 | 4 | 66.43 | [6.6](http://smserver.sti.ua.es/millbin/msdigest.cgi?missed_cleavages=2&msparams_dir=msparams_mill/&hide_protein_sequence=2&database=PA_haloferax_mediterranei_genbank&seqdb_dir=D:\SeqDB\&enzyme=Trypsin&access_method=Accession+Number&accession_num=AFK18684.1&coverage_map=0+312+16+7+12+57+9+141) | 1.84e+007 |
| A-type ATP synthase subunit H | [AFK18035.1](http://smserver.sti.ua.es/millhtml/SM_instruct/servadmn.htm#update_acclinks?AFK18035.1) | 18 | 12 | 212.54 | [80.9](http://smserver.sti.ua.es/millbin/msdigest.cgi?missed_cleavages=2&msparams_dir=msparams_mill/&hide_protein_sequence=2&database=PA_haloferax_mediterranei_genbank&seqdb_dir=D:\SeqDB\&enzyme=Trypsin&access_method=Accession+Number&accession_num=AFK18035.1&coverage_map=0+3+6+2+25+7+19+9+39) | 1.95e+008 |
| ABC-type dipeptide/oligopeptide/nickel transport system, substrate binding protein | [AFK20131.1](http://smserver.sti.ua.es/millhtml/SM_instruct/servadmn.htm#update_acclinks?AFK20131.1) | 16 | 11 | 204.37 | [36.4](http://smserver.sti.ua.es/millbin/msdigest.cgi?missed_cleavages=2&msparams_dir=msparams_mill/&hide_protein_sequence=2&database=PA_haloferax_mediterranei_genbank&seqdb_dir=D:\SeqDB\&enzyme=Trypsin&access_method=Accession+Number&accession_num=AFK20131.1&coverage_map=0+99+16+34+32+31+14+22+9+8+22+23+10+76+41+34+53+17) | 4.70e+007 |
| A-type ATP synthase subunit A | [AFK18041.1](http://smserver.sti.ua.es/millhtml/SM_instruct/servadmn.htm#update_acclinks?AFK18041.1) | 16 | 11 | 197.16 | [28.8](http://smserver.sti.ua.es/millbin/msdigest.cgi?missed_cleavages=2&msparams_dir=msparams_mill/&hide_protein_sequence=2&database=PA_haloferax_mediterranei_genbank&seqdb_dir=D:\SeqDB\&enzyme=Trypsin&access_method=Accession+Number&accession_num=AFK18041.1&coverage_map=0+268+18+8+15+15+15+75+12+35+37+3+30+7+19+3+23+3) | 1.94e+007 |
| poly(3-hydroxyalkanoate) synthase subunit PhaE (plasmid) | [AFK21053.1](http://smserver.sti.ua.es/millhtml/SM_instruct/servadmn.htm#update_acclinks?AFK21053.1) | 19 | 9 | 188.11 | [71.4](http://smserver.sti.ua.es/millbin/msdigest.cgi?missed_cleavages=2&msparams_dir=msparams_mill/&hide_protein_sequence=2&database=PA_haloferax_mediterranei_genbank&seqdb_dir=D:\SeqDB\&enzyme=Trypsin&access_method=Accession+Number&accession_num=AFK21053.1&coverage_map=0+5+21+36+100+11+9) | 1.74e+008 |
| iron-sulfur protein (4Fe-4S) | [AFK17960.2](http://smserver.sti.ua.es/millhtml/SM_instruct/servadmn.htm#update_acclinks?AFK17960.2) | 13 | 11 | 187.43 | [26.5](http://smserver.sti.ua.es/millbin/msdigest.cgi?missed_cleavages=2&msparams_dir=msparams_mill/&hide_protein_sequence=2&database=PA_haloferax_mediterranei_genbank&seqdb_dir=D:\SeqDB\&enzyme=Trypsin&access_method=Accession+Number&accession_num=AFK17960.2&coverage_map=0+46+11+237+10+20+7+105+19+10+25+67+23+13+11+2+27+2+30+1+24+14) | 3.63e+007 |
| nitrite reductase copper containing protein | [AFK19882.1](http://smserver.sti.ua.es/millhtml/SM_instruct/servadmn.htm#update_acclinks?AFK19882.1) | 17 | 12 | 182.42 | [46.3](http://smserver.sti.ua.es/millbin/msdigest.cgi?missed_cleavages=2&msparams_dir=msparams_mill/&hide_protein_sequence=2&database=PA_haloferax_mediterranei_genbank&seqdb_dir=D:\SeqDB\&enzyme=Trypsin&access_method=Accession+Number&accession_num=AFK19882.1&coverage_map=0+85+36+29+14+29+11+4+14+8+12+2+10+4+17+21+49+25+16) | 5.32e+007 |
| serine protease (plasmid) | [AFK21203.1](http://smserver.sti.ua.es/millhtml/SM_instruct/servadmn.htm#update_acclinks?AFK21203.1) | 12 | 11 | 181.73 | [28.1](http://smserver.sti.ua.es/millbin/msdigest.cgi?missed_cleavages=2&msparams_dir=msparams_mill/&hide_protein_sequence=2&database=PA_haloferax_mediterranei_genbank&seqdb_dir=D:\SeqDB\&enzyme=Trypsin&access_method=Accession+Number&accession_num=AFK21203.1&coverage_map=0+15+11+40+31+57+14+115+20+42+28+2+17+49+8+31+18+46+15+17) | 1.84e+007 |
| immunogenic protein | [AFK20638.1](http://smserver.sti.ua.es/millhtml/SM_instruct/servadmn.htm#update_acclinks?AFK20638.1) | 16 | 8 | 174.36 | [41.9](http://smserver.sti.ua.es/millbin/msdigest.cgi?missed_cleavages=2&msparams_dir=msparams_mill/&hide_protein_sequence=2&database=PA_haloferax_mediterranei_genbank&seqdb_dir=D:\SeqDB\&enzyme=Trypsin&access_method=Accession+Number&accession_num=AFK20638.1&coverage_map=0+57+82+71+37+63+22+4) | 3.71e+008 |
| poly(3-hydroxyalkanoate) synthase subunit PhaC (plasmid) | [AFK21054.1](http://smserver.sti.ua.es/millhtml/SM_instruct/servadmn.htm#update_acclinks?AFK21054.1) | 12 | 10 | 170.53 | [31](http://smserver.sti.ua.es/millbin/msdigest.cgi?missed_cleavages=2&msparams_dir=msparams_mill/&hide_protein_sequence=2&database=PA_haloferax_mediterranei_genbank&seqdb_dir=D:\SeqDB\&enzyme=Trypsin&access_method=Accession+Number&accession_num=AFK21054.1&coverage_map=0+13+11+66+46+32+20+43+33+185+43) | 1.59e+007 |
| SPFH domain, Band 7 family protein | [AFK18490.1](http://smserver.sti.ua.es/millhtml/SM_instruct/servadmn.htm#update_acclinks?AFK18490.1) | 12 | 11 | 169.54 | [32.3](http://smserver.sti.ua.es/millbin/msdigest.cgi?missed_cleavages=2&msparams_dir=msparams_mill/&hide_protein_sequence=2&database=PA_haloferax_mediterranei_genbank&seqdb_dir=D:\SeqDB\&enzyme=Trypsin&access_method=Accession+Number&accession_num=AFK18490.1&coverage_map=0+41+10+1+15+7+8+26+9+1+26+51+10+108+60+53) | 1.47e+007 |
| PBS lyase HEAT-like repeat protein | [AFK18736.1](http://smserver.sti.ua.es/millhtml/SM_instruct/servadmn.htm#update_acclinks?AFK18736.1) | 11 | 9 | 166.11 | [23](http://smserver.sti.ua.es/millbin/msdigest.cgi?missed_cleavages=2&msparams_dir=msparams_mill/&hide_protein_sequence=2&database=PA_haloferax_mediterranei_genbank&seqdb_dir=D:\SeqDB\&enzyme=Trypsin&access_method=Accession+Number&accession_num=AFK18736.1&coverage_map=0+73+27+96+16+70+24+35+9+18+8+24+13+7) | 9.36e+007 |
| nitrite reductase (NO-forming) | [AFK19872.1](http://smserver.sti.ua.es/millhtml/SM_instruct/servadmn.htm#update_acclinks?AFK19872.1) | 11 | 12 | 161.07 | [32.8](http://smserver.sti.ua.es/millbin/msdigest.cgi?missed_cleavages=2&msparams_dir=msparams_mill/&hide_protein_sequence=2&database=PA_haloferax_mediterranei_genbank&seqdb_dir=D:\SeqDB\&enzyme=Trypsin&access_method=Accession+Number&accession_num=AFK19872.1&coverage_map=0+50+18+132+13+7+43+41+29+11+15) | 2.40e+008 |
| dipeptide ABC transporter dipeptide-binding protein | [AFK17806.1](http://smserver.sti.ua.es/millhtml/SM_instruct/servadmn.htm#update_acclinks?AFK17806.1) | 10 | 10 | 151.07 | [21.1](http://smserver.sti.ua.es/millbin/msdigest.cgi?missed_cleavages=2&msparams_dir=msparams_mill/&hide_protein_sequence=2&database=PA_haloferax_mediterranei_genbank&seqdb_dir=D:\SeqDB\&enzyme=Trypsin&access_method=Accession+Number&accession_num=AFK17806.1&coverage_map=0+60+35+169+10+33+16+48+9+29+9+5+10+3+9+23+12+56+12+34+8+25) | 1.43e+007 |
| dihydroorotase | [AFK19587.1](http://smserver.sti.ua.es/millhtml/SM_instruct/servadmn.htm#update_acclinks?AFK19587.1) | 9 | 8 | 145.79 | [32.1](http://smserver.sti.ua.es/millbin/msdigest.cgi?missed_cleavages=2&msparams_dir=msparams_mill/&hide_protein_sequence=2&database=PA_haloferax_mediterranei_genbank&seqdb_dir=D:\SeqDB\&enzyme=Trypsin&access_method=Accession+Number&accession_num=AFK19587.1&coverage_map=0+160+43+14+24+17+11+11+6+57+16+2+18+30+20) | 3.36e+006 |
| periplasmic solute binding protein | [AFK20085.1](http://smserver.sti.ua.es/millhtml/SM_instruct/servadmn.htm#update_acclinks?AFK20085.1) | 8 | 8 | 144.87 | [31.5](http://smserver.sti.ua.es/millbin/msdigest.cgi?missed_cleavages=2&msparams_dir=msparams_mill/&hide_protein_sequence=2&database=PA_haloferax_mediterranei_genbank&seqdb_dir=D:\SeqDB\&enzyme=Trypsin&access_method=Accession+Number&accession_num=AFK20085.1&coverage_map=0+125+47+37+11+3+17+36+10+41+28+3) | 1.37e+007 |
| catalase (including: peroxidase) | [AFK19564.1](http://smserver.sti.ua.es/millhtml/SM_instruct/servadmn.htm#update_acclinks?AFK19564.1) | 12 | 8 | 141.07 | [14.5](http://smserver.sti.ua.es/millbin/msdigest.cgi?missed_cleavages=2&msparams_dir=msparams_mill/&hide_protein_sequence=2&database=PA_haloferax_mediterranei_genbank&seqdb_dir=D:\SeqDB\&enzyme=Trypsin&access_method=Accession+Number&accession_num=AFK19564.1&coverage_map=0+14+9+142+12+256+15+62+17+59+19+51+8+3+10+2+14+21) | 2.77e+007 |
| A-type ATP synthase subunit B | [AFK18042.1](http://smserver.sti.ua.es/millhtml/SM_instruct/servadmn.htm#update_acclinks?AFK18042.1) | 11 | 8 | 136.27 | [22](http://smserver.sti.ua.es/millbin/msdigest.cgi?missed_cleavages=2&msparams_dir=msparams_mill/&hide_protein_sequence=2&database=PA_haloferax_mediterranei_genbank&seqdb_dir=D:\SeqDB\&enzyme=Trypsin&access_method=Accession+Number&accession_num=AFK18042.1&coverage_map=0+107+12+84+21+5+13+32+16+63+41+74) | 2.13e+007 |
| halocyanin precursor-like protein | [AFK19879.1](http://smserver.sti.ua.es/millhtml/SM_instruct/servadmn.htm#update_acclinks?AFK19879.1) | 9 | 8 | 136.09 | [42.3](http://smserver.sti.ua.es/millbin/msdigest.cgi?missed_cleavages=2&msparams_dir=msparams_mill/&hide_protein_sequence=2&database=PA_haloferax_mediterranei_genbank&seqdb_dir=D:\SeqDB\&enzyme=Trypsin&access_method=Accession+Number&accession_num=AFK19879.1&coverage_map=0+52+10+27+62+62+22+28+61+42) | 1.16e+007 |
| nitrate reductase beta chain (plasmid) | [AFK20938.1](http://smserver.sti.ua.es/millhtml/SM_instruct/servadmn.htm#update_acclinks?AFK20938.1) | 11 | 9 | 136.08 | [27.5](http://smserver.sti.ua.es/millbin/msdigest.cgi?missed_cleavages=2&msparams_dir=msparams_mill/&hide_protein_sequence=2&database=PA_haloferax_mediterranei_genbank&seqdb_dir=D:\SeqDB\&enzyme=Trypsin&access_method=Accession+Number&accession_num=AFK20938.1&coverage_map=0+67+10+69+16+5+13+27+7+24+14+46+20+7+17+10) | 2.12e+007 |
| hypothetical protein HFX_6060 (plasmid) | [AFK21187.1](http://smserver.sti.ua.es/millhtml/SM_instruct/servadmn.htm#update_acclinks?AFK21187.1) | 9 | 8 | 133.28 | [30.3](http://smserver.sti.ua.es/millbin/msdigest.cgi?missed_cleavages=2&msparams_dir=msparams_mill/&hide_protein_sequence=2&database=PA_haloferax_mediterranei_genbank&seqdb_dir=D:\SeqDB\&enzyme=Trypsin&access_method=Accession+Number&accession_num=AFK21187.1&coverage_map=0+151+24+33+31+16+32+3+16+33) | 9.87e+006 |
| oxidoreductase | [AFK18898.2](http://smserver.sti.ua.es/millhtml/SM_instruct/servadmn.htm#update_acclinks?AFK18898.2) | 8 | 8 | 131.22 | [34.7](http://smserver.sti.ua.es/millbin/msdigest.cgi?missed_cleavages=2&msparams_dir=msparams_mill/&hide_protein_sequence=2&database=PA_haloferax_mediterranei_genbank&seqdb_dir=D:\SeqDB\&enzyme=Trypsin&access_method=Accession+Number&accession_num=AFK18898.2&coverage_map=0+19+13+8+10+6+27+12+24+25+10+24+12+29+8+72) | 5.65e+006 |
| hypothetical protein HFX_1575 | [AFK19282.2](http://smserver.sti.ua.es/millhtml/SM_instruct/servadmn.htm#update_acclinks?AFK19282.2) | 10 | 7 | 129.06 | [47.8](http://smserver.sti.ua.es/millbin/msdigest.cgi?missed_cleavages=2&msparams_dir=msparams_mill/&hide_protein_sequence=2&database=PA_haloferax_mediterranei_genbank&seqdb_dir=D:\SeqDB\&enzyme=Trypsin&access_method=Accession+Number&accession_num=AFK19282.2&coverage_map=0+77+46+4+36+14+27+24) | 4.61e+007 |
| carbohydrate ABC transporter substrate-binding protein, CUT1 family | [AFK20566.1](http://smserver.sti.ua.es/millhtml/SM_instruct/servadmn.htm#update_acclinks?AFK20566.1) | 10 | 6 | 126.05 | [26.2](http://smserver.sti.ua.es/millbin/msdigest.cgi?missed_cleavages=2&msparams_dir=msparams_mill/&hide_protein_sequence=2&database=PA_haloferax_mediterranei_genbank&seqdb_dir=D:\SeqDB\&enzyme=Trypsin&access_method=Accession+Number&accession_num=AFK20566.1&coverage_map=0+90+26+69+47+45+21+5+15+47+10+79) | 2.69e+007 |
| hypothetical protein HFX_1144 | [AFK18860.1](http://smserver.sti.ua.es/millhtml/SM_instruct/servadmn.htm#update_acclinks?AFK18860.1) | 8 | 8 | 122.77 | [45.1](http://smserver.sti.ua.es/millbin/msdigest.cgi?missed_cleavages=2&msparams_dir=msparams_mill/&hide_protein_sequence=2&database=PA_haloferax_mediterranei_genbank&seqdb_dir=D:\SeqDB\&enzyme=Trypsin&access_method=Accession+Number&accession_num=AFK18860.1&coverage_map=0+48+13+1+14+44+61+70+25+2+23) | 3.65e+006 |
| stress response protein (plasmid) | [AFK21518.1](http://smserver.sti.ua.es/millhtml/SM_instruct/servadmn.htm#update_acclinks?AFK21518.1) | 8 | 7 | 121.34 | [36.3](http://smserver.sti.ua.es/millbin/msdigest.cgi?missed_cleavages=2&msparams_dir=msparams_mill/&hide_protein_sequence=2&database=PA_haloferax_mediterranei_genbank&seqdb_dir=D:\SeqDB\&enzyme=Trypsin&access_method=Accession+Number&accession_num=AFK21518.1&coverage_map=0+43+16+69+32+4+32+68+25) | 3.14e+007 |
| dipeptide/oligopeptide/nickel ABC transporter periplasmic substrate-binding protein | [AFK18790.1](http://smserver.sti.ua.es/millhtml/SM_instruct/servadmn.htm#update_acclinks?AFK18790.1) | 8 | 8 | 120.96 | [17.6](http://smserver.sti.ua.es/millbin/msdigest.cgi?missed_cleavages=2&msparams_dir=msparams_mill/&hide_protein_sequence=2&database=PA_haloferax_mediterranei_genbank&seqdb_dir=D:\SeqDB\&enzyme=Trypsin&access_method=Accession+Number&accession_num=AFK18790.1&coverage_map=0+80+30+199+18+42+9+3+16+68+14+70+9+47+13) | 5.93e+006 |
| hypothetical protein HFX_0491 | AFK18222.1 | 10 | 6 | 117.31 | 52.4 | 4.89e+007 |
| hypothetical protein HFX_2343 | [AFK20030.1](http://smserver.sti.ua.es/millhtml/SM_instruct/servadmn.htm#update_acclinks?AFK20030.1) | 8 | 7 | 116.90 | [32.1](http://smserver.sti.ua.es/millbin/msdigest.cgi?missed_cleavages=2&msparams_dir=msparams_mill/&hide_protein_sequence=2&database=PA_haloferax_mediterranei_genbank&seqdb_dir=D:\SeqDB\&enzyme=Trypsin&access_method=Accession+Number&accession_num=AFK20030.1&coverage_map=0+6+13+87+25+35+18+28+27+31+17+37+13+14) | 6.63e+006 |
| sulfatase arylsulfatase A-like protein | [AFK18425.2](http://smserver.sti.ua.es/millhtml/SM_instruct/servadmn.htm#update_acclinks?AFK18425.2) | 9 | 8 | 115.50 | [15.3](http://smserver.sti.ua.es/millbin/msdigest.cgi?missed_cleavages=2&msparams_dir=msparams_mill/&hide_protein_sequence=2&database=PA_haloferax_mediterranei_genbank&seqdb_dir=D:\SeqDB\&enzyme=Trypsin&access_method=Accession+Number&accession_num=AFK18425.2&coverage_map=0+82+13+35+12+69+7+9+16+64+8+19+13+102) | 1.22e+007 |
| hypothetical protein HFX_1589 | [AFK19295.1](http://smserver.sti.ua.es/millhtml/SM_instruct/servadmn.htm#update_acclinks?AFK19295.1) | 7 | 7 | 112.25 | [17.4](http://smserver.sti.ua.es/millbin/msdigest.cgi?missed_cleavages=2&msparams_dir=msparams_mill/&hide_protein_sequence=2&database=PA_haloferax_mediterranei_genbank&seqdb_dir=D:\SeqDB\&enzyme=Trypsin&access_method=Accession+Number&accession_num=AFK19295.1&coverage_map=0+94+25+28+7+49+14+47+12+28+7+14+9+78+20+108) | 7.26e+006 |
| acetyl transferase | [AFK20422.1](http://smserver.sti.ua.es/millhtml/SM_instruct/servadmn.htm#update_acclinks?AFK20422.1) | 9 | 6 | 110.63 | [36.8](http://smserver.sti.ua.es/millbin/msdigest.cgi?missed_cleavages=2&msparams_dir=msparams_mill/&hide_protein_sequence=2&database=PA_haloferax_mediterranei_genbank&seqdb_dir=D:\SeqDB\&enzyme=Trypsin&access_method=Accession+Number&accession_num=AFK20422.1&coverage_map=0+15+13+57+41+31+16+11+6+38+14+2) | 1.55e+007 |
| naphthoate synthase | [AFK19237.1](http://smserver.sti.ua.es/millhtml/SM_instruct/servadmn.htm#update_acclinks?AFK19237.1) | 7 | 7 | 110.00 | [27.8](http://smserver.sti.ua.es/millbin/msdigest.cgi?missed_cleavages=2&msparams_dir=msparams_mill/&hide_protein_sequence=2&database=PA_haloferax_mediterranei_genbank&seqdb_dir=D:\SeqDB\&enzyme=Trypsin&access_method=Accession+Number&accession_num=AFK19237.1&coverage_map=0+35+28+2+16+15+22+83+7+67+12+18) | 7.35e+006 |
| hypothetical protein HFX_1586 | [AFK19292.1](http://smserver.sti.ua.es/millhtml/SM_instruct/servadmn.htm#update_acclinks?AFK19292.1) | 8 | 7 | 108.80 | [19.5](http://smserver.sti.ua.es/millbin/msdigest.cgi?missed_cleavages=2&msparams_dir=msparams_mill/&hide_protein_sequence=2&database=PA_haloferax_mediterranei_genbank&seqdb_dir=D:\SeqDB\&enzyme=Trypsin&access_method=Accession+Number&accession_num=AFK19292.1&coverage_map=0+115+16+10+11+26+11+2+16+66+12+3+21+8+14+186) | 6.32e+006 |
| ferredoxin (2Fe-2S) | [AFK19572.1](http://smserver.sti.ua.es/millhtml/SM_instruct/servadmn.htm#update_acclinks?AFK19572.1) | 6 | 6 | 106.51 | [39.1](http://smserver.sti.ua.es/millbin/msdigest.cgi?missed_cleavages=2&msparams_dir=msparams_mill/&hide_protein_sequence=2&database=PA_haloferax_mediterranei_genbank&seqdb_dir=D:\SeqDB\&enzyme=Trypsin&access_method=Accession+Number&accession_num=AFK19572.1&coverage_map=0+108+29+10+47) | 2.25e+007 |
| cytochrome b subunit of nitric oxide reductase | [AFK19877.1](http://smserver.sti.ua.es/millhtml/SM_instruct/servadmn.htm#update_acclinks?AFK19877.1) | 9 | 6 | 106.03 | [15.6](http://smserver.sti.ua.es/millbin/msdigest.cgi?missed_cleavages=2&msparams_dir=msparams_mill/&hide_protein_sequence=2&database=PA_haloferax_mediterranei_genbank&seqdb_dir=D:\SeqDB\&enzyme=Trypsin&access_method=Accession+Number&accession_num=AFK19877.1&coverage_map=0+40+16+48+40+131+26+396+37+27) | 4.20e+006 |
| molybdate transport protein | [AFK19675.1](http://smserver.sti.ua.es/millhtml/SM_instruct/servadmn.htm#update_acclinks?AFK19675.1) | 8 | 7 | 106.01 | [30.1](http://smserver.sti.ua.es/millbin/msdigest.cgi?missed_cleavages=2&msparams_dir=msparams_mill/&hide_protein_sequence=2&database=PA_haloferax_mediterranei_genbank&seqdb_dir=D:\SeqDB\&enzyme=Trypsin&access_method=Accession+Number&accession_num=AFK19675.1&coverage_map=0+87+41+25+18+9+15+7+20+28+12+89) | 4.63e+006 |
| thioredoxin | [AFK18964.1](http://smserver.sti.ua.es/millhtml/SM_instruct/servadmn.htm#update_acclinks?AFK18964.1) | 9 | 6 | 103.10 | [49.4](http://smserver.sti.ua.es/millbin/msdigest.cgi?missed_cleavages=2&msparams_dir=msparams_mill/&hide_protein_sequence=2&database=PA_haloferax_mediterranei_genbank&seqdb_dir=D:\SeqDB\&enzyme=Trypsin&access_method=Accession+Number&accession_num=AFK18964.1&coverage_map=0+72+58+25+37) | 4.30e+007 |
| hypothetical protein HFX_6278 (plasmid) | [AFK21400.1](http://smserver.sti.ua.es/millhtml/SM_instruct/servadmn.htm#update_acclinks?AFK21400.1) | 6 | 6 | 101.55 | [48.8](http://smserver.sti.ua.es/millbin/msdigest.cgi?missed_cleavages=2&msparams_dir=msparams_mill/&hide_protein_sequence=2&database=PA_haloferax_mediterranei_genbank&seqdb_dir=D:\SeqDB\&enzyme=Trypsin&access_method=Accession+Number&accession_num=AFK21400.1&coverage_map=0+65+62) | 3.29e+007 |
| acetyl-CoA C-acetyltransferase (plasmid) | [AFK21477.1](http://smserver.sti.ua.es/millhtml/SM_instruct/servadmn.htm#update_acclinks?AFK21477.1) | 7 | 7 | 100.56 | [28](http://smserver.sti.ua.es/millbin/msdigest.cgi?missed_cleavages=2&msparams_dir=msparams_mill/&hide_protein_sequence=2&database=PA_haloferax_mediterranei_genbank&seqdb_dir=D:\SeqDB\&enzyme=Trypsin&access_method=Accession+Number&accession_num=AFK21477.1&coverage_map=0+7+8+5+24+47+14+58+9+13+22+60+19+59+11+25) | 4.90e+006 |
| acetyl-CoA C-ac(et)yltransferase | [AFK19698.1](http://smserver.sti.ua.es/millhtml/SM_instruct/servadmn.htm#update_acclinks?AFK19698.1) | 4 | 4 | 60.33 | [16.9](http://smserver.sti.ua.es/millbin/msdigest.cgi?missed_cleavages=2&msparams_dir=msparams_mill/&hide_protein_sequence=2&database=PA_haloferax_mediterranei_genbank&seqdb_dir=D:\SeqDB\&enzyme=Trypsin&access_method=Accession+Number&accession_num=AFK19698.1&coverage_map=0+16+43+16+12+75+9+206) | 1.96e+006 |
| 2-methylcitrate dehydratase (plasmid) | [AFK21002.1](http://smserver.sti.ua.es/millhtml/SM_instruct/servadmn.htm#update_acclinks?AFK21002.1) | 7 | 6 | 94.97 | [22.9](http://smserver.sti.ua.es/millbin/msdigest.cgi?missed_cleavages=2&msparams_dir=msparams_mill/&hide_protein_sequence=2&database=PA_haloferax_mediterranei_genbank&seqdb_dir=D:\SeqDB\&enzyme=Trypsin&access_method=Accession+Number&accession_num=AFK21002.1&coverage_map=0+12+16+2+21+19+15+61+42+237+9+15) | 3.02e+006 |
| aldehyde reductase | [AFK18579.1](http://smserver.sti.ua.es/millhtml/SM_instruct/servadmn.htm#update_acclinks?AFK18579.1) | 6 | 6 | 93.57 | [37.3](http://smserver.sti.ua.es/millbin/msdigest.cgi?missed_cleavages=2&msparams_dir=msparams_mill/&hide_protein_sequence=2&database=PA_haloferax_mediterranei_genbank&seqdb_dir=D:\SeqDB\&enzyme=Trypsin&access_method=Accession+Number&accession_num=AFK18579.1&coverage_map=0+38+8+33+16+55+27+18+29+21+23+8) | 2.35e+006 |
| electron transfer flavoprotein alpha subunit | [AFK20418.1](http://smserver.sti.ua.es/millhtml/SM_instruct/servadmn.htm#update_acclinks?AFK20418.1) | 6 | 6 | 91.97 | [13.6](http://smserver.sti.ua.es/millbin/msdigest.cgi?missed_cleavages=2&msparams_dir=msparams_mill/&hide_protein_sequence=2&database=PA_haloferax_mediterranei_genbank&seqdb_dir=D:\SeqDB\&enzyme=Trypsin&access_method=Accession+Number&accession_num=AFK20418.1&coverage_map=0+40+10+150+28+151+17+76+26+96) | 4.73e+006 |
| hypothetical protein HFX_2807 | [AFK20480.1](http://smserver.sti.ua.es/millhtml/SM_instruct/servadmn.htm#update_acclinks?AFK20480.1) | 5 | 5 | 91.34 | [54.7](http://smserver.sti.ua.es/millbin/msdigest.cgi?missed_cleavages=2&msparams_dir=msparams_mill/&hide_protein_sequence=2&database=PA_haloferax_mediterranei_genbank&seqdb_dir=D:\SeqDB\&enzyme=Trypsin&access_method=Accession+Number&accession_num=AFK20480.1&coverage_map=0+17+34+5+24+26) | 1.63e+007 |
| dihydrolipoamide dehydrogenase (plasmid) | [AFK21581.1](http://smserver.sti.ua.es/millhtml/SM_instruct/servadmn.htm#update_acclinks?AFK21581.1) | 7 | 6 | 90.72 | [20.9](http://smserver.sti.ua.es/millbin/msdigest.cgi?missed_cleavages=2&msparams_dir=msparams_mill/&hide_protein_sequence=2&database=PA_haloferax_mediterranei_genbank&seqdb_dir=D:\SeqDB\&enzyme=Trypsin&access_method=Accession+Number&accession_num=AFK21581.1&coverage_map=0+101+18+86+16+44+19+37+11+59+31+31) | 4.26e+006 |
| NADPH-dependent FMN reductase | AFK20556.2 | 6 | 4 | 86.29 | 46.8 | 1.32e+007 |
| ABC-type iron(III) transport system,substrate-binding protein | AFK19499.1 | 7 | 5 | 85.78 | 27.1 | 4.53e+007 |
| gas-vesicle operon protein gvpC | [AFK19401.1](http://smserver.sti.ua.es/millhtml/SM_instruct/servadmn.htm#update_acclinks?AFK19401.1) | 7 | 5 | 85.02 | [18.6](http://smserver.sti.ua.es/millbin/msdigest.cgi?missed_cleavages=2&msparams_dir=msparams_mill/&hide_protein_sequence=2&database=PA_haloferax_mediterranei_genbank&seqdb_dir=D:\SeqDB\&enzyme=Trypsin&access_method=Accession+Number&accession_num=AFK19401.1&coverage_map=0+39+24+126+30+145+17) | 2.92e+007 |
| protein-disulfide isomerase | AFK18965.1 | 6 | 4 | 84.04 | 29.7 | 1.43e+007 |
| ferredoxin (2Fe-2S) | [AFK20674.1](http://smserver.sti.ua.es/millhtml/SM_instruct/servadmn.htm#update_acclinks?AFK20674.1) | 10 | 4 | 84.01 | [37.2](http://smserver.sti.ua.es/millbin/msdigest.cgi?missed_cleavages=2&msparams_dir=msparams_mill/&hide_protein_sequence=2&database=PA_haloferax_mediterranei_genbank&seqdb_dir=D:\SeqDB\&enzyme=Trypsin&access_method=Accession+Number&accession_num=AFK20674.1&coverage_map=0+65+48+16) | 1.03e+008 |
| phosphate ABC transporter ATP-binding protein | [AFK20072.1](http://smserver.sti.ua.es/millhtml/SM_instruct/servadmn.htm#update_acclinks?AFK20072.1) | 6 | 6 | 82.61 | [23.3](http://smserver.sti.ua.es/millbin/msdigest.cgi?missed_cleavages=2&msparams_dir=msparams_mill/&hide_protein_sequence=2&database=PA_haloferax_mediterranei_genbank&seqdb_dir=D:\SeqDB\&enzyme=Trypsin&access_method=Accession+Number&accession_num=AFK20072.1&coverage_map=0+98+17+2+13+26+14+4+25+97) | 2.05e+006 |
| nucleoside diphosphate kinase | [AFK20428.1](http://smserver.sti.ua.es/millhtml/SM_instruct/servadmn.htm#update_acclinks?AFK20428.1) | 7 | 5 | 81.52 | [40.2](http://smserver.sti.ua.es/millbin/msdigest.cgi?missed_cleavages=2&msparams_dir=msparams_mill/&hide_protein_sequence=2&database=PA_haloferax_mediterranei_genbank&seqdb_dir=D:\SeqDB\&enzyme=Trypsin&access_method=Accession+Number&accession_num=AFK20428.1&coverage_map=0+6+21+62+25+16+16+8) | 1.55e+007 |
| thermosome, beta subunit | [AFK18158.2](http://smserver.sti.ua.es/millhtml/SM_instruct/servadmn.htm#update_acclinks?AFK18158.2) | 6 | 5 | 81.10 | [17.1](http://smserver.sti.ua.es/millbin/msdigest.cgi?missed_cleavages=2&msparams_dir=msparams_mill/&hide_protein_sequence=2&database=PA_haloferax_mediterranei_genbank&seqdb_dir=D:\SeqDB\&enzyme=Trypsin&access_method=Accession+Number&accession_num=AFK18158.2&coverage_map=0+328+21+5+24+12+32+28+18+86) | 1.30e+006 |
| gas-vesicle operon protein gvpA | [AFK19402.1](http://smserver.sti.ua.es/millhtml/SM_instruct/servadmn.htm#update_acclinks?AFK19402.1) | 4 | 4 | 79.01 | [57.6](http://smserver.sti.ua.es/millbin/msdigest.cgi?missed_cleavages=2&msparams_dir=msparams_mill/&hide_protein_sequence=2&database=PA_haloferax_mediterranei_genbank&seqdb_dir=D:\SeqDB\&enzyme=Trypsin&access_method=Accession+Number&accession_num=AFK19402.1&coverage_map=0+15+45+18) | 1.43e+007 |
| 3-hydroxyacyl-CoA dehydrogenase | AFK19216.2 | 5 | 4 | 70.08 | 22.7 | 9.44e+006 |
| hypothetical protein HFX_2509 | [AFK20191.2](http://smserver.sti.ua.es/millhtml/SM_instruct/servadmn.htm#update_acclinks?AFK20191.2) | 5 | 5 | 67.93 | [17](http://smserver.sti.ua.es/millbin/msdigest.cgi?missed_cleavages=2&msparams_dir=msparams_mill/&hide_protein_sequence=2&database=PA_haloferax_mediterranei_genbank&seqdb_dir=D:\SeqDB\&enzyme=Trypsin&access_method=Accession+Number&accession_num=AFK20191.2&coverage_map=0+9+41+4+11+240) | 4.53e+006 |
| hypothetical protein HFX_0016 | [AFK17760.1](http://smserver.sti.ua.es/millhtml/SM_instruct/servadmn.htm#update_acclinks?AFK17760.1) | 5 | 5 | 67.77 | [29.1](http://smserver.sti.ua.es/millbin/msdigest.cgi?missed_cleavages=2&msparams_dir=msparams_mill/&hide_protein_sequence=2&database=PA_haloferax_mediterranei_genbank&seqdb_dir=D:\SeqDB\&enzyme=Trypsin&access_method=Accession+Number&accession_num=AFK17760.1&coverage_map=0+32+19+19+11+27+9+2+17+56) | 3.50e+006 |
| glycine dehydrogenase subunit 1 | [AFK20093.1](http://smserver.sti.ua.es/millhtml/SM_instruct/servadmn.htm#update_acclinks?AFK20093.1) | 5 | 5 | 67.52 | [15.4](http://smserver.sti.ua.es/millbin/msdigest.cgi?missed_cleavages=2&msparams_dir=msparams_mill/&hide_protein_sequence=2&database=PA_haloferax_mediterranei_genbank&seqdb_dir=D:\SeqDB\&enzyme=Trypsin&access_method=Accession+Number&accession_num=AFK20093.1&coverage_map=0+60+9+260+40+23+20+35) | 3.89e+006 |
| ABC-type dipeptide/oligopeptide/nickel transport system, substrate binding protein (plasmid) | [AFK20740.1](http://smserver.sti.ua.es/millhtml/SM_instruct/servadmn.htm#update_acclinks?AFK20740.1) | 4 | 4 | 66.15 | [10](http://smserver.sti.ua.es/millbin/msdigest.cgi?missed_cleavages=2&msparams_dir=msparams_mill/&hide_protein_sequence=2&database=PA_haloferax_mediterranei_genbank&seqdb_dir=D:\SeqDB\&enzyme=Trypsin&access_method=Accession+Number&accession_num=AFK20740.1&coverage_map=0+169+18+36+13+44+17+228+10+44) | 9.16e+005 |
| halocyanin precursor-like protein | [AFK18843.1](http://smserver.sti.ua.es/millhtml/SM_instruct/servadmn.htm#update_acclinks?AFK18843.1) | 6 | 4 | 63.83 | [40.3](http://smserver.sti.ua.es/millbin/msdigest.cgi?missed_cleavages=2&msparams_dir=msparams_mill/&hide_protein_sequence=2&database=PA_haloferax_mediterranei_genbank&seqdb_dir=D:\SeqDB\&enzyme=Trypsin&access_method=Accession+Number&accession_num=AFK18843.1&coverage_map=0+51+21+48+46) | 9.09e+007 |
| pterin-4-alpha-carbinolamine dehydratase | [AFK20006.1](http://smserver.sti.ua.es/millhtml/SM_instruct/servadmn.htm#update_acclinks?AFK20006.1) | 4 | 3 | 63.15 | [59.3](http://smserver.sti.ua.es/millbin/msdigest.cgi?missed_cleavages=2&msparams_dir=msparams_mill/&hide_protein_sequence=2&database=PA_haloferax_mediterranei_genbank&seqdb_dir=D:\SeqDB\&enzyme=Trypsin&access_method=Accession+Number&accession_num=AFK20006.1&coverage_map=0+27+33+7+12+3+9) | 4.90e+006 |
| acetyl-CoA acetyltransferase (plasmid) | [AFK21178.1](http://smserver.sti.ua.es/millhtml/SM_instruct/servadmn.htm#update_acclinks?AFK21178.1) | 5 | 4 | 63.14 | [19.8](http://smserver.sti.ua.es/millbin/msdigest.cgi?missed_cleavages=2&msparams_dir=msparams_mill/&hide_protein_sequence=2&database=PA_haloferax_mediterranei_genbank&seqdb_dir=D:\SeqDB\&enzyme=Trypsin&access_method=Accession+Number&accession_num=AFK21178.1&coverage_map=0+11+20+9+27+26+8+88+23+180) | 6.26e+006 |
| gluconate dehydratase | [AFK19260.1](http://smserver.sti.ua.es/millhtml/SM_instruct/servadmn.htm#update_acclinks?AFK19260.1) | 4 | 4 | 62.53 | [10.6](http://smserver.sti.ua.es/millbin/msdigest.cgi?missed_cleavages=2&msparams_dir=msparams_mill/&hide_protein_sequence=2&database=PA_haloferax_mediterranei_genbank&seqdb_dir=D:\SeqDB\&enzyme=Trypsin&access_method=Accession+Number&accession_num=AFK19260.1&coverage_map=0+18+12+67+8+28+13+28+11+227) | 2.42e+006 |
| Hemerythrin HHE cation binding region (plasmid) | [AFK20978.1](http://smserver.sti.ua.es/millhtml/SM_instruct/servadmn.htm#update_acclinks?AFK20978.1) | 5 | 4 | 62.30 | [24.4](http://smserver.sti.ua.es/millbin/msdigest.cgi?missed_cleavages=2&msparams_dir=msparams_mill/&hide_protein_sequence=2&database=PA_haloferax_mediterranei_genbank&seqdb_dir=D:\SeqDB\&enzyme=Trypsin&access_method=Accession+Number&accession_num=AFK20978.1&coverage_map=0+64+30+14+13+4+10+75+6+25) | 9.90e+006 |
| molybdopterin biosynthesis protein moeA | AFK20001.1 | 4 | 4 | 59.57 | 17.4 | 1.69e+006 |
| copper-binding plastocyanin like protein (plasmid) | [AFK20927.1](http://smserver.sti.ua.es/millhtml/SM_instruct/servadmn.htm#update_acclinks?AFK20927.1) | 4 | 3 | 59.55 | [36.9](http://smserver.sti.ua.es/millbin/msdigest.cgi?missed_cleavages=2&msparams_dir=msparams_mill/&hide_protein_sequence=2&database=PA_haloferax_mediterranei_genbank&seqdb_dir=D:\SeqDB\&enzyme=Trypsin&access_method=Accession+Number&accession_num=AFK20927.1&coverage_map=0+81+48+6+16+22) | 1.25e+007 |
| glutamate dehydrogenase (NAD(P)+) | AFK19225.1 | 4 | 4 | 58.56 | 12.4 | 1.77e+006 |
| putative NAD(P)H-dependent xylose reductase | [AFK18957.2](http://smserver.sti.ua.es/millhtml/SM_instruct/servadmn.htm#update_acclinks?AFK18957.2) | 4 | 4 | 57.78 | [13.8](http://smserver.sti.ua.es/millbin/msdigest.cgi?missed_cleavages=2&msparams_dir=msparams_mill/&hide_protein_sequence=2&database=PA_haloferax_mediterranei_genbank&seqdb_dir=D:\SeqDB\&enzyme=Trypsin&access_method=Accession+Number&accession_num=AFK18957.2&coverage_map=0+114+13+12+7+56+13+49+12+48) | 2.52e+006 |
| succinate dehydrogenase, subunit B (iron-sulfur protein) | [AFK20496.1](http://smserver.sti.ua.es/millhtml/SM_instruct/servadmn.htm#update_acclinks?AFK20496.1) | 4 | 4 | 56.65 | [17.4](http://smserver.sti.ua.es/millbin/msdigest.cgi?missed_cleavages=2&msparams_dir=msparams_mill/&hide_protein_sequence=2&database=PA_haloferax_mediterranei_genbank&seqdb_dir=D:\SeqDB\&enzyme=Trypsin&access_method=Accession+Number&accession_num=AFK20496.1&coverage_map=0+82+23+4+16+120+12+36) | 3.76e+006 |
| metallo-beta-lactamase superfamily protein | [AFK18753.1](http://smserver.sti.ua.es/millhtml/SM_instruct/servadmn.htm#update_acclinks?AFK18753.1) | 4 | 3 | 56.43 | [28.1](http://smserver.sti.ua.es/millbin/msdigest.cgi?missed_cleavages=2&msparams_dir=msparams_mill/&hide_protein_sequence=2&database=PA_haloferax_mediterranei_genbank&seqdb_dir=D:\SeqDB\&enzyme=Trypsin&access_method=Accession+Number&accession_num=AFK18753.1&coverage_map=0+43+34+32+29+86) | 4.41e+006 |
| hsp20-type chaperone | [AFK18151.1](http://smserver.sti.ua.es/millhtml/SM_instruct/servadmn.htm#update_acclinks?AFK18151.1) | 4 | 4 | 56.42 | [38.5](http://smserver.sti.ua.es/millbin/msdigest.cgi?missed_cleavages=2&msparams_dir=msparams_mill/&hide_protein_sequence=2&database=PA_haloferax_mediterranei_genbank&seqdb_dir=D:\SeqDB\&enzyme=Trypsin&access_method=Accession+Number&accession_num=AFK18151.1&coverage_map=0+3+12+3+9+26+31+51) | 6.95e+006 |
| short-chain dehydrogenase / reductase SDR / glucose 1-dehydrogenase | [AFK20158.1](http://smserver.sti.ua.es/millhtml/SM_instruct/servadmn.htm#update_acclinks?AFK20158.1) | 5 | 4 | 56.05 | [21.2](http://smserver.sti.ua.es/millbin/msdigest.cgi?missed_cleavages=2&msparams_dir=msparams_mill/&hide_protein_sequence=2&database=PA_haloferax_mediterranei_genbank&seqdb_dir=D:\SeqDB\&enzyme=Trypsin&access_method=Accession+Number&accession_num=AFK20158.1&coverage_map=0+60+22+60+13+7+15+58) | 4.26e+006 |
| hypothetical protein HFX_0694 | [AFK18417.1](http://smserver.sti.ua.es/millhtml/SM_instruct/servadmn.htm#update_acclinks?AFK18417.1) | 3 | 3 | 55.83 | [46.3](http://smserver.sti.ua.es/millbin/msdigest.cgi?missed_cleavages=2&msparams_dir=msparams_mill/&hide_protein_sequence=2&database=PA_haloferax_mediterranei_genbank&seqdb_dir=D:\SeqDB\&enzyme=Trypsin&access_method=Accession+Number&accession_num=AFK18417.1&coverage_map=0+19+15+23+21+39+34) | 3.57e+006 |
| CBS domain-containing protein | [AFK17996.1](http://smserver.sti.ua.es/millhtml/SM_instruct/servadmn.htm#update_acclinks?AFK17996.1) | 3 | 3 | 54.45 | [8.8](http://smserver.sti.ua.es/millbin/msdigest.cgi?missed_cleavages=2&msparams_dir=msparams_mill/&hide_protein_sequence=2&database=PA_haloferax_mediterranei_genbank&seqdb_dir=D:\SeqDB\&enzyme=Trypsin&access_method=Accession+Number&accession_num=AFK17996.1&coverage_map=0+196+12+103+13+85+16+40) | 1.04e+006 |
| dTMP kinase | [AFK19952.1](http://smserver.sti.ua.es/millhtml/SM_instruct/servadmn.htm#update_acclinks?AFK19952.1) | 4 | 4 | 54.12 | [23.8](http://smserver.sti.ua.es/millbin/msdigest.cgi?missed_cleavages=2&msparams_dir=msparams_mill/&hide_protein_sequence=2&database=PA_haloferax_mediterranei_genbank&seqdb_dir=D:\SeqDB\&enzyme=Trypsin&access_method=Accession+Number&accession_num=AFK19952.1&coverage_map=0+71+16+5+9+24+14+22+8+28) | 2.55e+006 |
| poly(3-hydroxyalkanoate) granule-associated 12 kDa protein (plasmid) | [AFK21051.1](http://smserver.sti.ua.es/millhtml/SM_instruct/servadmn.htm#update_acclinks?AFK21051.1) | 3 | 3 | 54.09 | [34.5](http://smserver.sti.ua.es/millbin/msdigest.cgi?missed_cleavages=2&msparams_dir=msparams_mill/&hide_protein_sequence=2&database=PA_haloferax_mediterranei_genbank&seqdb_dir=D:\SeqDB\&enzyme=Trypsin&access_method=Accession+Number&accession_num=AFK21051.1&coverage_map=0+21+11+43+27+8) | 1.27e+007 |
| monoamine oxidase regulatory protein (plasmid) | [AFK21482.1](http://smserver.sti.ua.es/millhtml/SM_instruct/servadmn.htm#update_acclinks?AFK21482.1) | 3 | 3 | 53.89 | [26.9](http://smserver.sti.ua.es/millbin/msdigest.cgi?missed_cleavages=2&msparams_dir=msparams_mill/&hide_protein_sequence=2&database=PA_haloferax_mediterranei_genbank&seqdb_dir=D:\SeqDB\&enzyme=Trypsin&access_method=Accession+Number&accession_num=AFK21482.1&coverage_map=0+48+28+44+14+22) | 8.42e+006 |
| hypothetical protein HFX_1141 | [AFK18857.1](http://smserver.sti.ua.es/millhtml/SM_instruct/servadmn.htm#update_acclinks?AFK18857.1) | 5 | 4 | 53.70 | [48.5](http://smserver.sti.ua.es/millbin/msdigest.cgi?missed_cleavages=2&msparams_dir=msparams_mill/&hide_protein_sequence=2&database=PA_haloferax_mediterranei_genbank&seqdb_dir=D:\SeqDB\&enzyme=Trypsin&access_method=Accession+Number&accession_num=AFK18857.1&coverage_map=0+14+6+29+43+9) | 1.71e+007 |
| enoyl-CoA hydratase (plasmid) | [AFK21050.1](http://smserver.sti.ua.es/millhtml/SM_instruct/servadmn.htm#update_acclinks?AFK21050.1) | 4 | 3 | 53.66 | [15.5](http://smserver.sti.ua.es/millbin/msdigest.cgi?missed_cleavages=2&msparams_dir=msparams_mill/&hide_protein_sequence=2&database=PA_haloferax_mediterranei_genbank&seqdb_dir=D:\SeqDB\&enzyme=Trypsin&access_method=Accession+Number&accession_num=AFK21050.1&coverage_map=0+132+17+23+17+30) | 9.72e+006 |
| cobalamin adenosyltransferase | [AFK20083.1](http://smserver.sti.ua.es/millhtml/SM_instruct/servadmn.htm#update_acclinks?AFK20083.1) | 4 | 3 | 53.52 | [22](http://smserver.sti.ua.es/millbin/msdigest.cgi?missed_cleavages=2&msparams_dir=msparams_mill/&hide_protein_sequence=2&database=PA_haloferax_mediterranei_genbank&seqdb_dir=D:\SeqDB\&enzyme=Trypsin&access_method=Accession+Number&accession_num=AFK20083.1&coverage_map=0+7+9+116+30+15) | 2.73e+006 |
| citrate (si)-synthase | [AFK18167.1](http://smserver.sti.ua.es/millhtml/SM_instruct/servadmn.htm#update_acclinks?AFK18167.1) | 3 | 3 | 53.24 | [12.4](http://smserver.sti.ua.es/millbin/msdigest.cgi?missed_cleavages=2&msparams_dir=msparams_mill/&hide_protein_sequence=2&database=PA_haloferax_mediterranei_genbank&seqdb_dir=D:\SeqDB\&enzyme=Trypsin&access_method=Accession+Number&accession_num=AFK18167.1&coverage_map=0+32+28+177+19+123) | 1.54e+006 |
| poly(3-hydroxyalkanoate) granule-associated protein(phasin) (plasmid) | AFK21052.1 | 7 | 3 | 51.48 | 20.1 | 1.24e+008 |
| UpsA domain-containing protein | [AFK19975.2](http://smserver.sti.ua.es/millhtml/SM_instruct/servadmn.htm#update_acclinks?AFK19975.2) | 3 | 3 | 50.09 | [15.4](http://smserver.sti.ua.es/millbin/msdigest.cgi?missed_cleavages=2&msparams_dir=msparams_mill/&hide_protein_sequence=2&database=PA_haloferax_mediterranei_genbank&seqdb_dir=D:\SeqDB\&enzyme=Trypsin&access_method=Accession+Number&accession_num=AFK19975.2&coverage_map=0+29+22+91) | 4.68e+006 |
| phosphonates ABC transporter ATP-binding protein | [AFK19846.1](http://smserver.sti.ua.es/millhtml/SM_instruct/servadmn.htm#update_acclinks?AFK19846.1) | 4 | 3 | 49.64 | [19.3](http://smserver.sti.ua.es/millbin/msdigest.cgi?missed_cleavages=2&msparams_dir=msparams_mill/&hide_protein_sequence=2&database=PA_haloferax_mediterranei_genbank&seqdb_dir=D:\SeqDB\&enzyme=Trypsin&access_method=Accession+Number&accession_num=AFK19846.1&coverage_map=0+93+11+28+19+64+21+28) | 2.05e+006 |
| halocyanin hcpG | [AFK18949.1](http://smserver.sti.ua.es/millhtml/SM_instruct/servadmn.htm#update_acclinks?AFK18949.1) | 4 | 4 | 48.68 | [5.1](http://smserver.sti.ua.es/millbin/msdigest.cgi?missed_cleavages=2&msparams_dir=msparams_mill/&hide_protein_sequence=2&database=PA_haloferax_mediterranei_genbank&seqdb_dir=D:\SeqDB\&enzyme=Trypsin&access_method=Accession+Number&accession_num=AFK18949.1&coverage_map=0+70+12+6+8+312+10+190+12+198) | 1.39e+006 |
| flavin-dependent dehydrogenase | [AFK20419.1](http://smserver.sti.ua.es/millhtml/SM_instruct/servadmn.htm#update_acclinks?AFK20419.1) | 3 | 3 | 48.68 | [8.6](http://smserver.sti.ua.es/millbin/msdigest.cgi?missed_cleavages=2&msparams_dir=msparams_mill/&hide_protein_sequence=2&database=PA_haloferax_mediterranei_genbank&seqdb_dir=D:\SeqDB\&enzyme=Trypsin&access_method=Accession+Number&accession_num=AFK20419.1&coverage_map=0+148+29+18+13+165+6+175) | 9.74e+005 |
| hypothetical protein HFX_1777 | AFK19482.1 | 4 | 3 | 47.63 | 26.2 | 2.01e+007 |
| protein of unknown function DUF1486 | [AFK20429.1](http://smserver.sti.ua.es/millhtml/SM_instruct/servadmn.htm#update_acclinks?AFK20429.1) | 3 | 3 | 47.38 | [22.4](http://smserver.sti.ua.es/millbin/msdigest.cgi?missed_cleavages=2&msparams_dir=msparams_mill/&hide_protein_sequence=2&database=PA_haloferax_mediterranei_genbank&seqdb_dir=D:\SeqDB\&enzyme=Trypsin&access_method=Accession+Number&accession_num=AFK20429.1&coverage_map=0+54+11+42+11+5+15+27) | 1.34e+006 |
| geranylgeranyl hydrogenase-like protein / electron-transferring-flavoprotein dehydrogenase | [AFK19181.1](http://smserver.sti.ua.es/millhtml/SM_instruct/servadmn.htm#update_acclinks?AFK19181.1) | 3 | 3 | 47.03 | [8.1](http://smserver.sti.ua.es/millbin/msdigest.cgi?missed_cleavages=2&msparams_dir=msparams_mill/&hide_protein_sequence=2&database=PA_haloferax_mediterranei_genbank&seqdb_dir=D:\SeqDB\&enzyme=Trypsin&access_method=Accession+Number&accession_num=AFK19181.1&coverage_map=0+265+8+151+15+2+14+1) | 1.56e+006 |
| ribose-1,5-bisphosphate isomerase (ribulose-bisphosphate forming) | [AFK18682.1](http://smserver.sti.ua.es/millhtml/SM_instruct/servadmn.htm#update_acclinks?AFK18682.1) | 4 | 4 | 46.44 | [13.2](http://smserver.sti.ua.es/millbin/msdigest.cgi?missed_cleavages=2&msparams_dir=msparams_mill/&hide_protein_sequence=2&database=PA_haloferax_mediterranei_genbank&seqdb_dir=D:\SeqDB\&enzyme=Trypsin&access_method=Accession+Number&accession_num=AFK18682.1&coverage_map=0+11+22+47+11+190+9+27) | 1.50e+006 |
| stress response protein | AFK20336.2 | 3 | 3 | 45.54 | 15.6 | 1.68e+006 |
| hypothetical protein HFX_5226 (plasmid) | [AFK21058.1](http://smserver.sti.ua.es/millhtml/SM_instruct/servadmn.htm#update_acclinks?AFK21058.1) | 3 | 3 | 45.49 | [48.5](http://smserver.sti.ua.es/millbin/msdigest.cgi?missed_cleavages=2&msparams_dir=msparams_mill/&hide_protein_sequence=2&database=PA_haloferax_mediterranei_genbank&seqdb_dir=D:\SeqDB\&enzyme=Trypsin&access_method=Accession+Number&accession_num=AFK21058.1&coverage_map=0+29+49+23) | 9.32e+005 |
| thiamine-binding periplasmic protein precursor-like protein | [AFK17765.2](http://smserver.sti.ua.es/millhtml/SM_instruct/servadmn.htm#update_acclinks?AFK17765.2) | 4 | 3 | 45.35 | [14.2](http://smserver.sti.ua.es/millbin/msdigest.cgi?missed_cleavages=2&msparams_dir=msparams_mill/&hide_protein_sequence=2&database=PA_haloferax_mediterranei_genbank&seqdb_dir=D:\SeqDB\&enzyme=Trypsin&access_method=Accession+Number&accession_num=AFK17765.2&coverage_map=0+185+36+105+18+34) | 1.80e+006 |
| putative phosphonate ABC transporter, periplasmic phosphonate-binding protein | [AFK19847.1](http://smserver.sti.ua.es/millhtml/SM_instruct/servadmn.htm#update_acclinks?AFK19847.1) | 3 | 2 | 45.07 | [10.1](http://smserver.sti.ua.es/millbin/msdigest.cgi?missed_cleavages=2&msparams_dir=msparams_mill/&hide_protein_sequence=2&database=PA_haloferax_mediterranei_genbank&seqdb_dir=D:\SeqDB\&enzyme=Trypsin&access_method=Accession+Number&accession_num=AFK19847.1&coverage_map=0+205+19+106+20+33) | 1.13e+006 |
| hypothetical protein HFX_2088 | [AFK19779.1](http://smserver.sti.ua.es/millhtml/SM_instruct/servadmn.htm#update_acclinks?AFK19779.1) | 3 | 3 | 44.78 | [11.4](http://smserver.sti.ua.es/millbin/msdigest.cgi?missed_cleavages=2&msparams_dir=msparams_mill/&hide_protein_sequence=2&database=PA_haloferax_mediterranei_genbank&seqdb_dir=D:\SeqDB\&enzyme=Trypsin&access_method=Accession+Number&accession_num=AFK19779.1&coverage_map=0+200+10+76+19+54+15+11) | 6.54e+005 |
| NAD synthetase | AFK19658.1 | 4 | 3 | 44.15 | 10.1 | 1.62e+006 |
| serine protease inhibitor family protein | [AFK20013.1](http://smserver.sti.ua.es/millhtml/SM_instruct/servadmn.htm#update_acclinks?AFK20013.1) | 3 | 3 | 44.03 | [7.5](http://smserver.sti.ua.es/millbin/msdigest.cgi?missed_cleavages=2&msparams_dir=msparams_mill/&hide_protein_sequence=2&database=PA_haloferax_mediterranei_genbank&seqdb_dir=D:\SeqDB\&enzyme=Trypsin&access_method=Accession+Number&accession_num=AFK20013.1&coverage_map=0+192+12+118+10+98+12+11) | 4.08e+006 |
| 4-alpha-glucanotransferase | [AFK19479.2](http://smserver.sti.ua.es/millhtml/SM_instruct/servadmn.htm#update_acclinks?AFK19479.2) | 3 | 3 | 43.94 | [5.4](http://smserver.sti.ua.es/millbin/msdigest.cgi?missed_cleavages=2&msparams_dir=msparams_mill/&hide_protein_sequence=2&database=PA_haloferax_mediterranei_genbank&seqdb_dir=D:\SeqDB\&enzyme=Trypsin&access_method=Accession+Number&accession_num=AFK19479.2&coverage_map=0+359+7+109+20+1) | 1.84e+006 |
| hypothetical protein HFX_2910 | [AFK20580.2](http://smserver.sti.ua.es/millhtml/SM_instruct/servadmn.htm#update_acclinks?AFK20580.2) | 3 | 3 | 43.42 | [36](http://smserver.sti.ua.es/millbin/msdigest.cgi?missed_cleavages=2&msparams_dir=msparams_mill/&hide_protein_sequence=2&database=PA_haloferax_mediterranei_genbank&seqdb_dir=D:\SeqDB\&enzyme=Trypsin&access_method=Accession+Number&accession_num=AFK20580.2&coverage_map=0+37+14+37+16+13+19) | 6.96e+005 |
| S-adenosylmethionine-dependent methyltransferase-like protein | [AFK18456.1](http://smserver.sti.ua.es/millhtml/SM_instruct/servadmn.htm#update_acclinks?AFK18456.1) | 3 | 3 | 43.13 | [11.9](http://smserver.sti.ua.es/millbin/msdigest.cgi?missed_cleavages=2&msparams_dir=msparams_mill/&hide_protein_sequence=2&database=PA_haloferax_mediterranei_genbank&seqdb_dir=D:\SeqDB\&enzyme=Trypsin&access_method=Accession+Number&accession_num=AFK18456.1&coverage_map=0+46+11+72+7+17+12+87) | 1.48e+006 |
| ubiquinone/menaquinone biosynthesis methyltransferase | [AFK18034.2](http://smserver.sti.ua.es/millhtml/SM_instruct/servadmn.htm#update_acclinks?AFK18034.2) | 3 | 2 | 41.83 | [16.3](http://smserver.sti.ua.es/millbin/msdigest.cgi?missed_cleavages=2&msparams_dir=msparams_mill/&hide_protein_sequence=2&database=PA_haloferax_mediterranei_genbank&seqdb_dir=D:\SeqDB\&enzyme=Trypsin&access_method=Accession+Number&accession_num=AFK18034.2&coverage_map=0+48+17+108+17+18) | 1.65e+006 |
| hypothetical protein HFX_2082 | [AFK19774.1](http://smserver.sti.ua.es/millhtml/SM_instruct/servadmn.htm#update_acclinks?AFK19774.1) | 3 | 3 | 41.64 | [21.2](http://smserver.sti.ua.es/millbin/msdigest.cgi?missed_cleavages=2&msparams_dir=msparams_mill/&hide_protein_sequence=2&database=PA_haloferax_mediterranei_genbank&seqdb_dir=D:\SeqDB\&enzyme=Trypsin&access_method=Accession+Number&accession_num=AFK19774.1&coverage_map=0+51+12+27+18+1+12+77) | 7.43e+005 |
| gas-vesicle operon protein gvpF | [AFK19405.1](http://smserver.sti.ua.es/millhtml/SM_instruct/servadmn.htm#update_acclinks?AFK19405.1) | 3 | 3 | 41.53 | [18.3](http://smserver.sti.ua.es/millbin/msdigest.cgi?missed_cleavages=2&msparams_dir=msparams_mill/&hide_protein_sequence=2&database=PA_haloferax_mediterranei_genbank&seqdb_dir=D:\SeqDB\&enzyme=Trypsin&access_method=Accession+Number&accession_num=AFK19405.1&coverage_map=0+28+7+67+15+21+17+58) | 1.31e+006 |
| hypothetical protein HFX_0141 | [AFK17882.2](http://smserver.sti.ua.es/millhtml/SM_instruct/servadmn.htm#update_acclinks?AFK17882.2) | 2 | 2 | 40.97 | [37.3](http://smserver.sti.ua.es/millbin/msdigest.cgi?missed_cleavages=2&msparams_dir=msparams_mill/&hide_protein_sequence=2&database=PA_haloferax_mediterranei_genbank&seqdb_dir=D:\SeqDB\&enzyme=Trypsin&access_method=Accession+Number&accession_num=AFK17882.2&coverage_map=0+38+25+4) | 2.71e+006 |
| aconitate hydratase | [AFK18238.1](http://smserver.sti.ua.es/millhtml/SM_instruct/servadmn.htm#update_acclinks?AFK18238.1) | 3 | 3 | 40.92 | [3.5](http://smserver.sti.ua.es/millbin/msdigest.cgi?missed_cleavages=2&msparams_dir=msparams_mill/&hide_protein_sequence=2&database=PA_haloferax_mediterranei_genbank&seqdb_dir=D:\SeqDB\&enzyme=Trypsin&access_method=Accession+Number&accession_num=AFK18238.1&coverage_map=0+44+8+280+9+56+16+513) | 1.22e+006 |
| thymidylate synthase | [AFK20583.1](http://smserver.sti.ua.es/millhtml/SM_instruct/servadmn.htm#update_acclinks?AFK20583.1) | 3 | 3 | 40.76 | [14.5](http://smserver.sti.ua.es/millbin/msdigest.cgi?missed_cleavages=2&msparams_dir=msparams_mill/&hide_protein_sequence=2&database=PA_haloferax_mediterranei_genbank&seqdb_dir=D:\SeqDB\&enzyme=Trypsin&access_method=Accession+Number&accession_num=AFK20583.1&coverage_map=0+113+12+57+8+77+29+40) | 1.00e+006 |
| molecular chaperone DnaK | [AFK19355.1](http://smserver.sti.ua.es/millhtml/SM_instruct/servadmn.htm#update_acclinks?AFK19355.1) | 2 | 2 | 40.67 | [5.1](http://smserver.sti.ua.es/millbin/msdigest.cgi?missed_cleavages=2&msparams_dir=msparams_mill/&hide_protein_sequence=2&database=PA_haloferax_mediterranei_genbank&seqdb_dir=D:\SeqDB\&enzyme=Trypsin&access_method=Accession+Number&accession_num=AFK19355.1&coverage_map=0+398+15+106+17+89) | 7.73e+005 |
| hypothetical protein HFX_1239 | [AFK18952.1](http://smserver.sti.ua.es/millhtml/SM_instruct/servadmn.htm#update_acclinks?AFK18952.1) | 2 | 2 | 40.38 | [17.6](http://smserver.sti.ua.es/millbin/msdigest.cgi?missed_cleavages=2&msparams_dir=msparams_mill/&hide_protein_sequence=2&database=PA_haloferax_mediterranei_genbank&seqdb_dir=D:\SeqDB\&enzyme=Trypsin&access_method=Accession+Number&accession_num=AFK18952.1&coverage_map=0+50+17+28+17+81) | 1.08e+006 |
| NADH dehydrogenase/oxidoreductase-like protein | [AFK19950.1](http://smserver.sti.ua.es/millhtml/SM_instruct/servadmn.htm#update_acclinks?AFK19950.1) | 2 | 2 | 40.00 | [10.2](http://smserver.sti.ua.es/millbin/msdigest.cgi?missed_cleavages=2&msparams_dir=msparams_mill/&hide_protein_sequence=2&database=PA_haloferax_mediterranei_genbank&seqdb_dir=D:\SeqDB\&enzyme=Trypsin&access_method=Accession+Number&accession_num=AFK19950.1&coverage_map=0+107+19+96+12+67) | 8.02e+005 |
| phosphate ABC transporter periplasmic substrate-binding protein | [AFK20069.1](http://smserver.sti.ua.es/millhtml/SM_instruct/servadmn.htm#update_acclinks?AFK20069.1) | 3 | 3 | 39.24 | [7.5](http://smserver.sti.ua.es/millbin/msdigest.cgi?missed_cleavages=2&msparams_dir=msparams_mill/&hide_protein_sequence=2&database=PA_haloferax_mediterranei_genbank&seqdb_dir=D:\SeqDB\&enzyme=Trypsin&access_method=Accession+Number&accession_num=AFK20069.1&coverage_map=0+163+10+52+7+103+9) | 2.98e+006 |
| acyl-CoA dehydrogenase | [AFK20404.1](http://smserver.sti.ua.es/millhtml/SM_instruct/servadmn.htm#update_acclinks?AFK20404.1) | 3 | 3 | 39.11 | [13.1](http://smserver.sti.ua.es/millbin/msdigest.cgi?missed_cleavages=2&msparams_dir=msparams_mill/&hide_protein_sequence=2&database=PA_haloferax_mediterranei_genbank&seqdb_dir=D:\SeqDB\&enzyme=Trypsin&access_method=Accession+Number&accession_num=AFK20404.1&coverage_map=0+245+25+16+12+61+13+9) | 7.59e+005 |
| hypothetical protein HFX_0940 | [AFK18659.1](http://smserver.sti.ua.es/millhtml/SM_instruct/servadmn.htm#update_acclinks?AFK18659.1) | 2 | 2 | 38.79 | [9.4](http://smserver.sti.ua.es/millbin/msdigest.cgi?missed_cleavages=2&msparams_dir=msparams_mill/&hide_protein_sequence=2&database=PA_haloferax_mediterranei_genbank&seqdb_dir=D:\SeqDB\&enzyme=Trypsin&access_method=Accession+Number&accession_num=AFK18659.1&coverage_map=0+45+13+73+12+121) | 1.19e+006 |
| dihydrolipoamide S-acyltransferase (pyruvate dehydrogenase E2 component) | [AFK20618.1](http://smserver.sti.ua.es/millhtml/SM_instruct/servadmn.htm#update_acclinks?AFK20618.1) | 3 | 3 | 38.71 | [7.4](http://smserver.sti.ua.es/millbin/msdigest.cgi?missed_cleavages=2&msparams_dir=msparams_mill/&hide_protein_sequence=2&database=PA_haloferax_mediterranei_genbank&seqdb_dir=D:\SeqDB\&enzyme=Trypsin&access_method=Accession+Number&accession_num=AFK20618.1&coverage_map=0+124+17+311+9+1+11+27) | 6.64e+005 |
| thermosome, alpha subunit | [AFK17883.2](http://smserver.sti.ua.es/millhtml/SM_instruct/servadmn.htm#update_acclinks?AFK17883.2) | 3 | 3 | 38.58 | [5.7](http://smserver.sti.ua.es/millbin/msdigest.cgi?missed_cleavages=2&msparams_dir=msparams_mill/&hide_protein_sequence=2&database=PA_haloferax_mediterranei_genbank&seqdb_dir=D:\SeqDB\&enzyme=Trypsin&access_method=Accession+Number&accession_num=AFK17883.2&coverage_map=0+358+10+7+22+163) | 1.44e+006 |
| hypothetical protein HFX_0080 | [AFK17822.1](http://smserver.sti.ua.es/millhtml/SM_instruct/servadmn.htm#update_acclinks?AFK17822.1) | 2 | 2 | 38.21 | [26.6](http://smserver.sti.ua.es/millbin/msdigest.cgi?missed_cleavages=2&msparams_dir=msparams_mill/&hide_protein_sequence=2&database=PA_haloferax_mediterranei_genbank&seqdb_dir=D:\SeqDB\&enzyme=Trypsin&access_method=Accession+Number&accession_num=AFK17822.1&coverage_map=0+71+28+6) | 3.30e+005 |
| hypothetical protein HFX_6434 (plasmid) | [AFK21553.1](http://smserver.sti.ua.es/millhtml/SM_instruct/servadmn.htm#update_acclinks?AFK21553.1) | 3 | 3 | 38.04 | [10.3](http://smserver.sti.ua.es/millbin/msdigest.cgi?missed_cleavages=2&msparams_dir=msparams_mill/&hide_protein_sequence=2&database=PA_haloferax_mediterranei_genbank&seqdb_dir=D:\SeqDB\&enzyme=Trypsin&access_method=Accession+Number&accession_num=AFK21553.1&coverage_map=0+69+10+14+9+181+18+58) | 1.87e+006 |
| hsp20-type chaperone (plasmid) | [AFK20982.1](http://smserver.sti.ua.es/millhtml/SM_instruct/servadmn.htm#update_acclinks?AFK20982.1) | 2 | 2 | 37.79 | [19](http://smserver.sti.ua.es/millbin/msdigest.cgi?missed_cleavages=2&msparams_dir=msparams_mill/&hide_protein_sequence=2&database=PA_haloferax_mediterranei_genbank&seqdb_dir=D:\SeqDB\&enzyme=Trypsin&access_method=Accession+Number&accession_num=AFK20982.1&coverage_map=0+66+17+3+11+50) | 7.72e+005 |
| hypothetical protein HFX_2289 | AFK19976.1 | 2 | 2 | 37.43 | 35.2 | 1.53e+006 |
| oxidoreductase (thioredoxin-disulfide reductase-like protein) | [AFK19539.2](http://smserver.sti.ua.es/millhtml/SM_instruct/servadmn.htm#update_acclinks?AFK19539.2) | 3 | 3 | 36.92 | [10.5](http://smserver.sti.ua.es/millbin/msdigest.cgi?missed_cleavages=2&msparams_dir=msparams_mill/&hide_protein_sequence=2&database=PA_haloferax_mediterranei_genbank&seqdb_dir=D:\SeqDB\&enzyme=Trypsin&access_method=Accession+Number&accession_num=AFK19539.2&coverage_map=0+101+9+51+15+105+9+24) | 9.46e+005 |
| dnaJ/dnaK ATPase stimulator grpE | AFK19357.1 | 2 | 2 | 35.99 | 19.4 | 8.83e+005 |
| NADH dehydrogenase, subunit H (ubiquinone) | [AFK18697.1](http://smserver.sti.ua.es/millhtml/SM_instruct/servadmn.htm#update_acclinks?AFK18697.1) | 3 | 2 | 35.68 | [7.1](http://smserver.sti.ua.es/millbin/msdigest.cgi?missed_cleavages=2&msparams_dir=msparams_mill/&hide_protein_sequence=2&database=PA_haloferax_mediterranei_genbank&seqdb_dir=D:\SeqDB\&enzyme=Trypsin&access_method=Accession+Number&accession_num=AFK18697.1&coverage_map=0+65+15+238+10+21) | 3.62e+006 |
| phytoene dehydrogenase (phytoene desaturase) | [AFK18509.1](http://smserver.sti.ua.es/millhtml/SM_instruct/servadmn.htm#update_acclinks?AFK18509.1) | 3 | 3 | 35.56 | [8.4](http://smserver.sti.ua.es/millbin/msdigest.cgi?missed_cleavages=2&msparams_dir=msparams_mill/&hide_protein_sequence=2&database=PA_haloferax_mediterranei_genbank&seqdb_dir=D:\SeqDB\&enzyme=Trypsin&access_method=Accession+Number&accession_num=AFK18509.1&coverage_map=0+20+14+58+8+32+14+280) | 1.18e+006 |
| glutamate dehydrogenase (NAD(P)+) | [AFK19867.1](http://smserver.sti.ua.es/millhtml/SM_instruct/servadmn.htm#update_acclinks?AFK19867.1) | 3 | 3 | 35.54 | [7.5](http://smserver.sti.ua.es/millbin/msdigest.cgi?missed_cleavages=2&msparams_dir=msparams_mill/&hide_protein_sequence=2&database=PA_haloferax_mediterranei_genbank&seqdb_dir=D:\SeqDB\&enzyme=Trypsin&access_method=Accession+Number&accession_num=AFK19867.1&coverage_map=0+150+6+233+17+11+10+12) | 5.63e+005 |
| glycerol kinase | [AFK19308.1](http://smserver.sti.ua.es/millhtml/SM_instruct/servadmn.htm#update_acclinks?AFK19308.1) | 2 | 2 | 35.49 | [5.6](http://smserver.sti.ua.es/millbin/msdigest.cgi?missed_cleavages=2&msparams_dir=msparams_mill/&hide_protein_sequence=2&database=PA_haloferax_mediterranei_genbank&seqdb_dir=D:\SeqDB\&enzyme=Trypsin&access_method=Accession+Number&accession_num=AFK19308.1&coverage_map=0+389+29+94) | 4.37e+006 |
| dipeptide/oligopeptide/nickel ABC transporter ATP-binding protein | [AFK17802.1](http://smserver.sti.ua.es/millhtml/SM_instruct/servadmn.htm#update_acclinks?AFK17802.1) | 3 | 3 | 35.47 | [5.7](http://smserver.sti.ua.es/millbin/msdigest.cgi?missed_cleavages=2&msparams_dir=msparams_mill/&hide_protein_sequence=2&database=PA_haloferax_mediterranei_genbank&seqdb_dir=D:\SeqDB\&enzyme=Trypsin&access_method=Accession+Number&accession_num=AFK17802.1&coverage_map=0+80+11+250+15+99) | 1.14e+006 |
| 1-(5-phosphoribosyl)-5-[(5- phosphoribosylamino)methylideneamino] imidazole-4-carboxamide isomerase | [AFK20645.1](http://smserver.sti.ua.es/millhtml/SM_instruct/servadmn.htm#update_acclinks?AFK20645.1) | 2 | 2 | 35.11 | [13.8](http://smserver.sti.ua.es/millbin/msdigest.cgi?missed_cleavages=2&msparams_dir=msparams_mill/&hide_protein_sequence=2&database=PA_haloferax_mediterranei_genbank&seqdb_dir=D:\SeqDB\&enzyme=Trypsin&access_method=Accession+Number&accession_num=AFK20645.1&coverage_map=0+48+16+148+17+9) | 4.19e+005 |
| putative mechanosensitive ion channel | [AFK19964.1](http://smserver.sti.ua.es/millhtml/SM_instruct/servadmn.htm#update_acclinks?AFK19964.1) | 2 | 2 | 34.97 | [10.3](http://smserver.sti.ua.es/millbin/msdigest.cgi?missed_cleavages=2&msparams_dir=msparams_mill/&hide_protein_sequence=2&database=PA_haloferax_mediterranei_genbank&seqdb_dir=D:\SeqDB\&enzyme=Trypsin&access_method=Accession+Number&accession_num=AFK19964.1&coverage_map=0+190+17+36+14+43) | 1.07e+006 |
| NADH dehydrogenase, subunit B (ubiquinone) | [AFK18695.1](http://smserver.sti.ua.es/millhtml/SM_instruct/servadmn.htm#update_acclinks?AFK18695.1) | 4 | 2 | 34.69 | [11.5](http://smserver.sti.ua.es/millbin/msdigest.cgi?missed_cleavages=2&msparams_dir=msparams_mill/&hide_protein_sequence=2&database=PA_haloferax_mediterranei_genbank&seqdb_dir=D:\SeqDB\&enzyme=Trypsin&access_method=Accession+Number&accession_num=AFK18695.1&coverage_map=0+98+14+101+13+7) | 5.15e+006 |
| anthranilate phosphoribosyltransferase | [AFK19965.1](http://smserver.sti.ua.es/millhtml/SM_instruct/servadmn.htm#update_acclinks?AFK19965.1) | 2 | 2 | 33.87 | [7.3](http://smserver.sti.ua.es/millbin/msdigest.cgi?missed_cleavages=2&msparams_dir=msparams_mill/&hide_protein_sequence=2&database=PA_haloferax_mediterranei_genbank&seqdb_dir=D:\SeqDB\&enzyme=Trypsin&access_method=Accession+Number&accession_num=AFK19965.1&coverage_map=0+307+12+4+14+18) | 6.55e+005 |
| superoxide dismutase, Fe-Mn family (plasmid) | AFK21142.1 | 2 | 2 | 33.25 | 14 | 8.31e+005 |
| halocyanin hcpH | [AFK18872.1](http://smserver.sti.ua.es/millhtml/SM_instruct/servadmn.htm#update_acclinks?AFK18872.1) | 2 | 2 | 33.03 | [28.3](http://smserver.sti.ua.es/millbin/msdigest.cgi?missed_cleavages=2&msparams_dir=msparams_mill/&hide_protein_sequence=2&database=PA_haloferax_mediterranei_genbank&seqdb_dir=D:\SeqDB\&enzyme=Trypsin&access_method=Accession+Number&accession_num=AFK18872.1&coverage_map=0+71+42+35) | 5.49e+006 |
| branched-chain-amino-acid aminotransferase | [AFK18053.1](http://smserver.sti.ua.es/millhtml/SM_instruct/servadmn.htm#update_acclinks?AFK18053.1) | 3 | 2 | 32.88 | [8.9](http://smserver.sti.ua.es/millbin/msdigest.cgi?missed_cleavages=2&msparams_dir=msparams_mill/&hide_protein_sequence=2&database=PA_haloferax_mediterranei_genbank&seqdb_dir=D:\SeqDB\&enzyme=Trypsin&access_method=Accession+Number&accession_num=AFK18053.1&coverage_map=0+269+28+15) | 1.19e+006 |
| putative iron transport protein | [AFK19393.1](http://smserver.sti.ua.es/millhtml/SM_instruct/servadmn.htm#update_acclinks?AFK19393.1) | 2 | 2 | 32.87 | [6.6](http://smserver.sti.ua.es/millbin/msdigest.cgi?missed_cleavages=2&msparams_dir=msparams_mill/&hide_protein_sequence=2&database=PA_haloferax_mediterranei_genbank&seqdb_dir=D:\SeqDB\&enzyme=Trypsin&access_method=Accession+Number&accession_num=AFK19393.1&coverage_map=0+129+17+53+10+195) | 1.21e+006 |
| mechanosensitive ion channel | [AFK19459.1](http://smserver.sti.ua.es/millhtml/SM_instruct/servadmn.htm#update_acclinks?AFK19459.1) | 2 | 2 | 32.77 | [7.7](http://smserver.sti.ua.es/millbin/msdigest.cgi?missed_cleavages=2&msparams_dir=msparams_mill/&hide_protein_sequence=2&database=PA_haloferax_mediterranei_genbank&seqdb_dir=D:\SeqDB\&enzyme=Trypsin&access_method=Accession+Number&accession_num=AFK19459.1&coverage_map=0+68+14+68+8+125) | 5.11e+006 |
| hypothetical protein HFX_1529 | [AFK19236.1](http://smserver.sti.ua.es/millhtml/SM_instruct/servadmn.htm#update_acclinks?AFK19236.1) | 2 | 2 | 32.70 | [6.1](http://smserver.sti.ua.es/millbin/msdigest.cgi?missed_cleavages=2&msparams_dir=msparams_mill/&hide_protein_sequence=2&database=PA_haloferax_mediterranei_genbank&seqdb_dir=D:\SeqDB\&enzyme=Trypsin&access_method=Accession+Number&accession_num=AFK19236.1&coverage_map=0+135+13+34+12+213) | 8.22e+005 |
| hypothetical protein HFX_2772 | [AFK20448.1](http://smserver.sti.ua.es/millhtml/SM_instruct/servadmn.htm#update_acclinks?AFK20448.1) | 3 | 2 | 32.53 | [20.5](http://smserver.sti.ua.es/millbin/msdigest.cgi?missed_cleavages=2&msparams_dir=msparams_mill/&hide_protein_sequence=2&database=PA_haloferax_mediterranei_genbank&seqdb_dir=D:\SeqDB\&enzyme=Trypsin&access_method=Accession+Number&accession_num=AFK20448.1&coverage_map=0+81+21) | 4.39e+006 |
| membrane protein Pan1 | [AFK18234.1](http://smserver.sti.ua.es/millhtml/SM_instruct/servadmn.htm#update_acclinks?AFK18234.1) | 2 | 2 | 32.33 | [6.1](http://smserver.sti.ua.es/millbin/msdigest.cgi?missed_cleavages=2&msparams_dir=msparams_mill/&hide_protein_sequence=2&database=PA_haloferax_mediterranei_genbank&seqdb_dir=D:\SeqDB\&enzyme=Trypsin&access_method=Accession+Number&accession_num=AFK18234.1&coverage_map=0+223+14+136+12+36) | 2.90e+005 |
| riboflavin synthase beta subunit (6,7-dimethyl-8-ribityllumazine synthase) | [AFK18690.1](http://smserver.sti.ua.es/millhtml/SM_instruct/servadmn.htm#update_acclinks?AFK18690.1) | 2 | 2 | 32.21 | [29.8](http://smserver.sti.ua.es/millbin/msdigest.cgi?missed_cleavages=2&msparams_dir=msparams_mill/&hide_protein_sequence=2&database=PA_haloferax_mediterranei_genbank&seqdb_dir=D:\SeqDB\&enzyme=Trypsin&access_method=Accession+Number&accession_num=AFK18690.1&coverage_map=0+10+10+35+30+49) | 1.16e+006 |
| ferredoxin: NAD+ oxidoreductase | [AFK20041.1](http://smserver.sti.ua.es/millhtml/SM_instruct/servadmn.htm#update_acclinks?AFK20041.1) | 2 | 2 | 32.18 | [11.3](http://smserver.sti.ua.es/millbin/msdigest.cgi?missed_cleavages=2&msparams_dir=msparams_mill/&hide_protein_sequence=2&database=PA_haloferax_mediterranei_genbank&seqdb_dir=D:\SeqDB\&enzyme=Trypsin&access_method=Accession+Number&accession_num=AFK20041.1&coverage_map=0+102+32+229+15+35) | 7.57e+005 |
| phosphate transport system regulatory protein PhoU | [AFK20073.1](http://smserver.sti.ua.es/millhtml/SM_instruct/servadmn.htm#update_acclinks?AFK20073.1) | 2 | 2 | 32.13 | [15](http://smserver.sti.ua.es/millbin/msdigest.cgi?missed_cleavages=2&msparams_dir=msparams_mill/&hide_protein_sequence=2&database=PA_haloferax_mediterranei_genbank&seqdb_dir=D:\SeqDB\&enzyme=Trypsin&access_method=Accession+Number&accession_num=AFK20073.1&coverage_map=0+79+7+7+27+106) | 9.19e+005 |
| aspartate aminotransferase | [AFK18689.1](http://smserver.sti.ua.es/millhtml/SM_instruct/servadmn.htm#update_acclinks?AFK18689.1) | 2 | 2 | 32.00 | [8.8](http://smserver.sti.ua.es/millbin/msdigest.cgi?missed_cleavages=2&msparams_dir=msparams_mill/&hide_protein_sequence=2&database=PA_haloferax_mediterranei_genbank&seqdb_dir=D:\SeqDB\&enzyme=Trypsin&access_method=Accession+Number&accession_num=AFK18689.1&coverage_map=0+191+18+37+16+122) | 3.61e+005 |
| hypothetical protein HFX_1590 | [AFK19296.1](http://smserver.sti.ua.es/millhtml/SM_instruct/servadmn.htm#update_acclinks?AFK19296.1) | 2 | 2 | 31.95 | [6.4](http://smserver.sti.ua.es/millbin/msdigest.cgi?missed_cleavages=2&msparams_dir=msparams_mill/&hide_protein_sequence=2&database=PA_haloferax_mediterranei_genbank&seqdb_dir=D:\SeqDB\&enzyme=Trypsin&access_method=Accession+Number&accession_num=AFK19296.1&coverage_map=0+170+21+173+12+139) | 3.11e+006 |
| hypothetical protein HFX_5081 (plasmid) | [AFK20916.2](http://smserver.sti.ua.es/millhtml/SM_instruct/servadmn.htm#update_acclinks?AFK20916.2) | 2 | 2 | 31.79 | [8.5](http://smserver.sti.ua.es/millbin/msdigest.cgi?missed_cleavages=2&msparams_dir=msparams_mill/&hide_protein_sequence=2&database=PA_haloferax_mediterranei_genbank&seqdb_dir=D:\SeqDB\&enzyme=Trypsin&access_method=Accession+Number&accession_num=AFK20916.2&coverage_map=0+240+16+48+12+11) | 4.94e+005 |
| NAD-dependent epimerase/dehydratase | AFK18563.1 | 2 | 2 | 31.36 | 10.3 | 2.81e+005 |
| 3-oxoacyl-[acyl-carrier protein] reductase | [AFK19230.1](http://smserver.sti.ua.es/millhtml/SM_instruct/servadmn.htm#update_acclinks?AFK19230.1) | 3 | 2 | 31.22 | [9.9](http://smserver.sti.ua.es/millbin/msdigest.cgi?missed_cleavages=2&msparams_dir=msparams_mill/&hide_protein_sequence=2&database=PA_haloferax_mediterranei_genbank&seqdb_dir=D:\SeqDB\&enzyme=Trypsin&access_method=Accession+Number&accession_num=AFK19230.1&coverage_map=0+7+13+148+12+72) | 1.98e+006 |
| glutamine synthetase | [AFK17986.2](http://smserver.sti.ua.es/millhtml/SM_instruct/servadmn.htm#update_acclinks?AFK17986.2) | 2 | 2 | 30.77 | [7.6](http://smserver.sti.ua.es/millbin/msdigest.cgi?missed_cleavages=2&msparams_dir=msparams_mill/&hide_protein_sequence=2&database=PA_haloferax_mediterranei_genbank&seqdb_dir=D:\SeqDB\&enzyme=Trypsin&access_method=Accession+Number&accession_num=AFK17986.2&coverage_map=0+372+9+21+26+28) | 3.23e+005 |
| triphosphoribosyl-dephospho-CoA synthase | [AFK20166.1](http://smserver.sti.ua.es/millhtml/SM_instruct/servadmn.htm#update_acclinks?AFK20166.1) | 2 | 2 | 30.25 | [13.6](http://smserver.sti.ua.es/millbin/msdigest.cgi?missed_cleavages=2&msparams_dir=msparams_mill/&hide_protein_sequence=2&database=PA_haloferax_mediterranei_genbank&seqdb_dir=D:\SeqDB\&enzyme=Trypsin&access_method=Accession+Number&accession_num=AFK20166.1&coverage_map=0+193+15+3+24+50) | 4.43e+005 |
| hypothetical protein HFX_2180 | [AFK19869.1](http://smserver.sti.ua.es/millhtml/SM_instruct/servadmn.htm#update_acclinks?AFK19869.1) | 2 | 2 | 30.95 | [3.5](http://smserver.sti.ua.es/millbin/msdigest.cgi?missed_cleavages=2&msparams_dir=msparams_mill/&hide_protein_sequence=2&database=PA_haloferax_mediterranei_genbank&seqdb_dir=D:\SeqDB\&enzyme=Trypsin&access_method=Accession+Number&accession_num=AFK19869.1&coverage_map=0+220+7+17+9+202) | 8.84e+005 |
| cytochrome b/b6 (plasmid) | [AFK20941.1](http://smserver.sti.ua.es/millhtml/SM_instruct/servadmn.htm#update_acclinks?AFK20941.1) | 2 | 2 | 30.23 | [8.6](http://smserver.sti.ua.es/millbin/msdigest.cgi?missed_cleavages=2&msparams_dir=msparams_mill/&hide_protein_sequence=2&database=PA_haloferax_mediterranei_genbank&seqdb_dir=D:\SeqDB\&enzyme=Trypsin&access_method=Accession+Number&accession_num=AFK20941.1&coverage_map=0+431+42+12) | 7.00e+005 |

| MEL**KRK**TIA**K**VIAVVFIFNLVVMGAGAWFAYQEAPPIPE**K**VVGPDGEVIVNGEEI**R**DG**KK**VFQQNGLMNHGSILGNGAYYGVDYTADALEL**K**VQYM**R**DYYAQE**R**HGESYSALDSATQAAIADVVE**K**DLDGTYEGGAIEYSEAE**R**YAHEQV**R**QEYVQ**R**YHEGDHE**R**GVPVGMIDSEAEAEQFADFAMWTAWFSHTD**R**PGSTHSYTNDWPYQPGAGNDATAASMTWSVIAMVLLVAGAGLGIWLY**K**SVELPEPSAEGISVPEPGEVSIFPSQ**R**AAL**R**FIPVAAGLFVAQVLLGGLLAHFYIE**R**AGFFGIETLFGIHILQLLPFSIA**K**TWHIDLAILWIAATWLGAGLFLPPLLTGYEP**RK**QSTYINGLLGAIVVVTLGGLGGIWLGANGYIDGPLWWILGNEGLEYLEVG**K**LWQFGILAGFLIWAGLAV**R**GL**K**PLLD**K**EPVYGLAHMILYAGGSIALLFTAGFLFTPDTNIAVTEFW**R**WWVVHMWVEGAFEFFIVAIVGLTLVSMNLLS**RR**SAE**K**AVMLQALLVMGTGIIGVSHHYWWVGMPDMWVPLGSVFSTLELIPLVFILYEALGQY**R**TMSTGENFPY**R**LPFMFIIASGVWNFVGAGVLGFFINLPLINYYEHGTYLTVGHAHAAMFGAFGFLALGMVTYMLQLSIDPA**R**WDGSWL**R**AAFWCWNVGLVLMVFVSVLPVGFLQLETAFTGSYAAA**R**SLAFYNQPIIQTLFWA**R**LPGDTLMILGTVIYAADLV**RKR**FVL**R**ESSDDPSVEDMAVAEGILGDD |
| --- |

**A**

**B**

| **Sequence** | **MH^+^ Matched (Da)** | **Spectra** | **Spectral intensity** |
| --- | --- | --- | --- |
| (K)DLDGTYEGGAIEYSEAER(Y) | 1974.86 | 2 | 4.32E5 |
| (R)FIPVAAGLFVAQVLLGGLLAHFYIER(A) | 2814.61 | 1 | 4.71E5 |
| (R)HGESYSALDSATQAAIADVVEK(D) | 2262.09 | 1 | 4.51E5 |
| (R)LPGDTLMILGTVIYAADLVR(K) | 2131.18 | 3 | 1.35E6 |
| SLAFYNQPIIQTLFWAR(L) | 2068.10 | 1 | 4.27E5 |
| (K)VVGPDGEVIVNGEEIR(D) | 1681.88 | 1 | 1.07E6 |

**Figure S1.** Panel A: amino acid sequence of *Haloferax mediterranei* qNor. Lysine residues (K) and arginine residues (R) are shown in bold letters. The 14 transmembrane segments are shown in grey. Panel B: sequence of the six different peptides detected by LC-MS/MS after trypsin digestion of qNor in micelles of the last step of enrichment.

| Nir-NO --------MLSTTRRRTLQLLGLGGVASLAGCASEAPTAAQSLDQTEEPTPAQQESPKIV 52  Nir-Copper MRKYVGAPGSTMSRREFLAATGGAGIFGLAGCTAPTNEDSNAAVGTDDTTTAATDNS--- 57  : :**. * * .*: .****:: : ::: *:: * * :.  Nir-NO EQVAANPTDIPDPITRSEPTEVDVTLR---------PE-EVTAEVE--EGVTFTYMTYNG 100  Nir-Copper ----ALPYTSPPEVVQVDDQGGKVTLKSAPARHAVHPGESMGGPVELPQVWAFSADDGDP 113  * * * :.: : .***: * .: . ** : :*: :  Nir-NO QVPGPFIRVRQGDTVNLTFENPEENSMPHNVDFHAVAGPGG-----GAEATMTNPGETVK 155  Nir-Copper SVPGPILRTTEGNDMEVTLDNTD-GMRPHTVHFHGAQKAWKDDGVPTTTGIRVDPGEKHT 172  .****::*. :*: :::*::* : . **.*.**.. : . .:***. .  Nir-NO IRFKATYPGAYIYHCAVPNMDMHISAGMFGLILVEPPEGLPEVDKEVYIGQHELYTDKKA 215  Nir-Copper YTIPANVPGTHLYHCHYQT-HRHIEMGMYGIFRVDPK-GYEPADKEYFMTVRDWDSRLP- 229  : *. **:::*** . . **. **:*:: *:* * .*** :: :: :  Nir-NO GKKGKHNFDFEAMRNEEPTYVVMNGEKYAW-TDAGRGPAATVNTGETVRVFFVDGGPNLS 274  Nir-Copper RQMAGEDVSYD-PRNRKPDVFTVNGKSAPRTLHPEDGSPIIVEHGDKVRLHYVNAGYMS- 287  : . .:..:: **.:* ..:**:. . * *: *:.**:.:*:.*  Nir-NO SSFHPIGSVWETLYPDGSLSTDPQT--HIQTRLVPPGSTTVATMSSPVPGDFKLVDHSLS 332  Nir-Copper HPMHIHNHRFQLVEKDGGVIPEAARYEEDVTNIAPAERHTIEFTADSEPGIYLMHCHKVN 347  :* . :: : **.: : . *.:.* *: :. ** : : *.:.  Nir-NO RVTRKG-----CMAVIRAEGPEDPEIFDPNPE------- 359  Nir-Copper HVMNGDFYPGGMLGGVVYKEAMKSDIFSQLMDYAGYEPQ 386  :* . . :. : : . :**. : |
| --- |

**Figure S2.** Amino acid sequence alignments between nitrite reductase-NO forming (Nir-NO) and copper-containing nitrite reductase (Nir-Copper) using the bioinformatic tool Clustal Omega (Sievers, F., Wilm, A., Dineen, D., Gibson, T.J., Karplus, K., Li, W., *et al*., 2011). The percentage of identity was 23.30%.
